# Supplementary material for: Effective light cone and digital quantum simulation of interacting bosons
Source: Nat Commun. 2024 Mar 21;15:2520. doi: 10.1038/s41467-024-46501-7 (PMC10957968; doi:10.1038/s41467-024-46501-7)
Supplement: Supplementary file 1 — Supplementary Information [file 41467_2024_46501_MOESM1_ESM.pdf]

Supplementary Information for "Effective light cone and digital quantum simulation of interacting bosons"

Tomotaka Kuwahara[1,2,3], Tan Van Vu[1], and Keiji Saito[4]
[1] *Analytical quantum complexity RIKEN Hakubi Research Team, RIKEN Center for Quantum Computing (RQC), Wako, Saitama 351-0198, Japan*
[2] *RIKEN Cluster for Pioneering Research (CPR), Wako, Saitama 351-0198, Japan*
[3] *PRESTO, Japan Science and Technology (JST), Kawaguchi, Saitama 332-0012, Japan and*
[4] *Department of Physics, Kyoto University, Kyoto 606-8502, Japan*

CONTENTS

Supplementary Note 1. Set up 3
A. Often used inequalities 6
1. Proof of Lemma 1 6
B. Boson operators 7
C. Bose-Hubbard type Hamiltonian 7
1. Assumptions on the Hamiltonian 8
D. Time evolution 8
E. Definitions on the boson density 9
1. Basic lemmas on the boson density 9
2. Proof of Lemma 2 10
3. Proof of Lemma 3 11

Supplementary Note 2. Upper bound on moment function after short-time evolution 12
A. Statement 12
1. Effective Hamiltonian 13
2. Imaginary time evolution 13
B. Proof of Subtheorem 1 14
1. Proof of Proposition 6 14
2. Proof of Lemma 7 16
3. Proof of Proposition 8 17
4. Proof of Lemma 9 21
C. More general statement 23
1. Proof of Proposition 10 23
2. Proof of Lemma 11 24
D. Lower bound on the moment function 26
1. Proof of Corollary 12 26

Supplementary Note 3. Refined moment bounds after short-time evolution 26
A. Strategy for refined bounds 26
B. Convenient lemmas 27
1. Proof of Lemma 13 28
2. Proof of Lemma 14 30
3. Proof of Lemma 15 31
C. Moment upper bound: 1st order 32
D. Proof of Proposition 16 32
1. Proof of Lemma 17 34
E. Moment upper bound: higher order 35
1. Difficulty for the generalization 35
2. Main results 36
3. Proof of Proposition 18 36

Supplementary Note 4. Main theorem on the boson concentration 37
A. Proof of Theorem 1 38
B. Proof of Proposition 19 39
C. Boson density after time evolution for an initial state with low-boson density 41
1. Proof of Proposition 20 42

Supplementary Note 5. Restriction on the outflow of bosons 43
A. Moment lower bound 44

1. Proof of Lemma 21 45
B. Bound on the probability distribution 46
C. Proof of Proposition 22 47
1. Proof of Lemma 24 49

Supplementary Note 6. Effective Hamiltonian theory 51
1. Proof of Lemma 25 52
A. Probability distribution for boson number after time evolution 54
1. Proof of Lemma 28 55
B. Effective Hamiltonian by the truncation of the boson number: first result 56
1. Proof of Proposition 29 57
C. Effective Hamiltonian by the truncation of the boson number: second result 58
1. Proof of Proposition 30 59
D. Effective Hamiltonian by the truncation of the boson number: third result 61
1. Proof of Proposition 32 63

Supplementary Note 7. Bosonic Lieb-Robinson bound: looser results 65
A. Proof of Theorem 2: basic strategy 65
B. Proof of Lemma 33: derivation of the Lieb-Robinson bound 67

Supplementary Note 8. Main subtheorem on small time evolution 70
A. Proof outline 70
B. Completing the proof of Subtheorem 2 73
C. Proof of Proposition 36: spectral constraint for construction of effective Hamiltonian 73
1. Proof of Lemma 37 74
2. Proof of Lemma 38 76
3. Proof of Corollary 39 77
D. Proof of Proposition 36: Error estimation for the effective Hamiltonian 78
1. Proof of Lemma 40 79
2. Proof of Lemma 41 80
E. Proof of Proposition 36: Lieb-Robinson bound for effective Hamiltonian 81
1. Proof of Lemma 42 83
2. Proof of Corollary 43 86
F. Generalization: small time evolution by effective Hamiltonian 87
G. Proof of Proposition 44 88

Supplementary Note 9. Optimal Lieb-Robinson bound: high-dimensional cases 92
A. Proof of Theorem 3: preparation 93
B. Proof of Theorem 3 93
C. Proof of Proposition 45 95
1. Proof of Lemma 46 98

Supplementary Note 10. Lieb-Robinson bound in one dimensional systems 100
A. Preparation 101
B. Main proposition and the proof 102
C. Proof of Proposition 48: refined decomposition of the time evolution 102
D. Proof of Proposition 48: inserting projections 104
E. Proof of Proposition 48: estimation of the decomposed operators $\Delta\tilde{O}_{\mathbf{s}_m,\boldsymbol{\sigma}_m}$ 106
1. Proof of Lemma 49 107
2. Proof of Lemma 51 109
F. Proof of Proposition 48: completing the proof 113

Supplementary Note 11. Possible Scenario for Acceleration by Time-Independent Hamiltonian 113

Supplementary References 115

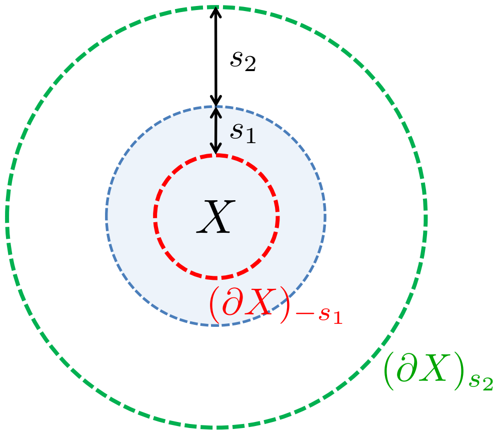

Supplementary Figure 1. Schematic picture of the definition of $(\partial X)_s$ for positive and negative s.

Supplementary Note 1. SET UP

We first show the detailed setup, which partially overlaps with the one in the main text. We consider a quantum system on a D dimensional lattice (graph), where the bosons interact with each other. The unbounded number of bosons can sit on each site; hence, the local Hilbert dimension is infinitely large. We denote Λ by the set of total sites and for an arbitrary partial set $X \subseteq \Lambda$, we represent the cardinality (the number of sites contained in X) by $|X|$. We also denote the complementary subset and the surface subset of X by $X^{\rm c} := \Lambda \setminus X$ and $\partial X := \{i \in X | d_{i,X^{\rm c}} = 1\}$, respectively.

For arbitrary subsets $X, Y \subseteq \Lambda$, we define $d_{X,Y}$ as the shortest path length on the graph that connects X and Y; that is, if $X \cap Y \neq \emptyset$, $d_{X,Y} = 0$. When X is composed of only one element (i.e., $X = \{i\}$), we denote $d_{\{i\},Y}$ by $d_{i,Y}$ for the simplicity. We also define $\mathrm{diam}(X)$ as follows:

$$\mathrm{diam}(X) := 1 + \max_{i,j \in X} (d_{i,j}). \tag{1}$$

For a subset $X \subseteq \Lambda$, we define the extended subset $X[r]$ as

$$X[r] := \{i \in \Lambda | d_{X,i} \le r\}, \tag{2}$$

where $X[0] = X$ and r is an arbitrary positive number (i.e., $r \in \mathbb{R}^+$).

Moreover, we define $(\partial X)_s$ as follows (see Fig. 1):

$$(\partial X)_s := \begin{cases} \{i \in X | d_{i,\partial X} = |s|\} & \text{for} \quad s \le 0, \\ \{i \in X^{\rm c} | d_{i,\partial X} = s\} & \text{for} \quad s > 0, \end{cases} \tag{3}$$

where $(\partial X)_0 = \partial X$ and we have

$$X = \bigcup_{s=-\infty}^{0} (\partial X)_s, \quad \Lambda = \bigcup_{s=-\infty}^{\infty} (\partial X)_s. \tag{4}$$

For a subset $X \subseteq \Lambda$, we define the extended subset $X[r]$ as

$$X[r] := \bigcup_{s=-\infty}^{r} (\partial X)_s, \tag{5}$$

where $X[0] = X$ and r is an arbitrary integer. In particular, for $r > 0$, $X[r]$ is an extended region from X:

$$X[r] = \{i \in \Lambda | d_{X,i} \le r\}. \tag{6}$$

We introduce a geometric parameter γ which is determined only by the lattice structure. We define $\gamma \ge 1$ as a lattice constant which gives

$$|X[\ell] \setminus X| \le \gamma \ell^D |\partial X| - 1, \quad |\partial(X[\ell])| \le \gamma \ell^{D-1} |X|. \tag{7}$$

for an arbitrary $X \subseteq \Lambda$ and $\ell \ge 1$. By choosing $X = \{i\}$, we have

$$|i[\ell]| \le \gamma \ell^D, \quad |\partial(i[\ell])| \le \gamma \ell^{D-1}. \tag{8}$$

Supplementary Table 1. Fundamental parameters in our statements

**Definition**	**Parameters**
Spatial dimension	D
Structural constant [see Ineq. (7)]	γ
Hopping amplitudes [see Eq. (26)]	$\bar{J}$
Maximum interaction length (see Def. 1)	k
Amplitudes of the boson-boson interactions (see Def. 2)	g, $\bar{v}$
Parameters in the low-boson-density condition (see Assumption 3)	b_ρ, κ

We often use the notation of $\Theta(x_1, x_2, \ldots, x_s)$ that means a function with respect to $\{x_1, x_2, \ldots, x_s\}$ as follows:

$$\Theta(x_1, x_2, \ldots, x_s) = \sum_{\sigma_1, \sigma_2, \ldots, \sigma_s = 0,1} c_{\sigma_1, \sigma_2, \ldots, \sigma_s} x_1^{\sigma_1} x_2^{\sigma_2} \cdots x_s^{\sigma_s} \quad (0 < c_{\sigma_1, \sigma_2, \ldots, \sigma_s} < \infty), \tag{9}$$

where the constants $\{c_{\sigma_1, \sigma_2, \ldots, \sigma_s}\}$ only depend on the fundamental parameters listed in Table 1. Also, we use the notation of $\tilde{\mathcal{O}}(x)$ in the following sense:

$$\tilde{\mathcal{O}}(x) = \mathcal{O}\left[x \cdot \mathrm{polylog}(x)\right]. \tag{10}$$

Throughout the paper, we mainly consider three types of norms: i) the operator norm $\|\cdot\|$, ii) the trace norm $\|\cdot\|_1$ and iii) the Frobenius norm $\|\cdot\|_F$. For an arbitrary operator O, the operator norm $\|O\|$ means the maximum eigenvalue of $\sqrt{O^\dagger O}$. Also, the trace norm $\|O\|_1$ is defined as $\mathrm{tr}\left(\sqrt{O^\dagger O}\right)$. Finally, the Frobenius norm $\|O\|_F$ is defined as $\sqrt{\mathrm{tr}\,(O^\dagger O)}$.

The overview of this supplementary information is summarized in Fig. 2.

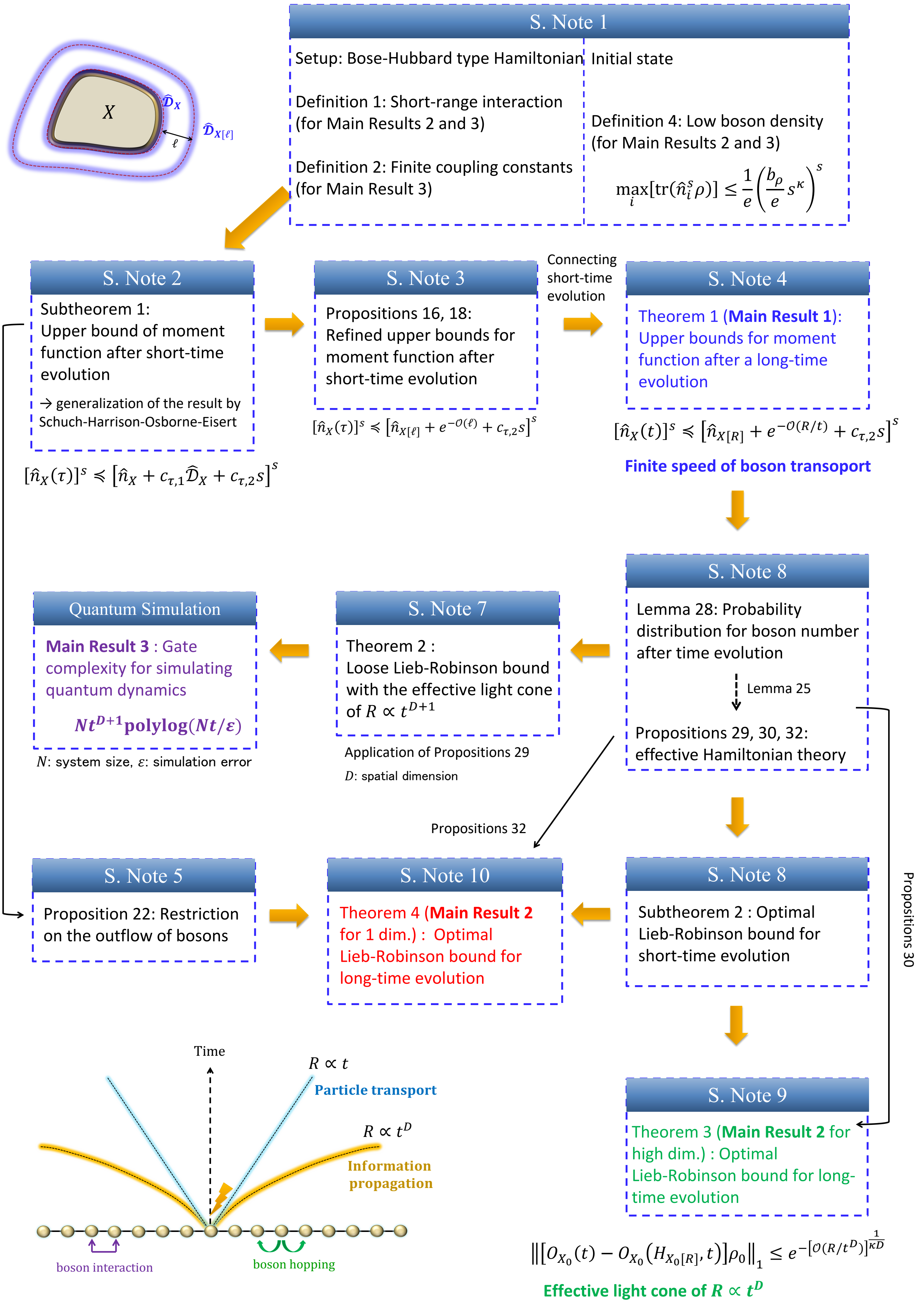

Supplementary Figure 2. Proof outline.

A. Often used inequalities

As convenient inequalities, we prove the following lemma:

Lemma 1. *Let D be an arbitrary positive integer. Then, for arbitrary positive parameters c, Φ, ξ, κ and s, we have*

$$\sum_{m=1}^{\infty} m^D e^{-cm} \le e^{-c} + e^{-c} 2^D D! \left[\max(1, 1/c)\right]^{D+1}, \tag{11}$$

$$\sum_{j\in\Lambda} e^{-cd_{i,j}} \le \lambda_c, \quad \lambda_c := 1 + c^{-D-1} e^c \gamma D!, \tag{12}$$

$$\sum_{i\in L[\ell]^c} \exp\left[-\left(\frac{qe^{d_{i,L[\ell]}/\xi}}{\Phi}\right)^{1/\kappa}\right] \le \gamma |L| 2^{2D+2} D! \max(\ell^D, 1) e^{-[q/\Phi]^{1/\kappa}/(\xi\kappa)} \quad \textit{for} \quad q \ge (\xi\kappa)^\kappa \Phi, \tag{13}$$

$$\sum_{x=1}^{\infty} (x-1)^s e^{-(x/\Phi)^{1/\kappa}} \le \Phi^{s+1} \kappa \Gamma[\kappa(s+1)], \tag{14}$$

$$x^s e^{-(x/\Phi)^{1/\kappa}} \le 2^s (2^\kappa \Phi)^{s+1} \kappa \Gamma[\kappa(s+1)] e^{-(x/\Phi)^{1/\kappa}/2} \quad \textit{for} \quad \kappa \ge 1, \quad \Phi \ge 1, \tag{15}$$

where $\Gamma(\cdot)$ is the gamma function.

1. *Proof of Lemma 1*

The proof of the inequality (11) is given as follows. First of all, for $D \ge 3$, we have

$$\sum_{m=1}^{\infty} m^D e^{-cm} = e^{-c} + \sum_{m=2}^{\infty} m^D e^{-cm} \le e^{-c} + e^{-c} \int_0^\infty (z+2)^D e^{-cz} dz$$
$$\le e^{-c} + e^{-c} 2^D D! \max(1, 1/c)^{D+1}. \tag{16}$$

For $D = 1, 2$, we use

$$\sum_{m=1}^{\infty} m^D e^{-cm} = \left(-\frac{d}{dc}\right)^D \sum_{m=1}^{\infty} e^{-cm} = \left(-\frac{d}{dc}\right)^D \frac{1}{e^c - 1}. \tag{17}$$

We have

$$\left(-\frac{d}{dc}\right) \frac{1}{e^c - 1} = \frac{e^c}{(e^c-1)^2}, \quad \left(-\frac{d}{dc}\right)^2 \frac{1}{e^c - 1} = \frac{2e^{2c}}{(e^c-1)^3} - \frac{e^c}{(e^c-1)^2}, \tag{18}$$

each of which has $e^{-c} + e^{-c} 2^D D! \max(1, 1/c)^{D+1}$ as the upper bound.

Also, we can prove the inequality (12) for an arbitrary site $i \in \Lambda$:

$$\sum_{j\in\Lambda} e^{-cd_{i,j}} = 1 + \sum_{x=1}^{\infty} \sum_{j:d_{i,j}=x} e^{-cx} \le 1 + \gamma \int_0^\infty x^D e^{c(1-x)} dx = 1 + c^{-D-1} e^c \gamma D! = \lambda_c, \tag{19}$$

where we use $\{j \in \Lambda : d_{i,j} = x\} = \partial i[x]$ and $|\partial i[x]| \le \gamma x^D$ from the inequality (8).

We here let $q_i = qe^{d_{i,L[\ell]}/\xi}$. We then upper-bound the LHS of (13) as

$$\sum_{i\in L[\ell]^c} e^{-(q_i/\Phi)^{1/\kappa}} \le \sum_{r=1}^{\infty} \sum_{i\in\partial(L[\ell+r])} e^{-\left[qe^{r/\xi}/\Phi\right]^{1/\kappa}}$$
$$\le \sum_{r=1}^{\infty} |L[\ell+r]| \, e^{-e^{r/(\xi\kappa)}[q/\Phi]^{1/\kappa}}$$
$$\le \gamma |L| \sum_{r=1}^{\infty} (\ell+r)^D e^{-r/(\xi\kappa)\cdot[q/\Phi]^{1/\kappa}}$$
$$\le \gamma 2^D |L| \sum_{r=1}^{\infty} \left(\ell^D + r^D\right) e^{-r/(\xi\kappa)\cdot[q/\Phi]^{1/\kappa}}, \tag{20}$$

where we use $e^{r/(\xi\kappa)} \geq r/(\xi\kappa)$ for $r > 0$ and $(x+y)^D \leq 2^D(x^D + y^D)$ for $x > 0$ and $y > 0$. By using the inequality (11), we can obtain

$$\sum_{r=1}^{\infty} \left(\ell^D + r^D\right) e^{-r/(\xi\kappa)\cdot[q/\Phi]^{1/\kappa}} \leq e^{-[q/\Phi]^{1/\kappa}/(\xi\kappa)} \left(2\ell^D + 1 + 2^D D!\right)$$
$$\leq 2^{D+2} D! \max(\ell^D, 1) e^{-[q/\Phi]^{1/\kappa}/(\xi\kappa)}, \tag{21}$$

where we use the condition $q \geq (\xi\kappa)^\kappa \Phi$, which implies $[q/\Phi]^{1/\kappa}/(\xi\kappa) \geq 1$, in the first inequality. By combining the inequalities (20) and (21), we prove the main inequality (13).

The inequality (14) is immediately derived as follows:

$$\sum_{x=1}^{\infty} (x-1)^s e^{-(x/\Phi)^{1/\kappa}} \leq \int_0^\infty x^s e^{-(x/\Phi)^{1/\kappa}} dx = \Phi^{s+1}\kappa\Gamma[\kappa(s+1)], \tag{22}$$

where $\Gamma(\cdot)$ is the gamma function. Using the above inequality, we can also prove the inequality (15). In the case of $x \geq 2$, we have $x^s \leq 2^s(x-1)^s$, and hence,

$$x^s e^{-(x/\Phi)^{1/\kappa}} \leq 2^s (x-1)^s e^{-(x/\Phi)^{1/\kappa}} \leq 2^s e^{-(x/\Phi)^{1/\kappa}/2} \sum_{x=1}^{\infty} (x-1)^s e^{-(x/\Phi)^{1/\kappa}/2}$$
$$\leq 2^s (2^\kappa \Phi)^{s+1} \kappa \Gamma[\kappa(s+1)] e^{-(x/\Phi)^{1/\kappa}/2}. \tag{23}$$

From the conditions $\Phi \geq 1$ and $\kappa \geq 1$, the above upper bound trivially holds for $x \leq 2$. This completes the proof of Lemma 1. □

B. Boson operators

We define b_i and $b_i^\dagger$ as the annihilation and the creation operators of boson, respectively. We also define $\hat{n}_i$ as the number operator of bosons on a site $i \in \Lambda$, namely $\hat{n}_i := b_i^\dagger b_i$. We denote the boson number on a subset X by $\hat{n}_X$, namely

$$\hat{n}_X = \sum_{i \in X} \hat{n}_i. \tag{24}$$

For an arbitrary subset $X \subseteq \Lambda$, we define $\Pi_{X,N}$ as the projection onto the eigenspace of $\hat{n}_X$ with the eigenvalue N:

$$\hat{n}_X \Pi_{X,N} = N \Pi_{X,N}. \tag{25}$$

In the case where X includes only one site (i.e., $X = \{i\}$), we denote $\Pi_{\{i\},N}$ by $\Pi_{i,N}$ for simplicity. We also define $\Pi_{X,\geq N}$ as $\sum_{N'=N}^{\infty} \Pi_{X,N'}$.

C. Bose-Hubbard type Hamiltonian

We consider a Hamiltonian in the form of

$$H = H_0 + V = \sum_{Z \subseteq \Lambda} h_Z,$$
$$H_0 := \sum_{\langle i,j \rangle} J_{i,j}(b_i b_j^\dagger + \text{h.c.}), \quad |J_{i,j}| \leq \bar{J}$$
$$V := f(\{\hat{n}_i\}_{i \in \Lambda}) = \sum_{Z \subseteq \Lambda} v_Z \tag{26}$$

where $\sum_{\langle i,j \rangle}$ means the summation for all the pairs of the adjacent sites $\{i,j\}$ on the lattice, the interaction terms h_Z or v_Z are supported on the subset $Z \subseteq \Lambda$, and $f(\{\hat{n}_i\}_{i \in \Lambda})$ is an arbitrary function for the boson number operators $\{\hat{n}_i\}_{i \in \Lambda}$. For example, $f(\{\hat{n}_i\}_{i \in \Lambda})$ includes interactions like $\hat{n}_i^4$, $\hat{n}_i^2 e^{\hat{n}_j}$, $e^{\hat{n}_i^2 \hat{n}_j^3}$ and so on. Throughout the proof, we consider the time-independent Hamiltonian for simplicity, but all the following results can be generalized to time-dependent Hamiltonians.

For an arbitrary subset $X \subseteq \Lambda$, we define the subset Hamiltonians $H_{0,X}$, V_X and H_X as follows:

$$H_X = H_{0,X} + V_X,$$
$$H_{0,X} := \sum_{i,j \in X} J_{i,j}(b_i b_j^\dagger + \text{h.c.}), \quad V_X := \sum_{Z \subseteq X} v_Z. \tag{27}$$

Supplementary Table 2. Assumptions for the main results

**Restrictions on $f(\{\hat{n}_i\}_{i\in\Lambda})$**	**Obtained theorems**
(i) No assumptions	Result 1 in the main manuscript (Theorem 1)
(ii) Short-range interactions (Def. 1) & Low boson density (Def. 3)	Result 2 in the main manuscript (Theorems 2, 3 and 4)
(iii) Short-range interactions (Def. 1) & Finite coupling constants (Def. 2) & Low boson density (Def. 3)	Result 3 in the main manuscript

Note that they are supported on the subset X. Also, we define the boundary hopping terms on the region X as follows:

$$\partial h_X := H_0 - H_{0,X} - H_{0,X^c} = \sum_{i\in\partial X} \sum_{j\in X^c:d_{i,j}=1} J_{i,j}(b_i b_j^\dagger + \text{h.c.}). \tag{28}$$

1. Assumptions on the Hamiltonian

We can derive several of our main results for the Hamiltonian without any assumptions on $f(\{\hat{n}_i\}_{i\in\Lambda})$. In Table 2, we summarize what types of Hamiltonians are assumed in deriving the main theorems. For example, in the derivation of Result 1 (or Theorem 1), $f(\{\hat{n}_i\}_{i\in\Lambda})$ can include even the macroscopic interactions like $\hat{n}_1\hat{n}_2\hat{n}_3\cdots\hat{n}_{|\Lambda|}$. We thus apply our results to arbitrary long-range interacting systems, e.g., the coulomb interaction, the dipole-dipole interaction, and so on.

However, in considering the Lieb-Robinson bound as shown in Theorems 2, 3 and 4, we need an additional assumption on the form of $f(\{\hat{n}_i\}_{i\in\Lambda})$, i.e., short-rangeness of the interactions:

Definition 1 (Short-range interactions)**.** *If all the boson-boson interactions in V are finite range with interaction length as most k, i.e.,*

$$V := f(\{\hat{n}_i\}_{i\in\Lambda}) = \sum_{Z\subset\Lambda:\text{diam}(Z)\le k} \|v_Z\|, \tag{29}$$

we call that the Hamiltonian is short-range. Here, v_Z is still an arbitrary function of $\{\hat{n}_i\}_{i\in Z}$; that is, it may be infinitely large.

The simplest example of the short-range Hamiltonian is the Bose-Hubbard model:

$$H = \sum_{\langle i,j\rangle} J(b_i b_j^\dagger + \text{h.c.}) + \frac{U}{2}\sum_{i\in\Lambda}\hat{n}_i(\hat{n}_i - 1) - \mu\sum_{i\in\Lambda}\hat{n}_i, \tag{30}$$

where U and μ are arbitrary constants.

Furthermore, in considering quantum simulation of the time evolution (Result 3 in the main manuscript), we have to assume the additional condition as follows:

Definition 2 (Finite coupling constants)**.** *The function $f(\{\hat{n}_i\}_{i\in\Lambda})$ is given by a polynomial with limited degrees and coefficients. That is, the boson-boson couplings satisfy the following inequality:*

$$\max_{i\in\Lambda}\sum_{Z:Z\ni i} v_Z \preceq g\left(\hat{n}_{i[k]}\right)^{\bar{v}}, \tag{31}$$

where g and $\bar{v}$ are $\mathcal{O}(1)$ constants.

In the case of the Bose-Hubbard Hamiltonian (30), we have $k=0$, $\bar{v}=2$, and $g=|U|/2+|\mu|$. Hence, the finiteness of g implies $U=\mathcal{O}(1)$ and $\mu=\mathcal{O}(1)$.

D. Time evolution

For an arbitrary operator O, we denote the time evolution by an operator A as $O(A,t)$, namely

$$O(A,t) := e^{iAt}Oe^{-iAt}. \tag{32}$$

In particular, when $A=H$, we often denote $O(H,t)$ by $O(t)$ for simplicity.

When the operator A is time-dependent, we utilize the following notations. As in Ref. [1], for an arbitrary time-dependent operator A_t, we define as

$$U_{A_t,\tau_1\to\tau_2} := \mathcal{T}e^{-i\int_{\tau_1}^{\tau_2} A_t dt},$$
$$O(A_t,\tau_1\to\tau_2) := U^\dagger_{A_t,\tau_1\to\tau_2} O U_{A_t,\tau_1\to\tau_2}, \tag{33}$$

where $\mathcal{T}$ is the time-ordering operator.

E. Definitions on the boson density

We here define the condition of the low boson density as follows:

Definition 3 (Low boson density)**.** *For a quantum state ρ, we say that the state satisfies the low-boson-density condition if there exists $\mathcal{O}(1)$ constants b_ρ and κ such that*

$$\max_{i\in\Lambda} \mathrm{tr}\,(\hat{n}_i^s\rho) \le \frac{1}{e}\left(\frac{b_\rho}{e}s^\kappa\right)^s. \tag{34}$$

Remark. This assumption is adopted in deriving Results 2 and 3 in the main manuscript, while Result 1 does not depend on the initial state and we do not need the assumption.

From the assumption, we can also obtain an upper bound for $\hat{n}_X$

$$\mathrm{tr}\,(\hat{n}_X^s\rho) \le \left\{\sum_{i\in X}[\mathrm{tr}\,(\hat{n}_i^s\rho)]^{1/s}\right\}^s \le \left(\frac{1}{e^{1/s}}\sum_{i\in X}\frac{b_\rho}{e}s^\kappa\right)^s = \frac{1}{e}\left(\frac{b_\rho}{e}s^\kappa|X|\right)^s. \tag{35}$$

Therefore, for $X = i[\ell]$, we have

$$\mathrm{tr}\left(\hat{n}_{i[\ell]}^s\rho\right) \le \frac{1}{e}\left(\frac{\gamma b_\rho}{e}s^\kappa\ell^D\right)^s. \tag{36}$$

Also, we often utilize the following set of boson numbers:

Definition 4. *Let $\mathbf{N}$ be a set of boson numbers on Λ, i.e., $\mathbf{N} = \{N_i\}_{i\in\Lambda}$. Then, we define the set $\mathcal{S}(L,\xi,N)$ as follows:*

$$\mathcal{S}(L,\xi,N) := \left\{\mathbf{N}|N_i \le Ne^{\xi d_{i,L}} \quad \text{for} \quad i\in\Lambda\right\}. \tag{37}$$

If $\mathbf{N}\in\mathcal{S}(L,\xi,N)$, the number of bosons cannot be too large around the region L. Also, we define the projection $\Pi_{\mathcal{S}(L,\xi,N)}$ as

$$\Pi_{\mathcal{S}(L,\xi,N)} := \sum_{\mathbf{N}\in\mathcal{S}(L,\xi,N)} P_\mathbf{N}, \tag{38}$$

where $P_\mathbf{N} = |\mathbf{N}\rangle\langle\mathbf{N}|$ is the projection onto the Mott state $|\mathbf{N}\rangle$ such that $\hat{n}_i|\mathbf{N}\rangle = N_i|\mathbf{N}\rangle$ ($i\in\Lambda$).

Remark. If a quantum state ρ satisfies

$$\Pi_{\mathcal{S}(L,\xi,N)}\rho = \rho, \tag{39}$$

we can expand the quantum state ρ by using the Mott state $|\mathbf{N}\rangle$ with $\mathbf{N}\in\mathcal{S}(L,\xi,N)$:

$$\rho = \sum_{\mathbf{N}\in\mathcal{S}(L,\xi,N)} P_\mathbf{N}\,\rho. \tag{40}$$

1. Basic lemmas on the boson density

From the condition, we can ensure that the probability for many bosons to be concentrated on one site is (sub)exponentially small. In the following, for a fixed $p\in\mathbb{N}$ and $\forall\ell\in\mathbb{N}$, we consider the quantity of

$$\mathrm{tr}\left(\rho\hat{n}_{i[\ell]}^p\Pi_{i[\ell],\ge x}\right), \tag{41}$$

which immediately yields the upper bounds on the probability distribution by choosing $p=0$

$$\mathrm{tr}\,(\rho\Pi_{i,\ge x}) \le \mathrm{tr}\left(\rho\Pi_{i[\ell],\ge x}\right). \tag{42}$$

In the following general lemma, we prove an upper bound on the probability distribution for boson numbers.

Lemma 2. *Let ρ be an arbitrary quantum state such that*

$$\mathrm{tr}(\rho \hat{n}_{i[\ell]}^{s}) \le \frac{1}{e}\left[\frac{b_\rho}{e}\left(q_0 + s^\kappa \ell^D\right)\right]^s \tag{43}$$

for a site $i \in \Lambda$ and $\ell \ge 1$, where b_ρ is an appropriate constant. Then, the assumption (43) implies the upper bound of the boson number distribution as

$$\mathrm{tr}\left(\rho \hat{n}_{i[\ell]}^{p} \Pi_{i[\ell],\ge x}\right) \le x^p e^{-\left[(x-b_\rho q_0)/(b_\rho \ell^D)\right]^{1/\kappa}} \quad \text{for} \quad x \ge \max(e, b_\rho q_0). \tag{44}$$

That is, for $p = 0$, the distribution decays (sub)exponentially for $x \gtrsim b_\rho q_0 + b_\rho \ell^D$.

Remark. If the assumption (43) holds up to $s = s_0$ with s_0 a fixed constant, the main inequality reduces to the form of

$$\mathrm{tr}\left(\rho \hat{n}_{i[\ell]}^{p} \Pi_{i[\ell],\ge x}\right) \le \max\left(x^p e^{-s_0-1}, x^p e^{-\left[(x-b_\rho q_0)/(b_\rho \bar{\ell}^D)\right]^{1/\kappa}}\right). \tag{45}$$

The inequality is immediately proved from the first inequality in (48).

2. Proof of Lemma 2

The proof immediately follows from the Markov inequality:

$$\begin{aligned}\mathrm{tr}\left(\rho \hat{n}_{i[\ell]}^{p} \Pi_{i[\ell],\ge x}\right) = \mathrm{tr}\left(\rho \hat{n}_{i[\ell]}^{p} \hat{n}_{i[\ell]}^{s-p} \cdot \hat{n}_{i[\ell]}^{-s+p} \Pi_{i[\ell],\ge x}\right) &\le \left\|\hat{n}_{i[\ell]}^{-s+p} \Pi_{i[\ell],\ge x}\right\| \mathrm{tr}\left(\rho \hat{n}_{i[\ell]}^{s}\right) \\ &\le \frac{1}{e}\left[\frac{b_\rho}{e}\left(q_0 + s^\kappa \ell^D\right)\right]^p \left(\frac{b_\rho}{e} \cdot \frac{q_0 + s^\kappa \ell^D}{x}\right)^{s-p},\end{aligned} \tag{46}$$

where we assume that $s \ge p$. Then, by choosing s such that

$$\frac{b_\rho}{e}\left(q_0 + s^\kappa \ell^D\right) \le \frac{x}{e} \longrightarrow s^\kappa \le \frac{x - b_\rho q_0}{b_\rho \ell^D} \longrightarrow s = \left\lfloor \left(\frac{x - b_\rho q_0}{b_\rho \ell^D}\right)^{1/\kappa} \right\rfloor, \tag{47}$$

we reduce the inequality (46) to

$$\begin{aligned}\mathrm{tr}\left(\rho \hat{n}_{i[\ell]}^{p} \Pi_{i[\ell],\ge x}\right) \le \frac{1}{e}\left(\frac{x}{e}\right)^p e^{-s+p} &\le \frac{1}{e}\left(\frac{x}{e}\right)^p \exp\left[p + 1 - \left(\frac{x - b_\rho q_0}{b_\rho \ell^D}\right)^{1/\kappa}\right] \\ &= x^p \exp\left[-\left(\frac{x - b_\rho q_0}{b_\rho \ell^D}\right)^{1/\kappa}\right].\end{aligned} \tag{48}$$

In the case of $s < p$ (or $s \le p - 1$) and $x \ge e$, the above inequality trivially holds since the first RHS of the above inequality (i.e., $\frac{1}{e}\left(\frac{x}{e}\right)^p e^{-s+p}$) is larger than 1. We thus prove the inequality (44). □

———————————————————— [**End of Proof of Lemma 2**]

We consider how an operator influences the moment function as another essential lemma. For example, we are interested in the moment difference between the original state ρ and $O_L^\dagger \rho O_L$, where O_L is supported on $L \subset \Lambda$. We here prove the following lemma:

Lemma 3. *Let O_q be an arbitrary operator such that*

$$\Pi_{L,>m+q} O_q \Pi_{L,m}, \quad \Pi_{L,<m-q} O_q \Pi_{L,m} = 0 \quad \text{for} \quad \forall m > 0. \tag{49}$$

Note that O_q is not necessarily supported on the subset L. Then, we obtain

$$O_q^\dagger \left(\sum_{i \in \Lambda} \nu_i \hat{n}_i\right)^m O_q \preceq 4^m \|O_q\|^2 \left(4\bar{\nu} q^3 + \bar{\nu} \hat{n}_L + \sum_{i \notin L} \nu_i \hat{n}_i\right)^m, \tag{50}$$

where we define $\bar{\nu} := \max_{i \in L}(\nu_i)$ with $\nu_i > 0$. Here, for arbitrary operators O_1 and O_2, the notation $O_1 \preceq O_2$ means that $O_2 - O_1$ is a semi-positive definite operator.

3. Proof of Lemma 3

We here define $\hat{Q}$ as

$$\hat{Q} := \left(\bar{\nu}\hat{n}_L + \sum_{i\notin L}\nu_i\hat{n}_i\right)^{1/2}. \tag{51}$$

Then, from the definition of $\bar{\nu}$, we obtain

$$O_q^\dagger\left(\sum_{i\in\Lambda}\nu_i\hat{n}_i\right)^m O_q \preceq O_q^\dagger\hat{Q}^{2m}O_q. \tag{52}$$

First of all, by using the condition (49), we aim to prove

$$\left\|[\hat{Q}, O_q]\right\| \le 2q\sqrt{\bar{\nu}q}\,\|O_q\|. \tag{53}$$

For proof, we first decompose

$$O_q = \sum_{q_1=-q}^{q} O_q^{(q_1)}, \tag{54}$$

where $O_q^{(q_1)}$ satisfies $\Pi_{L,m'}O_q^{(q_1)}\Pi_{L,m} \neq 0$ only for $m' = m + q_1$. For arbitrary $q_1 \in [-q, q]$, we obtain

$$\left[\hat{Q}, O_q^{(q_1)}\right] = \left[\left(\bar{\nu}\hat{n}_L + \sum_{i\notin L}\nu_i\hat{n}_i\right)^{1/2} - \left(\bar{\nu}(\hat{n}_L - q_1) + \sum_{i\notin L}\nu_i\hat{n}_i\right)^{1/2}\right] O_q^{(q_1)}, \tag{55}$$

which yields

$$\left\|\left[\hat{Q}, O_q^{(q_1)}\right]\right\| \le \sqrt{\bar{\nu}|q_1|}\left\|O_q^{(q_1)}\right\|. \tag{56}$$

where we use $|\sqrt{x} - \sqrt{x-y}| \le \sqrt{|y|}$. We then aim to prove

$$\left\|O_q^{(q_1)}\right\| \le \|O_q\|. \tag{57}$$

From the definition, we have $[O_q^{(q_1)\dagger}O_q^{(q_1)}, \hat{n}_L] = 0$, and hence the eigenstate of $O_q^{(q_1)\dagger}O_q^{(q_1)}$ is expressed as $|\psi_m\rangle$ such that $\hat{n}_L|\psi_m\rangle = m|\psi_m\rangle$, where $|\psi_m\rangle$ is an appropriate superposition of the eigenstates of $\hat{n}_L$ with the eigenvalue m. On the other hand, from the properties of $O_q^{(q_1)}$, we obtain

$$O_q^{(q_1)}|\psi_m\rangle = \Pi_{L,m+q_1}O_q^{(q_1)}|\psi_m\rangle, \quad O_q|\psi_m\rangle = \sum_{q_1=-q}^{q}\Pi_{L,m+q_1}O_q^{(q_1)}|\psi_m\rangle, \tag{58}$$

which immediately yields

$$\left\|O_q^{(q_1)}\right\| = \max_{|\psi_m\rangle}\left\|O_q^{(q_1)}|\psi_m\rangle\right\| \le \max_{|\psi_m\rangle}\|O_q|\psi_m\rangle\| \le \|O_q\|. \tag{59}$$

By combining the inequalities (56) and (59), we have

$$\left\|[\hat{Q}, O_q]\right\| \le \sum_{q_1=-q}^{q}\left\|\left[\hat{Q}, O_q^{(q_1)}\right]\right\| \le \|O_q\|\sum_{q_1=-q}^{q}\sqrt{\bar{\nu}|q_1|} \le 2q\sqrt{\bar{\nu}q}\,\|O_q\|, \tag{60}$$

which gives the desired inequality (53).

In the following, by induction on m, we aim to prove

$$\left\|\hat{Q}^m O_q|\psi\rangle\right\| \le 2^{m/2}\|O_q\|\cdot\left\|\left(\hat{Q} + 2q\sqrt{\bar{\nu}q}\right)^m|\psi\rangle\right\|. \tag{61}$$

for arbitrary quantum state $|\psi\rangle$ and operator O_q satisfying Eq. (49). For $m = 1$, we can immediately prove the inequality (61) as follows:

$$\begin{aligned}\left\|\hat{Q}O_q|\psi\rangle\right\| &\le \left\|[\hat{Q}, O_q]|\psi\rangle\right\| + \left\|O_q\hat{Q}|\psi\rangle\right\| \le \|O_q\|\left(\left\|2q\sqrt{\bar{\nu}q}|\psi\rangle\right\| + \left\|\hat{Q}|\psi\rangle\right\|\right) \\ &\le \sqrt{2}\,\|O_q\|\left(\left\|\left(\hat{Q} + 2q\sqrt{\bar{\nu}q}\right)|\psi\rangle\right\|\right).\end{aligned} \tag{62}$$

In deriving the last inequality above, for arbitrary positive commutative operators Φ_1 and Φ_2, we have used

$$\|(\Phi_1+\Phi_2)|\psi\rangle\|^2 = \langle\psi|(\Phi_1^2+2\Phi_1\Phi_2+\Phi_2^2)|\psi\rangle \geq \langle\psi|\Phi_1^2|\psi\rangle + \langle\psi|\Phi_2^2|\psi\rangle \geq \left(\frac{\sqrt{\langle\psi|\Phi_1^2|\psi\rangle}+\sqrt{\langle\psi|\Phi_2^2|\psi\rangle}}{\sqrt{2}}\right)^2, \tag{63}$$

which yields

$$\|\Phi_1|\psi\rangle\| + \|\Phi_2|\psi\rangle\| \leq \sqrt{2}\,\|(\Phi_1+\Phi_2)|\psi\rangle\|. \tag{64}$$

We then prove the case of $m+1$ by assuming Eq. (61) for a fixed m. We then obtain

$$\left\|\hat{Q}^{m+1}O_q|\psi\rangle\right\| = \left\|\hat{Q}^m\left([\hat{Q},O_q]+O_q\hat{Q}\right)|\psi\rangle\right\| \leq \left\|\hat{Q}^m[\hat{Q},O_q]|\psi\rangle\right\| + \left\|\hat{Q}^mO_q\hat{Q}|\psi\rangle\right\|. \tag{65}$$

First, for $\left\|\hat{Q}^m[\hat{Q},O_q]|\psi\rangle\right\|$ in the above inequality, Eq. (61) gives

$$\left\|\hat{Q}^m[\hat{Q},O_q]|\psi\rangle\right\| \leq 2^{m/2}\left\|[\hat{Q},O_q]\right\|\cdot\left\|\left(\hat{Q}+2q\sqrt{\bar{\nu}q}\right)^m|\psi\rangle\right\| \leq 2^{m/2}\|O_q\|\cdot\left\|2q\sqrt{\bar{\nu}q}\left(\hat{Q}+2q\sqrt{\bar{\nu}q}\right)^m|\psi\rangle\right\|, \tag{66}$$

where we use the inequality (61) by letting $O_q \to [\hat{Q},O_q]$ in the first inequality and apply (53) to the second inequality. Note that we can apply the inequality (61) to $[\hat{Q},O_q]$ since it still satisfies the condition (49). Second, for $\left\|\hat{Q}^mO_q\hat{Q}|\psi\rangle\right\|$ in the inequality (65), we have from Eq. (61)

$$\left\|\hat{Q}^mO_q\hat{Q}|\psi\rangle\right\| \leq 2^{m/2}\|O_q\|\cdot\left\|\left(\hat{Q}+2q\sqrt{\bar{\nu}q}\right)^m\hat{Q}|\psi\rangle\right\|. \tag{67}$$

By applying the inequalities (66) and (67) to (65), we obtain

$$\begin{aligned}\left\|\hat{Q}^{m+1}O_q|\psi\rangle\right\| &\leq 2^{m/2}\|O_q\|\left(\left\|2q\sqrt{\bar{\nu}q}\left(\hat{Q}+2q\sqrt{\bar{\nu}q}\right)^m|\psi\rangle\right\| + \left\|\hat{Q}\left(\hat{Q}+2q\sqrt{\bar{\nu}q}\right)^m|\psi\rangle\right\|\right)\\ &\leq 2^{(m+1)/2}\|O_q\|\cdot\left\|\left(\hat{Q}+2q\sqrt{\bar{\nu}q}\right)^{m+1}|\psi\rangle\right\|,\end{aligned} \tag{68}$$

where we use (64) in the second inequality. We thus prove the inequality (61) for $m+1$.

From the inequality (61), we obtain

$$\langle\psi|O_q^\dagger\hat{Q}^{2m}O_q|\psi\rangle = \left\|\hat{Q}^mO_q|\psi\rangle\right\|^2 \leq 2^m\|O_q\|^2\cdot\langle\psi|\left(\hat{Q}+2q\sqrt{\bar{\nu}q}\right)^{2m}|\psi\rangle \tag{69}$$

By using the definition (51) and the inequality of

$$\left(\hat{Q}+2q\sqrt{\bar{\nu}q}\right)^2 \preceq 2\left(\hat{Q}^2+4\bar{\nu}q^3\right), \tag{70}$$

we reduce the inequality (69) to the form of (50). This completes the proof. □

Supplementary Note 2. UPPER BOUND ON MOMENT FUNCTION AFTER SHORT-TIME EVOLUTION

A. Statement

We first consider the sth moment function in the form of

$$M_{X_1,X_2,\ldots,X_s}(\tau) := \mathrm{tr}\left[\hat{n}_{X_1}\hat{n}_{X_2}\cdots\hat{n}_{X_s}\rho(\tau)\right]. \tag{71}$$

As long as $X_j \supseteq X$ $(j=1,2,\ldots,s)$, because of

$$\hat{n}_X^s \preceq \hat{n}_{X_1}\hat{n}_{X_2}\cdots\hat{n}_{X_s}, \tag{72}$$

we have

$$M_X^{(s)}(\tau) \leq M_{X_1,X_2,\ldots,X_s}(\tau). \tag{73}$$

In the following, we aim to upper-bound a short-time evolution of the generalized moment function (71). The primary reason is to derive Proposition 18 by resolving the obstacle addressed in Sec. Supplementary Note 3 E 1 below.

In our analyses, the following subtheorem plays a central role:

Subtheorem 1. *Let ρ be an arbitrary quantum state, and $\{X_j\}_{j=1}^s$ be arbitrary subsets. Then, as long as $\tau \le 1/(4\gamma \bar{J})$ we obtain the following upper bound for $M_{X_1,X_2,\ldots,X_s}(\tau)$:*

$$M_{X_1,X_2,\ldots,X_s}(\tau) \le \mathrm{tr}\left[\prod_{j=1}^{s}\left(\hat{n}_{X_j} + c_{\tau,1}\hat{\mathcal{D}}_{X_j} + c_{\tau,2}s\right)\rho\right] \tag{74}$$

with

$$\hat{\mathcal{D}}_X := \sum_{i\in\partial X}\sum_{j\in\Lambda} e^{-d_{i,j}}\hat{n}_j \tag{75}$$

where $c_{\tau,1}$ and $c_{\tau,2}$ are defined as

$$c_{\tau,1} = 40e^{4\gamma\bar{J}\tau}, \quad c_{\tau,2} = e^{4\gamma\bar{J}\tau+1}\left(1 + 8\gamma\bar{J}\tau + \tau\right). \tag{76}$$

Notice that they are $\mathcal{O}(1)$ constants as long as $\tau = \mathcal{O}(1)$.

Remark. This subtheorem is a generalization of the previous work by Schuch, Harrison, Osborne, and Eisert [2] that essentially treated the case of $s = 1$. Because the inequality (74) holds for arbitrary quantum states unconditionally, we can also obtain the operator inequality as follows:

$$\prod_{j=1}^{s}\hat{n}_{X_j}(\tau) \preceq \prod_{j=1}^{s}\left(\hat{n}_{X_j} + c_{\tau,1}\hat{\mathcal{D}}_{X_j} + c_{\tau,2}s\right). \tag{77}$$

In particular, for $s = 1$, we have

$$\hat{n}_X(\tau) \preceq \hat{n}_X + c_{\tau,1}\hat{\mathcal{D}}_X + c_{\tau,2}. \tag{78}$$

1. Effective Hamiltonian

We here consider a time evolution by the Hamiltonian H, but the statement can be extended to a more general class of Hamiltonian as follow:

$$T_{\hat{n}}^{\dagger}HT_{\hat{n}}, \quad [T_{\hat{n}}, \hat{n}_i] = 0 \quad \text{for} \quad \forall i \in \Lambda, \quad \|T_{\hat{n}}\| \le 1. \tag{79}$$

By following the proof of Subtheorem 1, we can immediately prove that the same statement as Subtheorem 1 holds for the Hamiltonian $T_{\hat{n}}^{\dagger}HT_{\hat{n}}$:

Corollary 4. *For the Hamiltonian $T_{\hat{n}}^{\dagger}HT_{\hat{n}}$, the time evolution $M_{X_1,X_2,\ldots,X_s}(T_{\hat{n}}^{\dagger}HT_{\hat{n}}, \tau)$ satisfies the same inequality as (74). The proof is the same as that for Subtheorem 1.*

2. Imaginary time evolution

We here consider a real-time evolution as $e^{-iH\tau}$, but the same proof below can be applied to the imaginary time evolution. We then immediately obtain the following corollary:

Corollary 5. *For the imaginary time evolution*

$$\rho(i\tau) = e^{\tau H}\rho e^{-\tau H},$$

and

$$O(i\tau) = e^{-\tau H}Oe^{\tau H},$$

we obtain

$$\prod_{j=1}^{s}\hat{n}_{X_j}(i\tau) \preceq \prod_{j=1}^{s}\left(\hat{n}_{X_j} + c_{\tau,1}\hat{\mathcal{D}}_{X_j} + c_{\tau,2}s\right). \tag{80}$$

Using the upper bound, we can also obtain the same statement as Theorem 1. We hence ensure that during the imaginary time evolution the boson number can be truncated at each site up to $\mathcal{O}(\tau^D)$, which is helpful in quantum simulation of the imaginary time evolution[*1].

[*1] Precisely speaking, we have to estimate the expectation as

$$\langle\psi(i\tau)|\hat{n}_i^s|\psi(i\tau)\rangle := \langle\psi|e^{-\tau H}\hat{n}_i^s e^{-\tau H}|\psi\rangle$$

with $|\psi\rangle$ an appropriate initial state, where we set the energy origin such that $\left\|e^{-\tau H}|\psi\rangle\right\| = 1$. To connect the above expectation to $[\hat{n}_i(i\tau)]^s$, we rewrite as

$$\langle\psi(i\tau)|\hat{n}_i^s|\psi(i\tau)\rangle = \langle\psi(2i\tau)|[\hat{n}_i(i\tau)]^s|\psi\rangle \le \||\psi(2i\tau)\rangle\| \sqrt{\langle\psi|[\hat{n}_i(i\tau)]^{2s}|\psi\rangle}.$$

Thus, if $\||\psi(2i\tau)\rangle\| \,/\, \||\psi(i\tau)\rangle\| \lesssim 1$, we can obtain a meaningful upper bound by applying the inequality (80).

B. Proof of Subtheorem 1

In the proof, we consider the case of $X_1 = X_2 = \cdots = X_s$ for simplicity of the notations. The choice of the subsets does not influence the proof, and the generalization to generic $\{X_j\}_{j=1}^s$ is straightforward.

As a difficulty, it is not sufficient to analyze only the 1st moment in deriving the main inequality (74). Even if we obtain the operator inequality (78) for $s = 1$, i.e.,

$$\hat{n}_{X_1}(\tau) \preceq \hat{n}_{X_1} + c_{\tau,1}\hat{\mathcal{D}}_{X_1} + c_{\tau,2}, \quad \text{and} \quad \hat{n}_{X_2}(\tau) \preceq \hat{n}_{X_2} + c_{\tau,1}\hat{\mathcal{D}}_{X_2} + c_{\tau,2}, \tag{81}$$

we cannot simply connect the inequality to obtain the 2nd order moment:

$$\hat{n}_{X_1}(\tau)\hat{n}_{X_2}(\tau) \preceq \left(\hat{n}_{X_1} + c_{\tau,1}\hat{\mathcal{D}}_{X_1} + c_{\tau,2}\right)\left(\hat{n}_{X_2} + c_{\tau,1}\hat{\mathcal{D}}_{X_2} + c_{\tau,2}\right), \tag{82}$$

which is NOT correct. Usually, for two operators A and B, the operator inequalities $A \preceq A'$ and $B \preceq B'$ do not lead to $AB \preceq A'B'$. Therefore, we must develop an analytical technique to treat the sth moment.

In the following, we aim to obtain a simple inequality for $M_X^{(s)}(\tau)$ by appropriately upper-bounding the derivatives concerning the time τ. Our main purpose is to prove the inequality (133) below. To make the point clear, we first consider the first differential equation for $M_X^{(s)}(\tau)$:

$$\frac{d}{d\tau}M_X^{(s)}(\tau) = -i\mathrm{tr}\left(\hat{n}_X^s[H,\rho(\tau)]\right) = i\mathrm{tr}\left([H,\hat{n}_X^s]\rho(\tau)\right). \tag{83}$$

The form of the Hamiltonian (26) gives

$$[H,\hat{n}_X^s] = [H_0,\hat{n}_X^s] = [\partial h_X,\hat{n}_X^s] = \sum_{i\in\partial X}\sum_{j\in X^{\mathrm{c}}:d_{i,j}=1} J_{i,j}[b_i b_j^\dagger + \mathrm{h.c.},\hat{n}_X^s], \tag{84}$$

where we use $[H_{0,X},\hat{n}_X^s]=0$ and $[H_{0,X^{\mathrm{c}}},\hat{n}_X^s]=0$ [see (28) for the notation of ∂h_X]. Note that $[V,\hat{n}_X^s]=0$ because of $[\hat{n}_i,\hat{n}_j]=0$ for $\forall j\in\Lambda$. By using $[b_i,\hat{n}_X]=b_i$ for $\forall i\in X$, we have

$$[b_i b_j^\dagger,\hat{n}_X^s] = \sum_{s_1=0}^{s-1}\hat{n}_X^{s_1}[b_i b_j^\dagger,\hat{n}_X]\hat{n}_X^{s-s_1-1} = \sum_{s_1=0}^{s-1}\hat{n}_X^{s_1}b_i b_j^\dagger\hat{n}_X^{s-s_1-1}, \tag{85}$$

which yields

$$\begin{aligned}\left|\frac{d}{d\tau}M_X^{(s)}(\tau)\right| &= \left|\mathrm{tr}\big([H,\hat{n}_X^s]\rho(\tau)\big)\right| \\ &\le \sum_{s_1=0}^{s-1}\left|\mathrm{tr}\left(\hat{n}_X^{s_1}\sum_{i\in\partial X}\sum_{j\in X^{\mathrm{c}}:d_{i,j}=1}\bar{J}\left(b_i b_j^\dagger - \mathrm{h.c.}\right)\hat{n}_X^{s-s_1-1}\rho(\tau)\right)\right|.\end{aligned} \tag{86}$$

We now need to obtain an upper bound for

$$\left|\mathrm{tr}\left(\hat{n}_X^{s_1}b_i b_j^\dagger\hat{n}_X^{s-s_1-1}\rho(\tau)\right)\right|. \tag{87}$$

In general, we can prove the following proposition:

Proposition 6. *For arbitrary s_1, s_1', s_2 and s_2', we obtain the upper bound as*

$$\left|\mathrm{tr}\left[f(\hat{\mathbf{n}})\hat{n}_i^{s_1}\hat{n}_j^{s_2}b_i b_j^\dagger\hat{n}_i^{s_1'}\hat{n}_j^{s_2'}\rho(\tau)\right]\right| \le \frac{1}{2}\mathrm{tr}\left[f(\hat{\mathbf{n}})\left(\hat{n}_i^{s_1+s_1'}(\hat{n}_j+1)^{s_2+s_2'} + (\hat{n}_i+1)^{s_1+s_1'}\hat{n}_j^{s_2+s_2'}\right)(\hat{n}_i+\hat{n}_j)\rho(\tau)\right], \tag{88}$$

where $f(\hat{\mathbf{n}})$ is an arbitrary non-negative function with respect to $\{\hat{n}_s\}_{s\neq i,j}$ (i.e., it satisfies $[f(\hat{\mathbf{n}}),b_i]=[f(\hat{\mathbf{n}}),b_j]=0$).

1. Proof of Proposition 6

Let $|\mathbf{N}\rangle$ ($\mathbf{N}=\{N_i\}_{i\in\Lambda}$) be an eigenstate such of the boson number operators, i.e., $\hat{n}_i|\mathbf{N}\rangle = N_i|\mathbf{N}\rangle$ for $\forall i\in\Lambda$. For the proof, by using $b_i|\mathbf{N}\rangle = \sqrt{N_i}|N_i-1,N_j,\mathbf{N}_{i,j}\rangle$ and $b_j^\dagger|\mathbf{N}\rangle = \sqrt{N_j+1}|N_i,N_j+1,\mathbf{N}_{i,j}\rangle$, we first transform

$$\begin{aligned}\mathrm{tr}\left[f(\hat{\mathbf{n}})\hat{n}_i^{s_1}\hat{n}_j^{s_2}b_i b_j^\dagger\hat{n}_i^{s_1'}\hat{n}_j^{s_2'}\rho(\tau)\right] &= \mathrm{tr}\left[f(\hat{\mathbf{n}})\hat{n}_i^{s_1}\hat{n}_j^{s_2}b_i b_j^\dagger\hat{n}_i^{s_1'}\hat{n}_j^{s_2'}\sum_{\mathbf{N}}P_{\mathbf{N}}\rho(\tau)\right] \quad (P_{\mathbf{N}} := |\mathbf{N}\rangle\langle\mathbf{N}|) \\ &= \sum_{\mathbf{N}} f(\mathbf{N}_{i,j})N_i^{s_1'}N_j^{s_2'}\sqrt{N_i}\sqrt{N_j+1}(N_i-1)^{s_1}(N_j+1)^{s_2}\langle N_i,N_j,\mathbf{N}_{i,j}|\rho(\tau)|N_i-1,N_j+1,\mathbf{N}_{i,j}\rangle,\end{aligned} \tag{89}$$

where we use a notation of $\mathbf{N} = \{N_i, N_j, \mathbf{N}_{i,j}\}$, where $\mathbf{N}_{i,j}$ includes the boson number in $\Lambda \setminus \{i,j\}$. From the Cauchy-Schwartz inequality, we have

$$\begin{aligned}
&\langle N_i, N_j, \mathbf{N}_{i,j} | \rho(\tau) | N_i - 1, N_j + 1, \mathbf{N}_{i,j} \rangle \\
&\le \frac{1}{2} \left(\langle N_i, N_j, \mathbf{N}_{i,j} | \rho(\tau) | N_i, N_j, \mathbf{N}_{i,j} \rangle + \langle N_i - 1, N_j + 1, \mathbf{N}_{i,j} | \rho(\tau) | N_i - 1, N_j + 1, \mathbf{N}_{i,j} \rangle \right). \qquad (90)
\end{aligned}$$

By applying the above inequality to Eq. (89), we obtain

$$\begin{aligned}
&\left| \mathrm{tr} \left[f(\hat{\mathbf{n}}) \hat{n}_i^{s_1} \hat{n}_j^{s_2} b_i b_j^\dagger \hat{n}_i^{s_1'} \hat{n}_j^{s_2'} \rho(\tau) \right] \right| \\
&\le \frac{1}{2} \sum_{\mathbf{N}} f(\mathbf{N}_{i,j}) N_i^{s_1'} N_j^{s_2'} \sqrt{N_i} \sqrt{N_j + 1} (N_i - 1)^{s_1} (N_j + 1)^{s_2} \langle N_i, N_j, \mathbf{N}_{i,j} | \rho(\tau) | N_i, N_j, \mathbf{N}_{i,j} \rangle \\
&+ \frac{1}{2} \sum_{\mathbf{N}} f(\mathbf{N}_{i,j}) N_i^{s_1'} N_j^{s_2'} \sqrt{N_i} \sqrt{N_j + 1} (N_i - 1)^{s_1} (N_j + 1)^{s_2} \langle N_i - 1, N_j + 1, \mathbf{N}_{i,j} | \rho(\tau) | N_i - 1, N_j + 1, \mathbf{N}_{i,j} \rangle \\
&\le \frac{1}{2} \sum_{\mathbf{N}} f(\mathbf{N}_{i,j}) N_i^{s_1 + s_1'} (N_j + 1)^{s_2 + s_2'} (N_i + N_j) \langle N_i, N_j, \mathbf{N}_{i,j} | \rho(\tau) | N_i, N_j, \mathbf{N}_{i,j} \rangle \\
&+ \frac{1}{2} \sum_{\mathbf{N}} f(\mathbf{N}_{i,j}) (N_i + 1)^{s_1 + s_1'} N_j^{s_2 + s_2'} (N_i + N_j) \langle N_i, N_j, \mathbf{N}_{i,j} | \rho(\tau) | N_i, N_j, \mathbf{N}_{i,j} \rangle \\
&= \frac{1}{2} \sum_{\mathbf{N}} f(\mathbf{N}_{i,j}) \left(N_i^{s_1 + s_1'} (N_j + 1)^{s_2 + s_2'} + (N_i + 1)^{s_1 + s_1'} N_j^{s_2 + s_2'} \right) (N_i + N_j) \langle N_i, N_j, \mathbf{N}_{i,j} | \rho(\tau) | N_i, N_j, \mathbf{N}_{i,j} \rangle \qquad (91) \\
&= \frac{1}{2} \mathrm{tr} \left[f(\hat{\mathbf{n}}) \left(\hat{n}_i^{s_1 + s_1'} (\hat{n}_j + 1)^{s_2 + s_2'} + (\hat{n}_i + 1)^{s_1 + s_1'} \hat{n}_j^{s_2 + s_2'} \right) (\hat{n}_i + \hat{n}_j) \sum_{\mathbf{N}} P_{\mathbf{N}} \rho(\tau) \right] \\
&= \frac{1}{2} \mathrm{tr} \left[f(\hat{\mathbf{n}}) \left(\hat{n}_i^{s_1 + s_1'} (\hat{n}_j + 1)^{s_2 + s_2'} + (\hat{n}_i + 1)^{s_1 + s_1'} \hat{n}_j^{s_2 + s_2'} \right) (\hat{n}_i + \hat{n}_j) \rho(\tau) \right], \qquad (92)
\end{aligned}$$

where we use the facts that the operator $f(\hat{\mathbf{n}})$ commutes with b_i and b_j, $f(\mathbf{N}_{i,j}) \ge 0$, and $f(\hat{\mathbf{n}})|\mathbf{N}\rangle = f(\mathbf{N}_{i,j})|\mathbf{N}\rangle$. Note that

$$\max\left(\sqrt{N_i(N_j + 1)}, \sqrt{(N_i + 1)N_j} \right) \le N_i + N_j \quad \text{for} \quad \forall N_i, N_j \in \mathbb{N}.$$

This completes the proof. □

[End of Proof of Proposition 6]

By applying Proposition 6 to the inequality (86), we have

$$\begin{aligned}
\left| \frac{d}{d\tau} M_X^{(s)}(\tau) \right| &\le \sum_{s_1=0}^{s-1} \left| \mathrm{tr} \left(\hat{n}_X^{s_1} \sum_{i \in \partial X} \sum_{j \in X^c : d_{i,j}=1} \bar{J} \left(b_i b_j^\dagger - \mathrm{h.c.} \right) \hat{n}_X^{s - s_1 - 1} \rho(\tau) \right) \right| \\
&\le \sum_{s_1=0}^{s-1} \mathrm{tr} \left[(\hat{n}_X + 1)^{s_1} \sum_{i \in \partial X} \sum_{j \in X^c : d_{i,j}=1} 2\bar{J} (\hat{n}_i + \hat{n}_j) (\hat{n}_X + 1)^{s - s_1 - 1} \rho(\tau) \right] \\
&\le 2 \sum_{s_1=0}^{s-1} \mathrm{tr} \left[(\hat{n}_X + 1)^{s_1} \sum_{i \in \partial X} \left(\gamma \bar{J} \hat{n}_i + \sum_{j : d_{i,j}=1} \bar{J} \hat{n}_j \right) (\hat{n}_X + 1)^{s - s_1 - 1} \rho(\tau) \right], \qquad (93)
\end{aligned}$$

where we use $\sum_{j:d_{i,j}=1} 1 \le |i[1]| \le \gamma$ in the third inequality. Note that the parameter γ have been defined in Eq. (7).

In the same way, we can also upper-bound the derivative of

$$\left| \frac{d}{d\tau} \mathrm{tr} \left[(\hat{n}_X + 1)^{s_1} \sum_{i \in \partial X} \left(\gamma \bar{J} \hat{n}_i + \sum_{j : d_{i,j}=1} \bar{J} \hat{n}_j \right) (\hat{n}_X + 1)^{s - s_1 - 1} \rho(\tau) \right] \right| \qquad (94)$$

by using the same analyses and Proposition 6. In the following, we aim to upper-bound the mth-order derivative by utilizing the upper bound for $(m-1)$th-order derivative. However, the notation becomes more and more complicated as the order of derivatives increases.

To simplify the notation, we define the following super-operator $\mathcal{M}$, which acts on arbitrary $\hat{n}_i$ ($i \in \Lambda$), by

$$\mathcal{M}(\hat{n}_i) := \gamma \bar{J} \hat{n}_i + \sum_{j : d_{i,j}=1} \bar{J} \hat{n}_j \quad \text{for} \quad \forall i \in \Lambda. \qquad (95)$$

The super-operator $\mathcal{M}$ is linear in the sense that

$$\mathcal{M}\left(\sum_{i\in\Lambda}\nu_i\hat{n}_i\right) := \sum_{i\in\Lambda}\nu_i\mathcal{M}\left(\hat{n}_i\right). \tag{96}$$

Recursively, we can define $\mathcal{M}^m$ for general $m\in\mathbb{N}$ by

$$\mathcal{M}^0(\hat{n}_i) := \hat{n}_i, \tag{97}$$
$$\mathcal{M}^m(\hat{n}_i) := \mathcal{M}(\mathcal{M}^{m-1}(\hat{n}_i)), \tag{98}$$

which are also linear super-operators. Using the super-operator $\mathcal{M}$, we define the super-operator $\mathcal{D}^m$, which acts on $\hat{n}_X$, by

$$\mathcal{D}^0(\hat{n}_X) := \hat{n}_X, \quad \mathcal{D}^1(\hat{n}_X) := \sum_{i\in\partial X}\mathcal{M}(\hat{n}_i),$$
$$\mathcal{D}^{m+1}(\hat{n}_X) := \mathcal{M}(\mathcal{D}^m(\hat{n}_X)) \quad \text{for} \quad \forall m\ge 1. \tag{99}$$

From the definitions (95) and (99), by acting the super-operator $\mathcal{D}^m$, the operator $\mathcal{D}^m(\hat{n}_X)$ is still a linear combination of $\{\hat{n}_i\}_{i\in\Lambda}$. For the operator $\mathcal{D}^m(\hat{n}_X)$, we can prove the following lemma:

Lemma 7. *Let us define X_m as the set of all sites where the number operators $\{\hat{n}_i\}$ appear in $\mathcal{D}^m(\hat{n}_X)$, i.e.,*

$$\mathcal{D}^m(\hat{n}_X) = \sum_{i\in X_m}\alpha_i^{(m)}\hat{n}_i, \tag{100}$$

where $\alpha_i^{(m)}>0$. Then, we can upper-bound $\alpha_i^{(m)}$ as

$$\max_{i\in X_m}\left(\alpha_i^{(m)}\right) \le (2\gamma\bar{J})^m. \tag{101}$$

2. Proof of Lemma 7

Because of the definition (95) for $\mathcal{M}$, we obtain

$$\mathcal{D}^{m+1}(\hat{n}_X) = \sum_{i\in X_m}\alpha_i^{(m)}\mathcal{M}(\hat{n}_i) = \sum_{i\in X_m}\alpha_i^{(m)}\left(\gamma\bar{J}\hat{n}_i + \sum_{j:d_{i,j}=1}\bar{J}\hat{n}_j\right)$$
$$\preceq \sum_{i\in X_{m+1}}\left(\alpha_i^{(m)}\gamma\bar{J} + \sum_{j\in X_m:d_{i,j}=1}\alpha_j^{(m)}\bar{J}\right)\hat{n}_i,$$

which yields

$$\max_{i\in X_{m+1}}\left[\alpha_i^{(m+1)}\right] \le \left(\gamma\bar{J} + \sum_{j:d_{i,j}=1}\bar{J}\right)\max_{i\in X_m}\left[\alpha_i^{(m)}\right] \le 2\gamma\bar{J}\max_{i\in X_m}\left[\alpha_i^{(m)}\right], \tag{102}$$

where we use $\sum_{j:d_{i,j}=1}\bar{J} \le \bar{J}|i[1]| \le \gamma\bar{J}$. We thus prove the inequality (101) by iteratively using the inequality (102). This completes the proof. □

[End of Proof of Lemma 7]

By using the notation $\mathcal{D}$, we simplify the inequality (93) as follows:

$$\left|\frac{d}{d\tau}M_X^{(s)}(\tau)\right| \le 2\sum_{s_1=0}^{s-1}\mathrm{tr}\left[(\hat{n}_X+1)^{s_1}\mathcal{D}(\hat{n}_X)(\hat{n}_X+1)^{s-s_1-1}\rho(\tau)\right]. \tag{103}$$

Also, by using the same analytical techniques, we upper-bound the derivative

$$\left|\frac{d}{d\tau}\sum_{s_1=0}^{s-1}\mathrm{tr}\left[(\hat{n}_X+1)^{s_1}\mathcal{D}(\hat{n}_X)(\hat{n}_X+1)^{s-s_1-1}\rho(\tau)\right]\right| \tag{104}$$

in the form of

$$\begin{aligned}
&\left|\frac{d}{d\tau}\sum_{s_1=0}^{s-1}\mathrm{tr}\left[(\hat{n}_X+1)^{s_1}\mathcal{D}(\hat{n}_X)(\hat{n}_X+1)^{s-s_1-1}\rho(\tau)\right]\right| \\
\leq& 4\sum_{0\leq s_1\leq s_2\leq s-2}\mathrm{tr}\left[(\hat{n}_X+2)^{s_1}[\mathcal{D}(\hat{n}_X)+2\gamma\bar{J}](\hat{n}_X+2)^{s_2-s_1}[\mathcal{D}(\hat{n}_X)+2\gamma\bar{J}](\hat{n}_X+2)^{s-s_2-2}\rho(\tau)\right] \\
&+2\sum_{0\leq s_1\leq s-1}\mathrm{tr}\left[(\hat{n}_X+2)^{s_1}\mathcal{D}^2(\hat{n}_X)(\hat{n}_X+2)^{s-s_1-1}\rho(\tau)\right]. \qquad (105)
\end{aligned}$$

Here, by changing as $\hat{n}_i \to \hat{n}_i + 1$ $(i \in X)$ in using Proposition 6, we need to perform the replacement of

$$\mathcal{D}(\hat{n}_X) \to \mathcal{D}(\hat{n}_X) + 2\gamma\bar{J} \qquad (106)$$

from the inequality (101) in Lemma 7.

The challenging task here is to further simplify the upper bounds like (105) in considering the higher order derivatives. As a key technique, we prove the following proposition:

Proposition 8. *Let us define*

$$\tilde{\mathcal{K}}^m(\hat{n}_X^s) := \sum_{m_1+\cdots+m_s=m}\frac{m!}{m_1!\cdots m_s!}\left[\mathcal{D}^{m_1}(\hat{n}_X)+m(2\gamma\bar{J})^{m_1}\right]\cdots\left[\mathcal{D}^{m_s}(\hat{n}_X)+m(2\gamma\bar{J})^{m_s}\right], \qquad (107)$$

$$M_X^{(s,m)}(\tau) := \mathrm{tr}\left[\tilde{\mathcal{K}}^m(\hat{n}_X^s)\rho(\tau)\right]. \qquad (108)$$

It is evident that $\tilde{\mathcal{K}}^0(\hat{n}_X^s) = \hat{n}_X^s$*, and hence* $M_X^{(s,0)}(\tau) = \mathrm{tr}\,(\hat{n}_X^s\rho(\tau))$*. Then, we can prove that*

$$\left|\frac{d}{d\tau}M_X^{(s,m)}(\tau)\right| \leq 2M_X^{(s,m+1)}(\tau) \quad \textit{for} \quad \forall m \geq 0. \qquad (109)$$

Remark. In considering $M_{X_1,X_2,\ldots,X_s}(\tau)$ in Eq. (71), we need to replace the inequality (109) as follows:

$$\left|\frac{d}{d\tau}M_{X_1,X_2,\ldots,X_s}^{(s,m)}(\tau)\right| \leq 2M_{X_1,X_2,\ldots,X_s}^{(s,m+1)}(\tau) \quad \text{for} \quad \forall m \geq 0. \qquad (110)$$

with

$$\tilde{\mathcal{K}}^m(\hat{n}_{X_1}\hat{n}_{X_2}\cdots\hat{n}_{X_s}) := \sum_{m_1+\cdots+m_s=m}\frac{m!}{m_1!\cdots m_s!}\left[\mathcal{D}^{m_1}(\hat{n}_{X_1})+m(2\gamma\bar{J})^{m_1}\right]\cdots\left[\mathcal{D}^{m_s}(\hat{n}_{X_s})+m(2\gamma\bar{J})^{m_s}\right], \qquad (111)$$

$$M_{X_1,X_2,\ldots,X_s}^{(s,m)}(\tau) := \mathrm{tr}\left[\tilde{\mathcal{K}}^m(\hat{n}_{X_1}\hat{n}_{X_2}\cdots\hat{n}_{X_s})\rho(\tau)\right]. \qquad (112)$$

We notice that the proof for the inequality (110) is exactly same as that for (109).

3. *Proof of Proposition 8*

To derive the upper bound (109), we start from the differential equation for $M_X^{(s,m)}(\tau)$:

$$\begin{aligned}
\frac{d}{d\tau}M_X^{(s,m)}(\tau) &= -i\mathrm{tr}\left(\sum_{m_1+\cdots+m_s=m}\frac{m!}{m_1!\cdots m_s!}\left(\mathcal{D}^{m_1}(\hat{n}_X)+m(2\gamma\bar{J})^{m_1}\right)\cdots\left(\mathcal{D}^{m_s}(\hat{n}_X)+m(2\gamma\bar{J})^{m_s}\right)[H,\rho(\tau)]\right) \\
&= i\mathrm{tr}\left(\sum_{m_1+\cdots+m_s=m}\frac{m!}{m_1!\cdots m_s!}\left[H,\left(\mathcal{D}^{m_1}(\hat{n}_X)+m(2\gamma\bar{J})^{m_1}\right)\cdots\left(\mathcal{D}^{m_s}(\hat{n}_X)+m(2\gamma\bar{J})^{m_s}\right)\right]\rho(\tau)\right) \\
&= i\sum_{m_1+\cdots+m_s=m}\frac{m!}{m_1!\cdots m_s!}\sum_{k=1}^{s}\mathrm{tr}\left(\mathcal{D}_{<k}^{(m)}\left[H,\mathcal{D}^{m_k}(\hat{n}_X)\right]\mathcal{D}_{>k}^{(m)}\rho(\tau)\right), \qquad (113)
\end{aligned}$$

where we have defined

$$\mathcal{D}_{<k}^{(m)} := \prod_{l<k}[\mathcal{D}^{m_l}(\hat{n}_X)+m(2\gamma\bar{J})^{m_l}], \quad \mathcal{D}_{>k}^{(m)} := \prod_{l>k}[\mathcal{D}^{m_l}(\hat{n}_X)+m(2\gamma\bar{J})^{m_l}]. \qquad (114)$$

The form of the Hamiltonian (26) gives

$$[H,\mathcal{D}^{m_k}(\hat{n}_X)] = [H_0,\mathcal{D}^{m_k}(\hat{n}_X)], \qquad (115)$$

where we have used $[V, \mathcal{D}^{m_k}(\hat{n}_X)] = 0$ because of $[\hat{n}_i, \hat{n}_j] = 0$ for $\forall i, j \in \Lambda$. To estimate the upper bound of the norm of (115), we start from the case of $m_k = 0$, i.e., $[H_0, \mathcal{D}^{m_k}(\hat{n}_X)] = [H_0, \mathcal{D}^0(\hat{n}_X)] = [H_0, \hat{n}_X]$. In this case, we have already obtained Eqs. (84) and (85), which give

$$[H_0, \mathcal{D}^0(\hat{n}_X)] = \sum_{i\in\partial X}\sum_{j\in X^{\rm c}:d_{i,j}=1} J_{i,j}\left(b_i b_j^\dagger - b_i^\dagger b_j\right). \tag{116}$$

We thus obtain

$$\begin{aligned}\left|{\rm tr}\left(\mathcal{D}_{<k}^{(m)}\left[H, \mathcal{D}^{m_k}(\hat{n}_X)\right]\mathcal{D}_{>k}^{(m)}\rho(\tau)\right)\right| &\le \sum_{i\in\partial X}\sum_{j\in X^{\rm c}:d_{i,j}=1}\left|J_{i,j}{\rm tr}\left(\mathcal{D}_{<k}^{(m)}\left(b_i b_j^\dagger - b_i^\dagger b_j\right)\mathcal{D}_{>k}^{(m)}\rho(\tau)\right)\right| \\ \le \sum_{i\in\partial X}\sum_{j\in X^{\rm c}:d_{i,j}=1}\bar{J}\left(\left|{\rm tr}\left(\mathcal{D}_{<k}^{(m)}b_i b_j^\dagger\mathcal{D}_{>k}^{(m)}\rho(\tau)\right)\right| + \left|{\rm tr}\left(\mathcal{D}_{<k}^{(m)}b_i^\dagger b_j\mathcal{D}_{>k}^{(m)}\rho(\tau)\right)\right|\right).\end{aligned} \tag{117}$$

We here need to obtain an upper bound for

$$\left|{\rm tr}\left(\mathcal{D}_{<k}^{(m)}b_i b_j^\dagger\mathcal{D}_{>k}^{(m)}\rho(\tau)\right)\right| \quad \text{and} \quad \left|{\rm tr}\left(\mathcal{D}_{<k}^{(m)}b_i^\dagger b_j\mathcal{D}_{>k}^{(m)}\rho(\tau)\right)\right|. \tag{118}$$

For the purpose, we apply the proposition 6. Here, because of the inequality (101) in Lemma 7, when one of $\{\hat{n}_i\}_{i\in X_{m_l}}$ increases as $\hat{n}_i \to \hat{n}_i + 1$, the operator $\mathcal{D}^{m_l}(\hat{n}_X)$ is replaced by

$$\mathcal{D}^{m_l}(\hat{n}_X) \to \mathcal{D}^{m_l}(\hat{n}_X) + (2\gamma\bar{J})^{m_l}, \tag{119}$$

which leads to the replacement of

$$\mathcal{D}_{<k}^{(m)} \to \mathcal{D}_{<k}^{(m+1)}, \quad \mathcal{D}_{>k}^{(m)} \to \mathcal{D}_{>k}^{(m+1)} \tag{120}$$

from the definitions (114). We thus apply the inequality (88) to $\left|{\rm tr}\left(\mathcal{D}_{<k}^{(m)}b_i b_j^\dagger\mathcal{D}_{>k}^{(m)}\rho(\tau)\right)\right|$ and obtain

$$\left|{\rm tr}\left(\mathcal{D}_{<k}^{(m)}b_i b_j^\dagger\mathcal{D}_{>k}^{(m)}\rho(\tau)\right)\right| \le {\rm tr}\left(\mathcal{D}_{<k}^{(m+1)}(\hat{n}_i + \hat{n}_j)\mathcal{D}_{>k}^{(m+1)}\rho(\tau)\right). \tag{121}$$

We can obtain the same upper bound for $\left|{\rm tr}\left(\mathcal{D}_{<k}^{(m)}b_i^\dagger b_j\mathcal{D}_{>k}^{(m)}\rho(\tau)\right)\right|$.

By combining the inequality (121) with (117), we obtain

$$\begin{aligned}\left|{\rm tr}\left(\mathcal{D}_{<k}^{(m)}\left[H, \mathcal{D}^0(\hat{n}_X)\right]\mathcal{D}_{>k}^{(m)}\rho(\tau)\right)\right| &\le 2{\rm tr}\left(\mathcal{D}_{<k}^{(m+1)}\sum_{i\in\partial X}\sum_{j\in X^{\rm c}:d_{i,j}=1}\bar{J}(\hat{n}_i + \hat{n}_j)\mathcal{D}_{>k}^{(m+1)}\rho(\tau)\right) \\ &\le 2{\rm tr}\left(\mathcal{D}_{<k}^{(m+1)}\sum_{i\in\partial X}\left(\gamma\bar{J}\hat{n}_i + \sum_{j\in\Lambda:d_{i,j}=1}\bar{J}\hat{n}_j\right)\mathcal{D}_{>k}^{(m+1)}\rho(\tau)\right) \\ &= 2{\rm tr}\left(\mathcal{D}_{<k}^{(m+1)}\mathcal{D}(\hat{n}_X)\mathcal{D}_{>k}^{(m+1)}\rho(\tau)\right),\end{aligned} \tag{122}$$

where we use the definitions (95) and (99) to obtain $\mathcal{D}(\hat{n}_X) = \sum_{i\in\partial X}\left(\gamma\bar{J}\hat{n}_i + \sum_{j\in\Lambda:d_{i,j}=1}\bar{J}\hat{n}_j\right)$.

For general m_k that is larger than 0, we use the same analyses to estimate the upper bound of the norm of (115). By expanding as $\mathcal{D}^{m_k}(\hat{n}_X) = \sum_{i\in X_{m_k}}\alpha_i^{(m_k)}\hat{n}_i$, we have

$$[H, \mathcal{D}^{m_k}(\hat{n}_X)] = \sum_{i\in X_{m_k}}\alpha_i^{(m_k)}\sum_{j\in\Lambda:d_{i,j}=1}J_{i,j}\left(b_i b_j^\dagger - b_i^\dagger b_j\right). \tag{123}$$

in the same way as the derivation of Eq. (116). We therefore obtain a similar inequality to (117) as follows:

$$\begin{aligned}\left|{\rm tr}\left(\mathcal{D}_{<k}^{(m)}\left[H, \mathcal{D}^{m_k}(\hat{n}_X)\right]\mathcal{D}_{>k}^{(m)}\rho(\tau)\right)\right| &\le 2\bar{J}\sum_{i\in X_{m_k}}\sum_{j\in\Lambda:d_{i,j}=1}\alpha_i^{(m_k)}\left|{\rm tr}\left(\mathcal{D}_{<k}^{(m)}b_i b_j^\dagger\mathcal{D}_{>k}^{(m)}\rho(\tau)\right)\right| \\ &\le 2\sum_{i\in X_{m_k}}\alpha_i^{(m_k)}{\rm tr}\left[\mathcal{D}_{<k}^{(m+1)}\left(\gamma\bar{J}\hat{n}_i + \sum_{j\in\Lambda:d_{i,j}=1}\bar{J}\hat{n}_j\right)\mathcal{D}_{>k}^{(m+1)}\rho(\tau)\right],\end{aligned} \tag{124}$$

where in the last inequality we use the inequality (121). Then, from the definitions (95) and (99), we have

$$\sum_{i\in X_{m_k}}\alpha_i^{(m_k)}\left(\gamma\bar{J}\hat{n}_i + \sum_{j\in\Lambda:d_{i,j}=1}\bar{J}\hat{n}_j\right) = \sum_{i\in X_{m_k}}\alpha_i^{(m_k)}\mathcal{M}(\hat{n}_i) = \mathcal{M}(\mathcal{D}^{m_k}(\hat{n}_X)) = \mathcal{D}^{m_k+1}(\hat{n}_X), \tag{125}$$

which reduces the inequality (124) to

$$\left|\mathrm{tr}\left(\mathcal{D}_{<k}^{(m+1)}\left[H,\mathcal{D}^{m_k}(\hat{n}_X)\right]\mathcal{D}_{>k}^{(m+1)}\rho(\tau)\right)\right| \le 2\mathrm{tr}\left[\mathcal{D}_{<k}^{(m+1)}\mathcal{D}^{m_k+1}(\hat{n}_X)\mathcal{D}_{>k}^{(m+1)}\rho(\tau)\right] \quad \text{for} \quad m_k>0. \tag{126}$$

By combining the inequalities (122) and (126), we obtain

$$\left|\mathrm{tr}\left(\mathcal{D}_{<k}^{(m+1)}\left[H,\mathcal{D}^{m_k}(\hat{n}_X)\right]\mathcal{D}_{>k}^{(m+1)}\rho(\tau)\right)\right| \le 2\mathrm{tr}\left[\mathcal{D}_{<k}^{(m+1)}\mathcal{D}^{m_k+1}(\hat{n}_X)\mathcal{D}_{>k}^{(m+1)}\rho(\tau)\right] \tag{127}$$

for arbitrary $m_k \ge 0$. Hence, from Eq. (113), we arrive at the desired inequality (109):

$$\begin{aligned}\left|\frac{d}{d\tau}M_X^{(s,m)}(\tau)\right| &\le \sum_{m_1+\cdots+m_s=m}\frac{m!}{m_1!\cdots m_s!}\sum_{k=1}^{s}\left|\mathrm{tr}\left(\mathcal{D}_{<k}^{(m+1)}\left[H,\mathcal{D}^{m_k}(\hat{n}_X)\right]\mathcal{D}_{>k}^{(m+1)}\rho(\tau)\right)\right| \\ &\le 2\sum_{m_1+\cdots+m_s=m}\frac{m!}{m_1!\cdots m_s!}\sum_{k=1}^{s}\mathrm{tr}\left(\mathcal{D}_{<k}^{(m+1)}\mathcal{D}^{m_k+1}(\hat{n}_X)\mathcal{D}_{>k}^{(m+1)}\rho(\tau)\right) \\ &\le 2\mathrm{tr}\left[\tilde{\mathcal{K}}^{m+1}(\hat{n}_X^s)\rho(\tau)\right] = 2M_X^{(s,m+1)}(\tau),\end{aligned} \tag{128}$$

where in the last inequality, we use the following inequality:

$$\begin{aligned}&\sum_{m_1+\cdots+m_s=m}\frac{m!}{m_1!\cdots m_s!}\sum_{k=1}^{s}\mathrm{tr}\left(\mathcal{D}_{<k}^{(m+1)}\mathcal{D}^{m_k+1}(\hat{n}_X)\mathcal{D}_{>k}^{(m+1)}\rho(\tau)\right) \\ &\le \sum_{m_1+\cdots+m_s=m}\frac{m!}{m_1!\cdots m_s!}\sum_{k=1}^{s}\mathrm{tr}\left(\mathcal{D}_{<k}^{(m+1)}\left[\mathcal{D}^{m_k+1}(\hat{n}_X)+(m+1)(2\gamma\bar{J})^{m_k+1}\right]\mathcal{D}_{>k}^{(m+1)}\rho(\tau)\right) \\ &= \sum_{k=1}^{s}\sum_{\substack{m_1+\cdots+m_s=m+1\\ m_k\ge 1}}\frac{m!}{m_1!\cdots m_{k-1}!(m_k-1)!m_{k+1}!\cdots m_s!}\mathrm{tr}\left(\prod_i\left[\mathcal{D}^{m_i}(\hat{n}_X)+(m+1)(2\gamma\bar{J})^{m_i}\right]\rho(\tau)\right) \\ &= \sum_{m_1+\cdots+m_s=m+1}\left(\sum_{k=1}^{s}\frac{m_k}{m+1}\right)\frac{(m+1)!}{m_1!\cdots m_s!}\mathrm{tr}\left(\prod_i\left[\mathcal{D}^{m_i}(\hat{n}_X)+(m+1)(2\gamma\bar{J})^{m_i}\right]\rho(\tau)\right) \\ &= \sum_{m_1+\cdots+m_s=m+1}\frac{(m+1)!}{m_1!\cdots m_s!}\mathrm{tr}\left(\prod_i\left[\mathcal{D}^{m_i}(\hat{n}_X)+(m+1)(2\gamma\bar{J})^{m_i}\right]\rho(\tau)\right) = \mathrm{tr}\left(\tilde{\mathcal{K}}^{m+1}(\hat{n}_X^s)\rho(\tau)\right),\end{aligned} \tag{129}$$

where we use the definition (107) in the last equation. This completes the proof. □

———————————————————————— **[End of Proof of Proposition 8]**

Applying Eq. (128) immediately yields

$$M_X^{(s,m)}(\tau) \le M_X^{(s,m)}(0) + 2\int_0^\tau M_X^{(s,m+1)}(\tau)d\tau. \tag{130}$$

For $m=0$, we have

$$M_X^{(s)}(\tau) = M_X^{(s,0)}(\tau) \le M_X^{(s,0)}(0) + 2\int_0^\tau M_X^{(s,1)}(\tau)d\tau. \tag{131}$$

In the same way, by applying the inequality (130) for $m=1$, we have

$$\begin{aligned}M_X^{(s)}(\tau) &\le M_X^{(s,0)}(0) + 2\int_0^\tau\left(M_X^{(s,1)}(0) + 2\int_0^{\tau_1} M_X^{(s,2)}(\tau_2)d\tau_2\right)d\tau_1 \\ &= M_X^{(s,0)}(0) + 2\tau M_X^{(s,1)}(0) + 2^2\int_0^\tau\int_0^{\tau_1} M_X^{(s,2)}(\tau_2)d\tau_2 d\tau_1.\end{aligned} \tag{132}$$

Then, by iteratively repeating this process, we finally obtain

$$M_X^{(s)}(\tau) \le \sum_{m=0}^{\infty}\frac{(2\tau)^m}{m!}M_X^{(s,m)}(0) = \sum_{m=0}^{\infty}\frac{(2\tau)^m}{m!}\mathrm{tr}\left(\tilde{\mathcal{K}}^m(\hat{n}_X^s)\rho\right). \tag{133}$$

Next, we further treat the operator $\tilde{\mathcal{K}}^m(\hat{n}_X^s)$ in Eq. (107):

$$\tilde{\mathcal{K}}^m(\hat{n}_X^s) = \sum_{m_1+\cdots+m_s=m} \frac{m!}{m_1!\cdots m_s!}[\mathcal{D}^{m_1}(\hat{n}_X)+m(2\gamma\bar{J})^{m_1}]\cdots[\mathcal{D}^{m_s}(\hat{n}_X)+m(2\gamma\bar{J})^{m_s}]$$
$$= \sum_{s_1=0}^{s}\sum_{m_1+\cdots+m_s=m} \frac{m!}{m_1!\cdots m_s!}\sum_{\{i_1,\ldots,i_{s_1}\}\subset[s]} \mathcal{D}^{m_{i_1}}(\hat{n}_X)\cdots\mathcal{D}^{m_{i_{s_1}}}(\hat{n}_X)m^{s-s_1}(2\gamma\bar{J})^{m_{j_1}+m_{j_2}+\cdots+m_{j_{s-s_1}}}, \tag{134}$$

where we denote $[s]\setminus\{i_1,i_2,\ldots,i_{s_1}\}=\{j_1,j_2,\ldots,j_{s-s_1}\}$. Then, we use the following relations

$$\tau^m m^{s-s_1} \le \frac{d^{s-s_1}}{d\tau^{s-s_1}}\left(\tau^{m+s-s_1}\right), \quad \sum_{m=0}^{\infty}\sum_{m_1+\cdots+m_s=m} = \sum_{m_1=0}^{\infty}\sum_{m_2=0}^{\infty}\cdots\sum_{m_s=0}^{\infty}, \tag{135}$$

which yields the inequality of

$$\sum_{m=0}^{\infty}\frac{(2\tau)^m}{m!}\tilde{\mathcal{K}}^m(\hat{n}_X^s)$$
$$\preceq \sum_{s_1=0}^{s}\sum_{\{i_1,i_2,\ldots,i_{s_1}\}\subset[s]}\frac{d^{s-s_1}}{d\tau^{s-s_1}}\tau^{s-s_1}\sum_{m_1=0}^{\infty}\frac{(2\tau)^{m_1}}{m_1!}\cdots\sum_{m_s=0}^{\infty}\frac{(2\tau)^{m_s}}{m_s!}\mathcal{D}^{m_{i_1}}(\hat{n}_X)\cdots\mathcal{D}^{m_{i_{s_1}}}(\hat{n}_X)(2\gamma\bar{J})^{m_{j_1}+\cdots+m_{j_{s-s_1}}}$$
$$= \sum_{s_1=0}^{s}\sum_{\{i_1,\ldots,i_{s_1}\}\subset[s]}\frac{d^{s-s_1}}{d\tau^{s-s_1}}\tau^{s-s_1}\left(e^{4\gamma\bar{J}\tau}\right)^{s-s_1}\left(\sum_{m=0}^{\infty}\frac{(2\tau)^m}{m!}\mathcal{D}^m(\hat{n}_X)\right)^{s_1}$$
$$= \sum_{s_1=0}^{s}\sum_{\{i_1,\ldots,i_{s_1}\}\subset[s]}\frac{d^{s'}}{d\tau^{s'}}\left(\tau^{s'}e^{4\gamma\bar{J}s'\tau}\left[\hat{n}_X+\tilde{\mathcal{D}}_\tau(\hat{n}_X)\right]^{s_1}\right), \tag{136}$$

where we set $s'=s-s_1$ and define $\tilde{\mathcal{D}}_\tau(\hat{n}_X)$ as

$$\tilde{\mathcal{D}}_\tau(\hat{n}_X) := \sum_{m=1}^{\infty}\frac{(2\tau)^m}{m!}\mathcal{D}^m(\hat{n}_X). \tag{137}$$

To estimate the upper bound of the derivative in the RHS of (136), we separately consider

$$\frac{d^p}{d\tau^p}\left(\tau^{s'}e^{4\gamma\bar{J}s'\tau}\right), \quad \frac{d^p}{d\tau^p}\left[\hat{n}_X+\tilde{\mathcal{D}}_\tau(\hat{n}_X)\right]^{s_1} \quad \text{for} \quad p\le s'. \tag{138}$$

For the first, we obtain

$$\frac{d^p}{d\tau^p}\left(\tau^{s'}e^{4\gamma\bar{J}s'\tau}\right) = \sum_{p_1=0}^{p}\binom{p}{p_1}\frac{d^{p_1}}{d\tau^{p_1}}\left(\tau^{s'}\right)\frac{d^{p-p_1}}{d\tau^{p-p_1}}e^{4\gamma\bar{J}s'\tau}$$
$$= \sum_{p_1=0}^{p}\binom{p}{p_1}s'(s'-1)\cdots(s'-p_1+1)\tau^{s'-p_1}\left(4\gamma\bar{J}s'\right)^{p-p_1}e^{4\gamma\bar{J}s'\tau}$$
$$\le \tau^{s'-p}\sum_{p_1=0}^{p}\binom{p}{p_1}(s')^{p_1}\tau^{p-p_1}\left(4\gamma\bar{J}s'\right)^{p-p_1}e^{4\gamma\bar{J}s'\tau} \le (s')^p\tau^{s'-p}e^{4\gamma\bar{J}s'\tau}(1+4\gamma\bar{J}\tau)^p. \tag{139}$$

Second, we consider the derivatives of $\left[\hat{n}_X+\tilde{\mathcal{D}}_\tau(\hat{n}_X)\right]^{s_1}$. For convenience, we here define $\tilde{\mathcal{D}}_\tau^{(p)}(\hat{n}_X)$ as

$$\tilde{\mathcal{D}}_\tau^{(p)}(\hat{n}_X) := \frac{d^p}{d\tau^p}\tilde{\mathcal{D}}_\tau(\hat{n}_X). \tag{140}$$

We then prove the following lemma:

Lemma 9. *For an arbitrary integer $p\ge 0$, under the condition of $\tau\le 1/(4\gamma\bar{J})$, we have*

$$\tilde{\mathcal{D}}_\tau^{(p)}(\hat{n}_X) \preceq p!(4\gamma\bar{J})^p\bar{\mathcal{D}}_\tau(\hat{n}_X), \tag{141}$$

where $\bar{\mathcal{D}}_\tau(\hat{n}_X)$ is defined as

$$\bar{\mathcal{D}}_\tau(\hat{n}_X) := 40e^{4\gamma\bar{J}\tau}\sum_{i\in\partial X}\sum_{j\in\Lambda}e^{-d_{i,j}}\hat{n}_j. \tag{142}$$

Note that by considering the case of $p=0$, we obtain the upper bound of

$$\tilde{\mathcal{D}}_\tau(\hat{n}_X) = \tilde{\mathcal{D}}_\tau^{(0)}(\hat{n}_X) \le \bar{\mathcal{D}}_\tau(\hat{n}_X). \tag{143}$$

By using the definitions of $\mathcal{D}$ in Eq. (99), we have

$$\tilde{\mathcal{D}}_\tau(\hat{n}_X) := \sum_{m=1}^{\infty} \frac{(2\tau)^m}{m!} \mathcal{D}^m(\hat{n}_X) = \sum_{i\in\partial X} \sum_{m=1}^{\infty} \frac{(2\tau)^m}{m!} \mathcal{M}^m(\hat{n}_i), \tag{144}$$

and hence,

$$\tilde{\mathcal{D}}_\tau^{(p)}(\hat{n}_X) = \frac{d^p}{d\tau^p} \tilde{\mathcal{D}}_\tau(\hat{n}_X) \preceq 2^p \sum_{i\in\partial X} \sum_{m=p}^{\infty} \frac{(2\tau)^{m-p}}{(m-p)!} \mathcal{M}^m(\hat{n}_i). \tag{145}$$

Now, let us define $(\mathcal{M}^m)_{i,j} \in \mathbb{R}^+$ ($q \in \mathbb{N}$) by

$$\mathcal{M}^m(\hat{n}_i) = \sum_{j\in\Lambda} (\mathcal{M}^m)_{i,j} \hat{n}_j. \tag{146}$$

That is, $(\mathcal{M}^m)_{i,j}$ is the coefficient for $\hat{n}_j$ in expanding $\mathcal{M}^m(\hat{n}_i)$. Then, the definition (95) implies that $(\mathcal{M}^m)_{i,j} = 0$ as long as $m < d_{i,j}$. On the other hand, for $m \ge d_{i,j}$, we have

$$(\mathcal{M}^m)_{i,j} \le (2\gamma \bar{J})^m \quad \text{for} \quad m \ge d_{i,j}, \tag{147}$$

which immediately results from Lemma 7. By applying (146) and (147) to Eq. (145), we have

$$\tilde{\mathcal{D}}_\tau^{(p)}(\hat{n}_X) \preceq \sum_{i\in\partial X} \sum_{j\in\Lambda} (\bar{\mathcal{M}})_{i,j} \hat{n}_j, \tag{148}$$

where the matrix $\bar{\mathcal{M}}$ is defined as

$$\begin{aligned} \left(\bar{\mathcal{M}}\right)_{i,j} &:= 2^p \sum_{m=p}^{\infty} \frac{(2\tau)^{m-p}}{(m-p)!} \left(\mathcal{M}^m\right)_{i,j} = 2^p \sum_{m=0}^{\infty} \frac{(2\tau)^m}{m!} \left(\mathcal{M}^{m+p}\right)_{i,j} \\ &\le 2^p \sum_{m\ge \max\{0, d_{i,j}-p\}}^{\infty} \frac{(2\tau)^m}{m!} (2\gamma\bar{J})^{m+p} \\ &\le \begin{cases} (4\gamma\bar{J})^{d_{i,j}} e^{4\gamma\bar{J}\tau} \tau^{d_{i,j}-p} \frac{1}{(d_{i,j}-p)!} & \text{for} \quad p \le d_{i,j}, \\ (4\gamma\bar{J})^p e^{4\gamma\bar{J}\tau} & \text{for} \quad p > d_{i,j}, \end{cases} \end{aligned} \tag{149}$$

where in the last inequality, we have used

$$\sum_{m\ge m_0} \frac{z^m}{m!} = \sum_{m=0}^{\infty} \frac{z^{m+m_0}}{(m+m_0)!} \le \sum_{m=0}^{\infty} \frac{z^{m+m_0}}{m! \cdot m_0!} = e^z \frac{z^{m_0}}{m_0!}. \tag{150}$$

Finally, by using the inequality

$$\frac{1}{(d_{i,j}-p)!} = \frac{p!}{d_{i,j}!} \binom{d_{i,j}}{p} \le 2^{d_{i,j}} \frac{p!}{d_{i,j}!}, \tag{151}$$

and the condition

$$\tau \le \frac{1}{4\gamma\bar{J}}, \tag{152}$$

we have for $p \le d_{i,j}$

$$(4\gamma\bar{J})^{d_{i,j}} e^{4\gamma\bar{J}\tau} \tau^{d_{i,j}-p} \frac{1}{(d_{i,j}-p)!} \le (4\gamma\bar{J})^p e^{4\gamma\bar{J}\tau} 2^{d_{i,j}} \frac{p!}{d_{i,j}!} = p!(4\gamma\bar{J})^p \cdot e^{4\gamma\bar{J}\tau} \frac{2^{d_{i,j}}}{d_{i,j}!}. \tag{153}$$

In the case of $p > d_{i,j}$ in (149), the following inequality trivially holds:

$$(4\gamma\bar{J})^p e^{4\gamma\bar{J}\tau} \le p!(4\gamma\bar{J})^p \cdot e^{4\gamma\bar{J}\tau} \frac{2^{d_{i,j}}}{d_{i,j}!}. \tag{154}$$

Thus, by applying the inequalities (153) and (154) to (149), we arrive at the inequality of

$$\left(\bar{\mathcal{M}}\right)_{i,j} \le p!(4\gamma\bar{J})^p \cdot e^{4\gamma\bar{J}\tau} \frac{2^{d_{i,j}}}{d_{i,j}!}. \tag{155}$$

Applying this inequality to Eq. (148), we have

$$\tilde{\mathcal{D}}_\tau^{(p)}(\hat{n}_X) \preceq p!(4\gamma\bar{J})^p \sum_{i\in\partial X}\sum_{j\in\Lambda} e^{4\gamma\bar{J}\tau}\frac{2^{d_{i,j}}}{d_{i,j}!}\hat{n}_j,$$
$$\preceq p!(4\gamma\bar{J})^p \cdot e^{4\gamma\bar{J}\tau} \sum_{i\in\partial X}\sum_{j\in\Lambda} \frac{2^{d_{i,j}}}{d_{i,j}!}\hat{n}_j,$$
$$\preceq p!(4\gamma\bar{J})^p \cdot 40e^{4\gamma\bar{J}\tau} \sum_{i\in\partial X}\sum_{j\in\Lambda} e^{-d_{i,j}}\hat{n}_j =: p!(4\gamma\bar{J})^p\bar{\mathcal{D}}_\tau(\hat{n}_X), \tag{156}$$

where in the third inequality, we have applied the following inequality:

$$\frac{2^{d_{i,j}}}{d_{i,j}!} \le 40e^{-d_{i,j}}. \tag{157}$$

This completes the proof. □

———————————————————————————————————— [**End of Proof of Lemma 9**]

By using Lemma 9, we obtain the upper bound of

$$\hat{n}_X + \tilde{\mathcal{D}}_\tau^{(p)}(\hat{n}_X) \le \hat{n}_X + p!(4\gamma\bar{J})^p\bar{\mathcal{D}}_\tau(\hat{n}_X) \le p!(4\gamma\bar{J}+1)^p\left[\hat{n}_X + \bar{\mathcal{D}}_\tau(\hat{n}_X)\right]. \tag{158}$$

By using the above inequality, we upper-bound the derivatives of $\left[\hat{n}_X + \tilde{\mathcal{D}}_\tau(\hat{n}_X)\right]^{s_1}$ as follows:

$$\frac{d^p}{d\tau^p}\left[\hat{n}_X + \tilde{\mathcal{D}}_\tau(\hat{n}_X)\right]^{s_1} \preceq \sum_{\substack{p_1,p_2,\ldots,p_{s_1}\\ p_1+p_2+\cdots+p_{s_1}=p}} \frac{p!}{p_1!p_2!\cdots p_{s_1}!}\prod_{j=1}^{s_1}\left[\hat{n}_X + \tilde{\mathcal{D}}_\tau^{(p_j)}(\hat{n}_X)\right]$$
$$\preceq \sum_{\substack{p_1,p_2,\ldots,p_{s_1}\\ p_1+p_2+\cdots+p_{s_1}=p}} \frac{p!}{p_1!p_2!\cdots p_{s_1}!}(4\gamma\bar{J}+1)^{p_1+p_2+\cdots+p_{s_1}}p_1!p_2!\cdots p_{s_1}!\left[\hat{n}_X + \bar{\mathcal{D}}_\tau(\hat{n}_X)\right]^{s_1}$$
$$= (4\gamma\bar{J}+1)^p p! \sum_{\substack{p_1,p_2,\ldots,p_{s_1}\\ p_1+p_2+\cdots+p_{s_1}=p}} \left[\hat{n}_X + \bar{\mathcal{D}}_\tau(\hat{n}_X)\right]^{s_1}$$
$$= (4\gamma\bar{J}+1)^p p! \left(\!\!\binom{s_1}{p}\!\!\right)\left[\hat{n}_X + \bar{\mathcal{D}}_\tau(\hat{n}_X)\right]^{s_1}, \tag{159}$$

where $\left(\!\binom{s_1}{p}\!\right)$ is the (p)-multicombination from a set of s_1 elements, which satisfies

$$\left(\!\!\binom{s_1}{p}\!\!\right) = \binom{s_1+p-1}{p}. \tag{160}$$

By combining the inequalities (139) and (159), we obtain

$$\frac{d^{s'}}{d\tau^{s'}}\left(\tau^{s'}e^{4\gamma\bar{J}s'\tau}\left[\hat{n}_X + \tilde{\mathcal{D}}_\tau(\hat{n}_X)\right]^{s_1}\right)$$
$$= \sum_{p=0}^{s'}\binom{s'}{p}\frac{d^p}{d\tau^p}\left(\tau^{s'}e^{4\gamma\bar{J}s'\tau}\right)\frac{d^{s'-p}}{d\tau^{s'-p}}\left[\hat{n}_X + \tilde{\mathcal{D}}_\tau(\hat{n}_X)\right]^{s_1}$$
$$\preceq \sum_{p=0}^{s'}\binom{s'}{p}(s')^p\tau^{s'-p}e^{4\gamma\bar{J}s'\tau}(1+4\gamma\bar{J}\tau)^p\cdot(4\gamma\bar{J}+1)^{s'-p}(s'-p)!\left(\!\!\binom{s_1}{s'-p}\!\!\right)\left[\hat{n}_X + \bar{\mathcal{D}}_\tau(\hat{n}_X)\right]^{s_1}$$
$$\preceq (s')^{s'}e^{4\gamma\bar{J}s'\tau}\left(\!\!\binom{s_1}{s'}\!\!\right)\left[\hat{n}_X + \bar{\mathcal{D}}_\tau(\hat{n}_X)\right]^{s_1}\sum_{p=0}^{s'}\binom{s'}{p}\left(1+4\gamma\bar{J}\tau\right)^p\left[\tau(4\gamma\bar{J}+1)\right]^{s'-p}$$
$$= \left(\!\!\binom{s_1}{s'}\!\!\right)(s')^{s'}\left[e^{4\gamma\bar{J}\tau}\left(1+8\gamma\bar{J}\tau+\tau\right)\right]^{s'}\left[\hat{n}_X + \bar{\mathcal{D}}_\tau(\hat{n}_X)\right]^{s_1}$$
$$\preceq \left[e^{4\gamma\bar{J}\tau+1}\left(1+8\gamma\bar{J}\tau+\tau\right)s\right]^{s'}\left[\hat{n}_X + \bar{\mathcal{D}}_\tau(\hat{n}_X)\right]^{s_1}, \tag{161}$$

where in the second inequality, we use the fact that the multiset $\left(\!\binom{s_1}{p}\!\right)$ monotonically increases with p, and in the last inequality, we use $s' = s - s_1$ and

$$\left(\!\binom{s_1}{s'}\!\right)(s')^{s'} \le e^{s'}\binom{s_1+s'-1}{s'}s'! = e^{s'}\binom{s-1}{s'}s'! = e^{s'}\frac{(s-1)!}{(s-1-s')!} \le (es)^{s'}. \tag{162}$$

Note that $s'! \ge (s'/e)^{s'}$. By applying the inequality (161) to (136), we finally obtain

$$\begin{aligned}\sum_{m=0}^{\infty}\frac{(2\tau)^m}{m!}\tilde{\mathcal{K}}^m(\hat{n}_X^s) &\preceq \sum_{s_1=0}^{s}\sum_{\{i_1,\ldots,i_{s_1}\}\subset[s]}\left[e^{4\gamma\bar{J}\tau+1}\left(1+8\gamma\bar{J}\tau+\tau\right)s\right]^{s'}\left[\hat{n}_X+\bar{\mathcal{D}}_\tau(\hat{n}_X)\right]^{s_1} \\ &= \sum_{s_1=0}^{s}\binom{s}{s_1}\left[e^{4\gamma\bar{J}\tau+1}\left(1+8\gamma\bar{J}\tau+\tau\right)s\right]^{s'}\left[\hat{n}_X+\bar{\mathcal{D}}_\tau(\hat{n}_X)\right]^{s_1} \\ &= \left[\hat{n}_X+40e^{4\gamma\bar{J}\tau}\sum_{i\in\partial X}\sum_{j\in\Lambda}e^{-d_{i,j}}\hat{n}_j+e^{4\gamma\bar{J}\tau+1}\left(1+8\gamma\bar{J}\tau+\tau\right)s\right]^{s'},\end{aligned} \tag{163}$$

where in the last equation, we use the explicit form (142) of $\bar{\mathcal{D}}_\tau(\hat{n}_X)$. Hence, by choosing $c_{\tau,1}$ and $c_{\tau,2}$ as in Eq. (76), we obtain the main inequality (74). This completes the proof. □

C. More general statement

In some cases, we are interested in the moment function in the form of $|\hat{n}_X - N|^s$ instead of $\hat{n}_X^s$, where N is an arbitrary integer. In particular, we indeed utilize it in deriving Proposition 22 below, which plays a critical role in proving the optimal Lieb-Robinson bound in one-dimensional systems (see Sec. Supplementary Note 10 E 2 for the proof of Lemma 51). We here consider

$$\begin{aligned}&M'_{X_1,X_2,\ldots,X_s}(\tau) := \mathrm{tr}\left[\hat{n}'_{X_1}\hat{n}'_{X_2}\cdots\hat{n}'_{X_s}\rho(\tau)\right], \\ &\hat{n}'_{X_j} := |\hat{n}_{X_j} - N_j| \quad \text{for} \quad j = 1,2,\ldots,s,\end{aligned} \tag{164}$$

where $\{N_j\}_{j=1}^s$ are arbitrary positive numbers. For $M'_{X_1,X_2,\ldots,X_s}(\tau)$, we can prove the same statement as Subtheorem 1.

Proposition 10. *Let ρ be an arbitrary quantum state, and $\{X_j\}_{j=1}^s$ be arbitrary subsets. Then, as long as $\tau \le 1/(4\gamma\bar{J})$, we obtain the following upper bound for $M'_{X_1,X_2,\ldots,X_s}(\tau)$:*

$$M'_{X_1,X_2,\ldots,X_s}(\tau) \le \mathrm{tr}\left[\prod_{j=1}^{s}\left(\hat{n}'_{X_j}+c_{\tau,1}\hat{\mathcal{D}}_{X_j}+c_{\tau,2}s\right)\rho\right], \tag{165}$$

where $\hat{\mathcal{D}}_X$, $c_{\tau,1}$ and $c_{\tau,2}$ have been defined in Eqs. (75) and (76), respectively.

1. *Proof of Proposition 10*

For the proof, we utilize the same technique. Here, we slightly extend the definition of the super-operator $\mathcal{D}$ in Eq. (99) as follows:

$$\begin{aligned}&\mathcal{D}^0(\hat{n}'_X) := \hat{n}'_X, \quad \mathcal{D}^1(\hat{n}'_X) := \sum_{i\in\partial X}\mathcal{M}(\hat{n}_i), \\ &\mathcal{D}^{m+1}(\hat{n}_X) := \mathcal{M}(\mathcal{D}^m(\hat{n}_X)) \quad \text{for} \quad \forall m \ge 1\end{aligned} \tag{166}$$

Note that we have

$$\mathcal{D}^m(\hat{n}'_X) := \mathcal{D}^m(\hat{n}_X) \quad \text{for} \quad m \ge 1. \tag{167}$$

Then, if the same statement as Proposition 8 is proved, we can derive the main inequality (165) by employing the same analyses as in the proof of Subtheorem 1. Note that we only replace the inequality (136) by

$$\sum_{m=0}^{\infty}\frac{(2\tau)^m}{m!}\tilde{\mathcal{K}}^m(\hat{n}_X^s) \preceq \sum_{s_1=0}^{s}\sum_{\{i_1,\ldots,i_{s_1}\}\subset[s]}\frac{d^{s'}}{d\tau^{s'}}\left(\tau^{s'}e^{4\gamma\bar{J}s'\tau}\left[\hat{n}'_X+\tilde{\mathcal{D}}_\tau(\hat{n}_X)\right]^{s_1}\right), \tag{168}$$

where we can adopt the same definition (137) for $\tilde{\mathcal{D}}_\tau(\hat{n}_X)$ because of Eq. (167).

For the proof of Proposition 8 in the present case, we start from the equation (113) as follows:

$$\frac{d}{d\tau}M_X'^{(s,m)}(\tau) = i \sum_{m_1+\cdots+m_s=m} \frac{m!}{m_1!\cdots m_s!} \sum_{k=1}^{s} \mathrm{tr}\left(\mathcal{D}_{<k}^{(m)} [H, \mathcal{D}^{m_k}(\hat{n}_X')] \mathcal{D}_{>k}^{(m)} \rho(\tau)\right) \tag{169}$$

with

$$\mathcal{D}_{<k}^{(m)} := \prod_{l<k} [\mathcal{D}^{m_l}(\hat{n}_X') + m(2\gamma\bar{J})^{m_l}], \quad \mathcal{D}_{>k}^{(m)} := \prod_{l>k} [\mathcal{D}^{m_l}(\hat{n}_X') + m(2\gamma\bar{J})^{m_l}]. \tag{170}$$

The equation (115) gives

$$[H, \mathcal{D}^{m_k}(\hat{n}_X')] = [H_0, \mathcal{D}^{m_k}(\hat{n}_X')], \tag{171}$$

For $m_k = 0$, because of

$$[\hat{n}_X', H_{0,X}] = 0, \ \& \ [\hat{n}_X', H_{0,X^c}] = 0, \tag{172}$$

we have

$$\begin{aligned}[H_0, \mathcal{D}^0(\hat{n}_X')] = [\partial h_X, \hat{n}_X'] &= \sum_{i\in\partial X} \sum_{j\in X^c: d_{i,j}=1} J_{i,j} [b_i b_j^\dagger + b_j b_i^\dagger, \hat{n}_X'] \\ &= \sum_{i\in\partial X} \sum_{j\in X^c: d_{i,j}=1} J_{i,j} [b_{X,i}' b_j^\dagger - b_j (b_{X,i}')^\dagger],\end{aligned} \tag{173}$$

where we use the notation of (28) and define

$$b_{X,i}' := [b_i, \hat{n}_X'] = [b_i, |\hat{n}_X - N|]. \tag{174}$$

Clearly, for $N=0$ (i.e., $|\hat{n}_X - N| = |\hat{n}_X| = \hat{n}_X$), we have $b_{X,i}' = [b_i, \hat{n}_i] = b_i$. By using the notation, we obtain a similar inequality to (117):

$$\begin{aligned}&\left|\mathrm{tr}\left(\mathcal{D}_{<k}^{(m)} [H, \mathcal{D}^{m_k}(\hat{n}_X)] \mathcal{D}_{>k}^{(m)} \rho(\tau)\right)\right| \\ &\le \sum_{i\in\partial X} \sum_{j\in X^c: d_{i,j}=1} \bar{J} \left(\left|\mathrm{tr}\left(\mathcal{D}_{<k}^{(m)} b_{X,i}' b_j^\dagger \mathcal{D}_{>k}^{(m)} \rho(\tau)\right)\right| + \left|\mathrm{tr}\left(\mathcal{D}_{<k}^{(m)} (b_{X,i}')^\dagger b_j \mathcal{D}_{>k}^{(m)} \rho(\tau)\right)\right| \right).\end{aligned} \tag{175}$$

Then, the primary problem is to derive upper bounds for

$$\left|\mathrm{tr}\left(\mathcal{D}_{<k}^{(m)} b_{X,i}' b_j^\dagger \mathcal{D}_{>k}^{(m)} \rho(\tau)\right)\right| \quad \text{and} \quad \left|\mathrm{tr}\left(\mathcal{D}_{<k}^{(m)} (b_{X,i}')^\dagger b_j \mathcal{D}_{>k}^{(m)} \rho(\tau)\right)\right|. \tag{176}$$

We note that the operator $b_{X,i}'$ is a non-local operator supported on the whole region of X. However, by taking the upper bound, the non-locality disappears as in (184), where $(b_{X,i}')^\dagger b_j$ is simply upper-bounded by using $\hat{n}_i + \hat{n}_j$.

We here extend the proposition 6 as follows:

Lemma 11. *Let us assume $i \in X$ and $j \in X^c$. Then, for arbitrary s_0, s_1, s_1', s_2 and s_2', we obtain the upper bound as*

$$\begin{aligned}&\left|\mathrm{tr}\left[f(\hat{\mathbf{n}})(\hat{n}_X')^{s_0} \hat{n}_i^{s_1} \hat{n}_j^{s_2} b_{X,i}' b_j^\dagger (\hat{n}_X')^{s_0'} \hat{n}_i^{s_1'} \hat{n}_j^{s_2'} \rho(\tau)\right]\right| \\ &\le \frac{1}{2}\left[f(\hat{\mathbf{n}})(\hat{n}_X'+1)^{s_0+s_0'} \left[\hat{n}_i^{s_1+s_1'}(\hat{n}_j+1)^{s_2+s_2'} + (\hat{n}_i+1)^{s_1+s_1'}\hat{n}_j^{s_2+s_2'}\right](\hat{n}_i+\hat{n}_j)\rho(\tau)\right],\end{aligned} \tag{177}$$

where $f(\hat{\mathbf{n}})$ is an arbitrary non-negative function with respect to $\{\hat{n}_s\}_{s\neq i,j}$ or equivalently satisfies $[f(\hat{\mathbf{n}}), b_i] = [f(\hat{\mathbf{n}}), b_j] = 0$. We have the same inequality if we replace $b_{X,i}' b_j^\dagger$ with $(b_{X,i}')^\dagger b_j$.

2. Proof of Lemma 11

We use the notation of $\mathbf{N} = \{N_i, \mathbf{N}_i\}$, where $\mathbf{N}_i$ includes the boson number in $\Lambda \setminus \{i\}$. In the same way, we define $\mathbf{N} = \{N_i, N_j, \mathbf{N}_{i,j}\}$, where $\mathbf{N}_{i,j}$ includes the boson number in $\Lambda \setminus \{i,j\}$.

For the proof, we first consider the explicit form of the operator $b'_{X,i}$:

$$\begin{aligned}
b'_{X,i} &:= [b_i, \hat{n}'_X] \\
&= \sum_{N_{X_i}=0}^{\infty}\sum_{N_i=0}^{\infty} |N_i + N_{X_i} - N| \cdot [b_i, |N_i, N_{X_i}\rangle\langle N_i, N_{X_i}|] \\
&= \sum_{N_{X_i}=0}^{\infty}\sum_{N_i=0}^{\infty} |N_i + N_{X_i} - N| \left(\sqrt{N_i}|N_i - 1, N_{X_i}\rangle\langle N_i, N_{X_i}| - \sqrt{N_i+1}|N_i, N_{X_i}\rangle\langle N_i+1, N_{X_i}|\right) \\
&= \sum_{N_{X_i}=0}^{\infty}\sum_{N_i=0}^{\infty} (|N_i + N_{X_i} - N| - |N_i - 1 + N_{X_i} - N|)\sqrt{N_i}|N_i - 1, N_{X_i}\rangle\langle N_i, N_{X_i}| \\
&=: \sum_{N_{X_i}=0}^{\infty}\sum_{N_i=0}^{\infty} g(N_i, N_{X_i}, N)\sqrt{N_i}|N_i - 1, N_{X_i}\rangle\langle N_i, N_{X_i}|,
\end{aligned} \tag{178}$$

where we define $g(N_i, N_{X_i}, N) := |N_i + N_{X_i} - N| - |N_i - 1 + N_{X_i} - N|$. From the definition, we trivially obtain

$$|g(N_i, N_{X_i}, N)| \le 1. \tag{179}$$

We therefore obtain

$$\begin{aligned}
b_{X,i}|\mathbf{N}\rangle &= g(N_i, N_{X_i}, N)\sqrt{N_i}|N_i - 1, N_j, \mathbf{N}_{i,j}\rangle, \\
(b_{X,i})^\dagger|\mathbf{N}\rangle &= g(N_i + 1, N_{X_i}, N)\sqrt{N_i+1}|N_i + 1, N_j, \mathbf{N}_{i,j}\rangle.
\end{aligned} \tag{180}$$

Because of $i \in X$ and $j \in X^{\mathrm{c}}$, we have $b'_{X,i}b_j^\dagger|\mathbf{N}\rangle \propto |N_i - 1, N_j + 1, \mathbf{N}_{i,j}\rangle$ and $\hat{n}'_X b'_{X,i}b_j^\dagger|\mathbf{N}\rangle = |N_X - 1 - N|^{s_0} b'_{X,i}b_j^\dagger|\mathbf{N}\rangle$. Hence, by using Eq. (180), we transform

$$\begin{aligned}
&\mathrm{tr}\left[f(\hat{\mathbf{n}})(\hat{n}'_X)^{s_0}\hat{n}_i^{s_1}\hat{n}_j^{s_2}b'_{X,i}b_j^\dagger\hat{n}_i^{s'_1}\hat{n}_j^{s'_2}\rho(\tau)\right] = \mathrm{tr}\left[f(\hat{\mathbf{n}})(\hat{n}'_X)^{s_0}\hat{n}_i^{s_1}\hat{n}_j^{s_2}b'_{X,i}b_j^\dagger\hat{n}_i^{s'_1}\hat{n}_j^{s'_2}\sum_{\mathbf{N}}P_{\mathbf{N}}\rho(\tau)\right] \\
&= \sum_{\mathbf{N}} f(\mathbf{N}_{i,j})|N_X - N|^{s'_0}N_i^{s'_1}N_j^{s'_2}\sqrt{N_i}\sqrt{N_j+1}|N_X - 1 - N|^{s_0}(N_i - 1)^{s_1}(N_j+1)^{s_2} \\
&\quad \times g(N_i, N_{X_i}, N)\langle N_i, N_j, \mathbf{N}_{i,j}|\rho(\tau)|N_i - 1, N_j + 1, \mathbf{N}_{i,j}\rangle \\
&\le \sum_{\mathbf{N}} f(\mathbf{N}_{i,j})|N_X - N|^{s'_0}N_i^{s'_1}N_j^{s'_2}\sqrt{N_i}\sqrt{N_j+1}|N_X - 1 - N|^{s_0}(N_i - 1)^{s_1}(N_j+1)^{s_2} \\
&\quad \times \frac{1}{2}\left(\langle N_i, N_j, \mathbf{N}_{i,j}|\rho(\tau)|N_i, N_j, \mathbf{N}_{i,j}\rangle + \langle N_i - 1, N_j + 1, \mathbf{N}_{i,j}|\rho(\tau)|N_i - 1, N_j + 1, \mathbf{N}_{i,j}\rangle\right),
\end{aligned} \tag{181}$$

where we use the inequalities (90) and (179) in the last inequality. Then, by using $\sqrt{N_i(N_j+1)} \le N_i + N_j$ and $\sqrt{(N_i+1)N_j} \le N_i + N_j$ for $\forall N_i, N_j \in \mathbb{N}$, we can reduce

$$\begin{aligned}
&\frac{1}{2}\sum_{\mathbf{N}} f(\mathbf{N}_{i,j})|N_X - N|^{s'_0}N_i^{s'_1}N_j^{s'_2}\sqrt{N_i}\sqrt{N_j+1}|N_X - 1 - N|^{s_0}(N_i - 1)^{s_1}(N_j+1)^{s_2}\langle N_i, N_j, \mathbf{N}_{i,j}|\rho(\tau)|N_i, N_j, \mathbf{N}_{i,j}\rangle \\
&\le \frac{1}{2}\mathrm{tr}\left[f(\hat{\mathbf{n}})(|\hat{n}_X - N| + 1)^{s_0+s'_0}\hat{n}_i^{s'_1}\hat{n}_j^{s'_2}(\hat{n}_i + \hat{n}_j)(\hat{n}_i - 1)^{s_1}(\hat{n}_j + 1)^{s_2}\rho(\tau)\right] \\
&\le \frac{1}{2}\mathrm{tr}\left[f(\hat{\mathbf{n}})(\hat{n}'_X + 1)^{s_0+s'_0}\hat{n}_i^{s_1+s'_1}(\hat{n}_j + 1)^{s_2+s'_2}(\hat{n}_i + \hat{n}_j)\rho(\tau)\right].
\end{aligned} \tag{182}$$

In the same way, we obtain

$$\begin{aligned}
&\frac{1}{2}\sum_{\mathbf{N}} f(\mathbf{N}_{i,j})|N_X - N|^{s'_0}N_i^{s'_1}N_j^{s'_2}\sqrt{N_i}\sqrt{N_j+1}|N_X - 1 - N|^{s_0}(N_i - 1)^{s_1}(N_j+1)^{s_2} \\
&\qquad \times \langle N_i - 1, N_j + 1, \mathbf{N}_{i,j}|\rho(\tau)|N_i - 1, N_j + 1, \mathbf{N}_{i,j}\rangle \\
&= \frac{1}{2}\sum_{\mathbf{N}} f(\mathbf{N}_{i,j})|N_X - N + 1|^{s'_0}(N_i + 1)^{s'_1}(N_j - 1)^{s'_2}\sqrt{N_i+1}\sqrt{N_j}|N_X - N|^{s_0}N_i^{s_1}N_j^{s_2}\langle N_i, N_j, \mathbf{N}_{i,j}|\rho(\tau)|N_i, N_j, \mathbf{N}_{i,j}\rangle \\
&= \frac{1}{2}\mathrm{tr}\left[f(\hat{\mathbf{n}})|\hat{n}_X - N + 1|^{s'_0}(\hat{n}_i + 1)^{s'_1}(\hat{n}_j - 1)^{s'_2}(\hat{n}_i + \hat{n}_j)|\hat{n}_X - N|^{s_0}\hat{n}_i^{s_1}\hat{n}_j^{s_2}\rho(\tau)\right] \\
&\le \frac{1}{2}\mathrm{tr}\left[f(\hat{\mathbf{n}})(\hat{n}'_X + 1)^{s_0+s'_0}(\hat{n}_i + 1)^{s_1+s'_1}\hat{n}_j^{s_2+s'_2}(\hat{n}_i + \hat{n}_j)\rho(\tau)\right].
\end{aligned} \tag{183}$$

By combining the inequalities (182) and (183), we obtain the desired inequality (177). This completes the proof. □

[**End of Proof of Lemma 11**]

By using Lemma 11, we can upper-bound the quantities (176) in the same way of (121):

$$\begin{aligned}\left|\mathrm{tr}\left(\mathcal{D}_{<k}^{(m)} b_{X,i} b_j^{\dagger} \mathcal{D}_{>k}^{(m)} \rho(\tau)\right)\right| &\le \mathrm{tr}\left(\mathcal{D}_{<k}^{(m+1)}(\hat{n}_i+\hat{n}_j)\mathcal{D}_{>k}^{(m+1)}\rho(\tau)\right), \\ \left|\mathrm{tr}\left(\mathcal{D}_{<k}^{(m)} (b_{X,i})^{\dagger} b_j \mathcal{D}_{>k}^{(m)} \rho(\tau)\right)\right| &\le \mathrm{tr}\left(\mathcal{D}_{<k}^{(m+1)}(\hat{n}_i+\hat{n}_j)\mathcal{D}_{>k}^{(m+1)}\rho(\tau)\right),\end{aligned} \tag{184}$$

which yields the same inequality as (122).

For general $m_k \ge 1$, we apply the same analyses because of

$$[H, \mathcal{D}^{m_k}(\hat{n}'_X)] = [H_0, \mathcal{D}^{m_k}(\hat{n}_X)] \quad \text{for} \quad m_k \ge 1, \tag{185}$$

and hence we immediately obtain all the inequalities from (124) and (129). We thus derive the same statement as Proposition 8. As mentioned, based on the statement, we can immediately prove our main inequality (165). This completes the proof. □

D. Lower bound on the moment function

We have derived the upper bound for the sth moment function. The same analyses can be applied to derive a lower bound. We here prove the following corollary:

Corollary 12. *For an arbitrary subset X, the moment function $M_X^{(s)}(\tau)$ is lower-bounded by*

$$M_X^{(s)}(\tau) \ge \left\langle \hat{n}_X - c_{\tau,1}\hat{\mathcal{D}}_X - c_{\tau,2}\right\rangle_{\rho}^{s}, \tag{186}$$

where $\tau \le 1/(4\bar{J}\gamma)$ and $\left\langle \hat{n}_X - c_{\tau,1}\hat{\mathcal{D}}_X - c_{\tau,2}\right\rangle_{\rho} \ge 0$ is assumed.

1. Proof of Corollary 12

For the proof, it is enough to derive the first order moment because of

$$\mathrm{tr}(\rho(\tau)\hat{n}_X^s) \ge [\mathrm{tr}(\rho(\tau)\hat{n}_X)]^s \tag{187}$$

for $s \ge 1$. Here, the inequality (133) is changed to

$$\begin{aligned} M_X^{(s)}(\tau) &\ge M_X^{(s)}(0) - \sum_{m=1}^{\infty} \frac{(2\tau)^m}{m!}\mathrm{tr}\left(\tilde{\mathcal{K}}^m(\hat{n}_X^s)\rho\right) \\ &= 2M_X^{(s)}(0) - \sum_{m=0}^{\infty} \frac{(2\tau)^m}{m!}\mathrm{tr}\left(\tilde{\mathcal{K}}^m(\hat{n}_X^s)\rho\right) \\ &\ge 2M_X^{(s)}(0) - \mathrm{tr}\left[\left(\hat{n}_X + c_{\tau,1}\hat{\mathcal{D}}_X + c_{\tau,2}s\right)^s \rho\right], \end{aligned} \tag{188}$$

where in the last inequality, we use the upper bound (74). By considering $s = 1$, we prove

$$\hat{n}_X(\tau) \succeq 2\hat{n}_X - \left(\hat{n}_X + c_{\tau,1}\hat{\mathcal{D}}_X + c_{\tau,2}\right) = \hat{n}_X - c_{\tau,1}\hat{\mathcal{D}}_X - c_{\tau,2}, \tag{189}$$

which reduces the inequality (187) to

$$\mathrm{tr}\left[\rho\hat{n}_X(\tau)^s\right] \ge \{\mathrm{tr}\left[\rho\hat{n}_X(\tau)\right]\}^s \ge \left\langle \hat{n}_X - c_{\tau,1}\hat{\mathcal{D}}_X - c_{\tau,2}\right\rangle_{\rho}^{s}. \tag{190}$$

This completes the proof. □

Supplementary Note 3. REFINED MOMENT BOUNDS AFTER SHORT-TIME EVOLUTION

A. Strategy for refined bounds

We here show a rough description of refining the obtained bound in Subtheorem 1. We have shown a similar explanation in the Method section of the main manuscript (see the subsection entitled "Outline of the proof for Result 1").

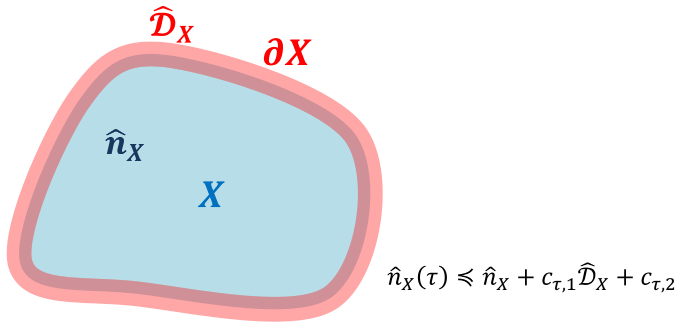

Supplementary Figure 3. In the upper bound (191) on the moment function, the operator $\hat{n}_X(\tau)$ is bounded from above by $\left(\hat{n}_X + c_{\tau,1}\hat{\mathcal{D}}_X + c_{\tau,2}\right)$. Here, the operator $\hat{\mathcal{D}}_X$ is roughly approximated on the surface region ∂X. Hence, we expect that $\hat{n}_X + c_{\tau,1}\hat{\mathcal{D}}_X + c_{\tau,2} \approx \hat{n}_X\left(1 + |\partial X|/|X|\right)$. To make the expectation rigorous, we need to consider the possibility that most of the bosons concentrate on ∂X, which spoils the above discussions. However, by carefully choosing the subsets $X[r]$ in (193), we can obtain a refined upper bound as in (194).

According to Subtheorem 1, we have

$$M_X^{(1)}(\tau) = \mathrm{tr}\left[\hat{n}_X^s \rho(\tau)\right] \le \mathrm{tr}\left[\left(\hat{n}_X + c_{\tau,1}\hat{\mathcal{D}}_X + c_{\tau,2}\right)\rho\right], \quad \hat{\mathcal{D}}_X = \sum_{i\in\partial X}\sum_{j\in\Lambda} e^{-d_{i,j}}\hat{n}_j, \tag{191}$$

where we consider $s = 1$ for simplicity. The operator $\hat{\mathcal{D}}_X$ is approximately supported around the surface region ∂X. Therefore, we expect that the contribution from $\hat{\mathcal{D}}_X$ is ignorable in comparison with that from $\hat{n}_X$ as the size of X increases (see Fig. 3). Note that we have $|\partial X|/|X| \to 0$ as $\mathrm{diam}(X) \to \infty$. However, we have a possibility that the bosons concentrate on the surface region, which may imply

$$\mathrm{tr}\left[\left(\hat{n}_X + c_{\tau,1}\hat{\mathcal{D}}_X\right)\rho\right] = [1+\mathcal{O}(1)]\mathrm{tr}\left(\hat{n}_X\rho\right) \longrightarrow M_X^{(1)}(\tau) = [1+\mathcal{O}(1)]M_X^{(1)}(0). \tag{192}$$

In the above case, by connecting the short-time evolution, the moment function $M_X^{(1)}(\tau)$ exponentially increases as $e^{\mathcal{O}(t/\tau)}$ for general t, which leads to a meaningless upper bound.

In the following, we start from the following trivial inequality:

$$M_X^{(1)}(\tau) \le M_{X[r]}^{(1)}(\tau) = \mathrm{tr}\left[\left(\hat{n}_{X[r]} + c_{\tau,1}\hat{\mathcal{D}}_{X[r]} + c_{\tau,2}\right)\rho\right]. \tag{193}$$

We then prove that by appropriately choosing r in (193) ($0 \le r \le \ell$), we can upper-bound $M_X^{(1)}(\tau)$ in the form of

$$M_X^{(1)}(\tau) \le \mathrm{tr}\left[\left(\hat{n}_{X[\ell]} + c_{\tau,2}s + e^{-\Omega(\ell)}\right)\rho\right] \tag{194}$$

with ℓ a control parameter. In Sec. Supplementary Note 3 C, we consider the case of $s = 1$, and afterward, we extend the result to general s in Sec. Supplementary Note 3 E.

B. Convenient lemmas

Before going to the proof, we first derive several convenient lemmas for our analyses. The main statement starts from Sec. Supplementary Note 3 C and readers can skip this subsection.

Let $\{\alpha_j\}_{j=0}^{\infty}$ be an arbitrary positive sequence. For fixed $\{N_j\}_{j=1}^{\tilde{p}}$ ($N_j \in \mathbb{N}$, $j \in [1:\tilde{p}]$), we here define $\tilde{N}_p$ as follows:

$$\tilde{N}_p := \sum_{j=1}^{\tilde{p}} \alpha_{|p-j|} N_j, \tag{195}$$

where $\{\alpha_j\}_{j=0}^{\infty}$ are arbitrary positive numbers. In practical applications, we consider $\{\alpha_j\}_{j=0}^{\infty}$ that α_j decays exponentially as j increases. We then consider the following quantity ($1 \le p \le \tilde{p}$):

$$\tilde{N}_p + N_{1:p}, \quad N_{1:p} = \sum_{j=1}^{p} N_j. \tag{196}$$

We, in the following, aim to estimate the minimization of

$$\min_{p\in[1:\tilde{p}]}\left(\tilde{N}_p + N_{1:p}\right). \tag{197}$$

We can prove the following statement, which claims that the above quantity is upper-bounded by $(1+e^{-\Omega(\tilde{p})})N_{1:\tilde{p}}$:

Lemma 13. *The quantity* (197) *is upper-bounded as follows:*

$$\min_{p\in[1:\tilde{p}]} \left(\tilde{N}_p + N_{1:p}\right) \le \left[1 + \bar{\alpha}_\infty \left(1 + \frac{1}{\bar{\alpha}_\infty + \chi_\alpha}\right)^{-\tilde{p}+1}\right] N_{1:\tilde{p}}, \tag{198}$$

where $\bar{\alpha}_\infty$ *and* χ_α *are defined by Eqs.* (211) *and* (213), *i.e.,*

$$\bar{\alpha}_\infty := \sum_{j=-\infty}^{\infty} \alpha_{|j|}, \quad \chi_\alpha := \max_{j\in[0,\infty]} \left(\sum_{p=1}^{\infty} \alpha_{p+j}/\alpha_j\right). \tag{199}$$

Remark. The parameters $\bar{\alpha}_\infty$ and χ_α may be unbounded. In particular, when α_j decays polynomially with j, the parameter χ_α is infinitely large. If α_j decays exponentially with j, both of the parameters $\bar{\alpha}_\infty$ and χ_α are $\mathcal{O}(1)$ constants. For example, for $\alpha_j = 0$ for $\forall j \ge 1$, we can trivially obtain $\bar{\alpha}_\infty = \alpha_0$ and $\chi_\alpha = 0$.

1. *Proof of Lemma 13*

For proof, we can restrict ourselves to the case of

$$\min_{p\in[1:\tilde{p}]} \left(\tilde{N}_p + N_{1:p}\right) \ge N_{1:\tilde{p}}. \tag{200}$$

Note that in the case where the above inequality does not hold, the main inequality (198) trivially holds. From the inequality (200), we obtain

$$\tilde{N}_p + N_{1:p} \ge N_{1:\tilde{p}} \longrightarrow \tilde{N}_p \ge N_{p+1:\tilde{p}}. \tag{201}$$

The key tool to analyze the condition (201) is the following inequality (see below for the proof):

$$\begin{aligned} &\tilde{N}_{p+1:\tilde{p}} \le \bar{\alpha}_\infty N_{p+1:\tilde{p}} + \chi_\alpha \tilde{N}_p \quad \text{for} \quad \forall p, \\ &\tilde{N}_{p_1:p_2} := \sum_{p\in[p_1,p_2]} \tilde{N}_p. \end{aligned} \tag{202}$$

By using the above inequality, the condition (201) reduces to

$$\begin{aligned} &\tilde{N}_p \ge N_{p+1:\tilde{p}} \ge \frac{1}{\bar{\alpha}_\infty} \left(\tilde{N}_{p+1:\tilde{p}} - \chi_\alpha \tilde{N}_p\right) \\ &\longrightarrow \tilde{N}_p \ge \frac{1}{\bar{\alpha}_\infty + \chi_\alpha} \tilde{N}_{p+1:\tilde{p}} =: \frac{1}{\bar{\alpha}} \tilde{N}_{p+1:\tilde{p}} \quad \text{for} \quad \forall p, \end{aligned} \tag{203}$$

where we define $\bar{\alpha} := \bar{\alpha}_\infty + \chi_\alpha$.

By using the inequality (203), we first reduce the quantity $\tilde{N}_{1:\tilde{p}}$ to

$$\tilde{N}_{1:\tilde{p}} = \tilde{N}_1 + \tilde{N}_{2:\tilde{p}} \ge \left(1 + \frac{1}{\bar{\alpha}}\right) \tilde{N}_{2:\tilde{p}}. \tag{204}$$

In the same way, we have

$$\tilde{N}_{1:\tilde{p}} \ge \left(1 + \frac{1}{\bar{\alpha}}\right) \tilde{N}_{2:\tilde{p}} \ge \left(1 + \frac{1}{\bar{\alpha}}\right) \left(\tilde{N}_2 + \tilde{N}_{3:\tilde{p}}\right) \ge \left(1 + \frac{1}{\bar{\alpha}}\right)^2 \tilde{N}_{3:\tilde{p}}. \tag{205}$$

By repeating the process, we obtain

$$\tilde{N}_{1:\tilde{p}} \ge \left(1 + \frac{1}{\bar{\alpha}}\right)^{\tilde{p}-1} \tilde{N}_{\tilde{p}:\tilde{p}} = \left(1 + \frac{1}{\bar{\alpha}}\right)^{\tilde{p}-1} \tilde{N}_{\tilde{p}}. \tag{206}$$

Therefore, we obtain

$$\tilde{N}_{\tilde{p}} \le \left(1 + \frac{1}{\bar{\alpha}}\right)^{-\tilde{p}+1} \tilde{N}_{1:\tilde{p}} \le \bar{\alpha}_\infty \left(1 + \frac{1}{\bar{\alpha}}\right)^{-\tilde{p}+1} N_{1:\tilde{p}}, \tag{207}$$

where we use the definition (211) below to obtain $\tilde{N}_{1:\tilde{p}} \le \bar{\alpha}_\infty N_{1:\tilde{p}}$ as follows:

$$\tilde{N}_{1:\tilde{p}} = \sum_{p'=1}^{\tilde{p}} \tilde{N}_{p'} = \sum_{p'=1}^{\tilde{p}} \sum_{j=1}^{\tilde{p}} \alpha_{|p'-j|} N_j \le \sum_{p'=-\infty}^{\infty} \alpha_{|p'|} \sum_{j=1}^{\tilde{p}} N_j = \bar{\alpha}_\infty N_{1:\tilde{p}}. \tag{208}$$

We thus arrive at the inequality of

$$N_{1:\tilde{p}} + \tilde{N}_{\tilde{p}} \le \left[1 + \bar{\alpha}_\infty \left(1 + \frac{1}{\bar{\alpha}}\right)^{-\tilde{p}+1}\right] N_{1:\tilde{p}}. \tag{209}$$

The above upper bound is applied to $\min_{p\in[1:\tilde{p}]} \left(N_{1:p} + \tilde{N}_p\right)$, which yields the desired inequality (198) by replacing $\bar{\alpha} \to \bar{\alpha}_\infty + \chi_\alpha$. This completes the proof. □

[**Proof of the inequality** (202)]
We here consider the quantity of

$$\tilde{N}_{p+1:\tilde{p}} = \sum_{p'=p+1}^{\tilde{p}} \tilde{N}_{p'} = \sum_{p'=p+1}^{\tilde{p}} \sum_{j=1}^{\tilde{p}} \alpha_{|p'-j|} N_j. \tag{210}$$

By defining $\bar{\alpha}_\infty$ as

$$\bar{\alpha}_\infty := \sum_{j=-\infty}^{\infty} \alpha_{|j|}, \tag{211}$$

we first obtain

$$\tilde{N}_{p+1:\tilde{p}} \le \bar{\alpha}_\infty N_{p+1:\tilde{p}} + \sum_{j=1}^{p} \sum_{p'=p+1}^{\tilde{p}} \alpha_{|p'-j|} N_j = \bar{\alpha}_\infty N_{p+1:\tilde{p}} + \sum_{j=1}^{p} \sum_{p'=1}^{\tilde{p}-p} \alpha_{p'+p-j} N_j. \tag{212}$$

We here introduce the parameter χ_α as

$$\chi_\alpha = \max_{j\in[0,\infty]} \left(\sum_{p=1}^{\infty} \alpha_{p+j}/\alpha_j \right), \tag{213}$$

which trivially satisfies

$$\sum_{p'=1}^{\tilde{p}-p} \alpha_{p'+p-j} \le \sum_{p'=1}^{\infty} \alpha_{p'+p-j} \le \chi_\alpha \alpha_{p-j} \quad \text{for} \quad \forall j \in [0,p]. \tag{214}$$

We then obtain

$$\sum_{j=1}^{p} \sum_{p'=1}^{\tilde{p}-p} \alpha_{p'+p-j} N_j \le \sum_{j=1}^{p} \chi_\alpha \alpha_{p-j} N_j \le \chi_\alpha \sum_{j=1}^{\tilde{p}} \alpha_{|p-j|} N_j = \chi_\alpha \tilde{N}_p, \tag{215}$$

where we use the definition (195) in the last equation. By combining the inequalities (212) and (215), we arrive at the main inequality (202). This completes the proof. □

———————————————————— [**End of Proof of Lemma 13**]

Conversely, we also consider the maximization of

$$\max_{p\in[1:\tilde{p}]} \left(-\tilde{N}_p + N_{1:p}\right). \tag{216}$$

We can prove the following similar statement:

Lemma 14. *The quantity* (216) *is lower-bounded as follows:*

$$\max_{p\in[1:\tilde{p}]} \left(-\tilde{N}_p + N_{1:p}\right) \ge -\bar{\alpha}_\infty \left(1 + \frac{1}{\bar{\alpha}_\infty + \chi_\alpha}\right)^{-\tilde{p}+1} N_{1:\tilde{p}}, \tag{217}$$

where $\bar{\alpha}_\infty$ *and* χ_α *have been defined in Eqs.* (211) *and* (213)*.*

2. Proof of Lemma 14

The proof is almost the same as the one for Lemma 13. We can restrict ourselves to the case of

$$\max_{p\in[1:\tilde{p}]} \left(-\tilde{N}_p + N_{1:p}\right) \le 0. \tag{218}$$

Otherwise, the inequality (217) trivially holds. Then, we obtain

$$-\tilde{N}_p + N_{1:p} \le 0 \longrightarrow \tilde{N}_p \ge N_{1:p}. \tag{219}$$

We can obtain a similar inequality to the inequality (202):

$$\tilde{N}_{1:p-1} \le \bar{\alpha}_\infty N_{1:p} + \chi_\alpha \tilde{N}_p \quad \text{for} \quad \forall p, \tag{220}$$

where we show the proof below. By using (220), we can reduce the inequality (219) to

$$\tilde{N}_p \ge N_{1:p} \ge \frac{1}{\bar{\alpha}_\infty}\left(\tilde{N}_{1:p-1} - \chi_\alpha \tilde{N}_p\right) \longrightarrow \tilde{N}_p \ge \frac{1}{\bar{\alpha}_\infty + \chi_\alpha}\tilde{N}_{1:p-1} \tag{221}$$

By using the inequality (221), we first reduce the quantity $\tilde{N}_{1:\tilde{p}}$ to

$$\tilde{N}_{1:\tilde{p}} = \tilde{N}_{\tilde{p}} + \tilde{N}_{1:\tilde{p}-1} \ge \left(1 + \frac{1}{\bar{\alpha}}\right)\tilde{N}_{1:\tilde{p}-1}, \quad \bar{\alpha} := \bar{\alpha}_\infty + \chi_\alpha. \tag{222}$$

In the same way, we have

$$\tilde{N}_{1:\tilde{p}} \ge \left(1 + \frac{1}{\bar{\alpha}}\right)\tilde{N}_{1:\tilde{p}-1} \ge \left(1 + \frac{1}{\bar{\alpha}}\right)\left(\tilde{N}_{\tilde{p}-1} + \tilde{N}_{1:\tilde{p}-2}\right) \ge \left(1 + \frac{1}{\bar{\alpha}}\right)^2 \tilde{N}_{1:\tilde{p}-2}. \tag{223}$$

By repeating the process, we obtain

$$\tilde{N}_{1:\tilde{p}} \ge \left(1 + \frac{1}{\bar{\alpha}}\right)^{\tilde{p}-1}\tilde{N}_{1:1} = \left(1 + \frac{1}{\bar{\alpha}}\right)^{\tilde{p}-1}\tilde{N}_1. \tag{224}$$

Therefore, we arrive at the inequality of

$$\tilde{N}_1 \le \left(1 + \frac{1}{\bar{\alpha}}\right)^{-\tilde{p}+1}\tilde{N}_{1:\tilde{p}} \le \bar{\alpha}_\infty \left(1 + \frac{1}{\bar{\alpha}}\right)^{-\tilde{p}+1} N_{1:\tilde{p}}, \tag{225}$$

where the second inequality immediately results from the inequality (208). We thus obtain the inequality of

$$N_{1:1} - \tilde{N}_1 \ge N_1 - \bar{\alpha}_\infty \left(1 + \frac{1}{\bar{\alpha}}\right)^{-\tilde{p}+1} N_{1:\tilde{p}}. \tag{226}$$

The above lower bound is also applied to $\max_{p\in[1:\tilde{p}]}\left(-\tilde{N}_p + N_{1:p}\right)$ under the condition of (218), which yields the desired inequality (217). This completes the proof. □

[**Proof of the inequality** (220)]
The proof is almost the same as for the inequality (202). We here consider

$$\tilde{N}_{1:p-1} = \sum_{p'=1}^{p-1} \tilde{N}_{p'} = \sum_{p'=1}^{p-1}\sum_{j=1}^{\tilde{p}} \alpha_{|p'-j|} N_j. \tag{227}$$

From the definition of $\bar{\alpha}_\infty := \sum_{j=-\infty}^{\infty} \alpha_{|j|}$, we first obtain

$$\tilde{N}_{1:p-1} \le \bar{\alpha}_\infty N_{1:p} + \sum_{j=p+1}^{\tilde{p}}\sum_{p'=1}^{p-1} \alpha_{|p'-j|} N_j = \bar{\alpha}_\infty N_{1:p} + \sum_{j=p+1}^{\tilde{p}}\sum_{p'=1}^{p-1} \alpha_{j-p'} N_j. \tag{228}$$

By using the parameter χ_α in Eq. (213), i.e.,

$$\chi_\alpha = \max_{j\in[0,\infty]} \left(\sum_{p=1}^{\infty} \alpha_{p+j}/\alpha_j\right),$$

we have

$$\sum_{p'=1}^{p-1} \alpha_{j-p'} = \sum_{p'=1}^{p-1} \alpha_{j-p+p-p'} \le \sum_{p''=1}^{\infty} \alpha_{j-p+p''} \le \chi_\alpha \alpha_{j-p} \quad \text{for} \quad j \in [p+1, \tilde{p}]. \tag{229}$$

We then obtain

$$\sum_{j=p+1}^{\tilde{p}} \sum_{p'=1}^{p-1} \alpha_{j-p'} N_j \le \sum_{j=p+1}^{\tilde{p}} \chi_\alpha \alpha_{j-p} N_j \le \chi_\alpha \sum_{j=1}^{\tilde{p}} \alpha_{|p-j|} N_j = \chi_\alpha \tilde{N}_p. \tag{230}$$

By combining the inequalities (228) and (230), we arrive at the main inequality (220). This completes the proof. □

————————————————————————[**End of Proof of Lemma 14**]

Finally, in proving Proposition 22, we need to estimate the minimization of

$$\min_{p\in[1:\tilde{p}]} \frac{\tilde{N}_p}{N_{1:p}}, \tag{231}$$

where $\tilde{N}_p := \sum_{j=1}^{\tilde{p}} \alpha_{|p-j|} N_j$. By using similar analyses to the proof of Lemma 14, we can prove the following statement:

Lemma 15. *If there does not exist a parameter $p \in [1 : \tilde{p}]$ such that*

$$\frac{\tilde{N}_p}{N_{1:p}} \le \frac{1}{\Phi}, \tag{232}$$

the quantity $\tilde{N}_1$ is upper-bounded by

$$\tilde{N}_1 \le \left(1 + \frac{1}{\Phi \bar{\alpha}_\infty + \chi_\alpha}\right)^{-\tilde{p}+1} \bar{\alpha}_\infty N_{1:\tilde{p}}, \tag{233}$$

where $\bar{\alpha}_\infty$ and χ_α are defined by Eqs. (211) and (213).

Remark. From the lemma, we can ensure that either of the following statements is satisfied:

$$\min_{p\in[1:\tilde{p}]} \frac{\tilde{N}_p}{N_{1:p}} \le \frac{1}{\Phi} \quad \text{or} \quad \tilde{N}_1 \le e^{-\Omega(\tilde{p})} N_{1:\tilde{p}}. \tag{234}$$

The lemma plays a central role in proving Lemma 24 below (see Case 2 in the proof of Lemma 24, Sec. Supplementary Note 5 C 1).

3. *Proof of Lemma 15*

We aim to estimate the upper bound of $\tilde{N}_1$ under the condition of

$$\frac{\tilde{N}_p}{N_{1:p}} \ge \frac{1}{\Phi} \quad \text{for} \quad \forall p \in [1, \tilde{p}]. \tag{235}$$

By using the inequality (220), we obtain

$$\tilde{N}_p \ge \frac{N_{1:p}}{\Phi} \ge \frac{1}{\Phi} \cdot \frac{\tilde{N}_{1:p-1} - \chi_\alpha \tilde{N}_p}{\bar{\alpha}_\infty} \longrightarrow \tilde{N}_p \ge \frac{\tilde{N}_{1:p-1}}{\Phi \bar{\alpha}_\infty + \chi_\alpha}, \tag{236}$$

which yields

$$\tilde{N}_{1:p} = \tilde{N}_{1:p-1} + \tilde{N}_p \ge \left(1 + \frac{1}{\Phi \bar{\alpha}_\infty + \chi_\alpha}\right) \tilde{N}_{1:p-1}. \tag{237}$$

By repeatedly using the above inequality, we reach the inequality of

$$\tilde{N}_{1:\tilde{p}} \ge \left(1 + \frac{1}{\Phi \bar{\alpha}_\infty + \chi_\alpha}\right)^{\tilde{p}-1} \tilde{N}_1. \tag{238}$$

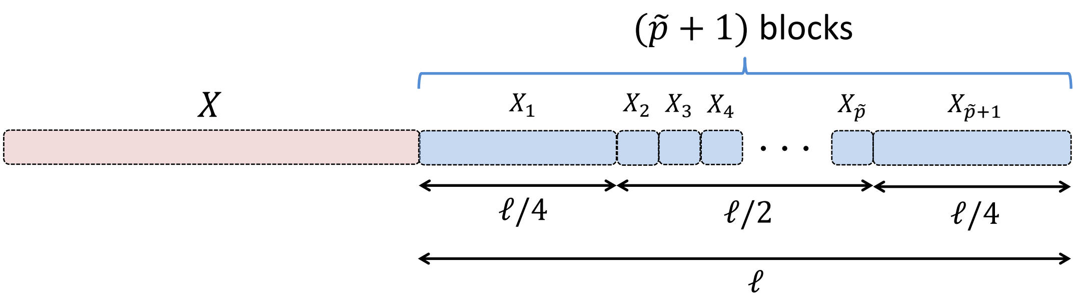

Supplementary Figure 4. We decompose the subset $X[\ell] \setminus X$ to $\tilde{p}+1$ pieces. In the picture, a one-dimensional case is considered. We define the extended subset of X from X_1 to X_p as $X_{0:p} := X \cup X_1 \cup \cdots \cup X_p$. Then, we upper-bound the moment function $M_X^{(1)}(\tau)$ by using $M_{X_{0:p}}^{(1)}(\tau)$ with an appropriate choice of p. In the choice, we need to suppress the contribution from the surface region (i.e., the term $\hat{\mathcal{D}}_{X_{0:p}}$).

Hence, by using $\tilde{N}_{1:\tilde{p}} \le \bar{\alpha}_\infty N_{1:\tilde{p}}$ from the inequality (208), we obtain the main inequality:

$$\tilde{N}_1 \le \left(1 + \frac{1}{\Phi\bar{\alpha}_\infty + \chi_\alpha}\right)^{-\tilde{p}+1} \bar{\alpha}_\infty N_{1:\tilde{p}}. \tag{239}$$

This completes the proof. □

———————————————————————[**End of Proof of Lemma 15**]

C. Moment upper bound: 1st order

We first consider the 1st order moment, i.e.,

$$M_X^{(1)}(\tau) = \langle \hat{n}_X(\tau) \rangle_\rho \le \left\langle \hat{n}_X + c_{\tau,1}\hat{\mathcal{D}}_X + c_{\tau,2} \right\rangle_\rho, \tag{240}$$

where we use Subtheorem 1 in the inequality. For the above quantity, we can prove the following proposition in the form of the operator inequality, which yields the desired bound as (194):

Proposition 16. *For an arbitrary time of $\tau \le 1/(4\gamma\bar{J})$, we obtain the following operator inequality:*

$$\hat{n}_X(\tau) \preceq \left(1 + 5c_{\tau,1}\lambda_{1/2}e^{-f_\tau \ell}\right)\hat{n}_{X[\ell]} + c_{\tau,1}e^{-3\ell/16}\hat{\mathscr{D}}_{X[\ell]} + c_{\tau,2}, \tag{241}$$

where we can choose the length ℓ arbitrarily, and the operator $\hat{\mathscr{D}}_{X[\ell]}$ and the constant f_τ are defined by using parameter λ_c in (12) as follows:

$$\hat{\mathscr{D}}_{X[\ell]} := \sum_{j \in X[\ell]^{\rm c}} e^{-3d_{j,X[\ell]}/4}\hat{n}_j \tag{242}$$

and

$$f_\tau := \frac{1}{2}\log\left(1 + \frac{1}{5c_{\tau,1}\lambda_{1/2} + 2}\right). \tag{243}$$

D. Proof of Proposition 16

We first define the subsets $\{X_p\}_{p=1}^{\tilde{p}+1}$ as follows (see Fig. 4):

$$\begin{aligned} &X_0 := X, \quad X_1 := X[\ell/4] \setminus X, \\ &X_p := X[\ell/4 + p - 1] \setminus X[\ell/4 + p - 2] \quad (2 \le p \le \tilde{p}), \\ &X_{\tilde{p}+1} := X[\ell] \setminus X[3\ell/4], \end{aligned} \tag{244}$$

where $\tilde{p} = \ell/2 + 1$. We then consider the first order moment as

$$M_{X_{0:p}}^{(1)}(\tau) = {\rm tr}\left[\hat{n}_{X_{0:p}}(\tau)\rho\right], \quad X_{0:p} := \bigcup_{j=0}^{p} X_j \tag{245}$$

for an arbitrary quantum state ρ. Note that because of $X \subseteq X_{0:p}$ (i.e., $\hat{n}_X \preceq \hat{n}_{X_{0:p}}$) we trivially obtain

$$M_X^{(1)}(\tau) \le M_{X_{0:p}}^{(1)}(\tau) \tag{246}$$

for arbitrary $p \ge 0$.

From the inequality (240), we obtain the upper bound of

$$\begin{aligned} M_{X_{0:p}}^{(1)}(\tau) &\le \mathrm{tr}\left[\left(\hat{n}_{X_{0:p}} + c_{\tau,1}\hat{\mathcal{D}}_{X_{0:p}} + c_{\tau,2}\right)\rho\right], \quad \hat{\mathcal{D}}_{X_{0:p}} = \sum_{i\in\partial(X_{0:p})}\sum_{j\in\Lambda} e^{-d_{i,j}}\hat{n}_j, \\ &= \left\langle \hat{n}_{X_{0:p}}\right\rangle_\rho + c_{\tau,1}\left\langle \hat{\mathcal{D}}_{X_{0:p}}\right\rangle_\rho + c_{\tau,2} \\ &= N_{X_{0:p}}^{(\rho)} + c_{\tau,1}\tilde{N}_{X_{0:p}}^{(\rho)} + c_{\tau,2}, \quad \tilde{N}_{X_{0:p}}^{(\rho)} := \sum_{i\in\partial(X_{0:p})}\sum_{j\in\Lambda} e^{-d_{i,j}} N_j^{(\rho)}, \end{aligned} \tag{247}$$

where we denote $\langle \hat{n}_i\rangle_\rho = N_i^{(\rho)}$ for $\forall i \in \Lambda$. In the following, we aim to estimate the best choice of p that minimizes the moment as

$$\min_{p\in[1,\tilde{p}]}\left(N_{X_{0:p}}^{(\rho)} + c_{\tau,1}\tilde{N}_{X_{0:p}}^{(\rho)}\right), \tag{248}$$

from which we obtain an optimal upper bound for $M_X^{(1)}(\tau)$ because of the inequality (246).

For the estimation of the RHS of the inequality (247), we reduce the problem (248) to the form for which Lemma 13 can be applied. For this purpose, we first need to upper-bound $\tilde{N}_{X_{0:p}}$ to make it a tractable form. We prove the following lemma (see Sec. Supplementary Note 3 D 1 below for the proof):

Lemma 17. *By using the parameter λ_c in Eq. (12), we obtain the upper bound of*

$$\begin{aligned} &\tilde{N}_{X_{0:p}}^{(\rho)} \le \lambda_{1/2}\sum_{j=1}^{\tilde{p}} e^{-|p-j|/2}M_j + \delta N_{X,\ell}^{(\rho)} \quad (1\le p\le \tilde{p}), \\ &M_j = \begin{cases} N_{X_j}^{(\rho)} & \textit{for} \quad 1\le j\le \tilde{p}-1, \\ N_{X_{\tilde{p}}}^{(\rho)} + N_{X_{\tilde{p}+1}}^{(\rho)} & \textit{for} \quad j=\tilde{p}, \end{cases} \end{aligned} \tag{249}$$

with

$$\delta N_{X,\ell}^{(\rho)} := \lambda_{1/4}e^{-3\ell/16}\left(\sum_{j\in X} N_j^{(\rho)} + \sum_{j\in X[\ell]^{\mathrm{c}}} e^{-3d_{j,X[\ell]}/4}N_j^{(\rho)}\right). \tag{250}$$

By applying Lemma 17 to Eq. (248), we reduce the optimization problem to

$$\min_{p\in[1,\tilde{p}]}\left(N_{X_{0:p}}^{(\rho)} + c_{\tau,1}\tilde{N}_{X_{0:p}}^{(\rho)}\right) \le N_X^{(\rho)} + c_{\tau,1}\delta N_{X,\ell}^{(\rho)} + \min_{p\in[1,\tilde{p}]}\left(M_{1:p} + c_{\tau,1}\lambda_{1/2}\sum_{j=1}^{\tilde{p}} e^{-|p-j|/2}M_j\right), \tag{251}$$

where $M_{1:p} = \sum_{j=1}^{p} M_j$ and we use the definition of $\{M_j\}_{j=1}^{\tilde{p}}$ to obtain

$$N_{X_{0:p}}^{(\rho)} = N_X^{(\rho)} + (M_1 + M_2 + \cdots + M_p) = N_X^{(\rho)} + M_{1:p} \quad \text{for} \quad p \le \tilde{p}-1.$$

To solve the minimization problem in (251), we utilize Lemma 13, where α_j corresponds to $\alpha_j = c_{\tau,1}\lambda_{1/2}e^{-j/2}$. The parameters $\bar{\alpha}_\infty$ and χ_α are estimated as follows:

$$\bar{\alpha}_\infty = c_{\tau,1}\lambda_{1/2}\sum_{j=-\infty}^{\infty} e^{-|j|/2} = \left(\frac{2}{1-e^{-1/2}} - 1\right)c_{\tau,1}\lambda_{1/2} = (4.0829\cdots)c_{\tau,1}\lambda_{1/2} \le 5c_{\tau,1}\lambda_{1/2}, \tag{252}$$

and

$$\chi_\alpha = \sum_{p=1}^{\infty} e^{-p/2} = \frac{e^{-1/2}}{1-e^{-1/2}} = (1.5414\cdots) < 2. \tag{253}$$

Thus, the inequality (198) yields

$$\begin{aligned} \min_{p\in[1,\tilde{p}]}\left(M_{1:p} + c_{\tau,1}\lambda_{1/2}\sum_{j=1}^{\tilde{p}} e^{-|p-j|/2}M_j\right) &\le \left[1 + 5c_{\tau,1}\lambda_{1/2}\left(1 + \frac{1}{5c_{\tau,1}\lambda_{1/2}+2}\right)^{-\tilde{p}+1}\right]M_{1:\tilde{p}} \\ &= \left[1 + 5c_{\tau,1}\lambda_{1/2}\left(1 + \frac{1}{5c_{\tau,1}\lambda_{1/2}+2}\right)^{-\ell/2}\right](N_{X[\ell]}^{(\rho)} - N_X^{(\rho)}), \end{aligned} \tag{254}$$

where we have used $\tilde{p} = \ell/2 + 1$ and $M_{1:\tilde{p}} = N^{(\rho)}_{X[\ell]} - N^{(\rho)}_{X}$. By combining the inequalities (251) and (254) with Eq. (250), we obtain

$$\min_{p\in[1,\tilde{p}]} \left(N^{(\rho)}_{X_{0:p}} + c_{\tau,1}\tilde{N}^{(\rho)}_{X_{0:p}}\right)$$
$$\leq \left[1 + 5c_{\tau,1}\lambda_{1/2}\left(1 + \frac{1}{5c_{\tau,1}\lambda_{1/2}+2}\right)^{-\ell/2}\right](N^{(\rho)}_{X[\ell]} - N^{(\rho)}_{X}) + N^{(\rho)}_{X} + c_{\tau,1}\lambda_{1/4}e^{-3\ell/16}\left(N^{(\rho)}_{X} + \sum_{j\in X[\ell]^{\mathrm{c}}} e^{-3d_{j,X[\ell]}/4}N^{(\rho)}_{j}\right)$$
$$\leq \left[1 + 5c_{\tau,1}\lambda_{1/2}\left(1 + \frac{1}{5c_{\tau,1}\lambda_{1/2}+2}\right)^{-\ell/2}\right]N^{(\rho)}_{X[\ell]} + c_{\tau,1}\lambda_{1/4}e^{-3\ell/16}\sum_{j\in X[\ell]^{\mathrm{c}}} e^{-3d_{j,X[\ell]}/4}N^{(\rho)}_{j}. \tag{255}$$

By applying the above inequality to (247) with (246), we finally obtain

$$M^{(1)}_{X}(\tau) = \mathrm{tr}\left[\rho\hat{n}_X(\tau)\right] \leq \left(1 + 5c_{\tau,1}\lambda_{1/2}e^{-f_\tau\ell}\right)N^{(\rho)}_{X[\ell]} + c_{\tau,1}\lambda_{1/4}e^{-3\ell/16}\sum_{j\in X[\ell]^{\mathrm{c}}} e^{-3d_{j,X[\ell]}/4}N^{(\rho)}_{j} + c_{\tau,2}$$
$$= \mathrm{tr}\left\{\rho\left[\left(1 + 5c_{\tau,1}\lambda_{1/2}e^{-f_\tau\ell}\right)\hat{n}_{X[\ell]} + c_{\tau,1}e^{-3\ell/16}\hat{\mathscr{D}}_{X[\ell]} + c_{\tau,2}\right]\right\}, \tag{256}$$

where we use the notation of $\hat{\mathscr{D}}_{X[\ell]}$ and f_τ in Eqs. (242) and (243). Because the above inequality holds for an arbitrary quantum state ρ, we prove the desired operator inequality (241). This completes the proof. □

1. *Proof of Lemma 17*

For arbitrary $i \in \partial(X_{0:p}) \subseteq X_p$ and $j' \in X_j$, we have $d_{i,j'} \geq d_{X_p,X_j} \geq |p-j|$ (see Fig. 4), and hence

$$\sum_{i\in\partial(X_{0:p})}\sum_{j'\in X_j} e^{-d_{i,j'}}N^{(\rho)}_{j'} \leq \sum_{j'\in X_j} N^{(\rho)}_{j'} \sum_{i:d_{i,j'}\geq|p-j|} e^{-d_{i,j'}} \leq e^{-|p-j|/2}N^{(\rho)}_{X_j}\sum_{i:d_{i,j'}\geq 0} e^{-d_{i,j'}/2}$$
$$\leq \lambda_{1/2}e^{-|p-j|/2}N^{(\rho)}_{X_j}, \tag{257}$$

where we apply the equation $\sum_{j'\in X_j} N^{(\rho)}_{j'} = N^{(\rho)}_{X_j}$ in the second inequality and use (12) with $c = 1/2$ in the last inequality. We thus reduce $\tilde{N}^{(\rho)}_{X_{0:p}}$ in (247) to the form of

$$\tilde{N}^{(\rho)}_{X_{0:p}} = \sum_{i\in\partial(X_{0:p})}\sum_{j\in\Lambda} e^{-d_{i,j}}N^{(\rho)}_{j} = \sum_{i\in\partial(X_{0:p})}\left(\sum_{j\in X_1} + \sum_{j\in X_2} + \cdots \sum_{j\in X_{\tilde{p}+1}} + \sum_{j\in X^{\mathrm{c}}_{1:\tilde{p}+1}}\right)e^{-d_{i,j}}N^{(\rho)}_{j}$$
$$\leq \lambda_{1/2}\sum_{j=1}^{\tilde{p}-1} e^{-|p-j|/2}N^{(\rho)}_{X_j} + \lambda_{1/2}\left(e^{-|p-\tilde{p}|/2}N^{(\rho)}_{X_{\tilde{p}}} + e^{-|p-\tilde{p}-1|/2}N^{(\rho)}_{X_{\tilde{p}+1}}\right) + \sum_{i\in\partial(X_{0:p})}\sum_{j\in X^{\mathrm{c}}_{1:\tilde{p}+1}} e^{-d_{i,j}}N^{(\rho)}_{j}$$
$$\leq \lambda_{1/2}\sum_{j=1}^{\tilde{p}} e^{-|p-j|/2}M_j + \sum_{i\in\partial(X_{0:p})}\sum_{j\in X^{\mathrm{c}}_{1:\tilde{p}+1}} e^{-d_{i,j}}N^{(\rho)}_{j} \quad (1\leq p\leq\tilde{p}), \tag{258}$$

with $M_j = N^{(\rho)}_{X_j}$ for $1 \leq j \leq \tilde{p}-1$ and $M_{\tilde{p}} = N^{(\rho)}_{X_{\tilde{p}}} + N^{(\rho)}_{X_{\tilde{p}+1}}$, where in the last inequality we use $e^{-|p-\tilde{p}-1|/2} \leq e^{-|p-\tilde{p}|/2}$ for $p \leq \tilde{p}$.

We then aim to upper-bound the second term of the RHS in (258). From the definitions (244), we obtain

$$d_{i,j} \geq \ell/4 \quad \text{for} \quad \forall j\in X, \quad \forall i\in\partial(X_{0:p}) \quad (1\leq p\leq\tilde{p}), \tag{259}$$
$$d_{j,X[\ell]} \leq d_{i,j} - \ell/4 \quad \text{for} \quad \forall j\in X[\ell]^{\mathrm{c}}, \quad \forall i\in\partial(X_{0:p}) \quad (1\leq p\leq\tilde{p}). \tag{260}$$

We recall that $X_{1:\tilde{p}+1} = X[\ell]\setminus X$ (i.e., $X^{\mathrm{c}}_{1:\tilde{p}+1} = X[\ell]^{\mathrm{c}}\cup X$) from the definition (244). Then, from $e^{-d_{i,j}} \leq e^{-d_{i,j}/4-3\ell/16}$ for $j\in X$ and $e^{-d_{i,j}} \leq e^{-d_{i,j}/4-(3/4)(d_{j,X[\ell]}+\ell/4)}$ for $j\in X[\ell]^{\mathrm{c}}$, we can decompose as

$$\sum_{i\in\partial(X_{0:p})}\sum_{j\in X^{\mathrm{c}}_{1:\tilde{p}+1}} e^{-d_{i,j}}N^{(\rho)}_{j} = \sum_{i\in\partial(X_{0:p})}\sum_{j\in X} e^{-d_{i,j}/4-3\ell/16}N^{(\rho)}_{j} + \sum_{i\in\partial(X_{0:p})}\sum_{j\in X[\ell]^{\mathrm{c}}} e^{-d_{i,j}/4-(3/4)(d_{j,X[\ell]}+\ell/4)}N^{(\rho)}_{j}$$
$$\leq \lambda_{1/4}e^{-3\ell/16}\left(\sum_{j\in X} N^{(\rho)}_{j} + \sum_{j\in X[\ell]^{\mathrm{c}}} e^{-3d_{j,X[\ell]}/4}N^{(\rho)}_{j}\right), \tag{261}$$

where we use the inequality (12) to derive

$$\sum_{i\in\partial(X_{0:p})} e^{-d_{i,j}/4} \le \sum_{i\in\Lambda} e^{-d_{i,j}/4} \le \lambda_{1/4} \quad \text{for} \quad \forall j \in \Lambda. \tag{262}$$

By combining the inequalities (258) and (261), we prove the main inequality (249). This completes the proof. □

[**End of Proof of Lemma 17**]

E. Moment upper bound: higher order

1. *Difficulty for the generalization*

In this section, we generalize Proposition 16 to the moment functions with arbitrary orders. Here, we have to consider

$$\mathrm{tr}\left(\hat{n}_X^s \rho(\tau)\right) = \mathrm{tr}\left(\hat{n}_X(\tau)^s \rho\right). \tag{263}$$

The problem is precisely the same as the case for the first order moment if the quantum state is given by the Mott state, i.e., $\rho = |\mathbf{N}\rangle\langle\mathbf{N}|$. In this case, we obtain

$$\mathrm{tr}\left(\hat{n}_X^s \rho(\tau)\right) \le \mathrm{tr}\left[(\hat{n}_X + c_{\tau,1}\hat{\mathcal{D}}_X + c_{\tau_2} s)^s |\mathbf{N}\rangle\langle\mathbf{N}|\right] = \left(\langle\mathbf{N}|\left(\hat{n}_X + c_{\tau,1}\hat{\mathcal{D}}_X + c_{\tau_2} s\right)|\mathbf{N}\rangle\right)^s. \tag{264}$$

Thus, we can apply Proposition 16 to $\langle\mathbf{N}|\left(\hat{n}_X + c_{\tau,1}\hat{\mathcal{D}}_X + c_{\tau_2} s\right)|\mathbf{N}\rangle$, which yields an upper bound in the form of

$$\begin{aligned}\mathrm{tr}\left(\hat{n}_X^s \rho(\tau)\right) &\le \left[\langle\mathbf{N}|\left(1 + 5c_{\tau,1}\lambda_{1/2}e^{-f_\tau \ell}\right)\hat{n}_{X[\ell]} + c_{\tau,1}e^{-3\ell/16}\hat{\mathscr{D}}_{X[\ell]} + c_{\tau,2} s|\mathbf{N}\rangle\right]^s \\ &= \langle\mathbf{N}|\left[\left(1 + 5c_{\tau,1}\lambda_{1/2}e^{-f_\tau \ell}\right)\hat{n}_{X[\ell]} + c_{\tau,1}e^{-3\ell/16}\hat{\mathscr{D}}_{X[\ell]} + c_{\tau,2} s\right]^s |\mathbf{N}\rangle.\end{aligned} \tag{265}$$

However, in general, we have

$$\langle\hat{n}_X^s\rangle_\rho \ge \langle\hat{n}_X\rangle_\rho^s, \tag{266}$$

and hence, we cannot utilize the same analyses to generic quantum states ρ by extending the analysis for 1st order moment.

As a remark, we note that the following discussion cannot be applied to extend the results for the Mott states to an arbitrary state ρ. To see the point, we consider the decomposition of

$$\begin{aligned}\mathrm{tr}(\rho(\tau)\hat{n}_X^s) = \mathrm{tr}(\rho\hat{n}_X(\tau)^s) &= \sum_{\mathbf{N}} \mathrm{tr}\left(\rho|\mathbf{N}\rangle\langle\mathbf{N}|\,[\hat{n}_X(\tau)]^s\right) \\ &\le \sum_{\mathbf{N}} \|\rho|\mathbf{N}\rangle\langle\mathbf{N}|\|_1 \, \|[\hat{n}_X(\tau)]^s\,|\mathbf{N}\rangle\| \\ &= \sum_{\mathbf{N}} \sqrt{\langle\mathbf{N}|\rho^2|\mathbf{N}\rangle}\sqrt{\langle\mathbf{N}|[\hat{n}_X(t)]^{2s}|\mathbf{N}\rangle},\end{aligned} \tag{267}$$

where we use the Hölder inequality in the inequality and $\|\cdot\|_1$ is the trace norm, i.e., $\|O\|_1 := \mathrm{tr}\left(\sqrt{O^\dagger O}\right)$. Now, we can apply Proposition 16 to $\langle\mathbf{N}|[\hat{n}_X(t)]^{2s}|\mathbf{N}\rangle$, but we cannot ensure

$$\sum_{\mathbf{N}} \sqrt{\langle\mathbf{N}|\rho^2|\mathbf{N}\rangle} = \mathcal{O}(1). \tag{268}$$

Indeed, when ρ is given by a random pure state, i.e., $\rho = |\mathrm{rand}\rangle\langle\mathrm{rand}|$, we have

$$\sum_{\mathbf{N}} \sqrt{\langle\mathbf{N}|\rho^2|\mathbf{N}\rangle} = \sum_{\mathbf{N}} |\langle\mathbf{N}|\mathrm{rand}\rangle| = \mathrm{e}^{\mathcal{O}(|\Lambda|)}, \tag{269}$$

which leads the inequality (267) to a meaningless upper bound. This point necessitates us to utilize more refined analyses.

In considering the first order moment, we have considered the upper bound of

$$\mathrm{tr}\left(\hat{n}_X(\tau)\rho\right) \le \min_{X':X'\supseteq X} \mathrm{tr}\left(\hat{n}_{X'}(\tau)\rho\right). \tag{270}$$

We have aimed to estimate the minimization in the RHS of the above inequality. A simple generalization to high-order moments gives

$$\mathrm{tr}\left(\hat{n}_X(\tau)^s\rho\right) \le \min_{X':X'\supseteq X} \mathrm{tr}\left(\hat{n}_{X'}(\tau)^s\rho\right). \tag{271}$$

However, it is not enough to control a single parameter X' in deriving the desired bound below. In deriving a meaningful bound for higher order moments, the key idea here is to utilize the multi-parameter optimization by using the inequality (72):

$$\mathrm{tr}\left(\hat{n}_X(\tau)^s\rho\right) \le \min_{X_1:X_1\supseteq X}\min_{X_2:X_2\supseteq X}\cdots\min_{X_s:X_s\supseteq X} \mathrm{tr}\left[\hat{n}_{X_1}(\tau)\hat{n}_{X_2}(\tau)\cdots\hat{n}_{X_s}(\tau)\rho\right]. \tag{272}$$

Also, in Subtheorem 1, we have already obtained a general upper bound for $M_{X_1,X_2,\dots,X_s}(\tau)$.

Then, by solving the multi-parameter optimization problem (272), we can prove the following proposition:

Proposition 18. *We here adopt the same setup as that in Proposition 16. Then, we obtain*

$$[\hat{n}_X(\tau)]^s \preceq \left[\left(1+5c_{\tau,1}\lambda_{1/2}e^{-f_\tau \ell}\right)\hat{n}_{X[\ell]} + c_{\tau,1}e^{-3\ell/16}\hat{\mathscr{D}}_{X[\ell]} + c_{\tau,2}s\right]^s, \tag{273}$$

where $\hat{\mathscr{D}}_{X[\ell]}$ and f_τ have been defined in Eqs. (242) and (243).

3. Proof of Proposition 18

Without loss of generality, we can assume that ρ is given by a pure state, i.e., $\rho = |\psi\rangle\langle\psi|$. We note that $O_1 \preceq O_2$ is derived from $\langle\psi|O_1|\psi\rangle \preceq \langle\psi|O_2|\psi\rangle$ for all the quantum states. Then, by using Subtheorem 1, the multi-parameter optimization (272) reduces to

$$\begin{aligned}
\langle\psi|\hat{n}_X(\tau)^s|\psi\rangle &\le \min_{X_1:X_1\supseteq X}\min_{X_2:X_2\supseteq X}\cdots\min_{X_s:X_s\supseteq X}\langle\psi|\hat{n}_{X_1}(\tau)\hat{n}_{X_2}(\tau)\cdots\hat{n}_{X_s}(\tau)|\psi\rangle \\
&\le \min_{X_1:X_1\supseteq X}\min_{X_2:X_2\supseteq X}\cdots\min_{X_s:X_s\supseteq X}\langle\psi|\prod_{j=1}^s\left(\hat{n}_{X_j}+c_{\tau,1}\hat{\mathcal{D}}_{X_j}+c_{\tau,2}s\right)|\psi\rangle \\
&= \min_{X_1:X_1\supseteq X}\min_{X_2:X_2\supseteq X}\cdots\min_{X_s:X_s\supseteq X}\langle\psi|\prod_{j=1}^s\hat{n}_{\tau,X_j}|\psi\rangle,
\end{aligned} \tag{274}$$

where in the last equation we denote $\hat{n}_{X_j}+c_{\tau,1}\hat{\mathcal{D}}_{X_j}+c_{\tau,2}s$ by $\hat{n}_{\tau,X_j}$. We note that each of $\{\hat{n}_{\tau,X_j}\}$ commutes with $\hat{n}_i$ for $\forall i\in\Lambda$.

We define the following unnormalized quantum states $|\psi_{X_{1:j}}\rangle$ $(1\le j\le s)$:

$$\begin{aligned}
|\psi_{X_{1:j-1}}\rangle &:= \left(\hat{n}_{\tau,X_1}\hat{n}_{\tau,X_2}\cdots\hat{n}_{\tau,X_{j-1}}\hat{\mathcal{N}}_{\tau,X[\ell]}^{s-j}\right)^{1/2}|\psi\rangle, \\
\hat{\mathcal{N}}_{\tau,X[\ell]} &:= \left(1+5c_{\tau,1}\lambda_{1/2}e^{-f_\tau\ell}\right)\hat{n}_{X[\ell]}+c_{\tau,1}e^{-3\ell/16}\hat{\mathscr{D}}_{X[\ell]}+c_{\tau,2}s.
\end{aligned} \tag{275}$$

We then rewrite the optimization problem in (274) as

$$\min_{X_1:X_1\supseteq X}\min_{X_2:X_2\supseteq X}\cdots\min_{X_s:X_s\supseteq X}\langle\psi|\prod_{j=1}^s\hat{n}_{\tau,X_j}|\psi\rangle = \min_{X_1:X_1\supseteq X}\min_{X_2:X_2\supseteq X}\cdots\min_{X_s:X_s\supseteq X}\langle\psi_{X_{1:s-1}}|\hat{n}_{\tau,X_s}|\psi_{X_{1:s-1}}\rangle. \tag{276}$$

We can apply Proposition 16 to $\langle\psi_{X_{1:s-1}}|\hat{n}_{\tau,X_s}|\psi_{X_{1:s-1}}\rangle$, which yields

$$\min_{X_s:X_s\supseteq X}\langle\psi_{X_{1:s-1}}|\hat{n}_{\tau,X_s}|\psi_{X_{1:s-1}}\rangle \le \langle\psi_{X_{1:s-1}}|\hat{\mathcal{N}}_{\tau,X[\ell]}|\psi_{X_{1:s-1}}\rangle \tag{277}$$

for arbitrary choices of $\{X_j\}_{j=1}^{s-1}$ in $|\psi_{X_{1:s-1}}\rangle$. We then reduce Eq. (276) to

$$\begin{aligned}
&\min_{X_1:X_1\supseteq X}\min_{X_2:X_2\supseteq X}\cdots\min_{X_s:X_s\supseteq X}\langle\psi|\prod_{j=1}^s\hat{n}_{\tau,X_j}|\psi\rangle \\
&\le \min_{X_1:X_1\supseteq X}\min_{X_2:X_2\supseteq X}\cdots\min_{X_{s-1}:X_{s-1}\supseteq X}\langle\psi_{X_{1:s-1}}|\hat{\mathcal{N}}_{\tau,X[\ell]}|\psi_{X_{1:s-1}}\rangle \\
&= \min_{X_1:X_1\supseteq X}\min_{X_2:X_2\supseteq X}\cdots\min_{X_{s-1}:X_{s-1}\supseteq X}\langle\psi_{X_{1:s-2}}|\hat{n}_{\tau,X_{s-1}}|\psi_{X_{1:s-2}}\rangle.
\end{aligned} \tag{278}$$

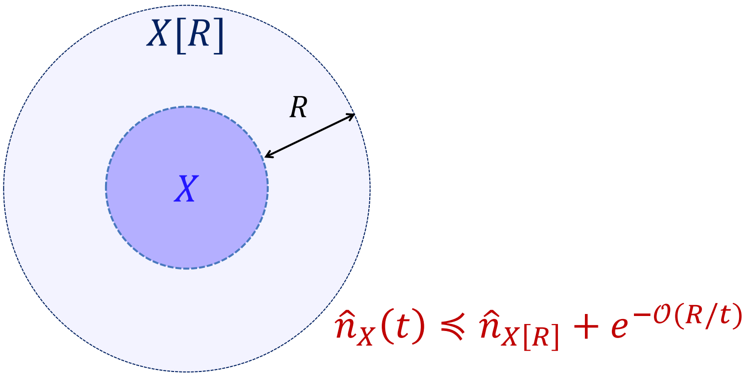

Supplementary Figure 5. Schematic picture of our setup for Theorem 1. We consider the boson number operator $\hat{n}_X$ in a region X. After a time evolution, $\hat{n}_X(t)$ is upper-bounded by the boson number $\hat{n}_{X[R]}$ on an extended subset $X[R]$, where the error is given by $e^{-\Omega(R/t)}$. The obtained inequality is given in the form of the operator inequality [see (282)], and hence it can be applied to an arbitrary initial state.

In the same way, we apply Proposition 16 to $\langle \psi_{X_{1:s-2}} | \hat{n}_{\tau,X_{s-1}} | \psi_{X_{1:s-2}} \rangle$, which further upper-bounds the RHS of the above inequality as

$$
\begin{aligned}
&\min_{X_1:X_1\supseteq X} \min_{X_2:X_2\supseteq X} \cdots \min_{X_s:X_s\supseteq X} \langle \psi | \prod_{j=1}^{s} \hat{n}_{\tau,X_j} | \psi \rangle \\
&\le \min_{X_1:X_1\supseteq X} \min_{X_2:X_2\supseteq X} \cdots \min_{X_{s-2}:X_{s-2}\supseteq X} \langle \psi_{X_{1:s-2}} | \hat{\mathcal{N}}_{\tau,X[\ell]} | \psi_{X_{1:s-2}} \rangle \\
&= \min_{X_1:X_1\supseteq X} \min_{X_2:X_2\supseteq X} \cdots \min_{X_{s-2}:X_{s-2}\supseteq X} \langle \psi_{X_{1:s-3}} | \hat{n}_{\tau,X_{s-2}} | \psi_{X_{1:s-3}} \rangle .
\end{aligned}
\tag{279}
$$

By repeating the same process, we finally obtain

$$
\min_{X_1:X_1\supseteq X} \min_{X_2:X_2\supseteq X} \cdots \min_{X_s:X_s\supseteq X} \langle \psi | \prod_{j=1}^{s} \hat{n}_{\tau,X_j} | \psi \rangle \le \langle \psi_{X_{1:0}} | \hat{\mathcal{N}}_{\tau,X[\ell]} | \psi_{X_{1:0}} \rangle = \langle \psi | \hat{\mathcal{N}}_{\tau,X[\ell]}^{s} | \psi \rangle . \tag{280}
$$

By applying the definition (275) to the above inequality, we prove the main inequality (273). This completes the proof. □

Supplementary Note 4. MAIN THEOREM ON THE BOSON CONCENTRATION

In this section, we consider a boson density after a time evolution for an arbitrary time t. For this purpose, we connect the short-time evolution by iteratively applying Proposition 18. Our goal is to prove that the boson number operator in a region X is upper-bounded by a number operator on an extended region $X[R]$ after a time evolution e^{-iHt}, where the length R is almost linear with the time t (see Fig. 5).

We here prove the following theorem:

Theorem 1. *Let τ be the maximum number such that $\tau \le 1/(4\gamma \bar{J})$ and t/τ becomes integer. Also, for $\ell = \lfloor R/(t/\tau) \rfloor = \Theta(R/t)$, we assume*

$$
e^{-f_\tau \ell} \le \frac{\tau}{5c_{\tau,1}\lambda_{1/2} t} \longrightarrow \ell \ge \frac{1}{f_\tau} \log\left(\frac{5c_{\tau,1}\lambda_{1/2} t}{\tau} \right), \tag{281}
$$

where f_τ has been defined in Eq. (243). We then upper-bound $[\hat{n}_X(t)]^s$ in the form of

$$
[\hat{n}_X(t)]^s \preceq \left[\left(1 + \frac{3t}{\tau}\delta_\ell \right) \hat{n}_{X[R]} + 2c_{\tau,1} e^{-3\ell/16} \hat{\mathscr{D}}_{X[R]} + \left(\frac{c_{\tau,2} t}{\tau} + \tilde{\delta}_\ell \right) s \right]^s \tag{282}
$$

with $\hat{\mathscr{D}}_{X[R]} = \sum_{j\in X[R]^c} e^{-3d_{j,X[R]}/4} \hat{n}_j$ as in Eq. (242) and

$$
\delta_\ell := 5c_{\tau,1}\lambda_{1/2} e^{-f_\tau \ell}, \quad \tilde{\delta}_\ell := \frac{c_{\tau,2} t}{\tau} \left(\frac{3t\delta_\ell}{\tau} + 2c_{\tau,1}\lambda_{3/4} e^{-3\ell/16} |\partial X[R]| \right). \tag{283}
$$

Remark. Because of the condition (281) and $\ell \propto R/t$, the length R should be as large as

$$
R \gtrsim t \log(t). \tag{284}
$$

Then, because of $\delta_\ell = e^{-\Theta(\ell)}$ and $\tilde{\delta}_\ell = e^{-\Theta(\ell)}$, we obtain

$$[\hat{n}_X(t)]^s \preceq \left[\hat{n}_{X[R]} + e^{-\Theta(R/t)}\left(\hat{n}_{X[R]} + \hat{\mathscr{D}}_{X[R]}\right) + \Theta(st)\right]^s, \tag{285}$$

where we use the Θ notation in Eq. (9). Therefore, for sufficiently large R, we have $[\hat{n}_X(t)]^s \lesssim \left[\hat{n}_{X[R]} + \Theta(st)\right]^s$.

A. Proof of Theorem 1

For the proof, we iteratively utilize Proposition 18. We decompose the total time t into m_0 pieces:

$$m_0 = \frac{t}{\tau} = \mathcal{O}(t), \tag{286}$$

where we have chosen the maximum τ such that $\tau \le 1/(4\gamma\bar{J})$ and t/τ becomes integer. In the proof, we consider the case that $\ell = \lfloor R/m_0 \rfloor = R/m_0$, but the generalization is straightforward. For simplicity of notation, we denote C_0 by

$$C_0 := 1 + 5c_{\tau,1}\lambda_{1/2}e^{-f_\tau \ell} = 1 + \delta_\ell, \quad \delta_\ell := 5c_{\tau,1}\lambda_{1/2}e^{-f_\tau \ell} \tag{287}$$

We then reduce the inequality (273) to

$$[\hat{n}_X(\tau)]^s \preceq \left[C_0\hat{n}_{X[\ell]} + c_{\tau,1}e^{-3\ell/16}\hat{\mathscr{D}}_{X[\ell]} + c_{\tau,2}s\right]^s \tag{288}$$

for arbitrary $X \subseteq \Lambda$ and $\tau \le 1/(4\gamma\bar{J})$.

The condition (281) immediately implies the inequality of $\delta_\ell \le 1/m_0$ from the definition $\delta_\ell := 5c_{\tau,1}\lambda_{1/2}e^{-f_\tau \ell}$. This yields

$$C_0^m = (1+\delta_\ell)^m \le 1 + m(e-1)\delta_\ell \le 1 + 2m\delta_\ell \quad \text{for} \quad m \le m_0, \tag{289}$$

where we use $(1+z)^m \le 1 + m(e-1)z$ for $z \le 1/m$. Moreover, $\delta_\ell \le 1/m_0 \le 1$ implies

$$e^{-f_\tau \ell} = \left(1 + \frac{1}{5c_{\tau,1}\lambda_{1/2} + 2}\right)^{-\ell/2} \le \frac{1}{5c_{\tau,1}\lambda_{1/2}}. \tag{290}$$

Because $\left[1 + 1/\left(5c_{\tau,1}\lambda_{1/2} + 2\right)\right]^{1/2} \le (8/7)^{1/2} \le e^{1/14}$ from $c_{\tau,1} \ge 1$ and $\lambda_{1/2} \ge 1$, we also obtain

$$e^{-\ell/14} \le \frac{1}{5c_{\tau,1}\lambda_{1/2}}. \tag{291}$$

For the time-evolved operator $[\hat{n}_X(m\tau)]^s$, we prove the following proposition:

Proposition 19. *For an arbitrary time $m\tau$ ($m \le m_0$), we obtain the operator inequality as*

$$[\hat{n}_X(m\tau)]^s \preceq \left[(1+3m\delta_\ell)\,\hat{n}_{X[m\ell]} + 2c_{\tau,1}e^{-3\ell/16}\hat{\mathscr{D}}_{X[m\ell]} + \left(c_{\tau,2}m + \tilde{\delta}_\ell\right)s\right]^s, \tag{292}$$

where we define $\tilde{\delta}_\ell$ as

$$\tilde{\delta}_\ell := \frac{c_{\tau,2}t}{\tau}\left(\frac{3t\delta_\ell}{\tau} + 2c_{\tau,1}\lambda_{3/4}e^{-3\ell/16}|\partial X[R]|\right). \tag{293}$$

From Proposition 19 with $m = m_0$ (i.e., $m\tau = m_0\tau = t$), we obtain

$$\begin{aligned}[\hat{n}_X(t)]^s &\preceq \left[(1+3m_0\delta_\ell)\,\hat{n}_{X[m_0\ell]} + 2c_{\tau,1}e^{-3\ell/16}\hat{\mathscr{D}}_{X[m_0\ell]} + \left(c_{\tau,2}m_0 + \tilde{\delta}_\ell\right)s\right]^s \\ &= \left[\left(1 + \frac{3t}{\tau}\delta_\ell\right)\hat{n}_{X[R]} + 2c_{\tau,1}e^{-3\ell/16}\hat{\mathscr{D}}_{X[R]} + \left(\frac{c_{\tau,2}t}{\tau} + \tilde{\delta}_\ell\right)s\right]^s,\end{aligned} \tag{294}$$

where we use $m_0\ell = R$ and $m_0 = t/\tau$. This proves the main inequality (282). □

B. Proof of Proposition 19

For the proof, we upper-bound $[\hat{n}_X(m\tau)]^s$ in the form of

$$[\hat{n}_X(m\tau)]^s \preceq \left[C_{m,0}\hat{n}_{X[m\ell]} + C_{m,1}\hat{\mathscr{D}}_{X[m\ell]} + C_{m,2}s\right]^s. \tag{295}$$

For $m=0$, we trivially obtain the inequality (295) by choosing

$$C_{0,0}=1, \quad C_{0,1}=C_{0,2}=0. \tag{296}$$

For $m=1$, the parameters are given by

$$C_{1,0}=C_0, \quad C_{0,1}=c_{\tau,1}e^{-3\ell/16}, \quad C_{0,2}=c_{\tau,2}. \tag{297}$$

In the following, we upper-bound the parameters $\{C_{m+1,0}, C_{m+1,1}, C_{m+1,2}\}$ under the inequality of (295) for a fixed m.

We start from the time evolution of

$$[\hat{n}_X(m\tau+\tau)]^s = e^{iH\tau}[\hat{n}_X(m\tau)]^s e^{-iH\tau} \preceq \left[C_{m,0}\hat{n}_{X[m\ell]}(\tau) + C_{m,1}\hat{\mathscr{D}}_{X[m\ell]}(\tau) + C_{m,2}s\right]^s, \tag{298}$$

where we use $U^\dagger O_1 U \preceq U^\dagger O_2 U$ for an arbitrary unitary operator U if $O_1 \preceq O_2$. By using Proposition 18, we obtain

$$\begin{aligned}\left[C_{m,0}\hat{n}_{X[m\ell]}(\tau) + C_{m,1}\hat{\mathscr{D}}_{X[m\ell]}(\tau) + C_{m,2}s\right]^s \preceq \Bigg\{ & C_{m,0}\left[C_0\hat{n}_{X[m\ell+\ell]} + c_{\tau,1}e^{-3\ell/16}\hat{\mathscr{D}}_{X[m\ell+\ell]} + c_{\tau,2}s\right] \\ & + C_{m,1}\sum_{j\in X[m\ell]^{\rm c}} e^{-3d_{j,X[m\ell]}/4}\left(\hat{n}_j + c_{\tau,1}\hat{\mathcal{D}}_j + c_{\tau,2}s\right) + C_{m,2}s\Bigg\}^s, \end{aligned} \tag{299}$$

where we apply Subtheorem 1 to $\hat{\mathscr{D}}_{X[m\ell]}(\tau) = \sum_{j\in X[m\ell]^{\rm c}} e^{-3d_{j,X[m\ell]}/4}\hat{n}_j(\tau)$. First of all, we consider the coefficient of s in (299), which is given by

$$c_{\tau,2}C_{m,0} + C_{m,2} + c_{\tau,2}C_{m,1}\sum_{j\in X[m\ell]^{\rm c}} e^{-3d_{j,X[m\ell]}/4} \preceq c_{\tau,2}C_{m,0} + C_{m,2} + c_{\tau,2}C_{m,1}\lambda_{3/4}|\partial X[m\ell]|, \tag{300}$$

where the inequality is derived from

$$\sum_{j\in X[m\ell]^{\rm c}} e^{-3d_{j,X[m\ell]}/4} \le \sum_{j\in X[m\ell]^{\rm c}}\sum_{i\in\partial X[m\ell]} e^{-3d_{i,j}/4} \le \sum_{i\in\partial X[m\ell]}\sum_{j\in\Lambda} e^{-3d_{i,j}/4} \le \lambda_{3/4}|\partial X[m\ell]|. \tag{301}$$

We next consider the following term in the inequality (299):

$$\begin{aligned} C_{m,1}\sum_{j\in X[m\ell]^{\rm c}} e^{-3d_{j,X[m\ell]}/4}\left(\hat{n}_j + c_{\tau,1}\hat{\mathcal{D}}_j\right) &= C_{m,1}\sum_{j\in X[m\ell]^{\rm c}} e^{-3d_{j,X[m\ell]}/4}\left(\hat{n}_j + c_{\tau,1}\sum_{j'\in\Lambda} e^{-d_{j,j'}}\hat{n}_{j'}\right) \\ &\preceq C_{m,1}\sum_{j\in\Lambda} e^{-3d_{j,X[m\ell]}/4}\left(1 + c_{\tau,1}\lambda_{1/4}\right)\hat{n}_j, \end{aligned} \tag{302}$$

where we use the inequality of $d_{j,X} + d_{j,j'} \ge d_{j',X}$ for $\forall X \subset \Lambda$, which yields

$$\sum_{j\in\Lambda} e^{-3d_{j,X[m\ell]}/4}\sum_{j'\in\Lambda} e^{-d_{j,j'}}\hat{n}_{j'} \le \sum_{j\in\Lambda}\sum_{j'\in\Lambda} e^{-3d_{j',X[m\ell]}/4} e^{-d_{j,j'}/4}\hat{n}_{j'} \le \lambda_{1/4}\sum_{j'\in\Lambda} e^{-3d_{j',X[m\ell]}/4}\hat{n}_{j'}. \tag{303}$$

We further decompose

$$\sum_{j\in\Lambda} e^{-3d_{j,X[m\ell]}/4}\hat{n}_j = \sum_{j\in X[m\ell+\ell]} e^{-3d_{j,X[m\ell]}/4}\hat{n}_j + \sum_{j\in X[m\ell+\ell]^{\rm c}} e^{-3d_{j,X[m\ell]}/4}\hat{n}_j. \tag{304}$$

For the first term in Eq. (304), we immediately obtain

$$\sum_{j\in X[m\ell+\ell]} e^{-3d_{j,X[m\ell]}/4}\hat{n}_j \le \sum_{j\in X[m\ell+\ell]} \hat{n}_j = \hat{n}_{X[m\ell+\ell]}. \tag{305}$$

For the second term in Eq. (304), by using $d_{j,X[m\ell]} = d_{j,X[m\ell+\ell]} + \ell$ for $j \in X[m\ell+\ell]^{\rm c}$, we have

$$\sum_{j\in X[m\ell+\ell]^{\rm c}} e^{-3d_{j,X[m\ell]}/4}\hat{n}_j = e^{-3\ell/4} \sum_{j\in X[m\ell+\ell]^{\rm c}} e^{-3d_{j,X[m\ell+\ell]}/4}\hat{n}_j = e^{-3\ell/4}\hat{\mathscr{D}}_{X[m\ell+\ell]}. \tag{306}$$

Then, by combining Eqs. (304), (305), and (306) with the inequality (302), we obtain

$$C_{m,1}\sum_{j\in X[m\ell]^{\rm c}} e^{-3d_{j,X[m\ell]}/4}\left(\hat{n}_j + c_{\tau,1}\hat{\mathcal{D}}_j\right) \preceq C_{m,1}\left(1+c_{\tau,1}\lambda_{1/4}\right)\left(\hat{n}_{X[m\ell+\ell]} + e^{-3\ell/4}\hat{\mathscr{D}}_{X[m\ell+\ell]}\right). \tag{307}$$

Finally, by applying the inequalities (300) and (307) to (299), we arrive at the inequality of

$$\begin{aligned}
&\left[C_{m,0}\hat{n}_{X[m\ell]}(\tau) + C_{m,1}\hat{\mathscr{D}}_{X[m\ell]}(\tau) + C_{m,2}s\right]^s \\
&\preceq \Bigg\{\left[C_0C_{m,0} + C_{m,1}\left(1+c_{\tau,1}\lambda_{1/4}\right)\right]\hat{n}_{X[m\ell+\ell]} + \left[c_{\tau,1}e^{-3\ell/16} + C_{m,1}\left(1+c_{\tau,1}\lambda_{1/4}\right)e^{-3\ell/4}\right]\hat{\mathscr{D}}_{X[m\ell+\ell]} \\
&\quad + s\left(c_{\tau,2}C_{m,0} + C_{m,2} + c_{\tau,2}C_{m,1}\lambda_{3/4}|\partial X[m\ell]|\right)\Bigg\}^s.
\end{aligned} \tag{308}$$

The above inequality yields

$$\begin{aligned}
C_{m+1,0} &= C_0C_{m,0} + C_{m,1}\left(1+c_{\tau,1}\lambda_{1/4}\right), \\
C_{m+1,1} &= c_{\tau,1}e^{-3\ell/16} + C_{m,1}\left(1+c_{\tau,1}\lambda_{1/4}\right)e^{-3\ell/4}, \\
C_{m+1,2} &= c_{\tau,2}C_{m,0} + C_{m,2} + c_{\tau,2}C_{m,1}\lambda_{3/4}|\partial X[m\ell]|.
\end{aligned} \tag{309}$$

For an arbitrary numerical sequence $\{a_m\}$ such that $a_{m+1} = b_1a_m + b_2$, we have

$$a_m = \frac{b_2}{1-b_1} + b_1^m\left(a_0 - \frac{b_2}{1-b_1}\right) \quad \text{for} \quad \forall m. \tag{310}$$

We first derive the explicit form of $C_{m,1}$. For the second equation in (309), we use Eq. (310) with $C_{0,1} = 0$ as in Eq. (296) to derive

$$C_{m,1} = \left\{1 - \left[\left(1+c_{\tau,1}\lambda_{1/4}\right)e^{-3\ell/4}\right]^m\right\}\frac{c_{\tau,1}e^{-3\ell/16}}{1-\left(1+c_{\tau,1}\lambda_{1/4}\right)e^{-3\ell/4}} \le 2c_{\tau,1}e^{-3\ell/16} \le 2c_{\tau,1}e^{-2f_\tau\ell}, \tag{311}$$

where we use the inequality (291) to derive $1-\left(1+c_{\tau,1}\lambda_{1/4}\right)e^{-3\ell/4} \ge 1/2$ and use $e^{-f_\tau} > e^{-1/14}$ to derive $e^{-3\ell/16} \le e^{-2f_\tau\ell}$.

We then reduce the first equation in (309) to

$$C_{m+1,0} \le C_0C_{m,0} + 2c_{\tau,1}e^{-2f_\tau\ell}\left(1+c_{\tau,1}\lambda_{1/4}\right), \tag{312}$$

which yields from $C_{0,0} = 1$ in Eq. (296)

$$\begin{aligned}
C_{m,0} &\le C_0^m + \frac{2c_{\tau,1}e^{-2f_\tau\ell}\left(1+c_{\tau,1}\lambda_{1/4}\right)}{1-C_0}(1-C_0^m) \\
&\le 1 + 2m\left[\delta_\ell + 2c_{\tau,1}e^{-2f_\tau\ell}\left(1+c_{\tau,1}\lambda_{1/4}\right)\right] \le 1 + 3m\delta_\ell.
\end{aligned} \tag{313}$$

In the second inequality above, we use the inequality (289) and the following upper bound:

$$\frac{C_0^m - 1}{C_0 - 1} = \sum_{m'=0}^{m-1} C_0^{m'} \le m + 2\delta_\ell\sum_{m'=0}^{m-1} m' = m + \delta_\ell m(m-1) \le 2m, \tag{314}$$

Note that we have $\delta_\ell \le 1/m_0 \le 1/m$. In the third inequality of (313), we use the definition of $\delta_\ell = 5c_{\tau,1}\lambda_{1/2}e^{-f_\tau\ell}$ and the inequality (290). Finally, by using the inequalities (311) and (313), we reduce the third equation in (309) to

$$\begin{aligned}
C_{m+1,2} &\le c_{\tau,2}\left(1+3m\delta_\ell\right) + C_{m,2} + 2c_{\tau,1}c_{\tau,2}e^{-3\ell/16}\lambda_{3/4}|\partial X[m\ell]| \\
&\le C_{m,2} + c_{\tau,2}\left[1 + 3m\delta_\ell + 2c_{\tau,1}\lambda_{3/4}e^{-3\ell/16}|\partial X[R]|\right],
\end{aligned} \tag{315}$$

where we use $m \le m_0$ and $m\ell \le R$. The above inequality immediately gives

$$C_{m,2} \le c_{\tau,2}m + c_{\tau,2}m_0\left(3m_0\delta_\ell + 2c_{\tau,1}\lambda_{3/4}e^{-3\ell/16}|\partial X[R]|\right) = c_{\tau,2}m + \tilde{\delta}_\ell, \tag{316}$$

where we use $C_{0,2} = 0$.

By using the inequalities (311), (313) and (316), we reduce the upper bound (295) to

$$[\hat{n}_X(m\tau)]^s \preceq \left[\left(1+3m\delta_\ell\right)\hat{n}_{X[m\ell]} + 2c_{\tau,1}e^{-3\ell/16}\hat{\mathscr{D}}_{X[m\ell]} + \left(c_{\tau,2}m + \tilde{\delta}_\ell\right)s\right]^s. \tag{317}$$

We thus prove Proposition 19. □

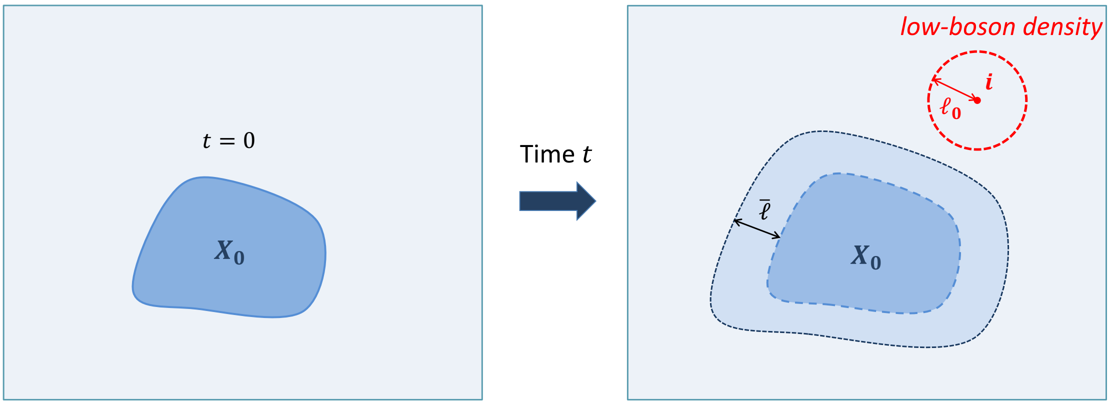

Supplementary Figure 6. Schematic picture of our setup for Proposition 20. We here consider an initial state where the low-boson density condition in Definition 3 is satisfied outside the region X_0. After a time evolution, the concentration of the boson density may occur. Around the region X_0 (or in the extended region $X_0[\bar{\ell}]$), we cannot ensure that the boson density should be suppressed. On the other hand, in the region $X_0[\bar{\ell}]^{\rm c}$, we can prove that the number of bosons in a ball region $i[\ell_0]$ obeys the (sub)exponentially decaying probability distribution as long as ℓ_0 satisfies (320). In our theorem, we prove that the length of $\bar{\ell}$ is roughly given by $t\log(t)$ [see (323)].

C. Boson density after time evolution for an initial state with low-boson density

We apply Theorem 1 to a boson density after a time evolution for an initial state ρ_0, which satisfies the following conditions:

$$\mathrm{tr}\left(\rho_0 \hat{n}^s_{X_0[r]}\right) \le \frac{1}{e}\left[\frac{b_0}{e}\left(q_0 + s^\kappa q_1 \max(r^D, 1)|X_0|\right)\right]^s, \tag{318}$$

$$\mathrm{tr}\left(\rho_0 \hat{n}^s_{i[\ell_0]}\right) \le \frac{1}{e}\left(\frac{b_0}{e} s^\kappa \ell_0^D\right)^s \quad \text{for} \quad i[\ell_0] \in X_0^{\rm c}, \quad \forall \ell_0 \ge \bar{\ell}_0, \tag{319}$$

where b_0 and κ (≥ 1) are constants of $\mathcal{O}(1)$. In this setup, unlike the condition in Definition 3, the boson density can be large only in the region X_0. Our purpose is to prove that the boson density around the site i is still small after a time evolution if i is sufficiently separated from the region X (see Fig. 6).

We here prove the following corollary:

Proposition 20. *Let us consider an initial state ρ_0 that satisfies the assumptions (318) and (319). Then, under the condition of*

$$\ell_0 \ge \max(\bar{\ell}_0, 2D\bar{\ell}), \tag{320}$$

we obtain

$$\mathrm{tr}\left[\rho_0(t) \hat{n}^s_{i[\ell_0]}\right] \le \frac{1}{e}\left(\frac{2b_0}{e} s^\kappa \ell_0^D\right)^s \quad \textit{for} \quad i[\ell_0] \in X_0[\bar{\ell}]^{\rm c}, \tag{321}$$

and

$$\mathrm{tr}\left[\rho_0(t) \hat{n}^s_{X_0[\ell_0]}\right] \le \frac{1}{e}\left[\frac{2b_0}{e}\left(q_0 + s^\kappa q_1 \ell_0^D |X_0|\right)\right]^s, \tag{322}$$

where $\bar{\ell}$ is given by

$$\bar{\ell} = t\log[\Theta(tq_0q_1|X_0|)]. \tag{323}$$

Remark. From the inequality (321) with $s = 1$, roughly speaking, the average boson number in a region $i[\bar{\ell}]$ (i.e., $s = 1$) is proportional to $\bar{\ell}^D$, which gives

$$\frac{1}{|i[\ell_0]|}\mathrm{tr}\left[\rho_0(t)\hat{n}^s_{i[\ell_0]}\right] = \mathcal{O}(1).$$

In particular, if we have no constraints in the region X_0, we can simplify Proposition 20. Here, we obtain the inequality (321) for arbitrary $i \in \Lambda$ with

$$\bar{\ell} = t\log[\Theta(t)]. \tag{324}$$

1. Proof of Proposition 20

Let us consider an arbitrary operator Q such that

$$Q = \nu_X \hat{n}_X + \sum_{i\in X^{\rm c}} \nu_i \hat{n}_i, \tag{325}$$

where X is arbitrarily chosen and we assume $\nu_X \ge 0$ and $\nu_i \ge 0$ ($i \in X^{\rm c}$). Then, for arbitrary quantum state ρ, we have

$$\mathrm{tr}\,(\rho Q^s) = \mathrm{tr}\left[\rho\left(\nu_X \hat{n}_X + \sum_{i\in X^{\rm c}} \nu_i \hat{n}_i\right)^s\right] \le \left(\nu_X \langle \hat{n}_X^s\rangle_\rho^{1/s} + \sum_{i\in\Lambda} \nu_i \langle \hat{n}_i^s\rangle_\rho^{1/s}\right)^s, \tag{326}$$

where we use the generalized Hölder inequality [3] to obtain

$$\mathrm{tr}\,(\rho \hat{n}_{X_1}\hat{n}_{X_2}\cdots\hat{n}_{X_s}) \le \prod_{j=1}^s \left\langle \hat{n}_{X_j}^s\right\rangle_\rho^{1/s}. \tag{327}$$

Note that $\langle \hat{n}_i^s\rangle_\rho$ means $\langle \hat{n}_i^s\rangle_\rho = \mathrm{tr}\,(\rho\hat{n}_i^s)$.

We here apply the inequality (326) to $\mathrm{tr}\,[\rho_0(t)\hat{n}_X^s] = \mathrm{tr}\,\{\rho_0[\hat{n}_X(t)]^s\}$. We first use Proposition 18 to derive

$$\mathrm{tr}\,[\rho_0(t)\hat{n}_X^s] \le \mathrm{tr}\left\{\rho_0\left[\left(1+\frac{3t}{\tau}\delta_\ell\right)\hat{n}_{X[R]} + 2c_{\tau,1}e^{-3\ell/16}\hat{\mathscr{D}}_{X[R]} + \left(\frac{c_{\tau,2}t}{\tau}+\tilde{\delta}_\ell\right)s\right]^s\right\} \tag{328}$$

with $\ell = \Theta(R/t)$, where we choose R such that the condition (281) is satisfied, i.e., $R \gtrsim t\log(t)$. The inequality (326) reduces the above inequality

$$\{\mathrm{tr}\,[\rho_0(t)\hat{n}_X^s]\}^{1/s} \le \left(1+\frac{3t}{\tau}\delta_\ell\right)\left\langle \hat{n}_{X[R]}^s\right\rangle_{\rho_0}^{1/s} + 2c_{\tau,1}e^{-3\ell/16}\left\langle \hat{\mathscr{D}}_{X[R]}^s\right\rangle_{\rho_0}^{1/s} + \left(\frac{c_{\tau,2}t}{\tau}+\tilde{\delta}_\ell\right)s. \tag{329}$$

We now consider the case of $X = i[\ell_0]$ such that $i[\ell_0+R]\cap X_0 = \emptyset$ and $\ell_0 \ge 2DR$. From the conditions (318) and (319), we obtain

$$\left\langle \hat{n}_{i[\ell_0+R]}^s\right\rangle_{\rho_0}^{1/s} \le \frac{1}{e^{1/s}}\frac{b_0}{e}s^\kappa(\ell_0+R)^D, \tag{330}$$

and

$$\begin{aligned}\left\langle \hat{\mathscr{D}}_{i[\ell_0+R]}^s\right\rangle_{\rho_0}^{1/s} &\le \langle \hat{n}_{X_0}^s\rangle_{\rho_0}^{1/s} + \left\langle\left(\sum_{j\in i[\ell_0+R]^{\rm c}\setminus X_0} e^{-3d_{j,i[\ell_0+R]}/4}\hat{n}_j\right)^s\right\rangle_{\rho_0}^{1/s} \\ &\le \frac{1}{e^{1/s}}\frac{b_0}{e}(q_0+s^\kappa q_1|X_0|) + \frac{1}{e^{1/s}}\left(\frac{b_0}{e}s^\kappa\ell_0^D\right)e^{3/2}\left[1+2^D D!(4/3)^{D+1}\right],\end{aligned} \tag{331}$$

where we use $R \le \ell_0$ from $R \le \ell_0/(2D)$ and

$$\begin{aligned}&\left\langle\left(\sum_{j\in i[\ell_0+R]^{\rm c}\setminus X_0} e^{-3d_{j,i[\ell_0+R]}/4}\hat{n}_j\right)^s\right\rangle_{\rho_0}^{1/s} \le \sum_{m=1}^\infty e^{-3(m-1)\ell_0/4}\left\langle \hat{n}_{i[(m+1)\ell_0+R]\setminus i[m\ell_0+R]}^s\right\rangle_{\rho_0}^{1/s} \\ &\le \sum_{m=1}^\infty e^{-3(m-1)/4}\left\langle \hat{n}_{i[(m+2)\ell_0]}^s\right\rangle_{\rho_0}^{1/s} \le \frac{1}{e^{1/s}}\left(\frac{b_0}{e}s^\kappa\ell_0^D\right)\sum_{m=1}^\infty e^{-3(m-1)/4}(m+2)^D \\ &\le \frac{1}{e^{1/s}}\left(\frac{b_0}{e}s^\kappa\ell_0^D\right)\sum_{m=1}^\infty e^{-3(m+2)/4+9/4}(m+2)^D \le \frac{1}{e^{1/s}}\left(\frac{b_0}{e}s^\kappa\ell_0^D\right)e^{3/2}\left[1+2^D D!(4/3)^{D+1}\right].\end{aligned} \tag{332}$$

By using the above inequalities, we have

$$\begin{aligned}&\left\{\mathrm{tr}\left[\rho_0(t)\hat{n}_{i[\ell_0]}^s\right]\right\}^{1/s} \\ &\le \frac{1}{e^{1/s}}\left(\frac{b_0}{e}s^\kappa\ell_0^D\right)\left[\left(1+\frac{3t}{\tau}\delta_\ell\right)(1+R/\ell_0)^D + 2c_{\tau,1}e^{-3\ell/16}\left(\frac{q_0 s^{-\kappa}+q_1|X_0|}{\ell_0^D} + e^{3/2}\left[1+2^D D!(4/3)^{D+1}\right]\right)\right. \\ &\quad\left. + \frac{e^{1+1/s}}{b_0\ell_0^D}\left(\frac{c_{\tau,2}t}{\tau}+\tilde{\delta}_\ell\right)s^{1-\kappa}\right].\end{aligned} \tag{333}$$

By using the Θ notation in Eq. (9), we can obtain

$$\left(1+\frac{3t}{\tau}\delta_\ell\right)(1+R/\ell_0)^D+2c_{\tau,1}e^{-3\ell/16}\left(\frac{q_0s^{-\kappa}+q_1|X_0|}{\ell_0^D}+e^{3/2}\left[1+2^DD!(4/3)^{D+1}\right]\right)+\frac{e^{1+1/s}}{b_0\ell_0^D}\left(\frac{c_{\tau,2}t}{\tau}+\tilde{\delta}_\ell\right)s^{1-\kappa}$$
$$\leq\left(1+\Theta(t)e^{-\Theta(R/t)}\right)(1+R/\ell_0)^D+e^{-\Theta(R/t)}\Theta\left(q_0,q_1|X_0|\right). \tag{334}$$

Thus, under the assumption of $R/\ell_0 \leq 1/(2D)$ (or $\ell_0 \geq 2DR$), we can find a length R such that

$$\left(1+\Theta(t)e^{-\Theta(R/t)}\right)(1+R/\ell_0)^D+e^{-\Theta(R/t)}\Theta\left(q_0,q_1|X_0|\right)\leq 2, \tag{335}$$

which is achieved by choosing $R=\bar{\ell}$ with

$$\bar{\ell}=t\log[\Theta(tq_0q_1|X_0|)]. \tag{336}$$

We thus prove the first inequality (321).

We then consider the case where $X=X_0[\ell_0]$. From the condition (318), we obtain

$$\left\langle \hat{n}^s_{X_0[\ell_0+R]}\right\rangle^{1/s}_{\rho_0}\leq\frac{1}{e}\left[\frac{b_0}{e}\left(q_0+s^\kappa q_1(\ell_0+R)^D|X_0|\right)\right], \tag{337}$$

and

$$\left\langle \hat{\mathscr{D}}^s_{X_0[\ell_0+R]}\right\rangle^{1/s}_{\rho_0}\leq\left\langle\left(\sum_{j\in X_0[\ell_0+R]^{\rm c}}e^{-3d_{j,X_0[\ell_0+R]}/4}\hat{n}_j\right)^s\right\rangle^{1/s}_{\rho_0}$$
$$\leq\frac{1}{e^{1/s}}\left[\frac{b_0}{e}\left(q_0+s^\kappa q_1\ell_0^D|X_0|\right)\right]e^{3/2}\left[1+2^DD!(4/3)^{D+1}\right], \tag{338}$$

where we use

$$\left\langle\left(\sum_{j\in X_0[\ell_0+R]^{\rm c}}e^{-3d_{j,X_0[\ell_0+R]}/4}\hat{n}_j\right)^s\right\rangle^{1/s}_{\rho_0}\leq\sum_{m=1}^\infty e^{-3(m-1)/4}\left\langle\hat{n}^s_{X_0[(m+1)\ell_0+R]\setminus X_0[m\ell_0+R]}\right\rangle^{1/s}_{\rho_0}$$
$$\leq\sum_{m=1}^\infty e^{-3(m-1)/4}\left\langle\hat{n}^s_{X_0[(m+2)\ell_0]}\right\rangle^{1/s}_{\rho_0}\leq\sum_{m=1}^\infty e^{-3(m-1)/4}\frac{1}{e^{1/s}}\left[\frac{b_0}{e}\left(q_0+s^\kappa q_1[(m+2)\ell_0]^D|X_0|\right)\right]$$
$$\leq\frac{1}{e^{1/s}}\left[\frac{b_0}{e}\left(q_0+s^\kappa q_1\ell_0^D|X_0|\right)\right]\sum_{m=1}^\infty e^{-3(m+2)/4+9/4}(m+2)^D\leq\frac{1}{e^{1/s}}\left[\frac{b_0}{e}\left(q_0+s^\kappa q_1\ell_0^D|X_0|\right)\right]e^{3/2}\left[1+2^DD!(4/3)^{D+1}\right].$$

By applying the above inequalities to (329), we can derive

$$\left\{{\rm tr}\left[\rho_0(t)\hat{n}^s_{X_0[\ell_0]}\right]\right\}^{1/s}$$
$$\leq\frac{1}{e}\left[\frac{b_0}{e}\left(q_0+s^\kappa q_1\ell_0^D|X_0|\right)\right]\left[\left(1+\frac{3t}{\tau}\delta_\ell\right)(1+R/\ell_0)^D+2c_{\tau,1}e^{-3\ell/16}\cdot e^{3/2}\left[1+2^DD!(4/3)^{D+1}\right]\right.$$
$$\left.+\frac{e^{1+1/s}}{b_0q_1\ell_0^D|X_0|}\left(\frac{c_{\tau,2}t}{\tau}+\tilde{\delta}_\ell\right)s^{1-\kappa}\right]. \tag{339}$$

Therefore, under the assumption of $\ell_0\geq 2DR$, we can obtain

$$\left(1+\frac{3t}{\tau}\delta_\ell\right)(1+R/\ell_0)^D+2c_{\tau,1}e^{-3\ell/16}e^{3/2}\left[1+2^DD!(4/3)^{D+1}\right]+\frac{e^{1+1/s}}{b_0q_1\ell_0^D|X_0|}\left(\frac{c_{\tau,2}t}{\tau}+\tilde{\delta}_\ell\right)s^{1-\kappa}\leq 2 \tag{340}$$

by choosing $R=\bar{\ell}$ as in Eq. (336). We thus prove the second inequality (322). This completes the proof. □

Supplementary Note 5. RESTRICTION ON THE OUTFLOW OF BOSONS

In this section, we aim to bound a boson number in a particular region from below. As in Fig. 7, we consider a setup that many bosons originally concentrated in a region X:

$$\Pi_{X,\geq N}\rho=\rho, \tag{341}$$

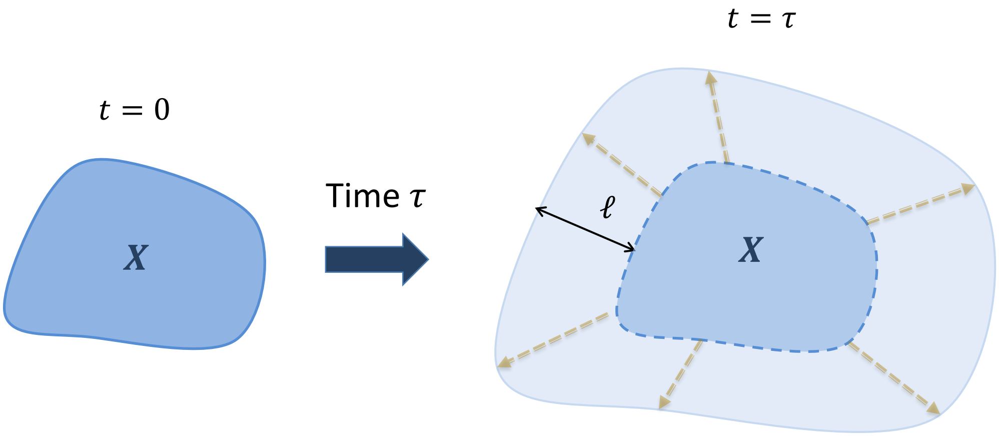

Supplementary Figure 7. Schematic picture of the setup in Sec. Supplementary Note 5. We consider an initial state where at least N bosons exist inside the region $X \subset \Lambda$. After a time evolution, these bosons may escape outside the region X. Even though, if the time is short, we expect that the bosons still remain in the region of around X. Mathematically, as the width ℓ increases, the probability of observing $\mathcal{O}(N)$ bosons in $X[\ell]$ is exponentially close to 1 (see Proposition 22).

where N is a fixed number. Unlike the setup in Ref. [2], there exist bosons outside the region X. Then, after a short-time evolution, the bosons are expected to concentrate around the region X, i.e.,

$$\mathrm{tr}\left[\Pi_{X[\ell],\le N-\delta N}\rho(\tau)\right] \approx g(\delta N) \tag{342}$$

for sufficiently large ℓ, where $g(x)$ is a rapidly decaying function. We need to consider such an upper bound in proving the optimal Lieb-Robinson bound in one-dimensional systems (see Sec. Supplementary Note 10 E 2).

A. Moment lower bound

First of all, we can prove that in the level of the expectation value. We can obtain

$$\langle\psi_0(\tau)|\hat{n}_{X[\ell]}|\psi_0(\tau)\rangle \gtrsim \langle\psi_0|\hat{n}_X|\psi_0\rangle - e^{-\Omega(\ell)} \ge N - e^{-\Omega(\ell)}. \tag{343}$$

Thus, we ensure that the number of bosons is lower-bounded as long as we consider an extended region $X[\ell]$ with a sufficiently large ℓ. For higher-order moments, we can also derive

$$\langle\psi_0(\tau)|\hat{n}^s_{X[\ell]}|\psi_0(\tau)\rangle \gtrsim \left(N - e^{-\Omega(\ell)}\right)^s. \tag{344}$$

Mathematically, we prove the following corollary by using the same analyses as in the proof for Proposition 16:

Lemma 21. *Let us consider the same setup as that in Proposition 16. For an arbitrary subset X, the moment function $M_X^{(1)}(\tau)$ is lower-bounded by*

$$\hat{n}_X(\tau) \succeq \hat{n}_{X[-\ell]} - c_{\tau,1}\lambda_{1/4}e^{-3\ell/16}\hat{\mathscr{D}}_X - 5c_{\tau,1}\lambda_{1/2}e^{-f_\tau \ell}\hat{n}_X - c_{\tau,2}, \tag{345}$$

where $\hat{\mathscr{D}}_X$ and f_τ have been defined in Eqs. (242) and (243), respectively. Also, for moment functions $M_X^{(s)}(\tau)$ with arbitrary orders, the inequality (186) yields

$$M_X^{(s)}(\tau) \ge \left(\hat{n}_{X[-\ell]} - c_{\tau,1}\lambda_{1/4}e^{-3\ell/16}\hat{\mathscr{D}}_X - 5c_{\tau,1}\lambda_{1/2}e^{-f_\tau \ell}\hat{n}_X - c_{\tau,2}\right)^s. \tag{346}$$

Remark. To derive the inequality (344), we consider the moment function of $M_{X[\ell]}^{(s)}(\tau)$ and apply the inequality (346), which yields

$$\begin{aligned} M_{X[\ell]}^{(s)}(\tau) &\ge \left(\langle\hat{n}_X\rangle_\rho - c_{\tau,1}\lambda_{1/4}e^{-3\ell/16}\left\langle\hat{\mathscr{D}}_X\right\rangle_\rho - 5c_{\tau,1}\lambda_{1/2}e^{-f_\tau\ell}\langle\hat{n}_X\rangle_\rho - c_{\tau,2}\right)^s \\ &\ge \left(\langle\hat{n}_X\rangle_\rho - e^{-\Omega(\ell)} - c_{\tau,2}\right)^s \ge \left(N - e^{-\Omega(\ell)} - c_{\tau,2}\right)^s, \end{aligned} \tag{347}$$

where we assume $\langle\hat{n}_X\rangle_\rho \ge N$ in the last inequality.

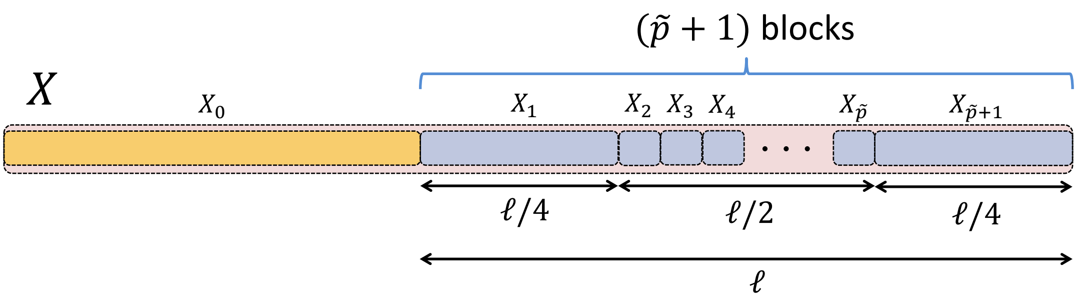

Supplementary Figure 8. Choice of the subsets $\{X_p\}_{p=0}^{\tilde{p}+1}$. Unlike Fig. 4, we define the subsets inside the region X. We can lower-bound the moment function $M_X^{(1)}(\tau)$ by using $M_{X_{0:p}}^{(1)}(\tau)$ because of $X_{0:p} \subseteq X$. Our task is to find the optimal $p \in [1, \tilde{p}]$ which gives the lower bound (345).

1. *Proof of Lemma 21*

As shown in Corollary 12, we have $\mathrm{tr}(\rho(\tau)\hat{n}_X^s) \ge [\mathrm{tr}(\rho(\tau)\hat{n}_X)]^s$ for an arbitrary quantum state ρ, and hence it is enough to consider the 1st order moment in proving the inequality (346) for the sth order moment. In the following, we aim to derive a lower bound for the moment function $M_X^{(1)}(\tau) = \mathrm{tr}[\rho(\tau)\hat{n}_X]$ for an arbitrary ρ.

We first define the subsets $\{X_p\}_{p=0}^{\tilde{p}+1}$ as follows (see Fig. 8):

$$\begin{aligned} &X_0 := X[-\ell], \quad X_1 := X_0[\ell/4] \setminus X_0, \\ &X_p := X_0[\ell/4 + p - 1] \setminus X_0[\ell/4 + p - 2] \quad (2 \le p \le \tilde{p}), \\ &X_{\tilde{p}+1} := X_0[\ell] \setminus X_0[3\ell/4], \end{aligned} \tag{348}$$

where $\tilde{p} = \ell/2 + 1$. We then obtain

$$M_X^{(1)}(\tau) \ge M_{X_{0:p}}^{(1)}(\tau) \tag{349}$$

for arbitrary $p \ge 0$, where we use $\hat{n}_X \succeq \hat{n}_{X_{0:p}}$ from $X \supseteq X_{0:p}$.

We start from a similar inequality to (247):

$$M_{X_{0:p}}^{(1)}(\tau) \ge N_{X_{0:p}}^{(\rho)} - \tilde{N}_{X_{0:p}}^{(\rho)} - c_{\tau,2}, \quad \tilde{N}_{X_{0:p}}^{(\rho)} := \sum_{i\in\partial(X_{0:p})} \sum_{j\in\Lambda} e^{-d_{i,j}} N_j^{(\rho)}, \tag{350}$$

where we have denoted $\langle \hat{n}_i \rangle_\rho$ by $N_i^{(\rho)}$ for $\forall i \in \Lambda$. Then, we aim to lower-bound

$$\max_{p\in[1,\tilde{p}]} \left(N_{X_{0:p}}^{(\rho)} - c_{\tau,1} \tilde{N}_{X_{0:p}}^{(\rho)} \right), \tag{351}$$

which yields the lower bound for $M_X^{(1)}(\tau)$ because of the inequality (349).

By using Lemma 17, we reduce the maximization problem (351) to the form of

$$\begin{aligned} &\max_{p\in[1,\tilde{p}]} \left(N_{X_{0:p}}^{(\rho)} - c_{\tau,1} \tilde{N}_{X_{0:p}}^{(\rho)} \right) \ge N_{X_0}^{(\rho)} - c_{\tau,1} \delta N_{X_0,\ell}^{(\rho)} + \max_{p\in[1,\tilde{p}]} \left(M_{1:p} - c_{\tau,1} \lambda_{1/2} \sum_{j=1}^{\tilde{p}} e^{-|p-j|/2} M_j \right), \\ &M_j = \begin{cases} N_{X_j}^{(\rho)} & \text{for} \quad 1 \le j \le \tilde{p} - 1, \\ N_{X_{\tilde{p}}}^{(\rho)} + N_{X_{\tilde{p}+1}}^{(\rho)} & \text{for} \quad j = \tilde{p}, \end{cases} \end{aligned} \tag{352}$$

where we use $N_{X_{0:p}}^{(\rho)} := N_{X_0}^{(\rho)} + M_{1:p}$ and $\delta N_{X,\ell}^{(\rho)}$ has been defined in Eq. (250), i.e.,

$$\delta N_{X_0,\ell}^{(\rho)} = \lambda_{1/4} e^{-3\ell/16} \mathrm{tr}\left[\rho \left(\hat{n}_{X_0} + \hat{\mathscr{D}}_{X_0[\ell]} \right) \right] \tag{353}$$

with $\hat{\mathscr{D}}_{X_0[\ell]}$ given by Eq. (242). Then, by using Lemma 14 with $\bar{\alpha}$ and χ_α as in Eqs. (252) and (253), we obtain

$$\begin{aligned} \max_{p\in[1,\tilde{p}]} \left(M_{1:p} - c_{\tau,1} \lambda_{1/2} \sum_{j=1}^{\tilde{p}} e^{-|p-j|/2} M_j \right) &\ge -5 c_{\tau,1} \lambda_{1/2} \left(1 + \frac{1}{5 c_{\tau,1} \lambda_{1/2} + 2} \right)^{-\tilde{p}+1} M_{1:\tilde{p}} \\ &= -5 c_{\tau,1} \lambda_{1/2} \left(1 + \frac{1}{5 c_{\tau,1} \lambda_{1/2} + 2} \right)^{-\ell/2} \left(N_X^{(\rho)} - N_{X_0}^{(\rho)} \right), \end{aligned} \tag{354}$$

where we have used $\tilde{p} = \ell/2 + 1$ and $M_{1:\tilde{p}} = N_X^{(\rho)} - N_{X_0}^{(\rho)}$. By combining the inequalities (352) and (354), we obtain

$$
\begin{aligned}
&\max_{p\in[1,\tilde{p}]} \left(N_{X_{0:p}}^{(\rho)} - c_{\tau,1}\tilde{N}_{X_{0:p}}^{(\rho)}\right) \\
&\geq N_{X_0}^{(\rho)} - c_{\tau,1}\lambda_{1/4}e^{-3\ell/16}\left[N_{X_0}^{(\rho)} + \mathrm{tr}\left(\rho\hat{\mathscr{D}}_{X_0[\ell]}\right)\right] - 5c_{\tau,1}\lambda_{1/2}\left(1+\frac{1}{5c_{\tau,1}\lambda_{1/2}+2}\right)^{-\ell/2}\left(N_X^{(\rho)} - N_{X_0}^{(\rho)}\right), \\
&\geq N_{X_0}^{(\rho)} - c_{\tau,1}\lambda_{1/4}e^{-3\ell/16}\mathrm{tr}\left(\rho\hat{\mathscr{D}}_{X_0[\ell]}\right) - 5c_{\tau,1}\lambda_{1/2}\left(1+\frac{1}{5c_{\tau,1}\lambda_{1/2}+2}\right)^{-\ell/2} N_X^{(\rho)},
\end{aligned}
\tag{355}
$$

which yields the main inequality (345) form $X_0 = X[-\ell]$. This completes the proof. □

B. Bound on the probability distribution

In the previous section, we have proved that

$$
\langle\psi_0(\tau)|\hat{n}_{X[\ell]}^s|\psi_0(\tau)\rangle \gtrsim \left(N - e^{-\Omega(\ell)}\right)^s. \tag{356}
$$

Unfortunately, we cannot utilize it to restrict the boson number distribution as in Eq. (342). For example, if a time-evolved state $|\psi_0(\tau)\rangle$ satisfies

$$
\left\|\Pi_{X[\ell],2N}|\psi_0(\tau)\rangle\right\|^2 = 1/2, \quad \left\|\Pi_{X[\ell],0}|\psi_0(\tau)\rangle\right\|^2 = 1/2, \tag{357}
$$

the inequality (356) holds, but we still have a probability of $\mathcal{O}(1)$ that all the bosons flow outside the region $X[\ell]$. In this sense, it is not enough to consider only the lower bound on the moment function. By refining the analyses, we can prove the following statement:

Proposition 22. *Let ρ be an arbitrary quantum state ρ such that*

$$
\Pi_{\mathcal{S}(X[\ell],1/2,q_*)}\rho = \rho, \quad \Pi_{X,\geq N}\rho = \rho, \tag{358}
$$

where the set $\mathcal{S}(L,\xi,N)$ has been defined in Eq. (37)[*2]*. We then obtain*

$$
\mathrm{tr}\left[\Pi_{X[\ell],\leq N-\delta N}\rho(\tau)\right] \leq \frac{2}{1-e^{-1/(8ec_{\tau,2})}}\exp\left[\frac{-\delta N}{8ec_{\tau,2}} + 3c'_\ell\left(1+c'_\ell D\right)\log^2\left(q_*\gamma|X|\right)\right], \tag{359}
$$

where the length ℓ is given by

$$
\ell = \left\lceil \frac{2}{\tilde{f}_\tau}\log\left(\frac{5c_{\tau,1}\lambda_{1/2}e^{2\tilde{f}_\tau}\gamma(D/\tilde{f}_\tau)^D q_*|X|}{2c_{\tau,2}}\right)\right\rceil = c'_\ell\log\left(q_*\gamma|X|\right), \tag{360}
$$

with

$$
\tilde{f}_\tau := \frac{1}{2}\log\left(1+\frac{1}{10ec_{\tau,1}\lambda_{1/2}+2}\right). \tag{361}
$$

Here, c'_ℓ is a constant of $\mathcal{O}(1)$.

Remark. From the proposition, as long as $\delta N \gtrsim \log^2(q_*)$, we obtain the desired restriction in Eq. (342). Here, the probability distribution obeys the large deviation bound, i.e.,

$$
\mathrm{tr}\left[\Pi_{X[\ell],\leq N-\delta N}\rho(\tau)\right] = e^{-\Theta(N)} \quad \text{for} \quad \delta N = \Theta(N). \tag{362}
$$

We will utilize the inequality (359) in deriving the upper bound (899) hereafter.

[*2] Roughly speaking, the projection $\Pi_{\mathcal{S}(X[\ell],1/2,q_*)}$ implies that there does not exist too many bosons around $X[\ell]$.

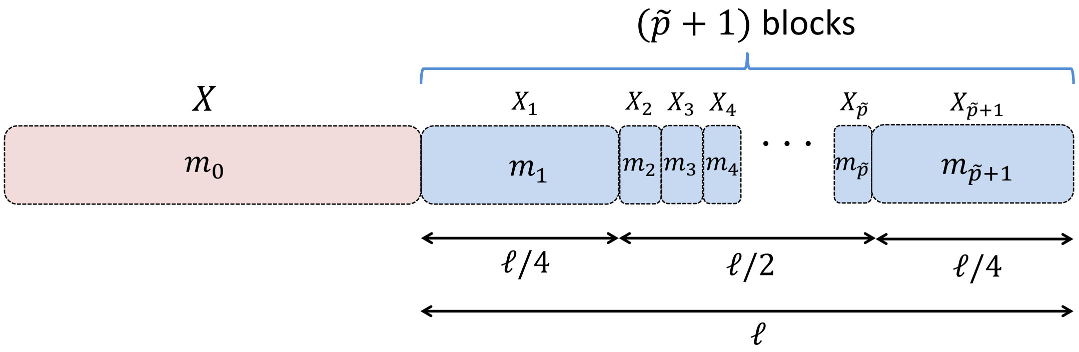

Supplementary Figure 9. We adopt the same notation as in Fig. 4. We decompose the extended subset $X[\ell] \setminus X$ to $(\tilde{p}+1)$ pieces. In the spectral decomposition (367), we assign the boson numbers $\{m_p\}_{p=0}^{\tilde{p}+1}$ in the subsets $\{X_p\}_{p=0}^{\tilde{p}+1}$, respectively. To prove the main inequality (359), we derive the upper bound of (381) and aim to identify the optimal p which minimizes the RHS of (381).

C. Proof of Proposition 22

By using the spectral decomposition of ρ as $\rho = \sum_j p_j |\psi_j\rangle\langle\psi_j|$, we have

$$\mathrm{tr}\left(\Pi_{X[\ell],\le N-\delta N}\rho(\tau)\right) = \sum_j p_j \langle\psi_j(\tau)|\Pi_{X[\ell],\le N-\delta N}|\psi_j(\tau)\rangle = \sum_j p_j \left\|\Pi_{X[\ell],\le N-\delta N}|\psi_j(\tau)\rangle\right\|^2,$$
$$\Pi_{\mathcal{S}(X[\ell],1/2,q_*)}|\psi_j\rangle = |\psi_j\rangle \quad \text{for} \quad \forall j. \tag{363}$$

Hence, we consider the case that ρ is given by a pure state $|\psi\rangle$ without loss of generality. In the following, we choose the length ℓ such that

$$\delta_{X,\ell,q_*} := e^{-\ell/8} q_* |X| \left(\lambda_{1/2} + \gamma\lambda_{1/4}\ell^{D-1}e^{-\ell/16}\right) \le \frac{c_{\tau,2}}{c_{\tau,1}} s, \tag{364}$$

where δ_{X,ℓ,q_*} is going to be defined in Eq. (373).

We adopt the same notation for the subsets $\{X_p\}_{p=1}^{\tilde{p}+1}$ as in Eq. (244):

$$X_0 := X, \quad X_1 := X[\ell/4] \setminus X,$$
$$X_p := X[\ell/4+p-1] \setminus X[\ell/4+p-2] \quad (2 \le p \le \tilde{p}),$$
$$X_{\tilde{p}+1} := X[\ell] \setminus X[3\ell/4], \tag{365}$$

where $\tilde{p} = \ell/2+1$. We here introduce the projection $\Pi_{m_{0:\tilde{p}+1}}$ as

$$\Pi_{m_{0:\tilde{p}+1}} := \Pi_{X,m_0} \prod_{j=1}^{\tilde{p}+1} \Pi_{X_j,m_j}. \tag{366}$$

Note that $\Pi_{X,N}$ has been defined in Eq. (25), i.e., $\hat{n}_X \Pi_{X,N} = N\Pi_{X,N}$. We then obtain

$$\left\|\Pi_{X[\ell],\le N-\delta N}|\psi(\tau)\rangle\right\| = \left\|\sum_{m_0,m_1,\ldots,m_{\tilde{p}+1}} \Pi_{X[\ell],\le N-\delta N} e^{-iHt} \Pi_{m_{0:\tilde{p}+1}}|\psi\rangle\right\|$$
$$\le \sum_{m_0,m_1,\ldots,m_{\tilde{p}+1}} \left\|\Pi_{X[\ell],\le N-\delta N} e^{-iHt}|\psi_{m_{0:\tilde{p}+1}}\rangle\right\|, \tag{367}$$

where we define the unnormalized state $|\psi_{m_{0:\tilde{p}+1}}\rangle := \Pi_{m_{0:\tilde{p}+1}}|\psi\rangle$. From the condition (358), we have $\Pi_{X,m_0}|\psi\rangle = 0$ for $m_0 < N$, and hence in the summation (367) we only have to consider the range of $m_0 \ge N$.

For the estimation of $\left\|\Pi_{X[\ell],\le N-\delta N} e^{-iHt}|\psi_{m_{0:\tilde{p}+1}}\rangle\right\|$, we consider

$$\left\|\Pi_{X[\ell],\le N-\delta N} e^{-iHt}|\psi_{m_{0:\tilde{p}+1}}\rangle\right\| \le \left\|\Pi_{X[\ell],\le N-\delta N}|\hat{n}_{X_{0:p}} - N_p|^{-s/2}\right\| \cdot \left\||\hat{n}_{X_{0:p}} - N_p|^{s/2} e^{-iHt}|\psi_{m_{0:\tilde{p}+1}}\rangle\right\| \tag{368}$$

for an arbitrary choice of $p \in [0, \tilde{p}+1]$, where we choose N_p appropriately such that N_p is larger than N. Because of $X[\ell] \supseteq X_{0:p}$ for $\forall p$, we have $\Pi_{X[\ell],\le N-\delta N} = \Pi_{X[\ell],\le N-\delta N}\Pi_{X_{0:p},\le N-\delta N}$, which yields

$$\left\|\Pi_{X[\ell],\le N-\delta N}|\hat{n}_{X_{0:p}} - N_p|^{-s/2}\right\| \le \frac{1}{|N_p - N + \delta N|^{s/2}}, \tag{369}$$

where we use $N_p \geq N$ to obtain the inequality. Also, by using Proposition 10, we obtain an upper bound of

$$
\begin{aligned}
\left\| |\hat{n}_{X_{0:p}} - N_p|^{s/2} e^{-iHt} |\psi_{m_{0:\tilde{p}+1}}\rangle \right\|^2 &= \langle \psi_{m_{0:\tilde{p}+1}} | e^{iHt} |\hat{n}_{X_{0:p}} - N_p|^s e^{-iHt} |\psi_{m_{0:\tilde{p}+1}}\rangle \\
&\leq \langle \psi_{m_{0:\tilde{p}+1}} | \left(|\hat{n}_{X_{0:p}} - N_p| + c_{\tau,1}\hat{\mathcal{D}}_{X_{0:p}} + c_{\tau,2}s \right)^s |\psi_{m_{0:\tilde{p}+1}}\rangle \\
&= \langle \psi_{m_{0:\tilde{p}+1}} | \left(|m_{0:p} - N_p| + c_{\tau,1}\hat{\mathcal{D}}_{X_{0:p}} + c_{\tau,2}s \right)^s |\psi_{m_{0:\tilde{p}+1}}\rangle \\
&= \langle \psi_{m_{0:\tilde{p}+1}} | \left(c_{\tau,1}\hat{\mathcal{D}}_{X_{0:p}} + c_{\tau,2}s \right)^s |\psi_{m_{0:\tilde{p}+1}}\rangle
\end{aligned} \tag{370}
$$

with $m_{0:p} = \sum_{j=0}^{p} m_j$, where we choose N_p as $N_p = m_{0:p}$ in the last equation. Note that the choice ensures $N_p \geq N$ because of $m_0 \geq N$.

From $\Pi_{\mathcal{S}(X[\ell],1/2,q_*)}\Pi_{m_{0:\tilde{p}+1}}|\psi_{m_{0:\tilde{p}+1}}\rangle = |\psi_{m_{0:\tilde{p}+1}}\rangle$, we then consider

$$
\begin{aligned}
&\langle \psi_{m_{0:\tilde{p}+1}} | \left(c_{\tau,1}\hat{\mathcal{D}}_{X_{0:p}} + c_{\tau,2}s \right)^s |\psi_{m_{0:\tilde{p}+1}}\rangle \\
&= \langle \psi_{m_{0:\tilde{p}+1}} | \Pi_{\mathcal{S}(X[\ell],1/2,q_*)}\Pi_{m_{0:\tilde{p}+1}} \left(c_{\tau,1}\hat{\mathcal{D}}_{X_{0:p}} + c_{\tau,2}s \right)^s \Pi_{\mathcal{S}(X[\ell],1/2,q_*)}\Pi_{m_{0:\tilde{p}+1}} |\psi_{m_{0:\tilde{p}+1}}\rangle \\
&\leq \left\| \Pi_{\mathcal{S}(X[\ell],1/2,q_*)}\Pi_{m_{0:\tilde{p}+1}} \left(c_{\tau,1}\hat{\mathcal{D}}_{X_{0:p}} + c_{\tau,2}s \right)^s \Pi_{\mathcal{S}(X[\ell],1/2,q_*)}\Pi_{m_{0:\tilde{p}+1}} \right\| \\
&= \left(c_{\tau,1} \left\| \Pi_{\mathcal{S}(X[\ell],1/2,q_*)}\Pi_{m_{0:\tilde{p}+1}} \hat{\mathcal{D}}_{X_{0:p}} \Pi_{m_{0:\tilde{p}+1}} \Pi_{\mathcal{S}(X[\ell],1/2,q_*)} \right\| + c_{\tau,2}s \right)^s,
\end{aligned} \tag{371}
$$

where we use the fact that $\hat{\mathcal{D}}_{X_{0:p}}$ commutes with $\Pi_{\mathcal{S}(X[\ell],1/2,q_*)}$ and $\Pi_{m_{0:\tilde{p}+1}}$. By applying the inequalities (369), (370), (371) to (368), we obtain

$$
\left\| \Pi_{X[\ell],\leq N-\delta N} e^{-iHt} |\psi_{m_{0:\tilde{p}+1}}\rangle \right\| \leq \frac{\left(c_{\tau,1} \left\| \Pi_{\mathcal{S}(X[\ell],1/2,q_*)}\Pi_{m_{0:\tilde{p}+1}} \hat{\mathcal{D}}_{X_{0:p}} \Pi_{m_{0:\tilde{p}+1}} \Pi_{\mathcal{S}(X[\ell],1/2,q_*)} \right\| + c_{\tau,2}s \right)^{s/2}}{|N_p - N + \delta N|^{s/2}}. \tag{372}
$$

By using the same analyses as the proof of Lemma 17, we prove the following lemma:

Lemma 23. *Let us define*

$$
\delta_{X,\ell,q_*} := e^{-\ell/8} q_* |X| \left(\lambda_{1/2} + \gamma \lambda_{1/4} \ell^{D-1} e^{-\ell/16} \right), \tag{373}
$$

We then obtain the upper bound of

$$
\left\| \Pi_{\mathcal{S}(X[\ell],1/2,q_*)}\Pi_{m_{0:\tilde{p}+1}} \hat{\mathcal{D}}_{X_{0:p}} \Pi_{m_{0:\tilde{p}+1}} \Pi_{\mathcal{S}(X[\ell],1/2,q_*)} \right\| \leq \lambda_{1/2} \sum_{j=1}^{\tilde{p}} e^{-|p-j|/2} M_j + \delta_{X,\ell,q_*} \quad (1 \leq p \leq \tilde{p}), \tag{374}
$$

with

$$
M_j = \begin{cases} m_j & \text{for} \quad 1 \leq j \leq \tilde{p} - 1, \\ m_{\tilde{p}} + m_{\tilde{p}+1} & \text{for} \quad j = \tilde{p}. \end{cases} \tag{375}
$$

Proof of Lemma 23. From the inequality (258), we can start from

$$
\begin{aligned}
&\left\| \Pi_{\mathcal{S}(X[\ell],1/2,q_*)}\Pi_{m_{0:\tilde{p}+1}} \hat{\mathcal{D}}_{X_{0:p}} \Pi_{m_{0:\tilde{p}+1}} \Pi_{\mathcal{S}(X[\ell],1/2,q_*)} \right\| \\
&\leq \lambda_{1/2} \sum_{j=1}^{\tilde{p}} e^{-|p-j|/2} M_j + \sum_{i \in \partial(X_{0:p})} \sum_{j \in X_{1:\tilde{p}+1}^{\mathrm{c}}} e^{-d_{i,j}} \left\| \hat{n}_j \Pi_{\mathcal{S}(X[\ell],1/2,q_*)} \right\| \\
&\leq \lambda_{1/2} \sum_{j=1}^{\tilde{p}} e^{-|p-j|/2} M_j + q_* \sum_{i \in \partial(X_{0:p})} \sum_{j \in X_{1:\tilde{p}+1}^{\mathrm{c}}} e^{-d_{i,j} + d_{j,X[\ell]}/2} \quad (1 \leq p \leq \tilde{p}),
\end{aligned} \tag{376}
$$

where we use the definition (37) in the last inequality. We then decompose

$$
\sum_{i \in \partial(X_{0:p})} \sum_{j \in X_{1:\tilde{p}+1}^{\mathrm{c}}} e^{-d_{i,j} + d_{j,X[\ell]}/2} = \sum_{i \in \partial(X_{0:p})} \sum_{j \in X} e^{-d_{i,j}} + \sum_{i \in \partial(X_{0:p})} \sum_{j \in X[\ell]^{\mathrm{c}}} e^{-d_{i,j} + d_{j,X[\ell]}/2}, \tag{377}
$$

where we use $d_{j,X[\ell]} = 0$ for $j \in X$. For the first term, we obtain

$$
\sum_{i \in \partial(X_{0:p})} \sum_{j \in X} e^{-d_{i,j}} \leq \sum_{j \in X} \sum_{i : d_{i,j} \geq \ell/4} e^{-d_{i,j}} \leq e^{-\ell/8} |X| \sum_{i \in \Lambda} e^{-d_{i,j}/2} \leq \lambda_{1/2} e^{-\ell/8} |X|, \tag{378}
$$

where, in the first inequality, we use $d_{i,j} \geq \ell/4$ for $i \in \partial(X_{0:p})$ and $j \in X$ (see Fig. 9), and in the third inequality, we apply the inequality (12). Also, for the second term in (377), we use Eq. (37) to obtain

$$\begin{aligned}\sum_{i\in\partial(X_{0:p})}\sum_{j\in X[\ell]^{\rm c}} e^{-d_{i,j}+d_{j,X[\ell]}/2} &\leq e^{-\ell/8}\sum_{i\in\partial(X_{0:p})}\sum_{j\in X[\ell]^{\rm c}} e^{-d_{i,j}/2} \\ &\leq e^{-\ell/8}\sum_{i\in\partial(X_{0:p})}\sum_{j:d_{i,j}\geq \ell/4} e^{-d_{i,j}/2} \\ &\leq \lambda_{1/4}e^{-3\ell/16}|\partial(X_{0:p})| \leq \gamma\lambda_{1/4}|X|\ell^{D-1}e^{-3\ell/16},\end{aligned} \tag{379}$$

where we use $d_{j,X[\ell]} \leq d_{i,j} - \ell/4$ for $i \in \partial(X_{0:p})$ and $j \in X[\ell]^{\rm c}$ in the first inequality, and in the last inequality, we use $|\partial(X_{0:p})| \leq |\partial X[\ell]| \leq \gamma\ell^{D-1}|X|$ from (7). By applying the inequalities (378) and (379) to (376) and (377), we have

$$q_* \sum_{i\in\partial(X_{0:p})}\sum_{j\in X^{\rm c}_{1:\tilde{p}+1}} e^{-d_{i,j}+d_{j,X[\ell]}/2} \leq e^{-\ell/8}q_*|X|\left(\lambda_{1/2}+\gamma\lambda_{1/4}\ell^{D-1}e^{-\ell/16}\right) = \delta_{X,\ell,q_*}, \tag{380}$$

where the last equation is the definition (373) for δ_{X,ℓ,q_*}. By combining the inequalities (376) and (380), we prove the main inequality (374). This completes the proof. □

———————————————————————————————[**End of Proof of Lemma 23**]

By combining the inequalities (372) and (374), we have

$$\begin{aligned}\left\|\Pi_{X[\ell],\leq N-\delta N}e^{-iHt}|\psi_{m_{0:\tilde{p}+1}}\rangle\right\| &\leq \frac{1}{|m_{0:p}-N+\delta N|^{s/2}}\left(c_{\tau,1}\lambda_{1/2}\sum_{j=1}^{\tilde{p}} e^{-|p-j|/2}M_j + c_{\tau,1}\delta_{X,\ell,q_*} + c_{\tau,2}s\right)^{s/2} \\ &= \left(\frac{\tilde{M}_p + 2c_{\tau,2}s}{M_{1:p}+m_0-N+\delta N}\right)^{s/2} \quad \text{for} \quad \forall p\in[1,\tilde{p}-1],\end{aligned} \tag{381}$$

where we define $M_{1:p} = \sum_{j=1}^{p} M_p$ and $\tilde{M}_p := c_{\tau,1}\lambda_{1/2}\sum_{j=1}^{\tilde{p}} e^{-|p-j|/2}M_j$. Note that $c_{\tau,1}\delta_{X,\ell,q_*} \leq c_{\tau,2}s$ from the condition (364).

Then, we aim to find the optimal p which minimizes the RHS of the inequality (381). We here prove the following lemma:

Lemma 24. *If the length ℓ satisfies*

$$\ell \geq \frac{2}{\tilde{f}_\tau}\log\left(\frac{5c_{\tau,1}\lambda_{1/2}e^{2\tilde{f}_\tau}\gamma(D/\tilde{f}_\tau)^D q_*|X|}{2c_{\tau,2}}\right) \tag{382}$$

with

$$\tilde{f}_\tau := \frac{1}{2}\log\left(1+\frac{1}{10ec_{\tau,1}\lambda_{1/2}+2}\right), \tag{383}$$

we reduce the inequality (381) to the form of

$$\left\|\Pi_{X[\ell],\leq N-\delta N}e^{-iHt}|\psi_{m_{0:\tilde{p}+1}}\rangle\right\| \leq 2\exp\left(\frac{-m_0+N-\delta N}{8ec_{\tau,2}}\right) \tag{384}$$

by choosing $p \in [1,\tilde{p}-1]$ appropriately.

1. *Proof of Lemma 24*

We here consider the following two cases:

$$\min_{p\in[1:\tilde{p}-1]}\left(\frac{\tilde{M}_p}{M_{1:p}}\right)\begin{cases}\leq \frac{1}{2e} & \text{for} \quad \text{Case 1}, \\ \geq \frac{1}{2e} & \text{for} \quad \text{Case 2}.\end{cases} \tag{385}$$

[Case 1]

In that case, we can find p_* such that

$$\frac{\tilde{M}_{p_*}}{M_{1:p_*}} \leq \frac{1}{2e}. \tag{386}$$

By using the inequality of

$$\frac{x'+y'}{x+y} = \frac{x'}{x+y} + \frac{y'}{x+y} \le \frac{x'}{x} + \frac{y'}{y} \tag{387}$$

for arbitrary positive x, y, x', y', we have

$$\frac{\tilde{M}_{p_*} + 2c_{\tau,2}s}{M_{1:p_*} + m_0 - N + \delta N} \le \frac{\tilde{M}_{p_*}}{M_{1:p_*}} + \frac{2c_{\tau,2}s}{m_0 - N + \delta N} \le \frac{1}{2e} + \frac{2c_{\tau,2}s}{m_0 - N + \delta N}. \tag{388}$$

Therefore, we choose s as

$$s = \left\lfloor \frac{m_0 - N + \delta N}{4ec_{\tau,2}} \right\rfloor, \tag{389}$$

which reduces the inequality (388) to

$$\frac{\tilde{M}_{p_*} + 2c_{\tau,2}s}{M_{1:p_*} + m_0 - N + \delta N} \le \frac{1}{e}. \tag{390}$$

By applying the above inequality to (381), we obtain the desired inequality of

$$\left\| \Pi_{X[\ell], \le N - \delta N} e^{-iHt} |\psi_{m_{0:\tilde{p}+1}}\rangle \right\| \le 2 \exp\left(\frac{-m_0 + N - \delta N}{8ec_{\tau,2}} \right), \tag{391}$$

where we use $e^{1/2} < 2$.

[Case 2]

In this case, we can utilize Lemma 15 with $\Phi = 2e$ to obtain

$$\begin{aligned} \tilde{M}_1 &\le \left(1 + \frac{1}{2e\bar{\alpha}_\infty + \chi_\alpha}\right)^{-\tilde{p}+2} \bar{\alpha}_\infty M_{1:\tilde{p}-1} \\ &\le \left(1 + \frac{1}{10ec_{\tau,1}\lambda_{1/2} + 2}\right)^{-\tilde{p}+2} 5c_{\tau,1}\lambda_{1/2} M_{1:\tilde{p}-1} = 5c_{\tau,1}\lambda_{1/2} e^{-\tilde{f}_\tau (2\tilde{p}-4)} M_{1:\tilde{p}-1} \end{aligned} \tag{392}$$

with $\tilde{f}_\tau$ defined in Eq. (383), where the parameters $\bar{\alpha}_\infty$ and χ_α have been estimated as in Eqs. (252) and (253): $\bar{\alpha}_\infty = (4.0829\cdots)c_{\tau,1}\lambda_{1/2}$ and $\chi_\alpha = 1.5414\cdots$. From $\tilde{p} = \ell/2 + 1$ and the upper bound of

$$M_{1:\tilde{p}-1} \le M_{0:\tilde{p}} \le \left\| \Pi_{\mathcal{S}(X[\ell],1/2,q_*)} \hat{n}_{X[\ell]} \right\| \le q_* |X[\ell]| \le \gamma \ell^D q_* |X|, \tag{393}$$

we reduce the inequality (392) to

$$\tilde{M}_1 \le 5c_{\tau,1}\lambda_{1/2} e^{2\tilde{f}_\tau} \gamma \ell^D q_* |X| e^{-\tilde{f}_\tau \ell} \le 5c_{\tau,1}\lambda_{1/2} e^{2\tilde{f}_\tau} \gamma (D/\tilde{f}_\tau)^D q_* |X| e^{-\tilde{f}_\tau \ell/2}, \tag{394}$$

where we use the inequality of $x^z e^{-x} \le z^z e^{-x/2}$ for $z > 0$. Therefore, by choosing

$$e^{\tilde{f}_\tau \ell/2} \ge \frac{5c_{\tau,1}\lambda_{1/2} e^{2\tilde{f}_\tau} \gamma (D/\tilde{f}_\tau)^D q_* |X|}{2c_{\tau,2}} \longrightarrow \ell \ge \frac{2}{\tilde{f}_\tau} \log\left(\frac{5c_{\tau,1}\lambda_{1/2} e^{2\tilde{f}_\tau} \gamma (D/\tilde{f}_\tau)^D q_* |X|}{2c_{\tau,2}} \right), \tag{395}$$

we have $\tilde{M}_1 \le 2c_{\tau,2} \le 2c_{\tau,2}s$ from the inequality (394).

Then, for $p = 1$, the inequality (381) reduces to

$$\left\| \Pi_{X[\ell], \le N - \delta N} e^{-iHt} |\psi_{m_{0:\tilde{p}+1}}\rangle \right\| \le \left(\frac{\tilde{M}_1 + 2c_{\tau,2}s}{M_1 + m_0 - N + \delta N} \right)^{s/2} \le \left(\frac{4c_{\tau,2}s}{m_0 - N + \delta N} \right)^{s/2}. \tag{396}$$

By choosing

$$s = \left\lfloor \frac{m_0 - N + \delta N}{4ec_{\tau,2}} \right\rfloor, \tag{397}$$

we reduce the inequality (396) to

$$\left\| \Pi_{X[\ell], \le N - \delta N} e^{-iHt} |\psi_{m_{0:\tilde{p}+1}}\rangle \right\| \le 2 \exp\left(\frac{-m_0 + N - \delta N}{8ec_{\tau,2}} \right). \tag{398}$$

From the inequalities (391) and (398), we prove the main inequality (384). This completes the proof. □

[**End of Proof of Lemma 24**]

By combining the inequalities (367) and (384), we obtain

$$\left\|\Pi_{X[\ell],\le N-\delta N}|\psi(\tau)\rangle\right\| \le \sum_{m_0,m_1,\ldots,m_{\tilde{p}+1}} 2\exp\left(\frac{-m_0+N-\delta N}{8ec_{\tau,2}}\right). \tag{399}$$

Because of $\Pi_{\mathcal{S}(X[\ell],1/2,q_*)}|\psi\rangle = |\psi\rangle$, the summation for m_p is taken from $m_p = 0$ to

$$m_p = \left\|\Pi_{\mathcal{S}(X[\ell],1/2,q_*)}\hat{n}_{X_p}\right\| \le q_*|X_p| \le q_*\gamma\ell^D|X|, \tag{400}$$

where we use $|X_p| \le |X[\ell]| \le \gamma\ell^D|X|$. We recall that m_p is defined as the number of bosons in the region X_p as in Eq. (366). Also, the summation with respect to m_0 is taken from $m_0 = N$, and hence, we have

$$\sum_{m_0=N}^{\infty} \exp\left(\frac{-m_0+N-\delta N}{8ec_{\tau,2}}\right) \le \frac{1}{1-e^{-1/(8ec_{\tau,2})}}\exp\left(\frac{-\delta N}{8ec_{\tau,2}}\right). \tag{401}$$

Therefore, the summation in (399) is upper-bounded by

$$\begin{aligned}\left\|\Pi_{X[\ell],\le N-\delta N}|\psi(\tau)\rangle\right\| &\le \frac{2\left(q_*\gamma\ell^D|X|\right)^{\tilde{p}+1}}{1-e^{-1/(8ec_{\tau,2})}}\exp\left(\frac{-\delta N}{8ec_{\tau,2}}\right) \\ &= \frac{2}{1-e^{-1/(8ec_{\tau,2})}}\exp\left[\frac{-\delta N}{8ec_{\tau,2}} + \frac{\ell+4}{2}\log\left(q_*\gamma\ell^D|X|\right)\right],\end{aligned} \tag{402}$$

where we use $\tilde{p} = \ell/2+1$. Under condition (382), we can choose ℓ such that

$$\ell = \left\lceil \frac{2}{\tilde{f}_\tau}\log\left(\frac{5c_{\tau,1}\lambda_{1/2}e^{2\tilde{f}_\tau}\gamma(D/\tilde{f}_\tau)^D q_*|X|}{2c_{\tau,2}}\right)\right\rceil = c'_\ell \log\left(q_*\gamma|X|\right), \tag{403}$$

where c'_ℓ is a constant of $\mathcal{O}(1)$. Then, we obtain an upper bound for

$$\begin{aligned}\frac{\ell+4}{2}\log\left(q_*\gamma\ell^D|X|\right) &= \frac{\ell+4}{2}\left[\log\left(q_*\gamma|X|\right) + D\log(\ell)\right] = \frac{\ell+4}{2}\left(\frac{\ell}{c'_\ell} + D\log(\ell)\right) \\ &\le 3\left(\frac{1}{c'_\ell}+D\right)\ell^2 = 3c'_\ell\left(1+c'_\ell D\right)\log^2\left(q_*\gamma|X|\right),\end{aligned} \tag{404}$$

where in the inequality, we use $\ell \ge 1$, $\ell/2+2 \le 5\ell/2 \le 3\ell$, and $\log(\ell) \le \ell$. By combining the inequalities (402) and (404), we obtain the main inequality (359). This completes the proof. □

Supplementary Note 6. EFFECTIVE HAMILTONIAN THEORY

In this section, we consider an effective Hamiltonian in the form of

$$\bar{\Pi}H\bar{\Pi}, \tag{405}$$

where $\bar{\Pi}$ is an arbitrary projection such that $[\bar{\Pi}, \hat{n}_i] = 0$ for $\forall i \in \Lambda$. Then, we often encounter a problem to estimate an error between the time evolutions $e^{-i\bar{\Pi}H\bar{\Pi}t}$ and e^{-iHt}. Usually, we cannot ensure

$$\left\|e^{-i\bar{\Pi}H\bar{\Pi}t} - e^{-iHt}\right\| \tag{406}$$

in the level of the operator norm. If we consider specific quantum state $|\psi\rangle$ and operator O, we may be able to prove

$$\left\|O(H,\tau)|\psi\rangle - O(\bar{\Pi}H\bar{\Pi},\tau)|\psi\rangle\right\| \approx 0. \tag{407}$$

In general, we can prove the following lemma which upper-bounds the error using the effective Hamiltonian.

Lemma 25 (Generalization of Lemma 13 in Ref. [1]). *Let $|\psi\rangle$ and O be a quantum state and an operator of interest, respectively. Then, for arbitrary projection $\bar{\Pi}$ and τ, we obtain*

$$\begin{aligned}\left\|O(H,\tau)|\psi\rangle - O(\bar{\Pi}H\bar{\Pi},\tau)|\psi\rangle\right\| &\le \left\|\bar{\Pi}^{\rm c}e^{iH\tau}Oe^{-iH\tau}|\psi\rangle\right\| + \left\|\bar{\Pi}^{\rm c}Oe^{-iH\tau}|\psi\rangle\right\| + \left\|\bar{\Pi}^{\rm c}|\psi\rangle\right\| + \left\|\bar{\Pi}^{\rm c}e^{-iH\tau}|\psi\rangle\right\| \\ &+ \int_0^\tau \left\|\bar{\Pi}H_0\bar{\Pi}^{\rm c}e^{iH(\tau-\tau_1)}Oe^{-iH\tau}|\psi\rangle\right\| d\tau_1 + \int_0^\tau \left\|\bar{\Pi}H_0\bar{\Pi}^{\rm c}e^{-iH\tau_1}|\psi\rangle\right\| d\tau_1\end{aligned} \tag{408}$$

with $\bar{\Pi}^{\rm c} := 1-\bar{\Pi}$, where we set $\|O\| = 1$.

We can easily extend the statement to a mixed state ρ:

Corollary 26. *Let ρ be an arbitrary quantum state. Then, we generalize the inequality (408) to*

$$\left\|O(H,\tau)\rho - O(\bar{\Pi}H\bar{\Pi},\tau)\rho\right\|_1 \le \left\|\bar{\Pi}^{\rm c}O(\tau)\sqrt{\rho}\right\|_F + \left\|\bar{\Pi}^{\rm c}O\sqrt{\rho(\tau)}\right\|_F + \left\|\bar{\Pi}^{\rm c}\sqrt{\rho}\right\|_F + \left\|\bar{\Pi}^{\rm c}\sqrt{\rho(\tau)}\right\|_F$$
$$+ \int_0^\tau \left\|\bar{\Pi}H_0\bar{\Pi}^{\rm c}O(\tau-\tau_1)\sqrt{\rho(\tau_1)}\right\|_F d\tau_1 + \int_0^\tau \left\|\bar{\Pi}H_0\bar{\Pi}^{\rm c}\sqrt{\rho(\tau_1)}\right\|_F d\tau_1, \tag{409}$$

where $\|\cdots\|_F$ is the Frobenius norm, i.e., $\|O\|_F = \sqrt{{\rm tr}(OO^\dagger)}$.

Proof of Corollary 26. Let us decompose the state ρ as

$$\rho = \sum_i p_i |\psi_i\rangle\langle\psi_i|. \tag{410}$$

Then, for an arbitrary operator $\tilde{O}$, we have

$$\left\|\tilde{O}|\psi_i\rangle\langle\psi_i|\right\|_1 = {\rm tr}\left(\sqrt{\tilde{O}|\psi_i\rangle\langle\psi_i|\tilde{O}^\dagger}\right) = \sqrt{\langle\psi_i|\tilde{O}^\dagger\tilde{O}|\psi_i\rangle} = \left\|\tilde{O}|\psi_i\rangle\right\|,$$

and hence

$$\left\|O(H,\tau)\rho - O(\bar{\Pi}H\bar{\Pi},\tau)\rho\right\|_1 \le \sum_i p_i\Big(\left\|\bar{\Pi}^{\rm c}e^{iH\tau}Oe^{-iH\tau}|\psi_i\rangle\right\| + \left\|\bar{\Pi}^{\rm c}Oe^{-iH\tau}|\psi_i\rangle\right\| + \left\|\bar{\Pi}^{\rm c}|\psi_i\rangle\right\| + \left\|\bar{\Pi}^{\rm c}e^{-iH\tau}|\psi_i\rangle\right\|$$
$$+ \int_0^\tau \left\|\bar{\Pi}H_0\bar{\Pi}^{\rm c}e^{iH(\tau-\tau_1)}Oe^{-iH\tau}|\psi_i\rangle\right\| d\tau_1 + \int_0^\tau \left\|\bar{\Pi}H_0\bar{\Pi}^{\rm c}e^{-iH\tau_1}|\psi_i\rangle\right\| d\tau_1\Big). \tag{411}$$

Moreover, for an arbitrary operator $\tilde{O}$, we prove

$$\sum_i p_i \left\|\tilde{O}|\psi_i\rangle\right\| = \sum_i p_i \sqrt{\langle\psi_i|\tilde{O}^\dagger\tilde{O}|\psi_i\rangle} \le \sqrt{\sum_i p_i\langle\psi_i|\tilde{O}^\dagger\tilde{O}|\psi_i\rangle} = \sqrt{{\rm tr}\left(\tilde{O}\rho\tilde{O}^\dagger\right)} = \left\|\tilde{O}\sqrt{\rho}\right\|_F. \tag{412}$$

By combining the above two inequalities, we derive the desired inequality (409). This completes the proof. □

As another extension, we can also generalize the results to the time-dependent Hamiltonian H_t, which is utilized in proving Lemma 46 below. Here, we utilize the notations in Eq. (33), i.e., $U_{A_t,\tau_1\to\tau_2} = \mathcal{T}e^{-i\int_{\tau_1}^{\tau_2}A_t dt}$ and $O(A_t,\tau_1\to\tau_2) = U^\dagger_{A_t,\tau_1\to\tau_2}OU_{A_t,\tau_1\to\tau_2}$.

Corollary 27. *Let us assume that H_t satisfies $\|dH_t/dt\| < \infty$. For simplicity, let us denote*

$$U_{\tau'} = U_{H_t,0\to\tau'} \tag{413}$$

for an arbitrary τ'. We then obtain

$$\left\|O(H_t,0\to\tau)\rho - O(\bar{\Pi}H_t\bar{\Pi},0\to\tau)\rho\right\|_1 \le \left\|\bar{\Pi}^{\rm c}U_\tau^\dagger OU_\tau\sqrt{\rho}\right\|_F + \left\|\bar{\Pi}^{\rm c}OU_\tau\sqrt{\rho}\right\|_F + \left\|\bar{\Pi}^{\rm c}\sqrt{\rho}\right\|_F + \left\|\bar{\Pi}^{\rm c}U_\tau\sqrt{\rho}\right\|_F$$
$$+ \int_0^\tau \left\|\bar{\Pi}H_{\tau-\tau_1}\bar{\Pi}^{\rm c}U_{\tau_1}U_\tau^\dagger OU_\tau\sqrt{\rho}\right\|_F d\tau_1 + \int_0^\tau \left\|\bar{\Pi}H_{\tau-\tau_1}\bar{\Pi}^{\rm c}U_{\tau_1}\sqrt{\rho}\right\|_F d\tau_1. \tag{414}$$

The proof is exactly the same as for Lemma 25.

1. *Proof of Lemma 25*

We take the same approach as Ref. [1, Supplementary Lemma 13]. For the convenience of the readers, we show the full proof of this lemma. Throughout the proof, we denote

$$\bar{\Pi}^{\rm c} = 1 - \bar{\Pi}, \quad \tilde{H} = \bar{\Pi}H\bar{\Pi} \tag{415}$$

for simplicity. We start from the inequality as

$$\begin{aligned}
&\left\|O(H,\tau)|\psi\rangle - O(\tilde{H},\tau)|\psi\rangle\right\| \\
&\le \left\|e^{iH\tau}Oe^{-iH\tau}|\psi\rangle - e^{i\tilde{H}\tau}Oe^{-iH\tau}|\psi\rangle\right\| + \left\|e^{i\tilde{H}\tau}O(e^{-iH\tau} - e^{-i\tilde{H}\tau})|\psi\rangle\right\| \\
&\le \left\|e^{iH\tau}Oe^{-iH\tau}|\psi\rangle - e^{i\tilde{H}\tau}Oe^{-iH\tau}|\psi\rangle\right\| + \left\||\psi\rangle - e^{i\tilde{H}\tau}e^{-iH\tau}|\psi\rangle\right\|,
\end{aligned} \tag{416}$$

where the first inequality is derived from the triangle inequality, and we use $\|O\| = 1$ in the second inequality. In order to estimate the RHS in the inequality (416), we need to upper-bound

$$\left\|e^{iH\tau}Oe^{-iH\tau}|\psi\rangle - e^{i\tilde{H}\tau}Oe^{-iH\tau}|\psi\rangle\right\| \tag{417}$$

for an arbitrary O. We note that by choosing $O = \hat{1}$ the second term in the RHS of (416) is also given by the form of (417). For the estimation of (417), we begin with the inequality of

$$\begin{aligned}
&\left\|e^{iH\tau}Oe^{-iH\tau}|\psi\rangle - e^{i\tilde{H}\tau}Oe^{-iH\tau}|\psi\rangle\right\| \\
\le &\left\|\bar{\Pi}^{\mathrm{c}}\left(e^{iH\tau}Oe^{-iH\tau}|\psi\rangle - e^{i\tilde{H}\tau}Oe^{-iH\tau}|\psi\rangle\right)\right\| + \left\|\bar{\Pi}\left(e^{iH\tau}Oe^{-iH\tau}|\psi\rangle - e^{i\tilde{H}\tau}Oe^{-iH\tau}|\psi\rangle\right)\right\| \\
= &\left\|\bar{\Pi}^{\mathrm{c}}e^{iH\tau}Oe^{-iH\tau}|\psi\rangle\right\| + \left\|\bar{\Pi}^{\mathrm{c}}Oe^{-iH\tau}|\psi\rangle\right\| + \left\|\bar{\Pi}\left(e^{iH\tau}O - e^{i\tilde{H}\tau}O\right)e^{-iH\tau}|\psi\rangle\right\|,
\end{aligned} \tag{418}$$

where, in the equation, we use

$$\left\|\bar{\Pi}^{\mathrm{c}}e^{i\tilde{H}\tau}Oe^{-iH\tau}|\psi\rangle\right\| = \left\|\bar{\Pi}^{\mathrm{c}}Oe^{-iH\tau}|\psi\rangle\right\| \tag{419}$$

because of $\bar{\Pi}^{\mathrm{c}}\tilde{H} = \bar{\Pi}^{\mathrm{c}}\bar{\Pi}H\bar{\Pi} = 0$.

In order to upper-bound the third term in the RHS of (418), let us decompose the time to infinitely many pieces (i.e., $\tau = m_0 d\tau$ and $m_0 \to \infty$). We then estimate the norm $\left\|\bar{\Pi}\left(e^{iH\tau}O - e^{i\tilde{H}\tau}O\right)e^{-iH\tau}|\psi\rangle\right\|$ by using the following equation:

$$\begin{aligned}
e^{iH\tau}O - e^{i\tilde{H}\tau}O &= \sum_{m=0}^{m_0-1}\left(e^{i\tilde{H}md\tau}e^{iH(m_0-m)d\tau}O - e^{i\tilde{H}(m+1)d\tau}e^{iH(m_0-1-m)d\tau}O\right) \\
&= \sum_{m=0}^{m_0-1}\left(e^{i\tilde{H}md\tau}e^{iH(\tau-md\tau)}O - e^{i\tilde{H}(md\tau+d\tau)}e^{iH(\tau-md\tau-d\tau)}O\right) \\
&= -i\int_0^\tau e^{i\tilde{H}\tau_1}\left(\tilde{H} - H\right)e^{iH(\tau-\tau_1)}Od\tau_1
\end{aligned} \tag{420}$$

in the limit of $m_0 \to \infty$, where, in the last equation, we use

$$\begin{aligned}
&e^{i\tilde{H}md\tau}e^{iH(\tau-md\tau)}O - e^{i\tilde{H}(md\tau+d\tau)}e^{iH(\tau-md\tau-d\tau)}O \\
= &-ie^{i\tilde{H}md\tau}\left(\tilde{H} - H\right)e^{iH(\tau-md\tau)}Od\tau + \mathcal{O}(d\tau^2).
\end{aligned} \tag{421}$$

We consider the decomposition of

$$\bar{\Pi}\left(\tilde{H} - H\right) = \bar{\Pi}H\bar{\Pi} - \bar{\Pi}H\left(\bar{\Pi} + 1 - \bar{\Pi}\right) = -\bar{\Pi}H\bar{\Pi}^{\mathrm{c}} = -\bar{\Pi}H_0\bar{\Pi}^{\mathrm{c}}, \tag{422}$$

where we use $[\bar{\Pi}, \tilde{H}] = 0$ and $[V, \bar{\Pi}] = 0$ which is derived from $[\hat{n}_i, \bar{\Pi}] = 0$ for $\forall i \in \Lambda$. We recall that $\bar{\Pi}^{\mathrm{c}}$ has been defined as $\bar{\Pi}^{\mathrm{c}} := 1 - \bar{\Pi}$. Therefore, from Eq. (420), we obtain

$$\begin{aligned}
\left\|\bar{\Pi}\left(e^{iH\tau}O - e^{i\tilde{H}\tau}O\right)e^{-iH\tau}|\psi\rangle\right\| &= \left\|i\int_0^\tau e^{i\tilde{H}\tau_1}\bar{\Pi}H_0\bar{\Pi}^{\mathrm{c}}e^{iH(\tau-\tau_1)}d\tau_1 \cdot Oe^{-iH\tau}|\psi\rangle\right\| \\
&\le \int_0^\tau \left\|\bar{\Pi}H_0\bar{\Pi}^{\mathrm{c}}e^{iH(\tau-\tau_1)}Oe^{-iH\tau}|\psi\rangle\right\| d\tau_1,
\end{aligned} \tag{423}$$

which reduces the inequality (418) to

$$\begin{aligned}
&\left\|e^{iH\tau}Oe^{-iH\tau}|\psi\rangle - e^{i\tilde{H}\tau}Oe^{-iH\tau}|\psi\rangle\right\| \\
\le &\left\|\bar{\Pi}^{\mathrm{c}}e^{iH\tau}Oe^{-iH\tau}|\psi\rangle\right\| + \left\|\bar{\Pi}^{\mathrm{c}}Oe^{-iH\tau}|\psi\rangle\right\| + \int_0^\tau \left\|\bar{\Pi}H_0\bar{\Pi}^{\mathrm{c}}e^{iH(\tau-\tau_1)}Oe^{-iH\tau}|\psi\rangle\right\| d\tau_1.
\end{aligned} \tag{424}$$

In the same way, we can derive

$$\left\||\psi\rangle - e^{i\tilde{H}\tau}e^{-iH\tau}|\psi\rangle\right\| \le \left\|\bar{\Pi}^{\mathrm{c}}|\psi\rangle\right\| + \left\|\bar{\Pi}^{\mathrm{c}}e^{-iH\tau}|\psi\rangle\right\| + \int_0^\tau \left\|\bar{\Pi}H_0\bar{\Pi}^{\mathrm{c}}e^{-iH\tau_1}|\psi\rangle\right\| d\tau_1 \tag{425}$$

by choosing $O = \hat{1}$ in the inequality (424). Therefore, by applying the inequalities (424) and (425) to (416), we obtain the desired inequality (408). This completes the proof. □

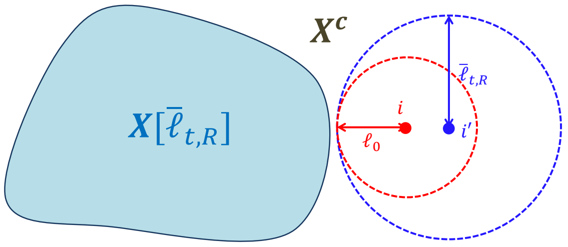

Supplementary Figure 10. For $\forall i[\ell_0] \in X[\bar{\ell}_{t,R}]^{\rm c}$ with $\ell_0 < \bar{\ell}_{t,R}$, we can find another site $i' \in X^{\rm c}$ such that $i'[\bar{\ell}_{t,R}] \subset X[\bar{\ell}_{t,R}]^{\rm c}$ and $i'[\bar{\ell}_{t,R}] \supset i[\ell_0]$.

A. Probability distribution for boson number after time evolution

In estimating the errors in (408) for the effective Hamiltonian, we have to estimate the norms like

$$\left\| \bar{\Pi}^{\rm c} e^{iH\tau_2} O e^{-iH\tau_1} |\psi\rangle \right\| \quad \text{and} \quad \left\| \bar{\Pi} H_0 \bar{\Pi}^{\rm c} e^{iH\tau_2} O e^{-iH\tau_1} |\psi\rangle \right\| . \tag{426}$$

For this purpose, we let

$$O \to O_{X_0} \quad (X_0 \subseteq i_0[r_0]), \quad |\psi\rangle \to \rho_0, \tag{427}$$

where ρ_0 satisfies the low-boson density condition in Assumption 3 with $b_\rho = b_0/\gamma$. We also assume that the operator O_{X_0} satisfies the following condition, which is characterized by a parameter q_0:

$$\Pi_{X_0,>m+q_0} O_{X_0} \Pi_{X_0,m} = 0, \quad \Pi_{X_0,<m-q_0} O_{X_0} \Pi_{X_0,m} = 0 \quad \text{for} \quad \forall m > 0. \tag{428}$$

Here, the constant q_0 is the maximum number of boson creation (or annihilation) by the operator O_{X_0}. The parameter q_0 appears in the Lieb-Robinson bound (see Theorems 2, 3 and 4). For example, if O_{X_0} conserve the boson number in the region X_0, we have $q_0 = 0$. We also set the norm of O_{X_0} as

$$\|O_{X_0}\| = \zeta_0 \tag{429}$$

for simplicity.

In the above setup, we consider a quantum state of[*3]

$$\tilde{\rho}_{\tau_1,\tau_2} := e^{iH\tau_2} O_{X_0} e^{-iH\tau_1} \rho_0 e^{iH\tau_1} O_{X_0}^\dagger e^{-iH\tau_2} \quad \text{with} \quad |\tau_1|, |\tau_2| \le t. \tag{431}$$

Note that $\tilde{\rho}_{\tau_1,\tau_2}$ is simply equal to $\rho_0(t)$ by choosing $\tau_1 = t$, $\tau_2 = 0$ and $O_{X_0} = \hat{1}$. Our purpose in this section is to estimate the upper bound of the probability distribution

$$\mathrm{tr}\left[\Pi_{i,>q_i} \tilde{\rho}_{\tau_1,\tau_2}\right] \quad \text{for} \quad i \in \Lambda. \tag{432}$$

As has been proved in Proposition 20, the distribution decays (sub)exponentially for $q_i \gtrsim [t\log(t)]^D$ if the site i is sufficiently separated from the region X_0. We here prove the following lemma:

Lemma 28. *We define $\bar{\ell}_{t,R}$ as*

$$\bar{\ell}_{t,R} := \bar{c}_0 t \log(Rq_0), \tag{433}$$

where $\bar{c}_0$ is an appropriate constant and R is a length scale such that $R \ge t$ and $R \ge r_0$ [see Eq. (427) for the definition of r_0]. Then, for a quantum state $\tilde{\rho}_{\tau_1,\tau_2}$ in Eq. (431), we obtain

$$\mathrm{tr}\left(\hat{n}_{i[\ell_0]}^p \Pi_{i[\ell_0],>q_i} \tilde{\rho}_{\tau_1,\tau_2} \right) \le \zeta_0^2 q_i^p e^{-\left[q_i/(4b_0\ell_0^D)\right]^{1/\kappa}} \quad \textit{for} \quad i[\ell_0] \subset X_0[\bar{\ell}_{t,R}]^{\rm c}, \tag{434}$$

and

$$\mathrm{tr}\left(\hat{n}_{X_0[\bar{\ell}_{t,R}]}^p \Pi_{X_0[\bar{\ell}_{t,R}],>q} \tilde{\rho}_{\tau_1,\tau_2} \right) \le \zeta_0^2 q^p e^{-\left[(q-32e^2q_0^3)/\xi_{t,R}\right]^{1/\kappa}}, \quad \xi_{t,R} := 16 b_0 \bar{\ell}_{t,R}^{2D} |X_0|, \tag{435}$$

where $k \in \mathbb{N}$ and ℓ_0 is assumed to be larger than $\bar{\ell}_{t,R}$ ($\ell_0 \ge \bar{\ell}_{t,R}$).

[*3] In the definition (431), we consider a time evolution by a time-independent Hamiltonian. But, all the discussion can be straightforwardly generalized to

$$\tilde{\rho}_{\tau_1,\tau_2} := U_{H_x',0\to\tau_2}^\dagger O_{X_0} U_{H_x,0\to\tau_1} \rho_0 U_{H_x,0\to\tau_1}^\dagger O_{X_0}^\dagger U_{H_x',0\to\tau_2}, \tag{430}$$

where H_x and H_x' are time-dependent Hamiltonians and we use the notation (33).

Remark. In the inequality (434), the condition $\ell_0 \ge \bar{\ell}_{t,R}$ is assumed. In the case of $\ell_0 < \bar{\ell}_{t,R}$, we can still prove the following inequality:

$$\mathrm{tr}\left(\hat{n}^p_{i[\ell_0]}\Pi_{i[\ell_0],>q_i}\tilde{\rho}_{\tau_1,\tau_2}\right) \le \zeta_0^2 q_i^p e^{-\left[q_i/(4b_0\bar{\ell}^D_{t,R})\right]^{1/\kappa}} \quad \text{for} \quad i[\ell_0] \subset X_0[\bar{\ell}_{t,R}]^{\mathrm{c}}. \tag{436}$$

The proof is given as follows. First, for $i[\ell_0] \subset X_0[\bar{\ell}_{t,R}]^{\mathrm{c}}$, there exists a site i' such that $i'[\bar{\ell}_{t,R}] \supset i[\ell_0]$ and $i'[\bar{\ell}_{t,R}] \subset X_0[\bar{\ell}_{t,R}]^{\mathrm{c}}$ (see Fig. 10). Hence, we obtain

$$\mathrm{tr}\left(\hat{n}^p_{i[\ell_0]}\Pi_{i[\ell_0],>q_i}\tilde{\rho}_{\tau_1,\tau_2}\right) \le \mathrm{tr}\left(\hat{n}^p_{i'[\bar{\ell}_{t,R}]}\Pi_{i'[\bar{\ell}_{t,R}],>q_i}\tilde{\rho}_{\tau_1,\tau_2}\right), \tag{437}$$

which yields the inequality (436) from (434) by choosing $\ell_0 = \bar{\ell}_{t,R}$.

1. *Proof of Lemma 28*

The proof relies on the iterative uses of Proposition 20. In the first step, we consider the moment function of

$$\mathrm{tr}\left(\rho_0(\tau_1)\hat{n}^s_{i[\ell_0]}\right) \quad \text{for} \quad |\tau_1| \le t \tag{438}$$

for $\forall i \in \Lambda$. From Assumption 3 with $b_\rho = b_0/\gamma$ in (34), the initial state ρ_0 satisfies

$$\mathrm{tr}\left(\rho_0\hat{n}^s_{i[\ell_0]}\right) \le \frac{1}{e}\left(\frac{b_0}{e}s^\kappa \ell_0^D\right)^s. \tag{439}$$

Hence, we obtain from Proposition 20

$$\mathrm{tr}\left(\rho_0(\tau_1)\hat{n}^s_{i[\ell_0]}\right) \le \frac{1}{e}\left(\frac{2b_0}{e}s^\kappa \ell_0^D\right)^s \quad \text{for} \quad \forall i \in \Lambda, \quad \ell_0 \ge \bar{\ell}_1, \tag{440}$$

where $\bar{\ell}_1$ is determined by the condition (324), i.e., $\bar{\ell}_1 \propto t\log t$.

In the second step, we first consider the moment function of

$$\mathrm{tr}\left(O_{X_0}\rho_0(\tau_1)O^\dagger_{X_0}\hat{n}^s_{i[\ell_0]}\right) =: \mathrm{tr}\left(\tilde{\rho}_{\tau_1}\hat{n}^s_{i[\ell_0]}\right) \quad \text{for} \quad i[\ell_0] \subset X_0^{\mathrm{c}}. \tag{441}$$

From the inequality (440) and $\left[O_{X_0}, \hat{n}^s_{i[\ell_0]}\right] = 0$, we trivially obtain

$$\mathrm{tr}\left(\tilde{\rho}_{\tau_1}\hat{n}^s_{i[\ell_0]}\right) \le \frac{\zeta_0^2}{e}\left(\frac{2b_0}{e}s^\kappa \ell_0^D\right)^s \quad \text{for} \quad i[\ell_0] \in X_0^{\mathrm{c}}, \quad \ell_0 \ge \bar{\ell}_1, \tag{442}$$

where we use $\|O_{X_0}\| = \zeta_0$. On the other hand, if we consider a boson number operator $\hat{n}_Z$ such that $Z \cap X_0 \ne \emptyset$, we cannot obtain the same inequality as (442) due to the boson creation by O_{X_0}. We then consider a subset $X[r]$ for generic $r \ge \bar{\ell}_1$ and $X \subset \Lambda$. From the condition (428), the operator O_{X_0} increases the boson number up to q_0 in the region X_0, and hence we utilize Lemma 3 with

$$O_q \to O_{X_0}, \quad \text{and} \quad (\nu_i = 1 \quad \text{for} \quad i \in X[r], \quad \& \quad \nu_i = 0 \quad \text{for} \quad i \in X[r]^{\mathrm{c}}),$$

which yields

$$\begin{aligned}\mathrm{tr}\left(\tilde{\rho}_{\tau_1}\hat{n}^s_{X[r]}\right) = \mathrm{tr}\left(O_{X_0}\rho_0(\tau_1)O^\dagger_{X_0}\hat{n}^s_{X[r]}\right) &\le 4^s\zeta_0^2 \mathrm{tr}\left[\rho_0(\tau_1)(4q_0^3 + \hat{n}_{X[r]})^s\right] \\ &\le 4^s\zeta_0^2\left\{4q_0^3 + \sum_{i\in X}\left[\mathrm{tr}\left(\rho_0(\tau_1)\hat{n}^s_{i[r]}\right)\right]^{1/s}\right\}^s.\end{aligned} \tag{443}$$

where in the second inequality we use $\hat{n}_{X[r]} \preceq \sum_{i\in X}\hat{n}_{i[r]}$ and

$$\langle (O_1 + O_2 + \cdots + O_m)^s\rangle \le \left(\langle O_1^s\rangle^{1/s} + \langle O_2^s\rangle^{1/s} + \cdots + \langle O_m^s\rangle^{1/s}\right)^s$$

for arbitrary positive semi-definite Hermitian operators $\{O_1, O_2, \dots, O_m\}$ as long as $\{O_j\}_{j=1}^m$ commute with each other. By applying the inequality (440) to (443), we have

$$\begin{aligned}\mathrm{tr}(\tilde{\rho}_{\tau_1}\hat{n}^s_{X[r]}) \le 4^s\zeta_0^2\left(4q_0^3 + \frac{|X|}{e^{1/s}}\cdot\frac{2b_0}{e}s^\kappa r^D\right)^s &\le \frac{\zeta_0^2}{e}\left(16eq_0^3 + |X|\cdot\frac{8b_0}{e}s^\kappa r^D\right)^s \quad \text{for} \quad \forall r \ge \bar{\ell}_1 \\ \le \frac{\zeta_0^2}{e}\left(16eq_0^3 + |X|\cdot\frac{8b_0\bar{\ell}_1^D}{e}s^\kappa \max(r^D, 1)\right)^s \quad \text{for} \quad \forall r \ge 0,\end{aligned} \tag{444}$$

where we can choose $\forall X \subset \Lambda$.

In the final step, we consider the moment function for the quantum state

$$\tilde{\rho}_{\tau_1,\tau_2} = e^{iH\tau_2} O_{X_0} e^{-iH\tau_1} \rho e^{iH\tau_1} O_{X_0}^\dagger e^{-iH\tau_2} = e^{iH\tau_2} \tilde{\rho}_{\tau_1} e^{-iH\tau_2} = \tilde{\rho}_{\tau_1}(-\tau_2). \tag{445}$$

Here, the initial state $\tilde{\rho}_{\tau_1}(0)$ satisfies the inequalities (444) and (442), which give the initial conditions (318) and (319) for Proposition 20, respectively, and hence we can let in Proposition 20

$$b_0 \to 2b_0, \quad q_0 \to 16eq_0^3, \quad q_1 \to \frac{8b_0\bar{\ell}_1^D}{e}, \quad \bar{\ell}_0 \to \bar{\ell}_1 \propto t\log(t). \tag{446}$$

Then, from the inequalities (321) and (322) we have

$$\mathrm{tr}\left(\tilde{\rho}_{\tau_1}(-\tau_2)\hat{n}_{i[\ell_0]}^s\right) \le \frac{\zeta_0^2}{e}\left(\frac{4b_0}{e}s^\kappa \ell_0^D\right)^s \quad \text{for} \quad i[\ell_0] \subset X_0[\bar{\ell}_2]^{\mathrm{c}}, \tag{447}$$

and

$$\mathrm{tr}\left(\tilde{\rho}_{\tau_1}(-\tau_2)\hat{n}_{X_0[\ell_0]}^s\right) \le \frac{\zeta_0^2}{e}\left(32eq_0^3 + \frac{16b_0\bar{\ell}_1^D\ell_0^D}{e}s^\kappa|X_0|\right)^s, \tag{448}$$

where $\ell_0 \ge \max(2D\bar{\ell}_2, \bar{\ell}_1)$ and $\bar{\ell}_2$ is given by the condition (323), i.e.,

$$\bar{\ell}_2 = t\log[\Theta(tq_0|X_0|)], \tag{449}$$

which is also given in the form of Eq. (433) because of $X_0 = i_0[r_0] \subseteq i_0[R]$. In the following, we define $\bar{\ell}_{t,R}$ as $2D\bar{\ell}_2$, i.e., $\bar{\ell}_2 := \bar{\ell}_{t,R}/(2D)$.

By applying Lemma 2 [i.e., the inequality (44)] to the inequality (447), we obtain

$$\mathrm{tr}\left(\hat{n}_{i[\ell_0]}^p \Pi_{i[\ell_0],>q_i}\tilde{\rho}_{\tau_1,\tau_2}\right) \le \zeta_0^2 q_i^p e^{-\left[q_i/(4b_0\ell_0^D)\right]^{1/\kappa}} \quad \text{for} \quad i[\ell_0] \subset X_0[\bar{\ell}_{t,R}/(2D)]^{\mathrm{c}}, \tag{450}$$

where we assume $\ell_0 \ge \bar{\ell}_{t,R}$. Note that $i[\ell_0] \subset X_0[\bar{\ell}_{t,R}/(2D)]^{\mathrm{c}}$ is included in the condition $i[\ell_0] \subset X_0[\bar{\ell}_{t,R}]^{\mathrm{c}}$. Moreover, we apply Lemma 2 to the inequality (448) with $\ell_0 = \bar{\ell}_{t,R}$, which yields

$$\mathrm{tr}\left(\hat{n}_{X_0[\bar{\ell}_{t,R}]}^p \Pi_{X_0[\bar{\ell}_{t,R}],>q}\tilde{\rho}_{\tau_1,\tau_2}\right) \le \zeta_0^2 q^p \exp\left[-\left(\frac{q - 32e^2q_0^3}{16b_0\bar{\ell}_{t,R}^{2D}|X_0|}\right)^{1/\kappa}\right], \tag{451}$$

where we use $\bar{\ell}_1 \le \bar{\ell}_{t,R}$. We thus prove the main inequalities (434) and (435). This completes the proof of Lemma 28. □

B. Effective Hamiltonian by the truncation of the boson number: first result

By combining the obtained statements in Lemma 25 (or Corollary 26) and Lemma 28, we aim to derive an explicit error bound for the effective Hamiltonian. We here define the projection operator $\bar{\Pi}_{L,\bar{q}}$ as

$$\bar{\Pi}_{L,\bar{q}} := \prod_{i\in L} \Pi_{i,\le\bar{q}}, \tag{452}$$

which uniformly truncates the boson number at each site in the region L by $\bar{q}$. This type of projection will be considered in proving a non-optimal Lieb-Robinson bound in Theorem 2 in Sec. Supplementary Note 7.

We here approximate the dynamics e^{-iHt} by using the effective Hamiltonian $\bar{\Pi}_{L,\bar{q}}H\bar{\Pi}_{L,\bar{q}}$, whose approximation error is obtained from the following proposition:

Proposition 29. *In the same setup as in Sec. Supplementary Note 6 A, we obtain the approximation error as*

$$\left\| O_{X_0}(H,t)\rho_0 - O_{X_0}(\bar{\Pi}_{L,\bar{q}}H\bar{\Pi}_{L,\bar{q}},t)\rho_0 \right\|_1 \le 4\zeta_0\gamma|L|\left(1+\bar{J}t\bar{q}\right)e^{-\left[\bar{q}/(4b_0\bar{\ell}_{t,R}^D)\right]^{1/\kappa}/2}, \tag{453}$$

where we assume $d_{X_0,L} \ge \bar{\ell}_{t,R} + 1$ (see Fig. 11).

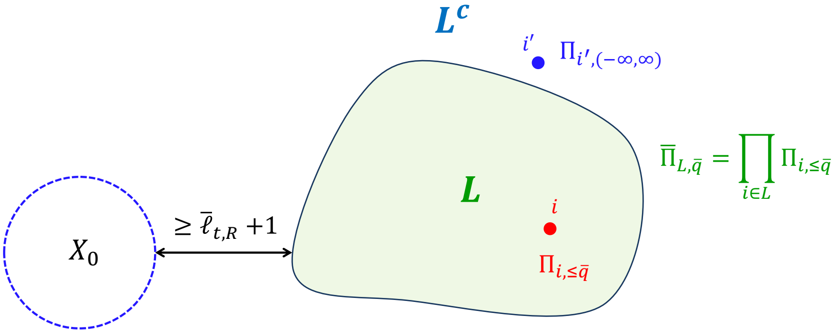

Supplementary Figure 11. Definition of the projection $\bar{\Pi}_{L,\bar{q}}$. In the projection, we truncate the boson number at each of the sites in the region L up to $\bar{q}$, while there are no restrictions on the boson numbers in $L^{\rm c}$. For the projection, the approximation error for dynamics by the effective Hamiltonian $\bar{\Pi}_{L,\bar{q}} H \bar{\Pi}_{L,\bar{q}}$ is estimated in Proposition 29.

1. *Proof of Proposition 29*

In applying Lemma 25 (or Corollary 26) to the estimation of the norm in (453), we need to estimate the norms of $\left\| \bar{\Pi}^{\rm c}_{L,\bar{q}} O_{X_0}(\tau_2)\sqrt{\rho_0(\tau_1)} \right\|_F$ and $\left\| \bar{\Pi}_{L,\bar{q}} H_0 \bar{\Pi}^{\rm c}_{L,\bar{q}} O_{X_0}(\tau_2)\sqrt{\rho_0(\tau_1)} \right\|_F$ for arbitrary $|\tau_1|, |\tau_2| \le t$. For the quantities, we aim to prove the following upper bounds:

$$\left\| \bar{\Pi}^{\rm c}_{L,\bar{q}} O_{X_0}(\tau_2)\sqrt{\rho_0(\tau_1)} \right\|_F \le \zeta_0 |L|^{1/2} e^{-\left[\bar{q}/(4b_0 \bar{\ell}^D_{t,R})\right]^{1/\kappa}/2}, \tag{454}$$

and

$$\left\| \bar{\Pi}_{L,\bar{q}} H_0 \bar{\Pi}^{\rm c}_{L,\bar{q}} O_{X_0}(\tau_2)\sqrt{\rho_0(\tau_1)} \right\|_F \le 2\gamma \bar{J} \zeta_0 \bar{q} |L| e^{-\left[\bar{q}/(4b_0 \bar{\ell}^D_{t,R})\right]^{1/\kappa}/2}. \tag{455}$$

By applying the above inequalities with appropriate τ_1 and τ_2 to the upper bounds in (409), we obtain

$$\left\| O_{X_0}(H,t)\rho_0 - O_{X_0}(\bar{\Pi}_{L,\bar{q}} H \bar{\Pi}_{L,\bar{q}}, t)\rho_0 \right\|_1 \le 4\zeta_0 |L|^{1/2} e^{-\left[\bar{q}/(4b_0 \bar{\ell}^D_{t,R})\right]^{1/\kappa}/2} + 4t\gamma \bar{J} \zeta_0 \bar{q} |L| e^{-\left[\bar{q}/(4b_0 \bar{\ell}^D_{t,R})\right]^{1/\kappa}/2}, \tag{456}$$

which reduces to the main inequality (453) from $\gamma \ge 1$. Therefore, the remaining tasks are proving the inequalities (454) and (455).

[Proofs of the inequalities (454) and (455)]
By using the inequality (436) with $p = 0$ in Lemma 28, we immediately prove the first inequality (454) as follows:

$$\begin{aligned} \left\| \bar{\Pi}^{\rm c}_{L,\bar{q}} O_{X_0}(\tau_2)\sqrt{\rho_0(\tau_1)} \right\|_F^2 &= {\rm tr}\left(\bar{\Pi}^{\rm c}_{L,\bar{q}} O_{X_0}(\tau_2)\rho_0(\tau_1) O_{X_0}(\tau_2)^\dagger \bar{\Pi}^{\rm c}_{L,\bar{q}} \right) \le \sum_{i\in L} {\rm tr}\left(\Pi_{i,>\bar{q}} O_{X_0}(\tau_2)\rho_0(\tau_1) O_{X_0}(\tau_2)^\dagger \right) \\ &\le \zeta_0^2 |L| e^{-\left[\bar{q}/(4b_0 \bar{\ell}^D_{t,R})\right]^{1/\kappa}}, \end{aligned} \tag{457}$$

where we use $i \in X_0[\bar{\ell}_{t,R}]^{\rm c}$ for $\forall i \in L$ from $d_{X_0,L} \ge \bar{\ell}_{t,R} + 1 > \bar{\ell}_{t,R}$, which is assumed in the statement of Proposition 29.

Second, to prove the inequality (455), we first decompose

$$\bar{\Pi}_{L,\bar{q}} H_0 \bar{\Pi}^{\rm c}_{L,\bar{q}} = \bar{\Pi}_{L,\bar{q}} \left(H_{0,L} + \partial h_L \right) \bar{\Pi}^{\rm c}_{L,\bar{q}}, \tag{458}$$

where we use the notation (28) for ∂h_L and $\bar{\Pi}_{L,\bar{q}} H_{0,L^{\rm c}} \bar{\Pi}^{\rm c}_{L,\bar{q}} = H_{0,L^{\rm c}} \bar{\Pi}_{L,\bar{q}} \bar{\Pi}^{\rm c}_{L,\bar{q}} = 0$. Then, by using

$$|b_i b_j^\dagger| = \sqrt{(b_i b_j^\dagger)^\dagger b_i b_j^\dagger} = \sqrt{\hat{n}_i(\hat{n}_j + 1)} \preceq \hat{n}_i + \hat{n}_j, \tag{459}$$

and

$$\bar{\Pi}_{L,\bar{q}} b_i b_j^\dagger \bar{\Pi}^{\rm c}_{L,\bar{q}} = \bar{\Pi}_{L,\bar{q}} b_i b_j^\dagger \bar{\Pi}^{\rm c}_{i,j,\bar{q}}, \quad \bar{\Pi}_{i,j,\bar{q}} := \begin{cases} \Pi_{i,\le\bar{q}} \Pi_{j,\le\bar{q}} & \text{for} \quad i,j \in L, \\ \Pi_{i,\le\bar{q}} & \text{for} \quad i \in L, \quad j \in L^{\rm c}, \end{cases} \tag{460}$$

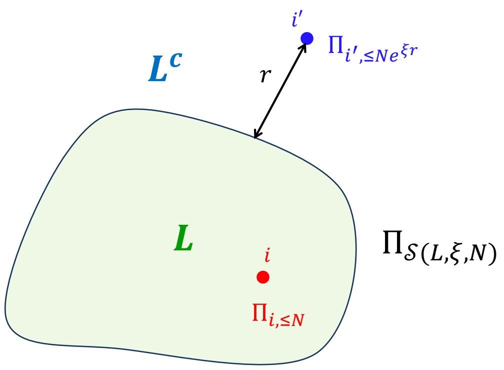

Supplementary Figure 12. Definition of the projection $\Pi_{\mathcal{S}(L,\xi,N)}$. In this projection, we truncate the boson number at each of the sites in the whole region up to q_i ($i \in \Lambda$), where $q_i = N$ for $i \in L$ and $q_i = Ne^{\xi d_{i,L}}$ for $i \in L^{\rm c}$. As the site i is separated from the region L, the truncation number rapidly increases with the distance. The approximation error for dynamics by the effective Hamiltonian $\Pi_{\mathcal{S}(L,1/2,N)} H \Pi_{\mathcal{S}(L,1/2,N)}$ is given by Proposition 30.

we obtain

$$\begin{aligned}\left\|\bar{\Pi}_{L,\bar{q}} H_0 \bar{\Pi}^{\rm c}_{L,\bar{q}} O_{X_0}(\tau_2)\sqrt{\rho_0(\tau_1)}\right\|_F &\leq \bar{J} \sum_{i\in L}\sum_{j:d_{i,j}=1} \left\|\bar{\Pi}_{L,\bar{q}} (b_i b_j^\dagger + {\rm h.c.}) \bar{\Pi}^{\rm c}_{i,j,\bar{q}} O_{X_0}(\tau_2)\sqrt{\rho_0(\tau_1)}\right\|_F \\ &\leq \bar{J} \sum_{i\in L}\sum_{j:d_{i,j}=1} \left\|\left(|b_i b_j^\dagger| + |b_j b_i^\dagger|\right) \bar{\Pi}^{\rm c}_{i,j,\bar{q}} O_{X_0}(\tau_2)\sqrt{\rho_0(\tau_1)}\right\|_F \\ &\leq 2\bar{J} \sum_{i\in L}\sum_{j:d_{i,j}=1} \left\|(\hat{n}_i + \hat{n}_j) \bar{\Pi}^{\rm c}_{i,j,\bar{q}} O_{X_0}(\tau_2)\sqrt{\rho_0(\tau_1)}\right\|_F . \end{aligned} \tag{461}$$

By applying the inequality (436) with $p=2$ to $\left\|(\hat{n}_i + \hat{n}_j) \bar{\Pi}^{\rm c}_{i,j,\bar{q}} O_{X_0}(\tau_2)\sqrt{\rho_0(\tau_1)}\right\|_F$, we obtain

$$\begin{aligned}\left\|(\hat{n}_i + \hat{n}_j) \bar{\Pi}^{\rm c}_{i,j,\bar{q}} O_{X_0}(\tau_2)\sqrt{\rho_0(\tau_1)}\right\|_F^2 &= {\rm tr}\left[(\hat{n}_i + \hat{n}_j)^2 \bar{\Pi}^{\rm c}_{i,j,\bar{q}} O_{X_0}(\tau_2)\rho_0(\tau_1) O_{X_0}(\tau_2)^\dagger\right] \\ &\leq {\rm tr}\left[\hat{n}_{i[1]}^2 \Pi_{i[1],>\bar{q}} O_{X_0}(\tau_2)\rho_0(\tau_1) O_{X_0}(\tau_2)^\dagger\right] \\ &\leq \zeta_0^2 \bar{q}^2 e^{-\left[\bar{q}/(4b_0 \bar{\ell}_{t,R}^D)\right]^{1/\kappa}}, \end{aligned} \tag{462}$$

where we use $\hat{n}_i + \hat{n}_j \preceq \hat{n}_{i[1]}$ and $\bar{\Pi}^{\rm c}_{i,j,\bar{q}} = \Pi_{i[1],>\bar{q}} \bar{\Pi}^{\rm c}_{i,j,\bar{q}}$ from $d_{i,j}=1$ in the first inequality, and in the second inequality, we ensure $i[1] \subset X_0[\bar{\ell}_{t,R}]^{\rm c}$ for $\forall i \in L$ from the condition $d_{X_0,L} \geq \bar{\ell}_{t,R}+1$.

By combining the inequalities (461) and (462), we arrive at the inequality (455) as follows:

$$\begin{aligned}\left\|\bar{\Pi}_{L,\bar{q}} H_0 \bar{\Pi}^{\rm c}_{L,\bar{q}} O_{X_0}(\tau_2)\sqrt{\rho_0(\tau_1)}\right\|_F &\leq 2\bar{J} \sum_{i\in L}\sum_{j:d_{i,j}=1} \zeta_0 \bar{q} e^{-\left[\bar{q}/(4b_0 \bar{\ell}_{t,R}^D)\right]^{1/\kappa}/2} \\ &\leq 2\gamma \bar{J} \zeta_0 \bar{q} |L| e^{-\left[\bar{q}/(4b_0 \bar{\ell}_{t,R}^D)\right]^{1/\kappa}/2}, \end{aligned} \tag{463}$$

where we use $\sum_{j:d_{i,j}=1} 1 \leq |i[1]| \leq \gamma$ for $\forall i \in \Lambda$. This completes the proof. $\square$

C. Effective Hamiltonian by the truncation of the boson number: second result

In Eq. (37), we have define the set $\mathcal{S}(L,\xi,N)$ as

$$\mathcal{S}(L,\xi,N) := \left\{\mathbf{N} | N_i \leq N e^{\xi d_{i,L}} \quad {\rm for} \quad i \in \Lambda\right\}. \tag{464}$$

We consider the effective Hamiltonian, which is defined by the projection of (see Fig. 12)

$$\Pi_{\mathcal{S}(L,\xi,N)} := \prod_{i\in L} \Pi_{i,\leq N} \prod_{i \in L^{\rm c}} \Pi_{i,\leq Ne^{\xi d_{i,L}}}. \tag{465}$$

In our analyses, it is often pretty convenient to restrict the Hilbert space which is spanned by $\Pi_{\mathcal{S}(L,\xi,N)}$.

In the following, we choose $\xi = 1/2$ and denote the projection

$$\Pi_{\mathcal{S}(L,1/2,N)} = \tilde{\Pi}_{(L,N)} \tag{466}$$

for simplicity. We here consider the approximate time evolution by using the following effective Hamiltonian:

$$\tilde{\Pi}_{(L,N)} H \tilde{\Pi}_{(L,N)} = \tilde{H}_{(L,N)}. \tag{467}$$

We aim to prove the following proposition:

Proposition 30. *Under the assumption of*

$$N \geq \max\left\{64e^2 q_0^3, 2(8\kappa)^\kappa \xi_{t,R}\right\}, \tag{468}$$

the approximation error using the effective Hamiltonian $\tilde{H}_{(L,N)}$ is upper-bounded by

$$\left\| \left[O_{X_0}(H,t) - O_{X_0}(\tilde{H}_{(L,N)},t) \right] \rho_0 \right\|_1 \leq 2^{2D+5} \zeta_0 \gamma |L| D! \left(1 + 2c_\kappa \gamma \bar{J} t \xi_{t,R}^2\right) e^{-[N/(2\xi_{t,R})]^{1/\kappa}/(8\kappa)}, \tag{469}$$

where $c_\kappa := 2^{4\kappa+3}\kappa\Gamma(2\kappa)$. We also obtain

$$\left\| O_{X_0}(H,t)\rho_0 - O_{X_0}(\tilde{H}_{(L,N)},t)\tilde{\Pi}_{(L,N)}\rho_0\tilde{\Pi}_{(L,N)} \right\|_1 \leq 2^{2D+6} \zeta_0 \gamma |L| D! \left(1 + c_\kappa \gamma \bar{J} t \xi_{t,R}^2\right) e^{-[N/(2\xi_{t,R})]^{1/\kappa}/(8\kappa)}. \tag{470}$$

1. *Proof of Proposition 30*

The proof is similar to the one for Proposition 29. To apply Corollary 26 to the approximation error, we aim to estimate the norms of

$$\left\| \tilde{\Pi}^{\rm c}_{(L,N)} O_{X_0}(\tau_2) \sqrt{\rho_0(\tau_1)} \right\|_F \quad \text{and} \quad \left\| \tilde{\Pi}_{(L,N)} H_0 \tilde{\Pi}^{\rm c}_{(L,N)} O_{X_0}(\tau_2) \sqrt{\rho_0(\tau_1)} \right\|_F$$

for arbitrary $|\tau_1|, |\tau_2| \leq t$. For the above two quantities, we prove the following inequalities for $N \geq 2(8\kappa)^\kappa \xi_{t,R}$ (see below for the proofs):

$$\left\| \tilde{\Pi}^{\rm c}_{(L,N)} O_{X_0}(\tau_2) \sqrt{\rho_0(\tau_1)} \right\|_F \leq 2^{2D+3} \zeta_0 \gamma |L| D! e^{-[N/(2\xi_{t,R})]^{1/\kappa}/(4\kappa)}, \tag{471}$$

and

$$\left\| \tilde{\Pi}_{(L,N)} H_0 \tilde{\Pi}^{\rm c}_{(L,N)} O_{X_0}(\tau_2) \sqrt{\rho_0(\tau_1)} \right\|_F \leq 2^{2D+5} c_\kappa \zeta_0 \bar{J} \xi_{t,R}^2 \gamma^2 |L| D! e^{-[N/(2\xi_{t,R})]^{1/\kappa}/(8\kappa)}, \tag{472}$$

where $c_\kappa := 2^{4\kappa+3}\kappa\Gamma(2\kappa)$.

By applying the inequalities (471) and (472) with appropriate choices of τ_1 and τ_2 to the upper bounds in (409), we have

$$\begin{aligned}
&\left\| O_{X_0}(H,t)\rho_0 - O_{X_0}(\tilde{H}_{(L,N)},t)\rho_0 \right\|_1 \\
&\leq 2^{2D+5} \zeta_0 \gamma |L| D! e^{-[N/(2\xi_{t,R})]^{1/\kappa}/(4\kappa)} + 2^{2D+6} t c_\kappa \zeta_0 \bar{J} \xi_{t,R}^2 \gamma^2 |L| D! e^{-[N/(2\xi_{t,R})]^{1/\kappa}/(8\kappa)} \\
&\leq 2^{2D+5} \zeta_0 \gamma |L| D! \left(1 + 2c_\kappa \gamma \bar{J} t \xi_{t,R}^2\right) e^{-[N/(2\xi_{t,R})]^{1/\kappa}/(8\kappa)}.
\end{aligned} \tag{473}$$

We thus prove the first main inequality (469).

In deriving the inequality of (470), because of

$$\begin{aligned}
&\left\| O_{X_0}(H,t)\rho_0 - O_{X_0}(\tilde{H}_{(L,N)},t)\tilde{\Pi}_{(L,N)}\rho_0\tilde{\Pi}_{(L,N)} \right\|_1 \\
&= \left\| O_{X_0}(H,t)\rho_0 - O_{X_0}(\tilde{H}_{(L,N)},t)\rho_0 + O_{X_0}(\tilde{H}_{(L,N)},t)\rho_0 - O_{X_0}(\tilde{H}_{(L,N)},t)\tilde{\Pi}_{(L,N)}\rho_0\tilde{\Pi}_{(L,N)} \right\|_1 \\
&\leq \left\| O_{X_0}(H,t)\rho_0 - O_{X_0}(\tilde{H}_{(L,N)},t)\rho_0 \right\|_1 + \left\| O_{X_0}(\tilde{H}_{(L,N)},t)\left(\rho_0 - \tilde{\Pi}_{(L,N)}\rho_0\tilde{\Pi}_{(L,N)}\right) \right\|_1,
\end{aligned} \tag{474}$$

we only have to estimate

$$\begin{aligned}
\left\| O_{X_0}(\tilde{H}_{(L,N)},t)\left(\rho_0 - \tilde{\Pi}_{(L,N)}\rho_0\tilde{\Pi}_{(L,N)}\right) \right\|_1 &\leq \zeta_0 \left\| \rho_0 - \tilde{\Pi}_{(L,N)}\rho_0\tilde{\Pi}_{(L,N)} \right\|_1 \\
&\leq 2\zeta_0 \left\| \rho_0 \tilde{\Pi}^{\rm c}_{(L,N)} \right\|_1 \leq 2\zeta_0 \left\| \tilde{\Pi}^{\rm c}_{(L,N)} \sqrt{\rho_0} \right\|_F,
\end{aligned} \tag{475}$$

where we use

$$\left\| \rho_0 - \tilde{\Pi}_{(L,N)}\rho_0\tilde{\Pi}_{(L,N)} \right\|_1 = \left\| (1-\tilde{\Pi}_{(L,N)})\rho_0\tilde{\Pi}_{(L,N)} + \rho_0(1-\tilde{\Pi}_{(L,N)}) \right\|_1 \leq 2\left\| \rho_0 \tilde{\Pi}^{\rm c}_{(L,N)} \right\|_1 \tag{476}$$

and [*4]

$$\left\|\rho_0\tilde{\Pi}^{\rm c}_{(L,N)}\right\|_1 = \left\|\tilde{\Pi}^{\rm c}_{(L,N)}\rho_0\right\|_1 \le \|\sqrt{\rho_0}\|_2 \left\|\tilde{\Pi}^{\rm c}_{(L,N)}\sqrt{\rho_0}\right\|_2 = \left\|\tilde{\Pi}^{\rm c}_{(L,N)}\sqrt{\rho_0}\right\|_F. \tag{479}$$

Note that Schatten 2 norm $\|\cdot\|_2$ is equivalent to the Frobenius norm $\|\cdot\|_F$. By using the inequality (471) with $O_{X_0} = \hat{1}$, we immediately obtain

$$\left\|\tilde{\Pi}^{\rm c}_{(L,N)}\sqrt{\rho_0}\right\|_F \le 2^{2D+3}\zeta_0\gamma|L|D! e^{-[N/(2\xi_{t,R})]^{1/\kappa}/(4\kappa)}. \tag{480}$$

Therefore, by combining the above inequality with (473),

$$\left\|O_{X_0}(\tilde{H}_{(L,N)},t)\left(\rho_0 - \tilde{\Pi}_{(L,N)}\rho_0\tilde{\Pi}_{(L,N)}\right)\right\|_1 \le 2^{2D+5}\zeta_0\gamma|L|D!\left(1/2+1+2c_\kappa\gamma\bar{J}t\xi_{t,R}^2\right)e^{-[N/(2\xi_{t,R})]^{1/\kappa}/(8\kappa)},$$

which yields the second main inequality (470). This completes the proof of Proposition 30. □

[Proof of the inequalities (471) and (472)]

From the inequalities (434) and (435) in Lemma 28, we can obtain the following looser bound which holds for all $i\in\Lambda$:

$$\mathrm{tr}\left(\hat{n}^p_{i[\bar{\ell}_{t,R}]}\Pi_{i[\bar{\ell}_{t,R}],>q_i}\tilde{\rho}_{\tau_1,\tau_2}\right) \le \zeta_0^2 q_i^p e^{-\left[(q_i-32e^2q_0^3)/\xi_{t,R}\right]^{1/\kappa}} \quad \text{for} \quad \forall i\in\Lambda. \tag{481}$$

We utilize the inequality (481) with $p=0$ for the estimation of the norm $\left\|\tilde{\Pi}^{\rm c}_{(L,N)}O_{X_0}(\tau_2)\sqrt{\rho_0(\tau_1)}\right\|_F$. For simplicity, we here adopt the notation of

$$\bar{N}_i := \begin{cases} N & \text{for} \quad i\in L, \\ Ne^{d_{i,L}/2} & \text{for} \quad i\in L^{\rm c}, \end{cases} \tag{482}$$

and obtain

$$\begin{aligned}\left\|\tilde{\Pi}^{\rm c}_{(L,N)}O_{X_0}(\tau_2)\sqrt{\rho_0(\tau_1)}\right\|_F &\le \sum_{i\in\Lambda}\left\|\Pi_{i,>\bar{N}_i}O_{X_0}(\tau_2)\sqrt{\rho_0(\tau_1)}\right\|_F \\ &\le \zeta_0\sum_{i\in\Lambda}\exp\left[-\frac{1}{2}\left(\frac{\bar{N}_i-32e^2q_0^3}{\xi_{t,R}}\right)^{1/\kappa}\right] \\ &\le \zeta_0\sum_{i\in\Lambda}e^{-[\bar{N}_i/(2\xi_{t,R})]^{1/\kappa}/2},\end{aligned} \tag{483}$$

where in the last inequality we use the condition $N\ge 64e^2q_0^3$ in (468) to derive $32e^2q_0^3 \le N/2 \le \bar{N}_i/2$. For the summation in the RHS of (483), we have

$$\begin{aligned}&\sum_{i\in\Lambda}e^{-\left[\bar{N}_i/(2\xi_{t,R})\right]^{1/\kappa}/2} = \sum_{i\in L}e^{-\left[N/(2^{\kappa+1}\xi_{t,R})\right]^{1/\kappa}} + \sum_{i\in L^{\rm c}}e^{-[Ne^{d_{i,L}/2}/(2^{\kappa+1}\xi_{t,R})]^{1/\kappa}} \\ &\le |L|e^{-\left[N/(2^{\kappa+1}\xi_{t,R})\right]^{1/\kappa}} + \gamma|L|2^{2D+2}D!e^{-\left[N/(2^{\kappa+1}\xi_{t,R})\right]^{1/\kappa}/(2\kappa)} \\ &\le 2^{2D+3}\gamma|L|D!e^{-[N/(2\xi_{t,R})]^{1/\kappa}/(4\kappa)} \quad \text{for} \quad N\ge 2^{\kappa+1}(2\kappa)^\kappa\xi_{t,R} = 2(4\kappa)^\kappa\xi_{t,R},\end{aligned} \tag{484}$$

where we apply the inequality (13) in Lemma 1 to the second term in the first line by choosing

$$\kappa\to\kappa, \quad \Phi\to 2^{\kappa+1}\xi_{t,R}, \quad q\to N, \quad \ell\to 0, \quad \xi\to 2. \tag{485}$$

Note that $N\ge 2(4\kappa)^\kappa\xi_{t,R}$ originates from the condition in (13), i.e., $q\ge(\xi\kappa)^\kappa\Phi$. By applying the inequality (484) to (483), we prove the first inequality (471).

[*4] For a general Schatten p norm, one can prove the generalized Hölder inequality (see Prop. 2.5 in Ref. [4]):

$$\left\|\prod_{j=1}^s O_j\right\|_p \le \prod_{j=1}^s \|O_j\|_{p_j}, \tag{477}$$

where $\sum_{j=1}^s 1/p_j = 1/p$. The inequality immediately yields

$$\|O_1O_2\|_1 \le \|O_1\|_2\|O_2\|_2, \tag{478}$$

where we set $p=1$, $p_1=2$ and $p_2=2$ in (477).

Second, we estimate the norm of $\left\|\tilde{\Pi}_{(L,N)} H_0 \tilde{\Pi}^{\rm c}_{(L,N)} O_{X_0}(\tau_2)\sqrt{\rho_0(\tau_1)}\right\|_F$. By using the same inequality as (461), we obtain

$$\begin{aligned}\left\|\tilde{\Pi}_{(L,N)} H_0 \tilde{\Pi}^{\rm c}_{(L,N)} O_{X_0}(\tau_2)\sqrt{\rho_0(\tau_1)}\right\|_F &\le \bar{J}\sum_{i\in\Lambda}\sum_{j:d_{i,j}=1}\left\|\tilde{\Pi}_{(L,N)}(b_i b_j^\dagger + {\rm h.c.})\left(\Pi_{i,\le\bar{N}_i}\Pi_{j,\le\bar{N}_j}\right)^{\rm c} O_{X_0}(\tau_2)\sqrt{\rho_0(\tau_1)}\right\|_F \\ &\le 2\bar{J}\sum_{i\in\Lambda}\sum_{j:d_{i,j}=1}\left\|(\hat{n}_i+\hat{n}_j)\left(\Pi_{i,\le\bar{N}_i}\Pi_{j,\le\bar{N}_j}\right)^{\rm c} O_{X_0}(\tau_2)\sqrt{\rho_0(\tau_1)}\right\|_F. \end{aligned} \tag{486}$$

Furthermore, from the same inequality as (462), we obtain

$$\begin{aligned}&\left\|(\hat{n}_i+\hat{n}_j)\left(\Pi_{i,\le\bar{N}_i}\Pi_{j,\le\bar{N}_j}\right)^{\rm c} O_{X_0}(\tau_2)\sqrt{\rho_0(\tau_1)}\right\|_F^2 \\ &= {\rm tr}\left[(\hat{n}_i+\hat{n}_j)^2\left(\Pi_{i,\le\bar{N}_i}\Pi_{j,\le\bar{N}_j}\right)^{\rm c} O_{X_0}(\tau_2)\rho_0(\tau_1)O_{X_0}(\tau_2)^\dagger\right] \\ &\le {\rm tr}\left[\hat{n}_{i[1]}^2\Pi_{i,>\bar{N}_i}O_{X_0}(\tau_2)\rho_0(\tau_1)O_{X_0}(\tau_2)^\dagger\right] + {\rm tr}\left[\hat{n}_{j[1]}^2\Pi_{j,>\bar{N}_j}O_{X_0}(\tau_2)\rho_0(\tau_1)O_{X_0}(\tau_2)^\dagger\right] \\ &\le \zeta_0^2\left(\bar{N}_i^2 e^{-\left[(\bar{N}_i-32e^2q_0^3)/\xi_{t,R}\right]^{1/\kappa}} + \bar{N}_j^2 e^{-\left[(\bar{N}_j-32e^2q_0^3)/\xi_{t,R}\right]^{1/\kappa}}\right),\end{aligned} \tag{487}$$

where we use $\hat{n}_i+\hat{n}_j \preceq \hat{n}_{i[1]}$ ($\hat{n}_i+\hat{n}_j \preceq \hat{n}_{j[1]}$) from $d_{i,j}=1$ in the first inequality, and in the second inequality, we apply the inequality (481) with $p=2$. By combining the inequalities (486) and (487), we have

$$\begin{aligned}&\left\|\tilde{\Pi}_{(L,N)} H_0 \tilde{\Pi}^{\rm c}_{(L,N)} O_{X_0}(\tau_2)\sqrt{\rho_0(\tau_1)}\right\|_F \\ &\le 2\zeta_0\bar{J}\sum_{i\in\Lambda}\sum_{j:d_{i,j}=1}\left(\bar{N}_i e^{-\left[(\bar{N}_i-32e^2q_0^3)/\xi_{t,R}\right]^{1/\kappa}/2} + \bar{N}_j e^{-\left[(\bar{N}_j-32e^2q_0^3)/\xi_{t,R}\right]^{1/\kappa}/2}\right) \\ &\le 4\zeta_0\gamma\bar{J}\sum_{i\in\Lambda}\bar{N}_i e^{-\left[(\bar{N}_i-32e^2q_0^3)/\xi_{t,R}\right]^{1/\kappa}/2} \le 4\zeta_0\gamma\bar{J}\sum_{i\in\Lambda}\bar{N}_i e^{-\left[\bar{N}_i/(2^{\kappa+1}\xi_{t,R})\right]^{1/\kappa}},\end{aligned} \tag{488}$$

where we use $\sqrt{x^2+y^2} \le x+y$ in taking the square root of (487), $\sum_{j:d_{i,j}=1} 1 \le |i[1]| \le \gamma$ for $\forall i \in \Lambda$, and $32e^2q_0^3 \le N_i/2$ from the condition $N \ge 64e^2q_0^3$. Finally, by employing the similar analyses to (484), we upper-bound the summation in (488). For the purpose, we first apply the inequality (15) to $\bar{N}_i e^{-\left[\bar{N}_i/(2^{\kappa+1}\xi_{t,R})\right]^{1/\kappa}}$ by letting

$$s \to 1, \quad \kappa \to \kappa, \quad \Phi \to (2^{\kappa+1}\xi_{t,R}), \tag{489}$$

which yields

$$\begin{aligned}\bar{N}_i e^{-\left[\bar{N}_i/(2^{\kappa+1}\xi_{t,R})\right]^{1/\kappa}} &\le 2(2^\kappa\cdot 2^{\kappa+1}\xi_{t,R})^2\kappa\Gamma(2\kappa)e^{-\left[\bar{N}_i/(2^{\kappa+1}\xi_{t,R})\right]^{1/\kappa}/2} \\ &= c_\kappa\xi_{t,R}^2 e^{-\left[\bar{N}_i/(2^{\kappa+1}\xi_{t,R})\right]^{1/\kappa}/2} \quad \text{with} \quad c_\kappa := 2^{4\kappa+3}\kappa\Gamma(2\kappa).\end{aligned} \tag{490}$$

By using the above upper bound, we obtain

$$\begin{aligned}&\sum_{i\in\Lambda}\bar{N}_i e^{-\left[\bar{N}_i/(2^{\kappa+1}\xi_{t,R})\right]^{1/\kappa}} \le c_\kappa\xi_{t,R}^2\sum_{i\in L}e^{-\left[N/(2^{\kappa+1}\xi_{t,R})\right]^{1/\kappa}/2} + c_\kappa\xi_{t,R}^2\sum_{i\in L^{\rm c}}e^{-\left[Ne^{d_{i,L}/2}/(2^{\kappa+1}\xi_{t,R})\right]^{1/\kappa}/2} \\ &= c_\kappa\xi_{t,R}^2|L|e^{-\left[N/(2^{\kappa+1}\xi_{t,R})\right]^{1/\kappa}/2} + c_\kappa\xi_{t,R}^2\sum_{i\in L^{\rm c}}e^{-\left[Ne^{d_{i,L}/2}/(2^{2\kappa+1}\xi_{t,R})\right]^{1/\kappa}} \\ &\le c_\kappa\xi_{t,R}^2|L|e^{-\left[N/(2^{\kappa+1}\xi_{t,R})\right]^{1/\kappa}/2} + c_\kappa\xi_{t,R}^2\gamma|L|2^{2D+2}D!e^{-\left[N/(2^{2\kappa+1}\xi_{t,R})\right]^{1/\kappa}/(2\kappa)} \\ &\le 2^{2D+3}c_\kappa\xi_{t,R}^2\gamma|L|D!e^{-\left[N/(2\xi_{t,R})\right]^{1/\kappa}/(8\kappa)} \quad \text{for} \quad N \ge 2^{2\kappa+1}(2\kappa)^\kappa\xi_{t,R} = 2(8\kappa)^\kappa\xi_{t,R},\end{aligned} \tag{491}$$

where in the second inequality, we use the inequality (13) with

$$\kappa \to \kappa, \quad \Phi \to 2^{2\kappa+1}\xi_{t,R}, \quad q \to N, \quad \ell \to 0, \quad \xi \to 2. \tag{492}$$

Here, the condition $N \ge 2(8\kappa)^\kappa\xi_{t,R}$ in (491) originates from the condition $q \ge (\xi\kappa)^\kappa\Phi$ in (13). By applying the inequality (491) to (488), we prove the desired inequality (472).

D. Effective Hamiltonian by the truncation of the boson number: third result

In this section, we further consider the projection $\Pi_{L',\le Q}$ onto the region L' such that (see Fig. 13)

$$L' \subseteq i_1[\ell_1 - 1] \quad (\ell_1 \ge \bar{\ell}_{t,R}), \quad d_{i_1[\ell_1],X_0} \ge \bar{\ell}_{t,R} \to i_1[\ell_1] \subset X_0[\bar{\ell}_{t,R}]^{\rm c}. \tag{493}$$

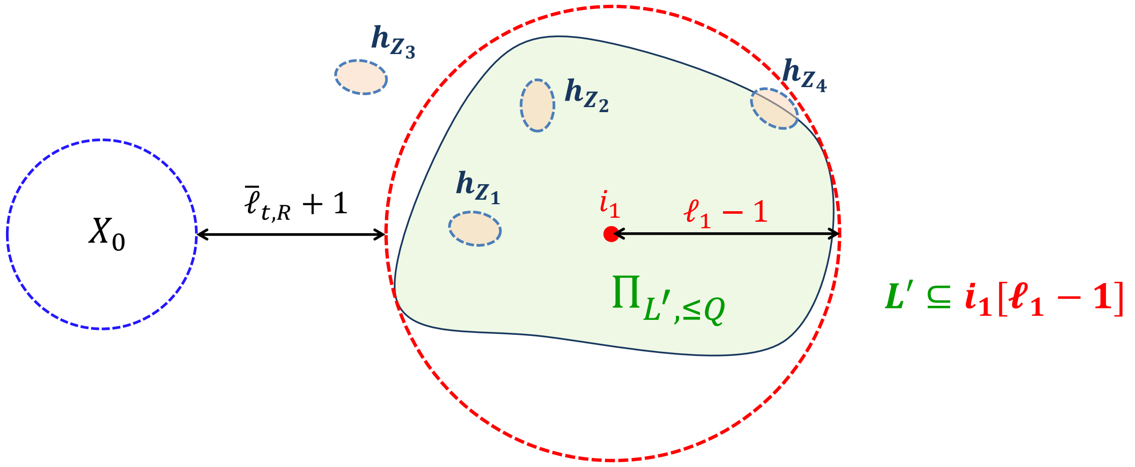

Supplementary Figure 13. Definition of the projection $\Pi_{L',\leq Q}$. In the projection, we truncate the boson number in the region L' up to Q, i.e., $\|\hat{n}_L \Pi_{L',\leq Q}\| = Q$, where the region $i_1[\ell_1]$ $(\supset L')$ is separated from the region X_0 at least by the distance of $\bar{\ell}_{t,R}$. Unlike the other projections $\bar{\Pi}_{L,\bar{q}}$ and $\tilde{\Pi}_{(L,N)}$ in Figs. 11 and 12, where the boson truncations are performed for each of the local sites, the boson truncation by $\Pi_{L',\leq Q}$ is non-local in the sense that it cannot be decomposed into the product form like $\prod_{i\in L'} \Pi_{i,\leq q_i}$. This point breaks down the locality of the effective Hamiltonian around the boundary. In the figure, the interactions h_{Z_1}, h_{Z_2} and h_{Z_3} commute with each other by the projection $\Pi_{L',\leq Q}$, while the boundary interaction h_{Z_4} becomes non-local by the projection $\Pi_{L',\leq Q}$. In Proposition 32, we consider the projection $\Pi_{L',\leq Q}\tilde{\Pi}_{(L,N)}$ and upper-bound the approximation error for dynamics by the effective Hamiltonian $\tilde{H}_{(L,N,L',Q)}$ in Eq. (494).

We then define the effective Hamiltonian $\tilde{H}_{(L,N,L',Q)}$ as

$$\tilde{H}_{(L,N,L',Q)} = \Pi_{L',\leq Q}\tilde{\Pi}_{(L,N)} H \tilde{\Pi}_{(L,N)}\Pi_{L',\leq Q}, \tag{494}$$

where the projection $\tilde{\Pi}_{(L,N)}$ has been defined in Eq. (466).

Here, we should note that, in general, the effective Hamiltonian $\tilde{H}_{(L,N,L',Q)}$ is no longer local in the sense that

$$[\Pi_{L',\leq Q} h_Z \Pi_{L',\leq Q}, \Pi_{L',\leq Q} h_{Z'} \Pi_{L',\leq Q}] \neq 0 \quad \text{for} \quad Z\cap Z' \neq \emptyset, \tag{495}$$

where $H=\sum_Z h_Z$ as in Eq. (26). To see the point, let us consider four interaction terms h_{Z_1}, h_{Z_2}, h_{Z_3} and h_{Z_4} such that (see Fig. 13)

$$Z_1, Z_2 \subset L' \quad (Z_1\cap Z_2=\emptyset), \quad Z_3 \subset L'^{\mathrm{c}}, \quad (Z_4\cap L') \neq \emptyset \ \& \ (Z_4 \cap L'^{\mathrm{c}}) \neq \emptyset.$$

Here, the effective interaction terms $\Pi_{L',\leq Q} h_{Z_1} \Pi_{L',\leq Q}$, $\Pi_{L',\leq Q} h_{Z_2} \Pi_{L',\leq Q}$ and $\Pi_{L',\leq Q} h_{Z_3} \Pi_{L',\leq Q}$ commute with each other since

$$[\Pi_{L',\leq Q}, h_{Z_i}] = 0 \quad \text{for} \quad i=1,2,3. \tag{496}$$

It is trivially obtained from $[\hat{n}_i, \Pi_{L',\leq Q}]=0$ for $\forall i\in\Lambda$ and $[b_i b_j^\dagger, \Pi_{L',\leq Q}]=0$ as long as $i,j\in L'$. We thus obtain

$$[\Pi_{L',\leq Q} h_{Z_i} \Pi_{L',\leq Q}, \Pi_{L',\leq Q} h_{Z_j} \Pi_{L',\leq Q}] = \Pi_{L',\leq Q}[h_{Z_i}, h_{Z_j}]\Pi_{L',\leq Q} = 0 \tag{497}$$

for $i,j=1,2,3$. On the other hand, we cannot ensure the above equation for h_{Z_4} because of

$$[\Pi_{L',\leq Q}, h_{Z_4}] \neq 0. \tag{498}$$

This point leads to the breakdown of the locality of the interactions as in (495). We also discuss this point in Sec. Supplementary Note 8 F when we consider the Lieb-Robinson bound for the effective Hamiltonian.

From Eq. (497), we at least prove the following corollary:

Corollary 31. *If we consider a subset Hamiltonian H_X such that $X\subseteq L'$ or $X\subseteq L'^{\mathrm{c}}$, the effective Hamiltonian $\tilde{H}_{X,(L,N,L',Q)}$ is still a local Hamiltonian in the sense that*

$$[\Pi_{L',\leq Q} h_Z \Pi_{L',\leq Q}, \Pi_{L',\leq Q} h_{Z'} \Pi_{L',\leq Q}] = 0 \quad \textit{for} \quad Z\cap Z' = \emptyset, \tag{499}$$

where h_Z and h'_Z are local interaction terms in H_X, i.e., $Z, Z'\subset X$.

On the approximation error for the effective Hamiltonian $\tilde{H}_{(L,N,L',Q)}$, we prove the following proposition:

Proposition 32. *We adopt the same assumption as Eq. (468) for N. Let us choose the subset L' such that the condition (493) is satisfied. Then, the approximation error using the effective Hamiltonian $\tilde{H}_{(L,N)}$ is upper-bounded by*

$$\begin{aligned}&\left\|\left[O_{X_0}(H,t)-O_{X_0}(\tilde{H}_{(L,N,L',Q)},t)\right]\rho_0\right\|_1\\&\le 2^{2D+5}\zeta_0\gamma|L|D!\left(1+2c_\kappa\gamma\bar{J}t\xi_{t,R}^2\right)e^{-[N/(2\xi_{t,R})]^{1/\kappa}/(8\kappa)}+4\zeta_0(1+2t\gamma\bar{J}Q)e^{-\left[Q/(4b_0\ell_1^D)\right]^{1/\kappa}/2},\end{aligned}\tag{500}$$

where c_κ has been defined in Proposition 30. Moreover, for $\tilde{\Pi}_{(L,N,L',Q)}\rho_0\tilde{\Pi}_{(L,N,L',Q)}$, we obtain the upper bound of

$$\begin{aligned}&\left\|\left[O_{X_0}(H,t)-O_{X_0}(\tilde{H}_{(L,N,L',Q)},t)\right]\tilde{\Pi}_{(L,N,L',Q)}\rho_0\tilde{\Pi}_{(L,N,L',Q)}\right\|_1\\&\le 2^{2D+6}\zeta_0\gamma|L|D!\left(1+c_\kappa\gamma\bar{J}t\xi_{t,R}^2\right)e^{-[N/(2\xi_{t,R})]^{1/\kappa}/(8\kappa)}+8\zeta_0(1+t\gamma\bar{J}Q)e^{-\left[Q/(4b_0\ell_1^D)\right]^{1/\kappa}/2}.\end{aligned}\tag{501}$$

1. *Proof of Proposition 32*

In the proof, we denote

$$\tilde{\Pi}_{(L,N,L',Q)}=\Pi_{L',\le Q}\tilde{\Pi}_{(L,N)}.\tag{502}$$

In applying Corollary 26, we aim to estimate the norms of

$$\left\|\tilde{\Pi}^{\rm c}_{(L,N,L',Q)}O_{X_0}(\tau_2)\sqrt{\rho_0(\tau_1)}\right\|_F\tag{503}$$

and

$$\left\|\tilde{\Pi}_{(L,N,L',Q)}H_0\tilde{\Pi}^{\rm c}_{(L,N,L',Q)}O_{X_0}(\tau_2)\sqrt{\rho_0(\tau_1)}\right\|_F\tag{504}$$

for arbitrary $|\tau_1|,|\tau_2|\le t$.

First, we have the decomposition of

$$\tilde{\Pi}^{\rm c}_{(L,N,L',Q)}=\Pi^{\rm c}_{L',\le Q}\tilde{\Pi}_{(L,N)}+\Pi_{L',\le Q}\tilde{\Pi}^{\rm c}_{(L,N)}+\Pi^{\rm c}_{L',\le Q}\tilde{\Pi}^{\rm c}_{(L,N)}=\Pi^{\rm c}_{L',\le Q}\tilde{\Pi}_{(L,N)}+\tilde{\Pi}^{\rm c}_{(L,N)},\tag{505}$$

where we use $\Pi_{L',\le Q}+\Pi^{\rm c}_{L',\le Q}=1$ in the second equation. By using the above, we upper-bound Eqs. (503) and (504) as follows:

$$\left\|\tilde{\Pi}^{\rm c}_{(L,N,L',Q)}O_{X_0}(\tau_2)\sqrt{\rho_0(\tau_1)}\right\|_F\le\left\|\tilde{\Pi}^{\rm c}_{(L,N)}O_{X_0}(\tau_2)\sqrt{\rho_0(\tau_1)}\right\|_F+\left\|\Pi^{\rm c}_{L',\le Q}O_{X_0}(\tau_2)\sqrt{\rho_0(\tau_1)}\right\|_F,\tag{506}$$

and

$$\begin{aligned}&\left\|\tilde{\Pi}_{(L,N,L',Q)}H_0\tilde{\Pi}^{\rm c}_{(L,N,L',Q)}O_{X_0}(\tau_2)\sqrt{\rho_0(\tau_1)}\right\|_F\\&\le\left\|\tilde{\Pi}_{(L,N,L',Q)}H_0\Pi^{\rm c}_{L',\le Q}\tilde{\Pi}_{(L,N)}O_{X_0}(\tau_2)\sqrt{\rho_0(\tau_1)}\right\|_F+\left\|\tilde{\Pi}_{(L,N,L',Q)}H_0\tilde{\Pi}^{\rm c}_{(L,N)}O_{X_0}(\tau_2)\sqrt{\rho_0(\tau_1)}\right\|_F\\&\le\left\|\Pi_{L',\le Q}H_0\Pi^{\rm c}_{L',\le Q}\tilde{\Pi}_{(L,N)}O_{X_0}(\tau_2)\sqrt{\rho_0(\tau_1)}\right\|_F+\left\|\tilde{\Pi}_{(L,N)}H_0\tilde{\Pi}^{\rm c}_{(L,N)}O_{X_0}(\tau_2)\sqrt{\rho_0(\tau_1)}\right\|_F.\end{aligned}\tag{507}$$

We have already upper-bounded $\left\|\tilde{\Pi}^{\rm c}_{(L,N)}O_{X_0}(\tau_2)\sqrt{\rho_0(\tau_1)}\right\|_F$ and $\left\|\tilde{\Pi}_{(L,N)}H_0\tilde{\Pi}^{\rm c}_{(L,N)}O_{X_0}(\tau_2)\sqrt{\rho_0(\tau_1)}\right\|_F$ in the inequalities (471) and (472), respectively. Therefore, we need to estimate the norms of $\left\|\Pi^{\rm c}_{L',\le Q}O_{X_0}(\tau_2)\sqrt{\rho_0(\tau_1)}\right\|_F$ and $\left\|\Pi_{L',\le Q}H_0\Pi^{\rm c}_{L',\le Q}\tilde{\Pi}_{(L,N)}O_{X_0}(\tau_2)\sqrt{\rho_0(\tau_1)}\right\|_F$, which are upper-bounded by the following inequalities (see below for the proofs):

$$\left\|\Pi^{\rm c}_{L',\le Q}O_{X_0}(\tau_2)\sqrt{\rho_0(\tau_1)}\right\|_F\le\zeta_0e^{-\left[Q/(4b_0\ell_1^D)\right]^{1/\kappa}/2},\tag{508}$$

and

$$\left\|\Pi_{L',\le Q}H_0\Pi^{\rm c}_{L',\le Q}\tilde{\Pi}_{(L,N)}O_{X_0}(\tau_2)\sqrt{\rho_0(\tau_1)}\right\|_F\le 4\zeta_0\gamma\bar{J}Qe^{-\left[Q/(4b_0\ell_1^D)\right]^{1/\kappa}/2}.\tag{509}$$

By applying the upper bounds in the inequalities (471), (472), (508) and (509) to to Corollary 26, we obtain the upper bound of

$$\begin{aligned}&\left\|\left[O_{X_0}(H,t)-O_{X_0}(\tilde{H}_{(L,N,L',Q)},t)\right]\rho_0\right\|_1\\&\le 2^{2D+5}\zeta_0\gamma|L|D!\left(1+2c_\kappa\gamma\bar{J}t\xi_{t,R}^2\right)e^{-[N/(2\xi_{t,R})]^{1/\kappa}/(8\kappa)}+\zeta_0(4+8t\gamma\bar{J}Q)e^{-\left[Q/(4b_0\ell_1^D)\right]^{1/\kappa}/2},\end{aligned}\tag{510}$$

where we use the inequality (473) to estimate the contributions from (471) and (472). We thus prove the main inequality (500).

In deriving the inequality (501), we only have to estimate [see also (475)]

$$\left\|O_{X_0}(\tilde{H}_{(L,N,L',Q)},t)\left(\rho_0-\tilde{\Pi}_{(L,N,L',Q)}\rho_0\tilde{\Pi}_{(L,N,L',Q)}\right)\right\|_1 \le 2\zeta_0\left\|\tilde{\Pi}^{\rm c}_{(L,N,L',Q)}\sqrt{\rho_0}\right\|_F \le 2\zeta_0\left(\left\|\tilde{\Pi}^{\rm c}_{(L,N)}\sqrt{\rho_0}\right\|_F+\left\|\Pi^{\rm c}_{L',\le Q}\sqrt{\rho_0}\right\|_F\right). \tag{511}$$

By applying the upper bounds (471) and (508) to the above inequality, we obtain the upper bound of

$$\left\|O_{X_0}(\tilde{H}_{(L,N,L',Q)},t)\left(\rho_0-\tilde{\Pi}_{(L,N,L',Q)}\rho_0\tilde{\Pi}_{(L,N,L',Q)}\right)\right\|_1 \le 2^{2D+4}\zeta_0\gamma|L|D!e^{-[N/(2\xi_{t,R})]^{1/\kappa}/(8\kappa)}+2\zeta_0 e^{-[Q/(4b_0\ell_1^D)]^{1/\kappa}/2}, \tag{512}$$

which yields the inequality (501) by combining the inequality (510). This completes the proof. □

[Proof of the inequalities (508) and (509)]

We can adopt similar techniques to the proof of Proposition 29. As in the inequality (457), we utilize the inequality (434) with $p=0$ in Lemma 28, which yields

$$\left\|\Pi^{\rm c}_{L',\le Q}O_{X_0}(\tau_2)\sqrt{\rho_0(\tau_1)}\right\|_F^2 = {\rm tr}\left[\Pi_{L',>Q}O_{X_0}(\tau_2)\rho_0(\tau_1)O_{X_0}(\tau_2)^\dagger\right] \le {\rm tr}\left[\Pi_{i_1[\ell_1],>Q}O_{X_0}(\tau_2)\rho_0(\tau_1)O_{X_0}(\tau_2)^\dagger\right] \le \zeta_0^2 e^{-[Q/(4b_0\ell_1^D)]^{1/\kappa}}, \tag{513}$$

where in the first inequality we use $\Pi_{L',>Q}=\Pi_{L',>Q}\Pi_{i_1[\ell_1],>Q}$ because of $L'\subseteq i_1[\ell_1-1]\subset i_1[\ell_1]$, and in the second inequality, we use $d_{i_1[\ell_1],X_0}\ge \bar{\ell}_{t,R}$ and $\ell_1\ge\bar{\ell}_{t,R}$. We thus prove the first inequality (508).

Next, to prove the second inequality (509), we first utilize the decomposition (458) as

$$\Pi_{L',\le Q}H_0\Pi^{\rm c}_{L',\le Q}=\Pi_{L',\le Q}\left(H_{0,L'}+\partial h_{L'}\right)\Pi^{\rm c}_{L',\le Q}. \tag{514}$$

We then obtain

$$\left\|\Pi_{L',\le Q}H_0\Pi^{\rm c}_{L',\le Q}\tilde{\Pi}_{(L,N)}O_{X_0}(\tau_2)\sqrt{\rho_0(\tau_1)}\right\|_F^2 = {\rm tr}\left[\left(H_{0,L'}+\partial h_{L'}\right)^\dagger\Pi_{L',\le Q}\left(H_{0,L'}+\partial h_{L'}\right)\Pi^{\rm c}_{L',\le Q}\tilde{\Pi}_{(L,N)}O_{X_0}(\tau_2)\rho_0(\tau_1)O_{X_0}(\tau_2)\tilde{\Pi}_{(L,N)}\Pi^{\rm c}_{L',\le Q}\right]. \tag{515}$$

To obtain an operator upper bound for $\left(H_{0,L'}+\partial h_{L'}\right)^\dagger\Pi_{L',\le Q}\left(H_{0,L'}+\partial h_{L'}\right)$, we first derive

$$|H_{0,L'}+\partial h_{L'}|\preceq \bar{J}\sum_{i\in L'}\sum_{j:d_{i,j}=1}\left(|b_ib_j^\dagger|+|b_i^\dagger b_j|\right)\preceq 2\bar{J}\sum_{i\in L'}\sum_{j:d_{i,j}=1}(\hat{n}_i+\hat{n}_j)\preceq 4\gamma\bar{J}\hat{n}_{L'[1]}, \tag{516}$$

where we use the operator inequality (459) in the second inequality, and the last inequality is derived from

$$\sum_{i\in L'}\sum_{j:d_{i,j}=1}\hat{n}_i\preceq\gamma\sum_{i\in L'}\hat{n}_i\preceq\gamma\hat{n}_{L'[1]},$$
$$\sum_{i\in L'}\sum_{j:d_{i,j}=1}\hat{n}_j\preceq\sum_{j\in L'[1]}\sum_{i:d_{i,j}=1}\hat{n}_j\preceq\gamma\sum_{j\in L'[1]}\hat{n}_j\preceq\gamma\hat{n}_{L'[1]}. \tag{517}$$

Note that $i\in L'$ and $j:d_{i,j}=1$ implies $j\in L'[1]$. Because $[|H_{0,L'}+\partial h_{L'}|,\hat{n}_{L'[1]}]=0$, we can obtain from (516)

$$\left(H_{0,L'}+\partial h_{L'}\right)^\dagger\Pi_{L',\le Q}\left(H_{0,L'}+\partial h_{L'}\right)\preceq|H_{0,L'}+\partial h_{L'}|^2\preceq 16\gamma^2\bar{J}^2\hat{n}^2_{L'[1]}, \tag{518}$$

where we use the fact that for arbitrary Hermitian operators A and B such that $|A|\preceq|B|$, we have $|A|^s\preceq|B|^s$ ($s\in\mathbb{N}$) as long as $[|A|,|B|]=0$[*5].

By combining the inequalities (515) and (518), we obtain

$$\begin{aligned}&\left\|\Pi_{L',\le Q}H_0\Pi^{\rm c}_{L',\le Q}\tilde{\Pi}_{(L,N)}O_{X_0}(\tau_2)\sqrt{\rho_0(\tau_1)}\right\|_F^2\\ &\le 16\gamma^2\bar{J}^2{\rm tr}\left[\hat{n}^2_{L'[1]}\Pi^{\rm c}_{L',\le Q}\tilde{\Pi}_{(L,N)}O_{X_0}(\tau_2)\rho_0(\tau_1)O_{X_0}(\tau_2)\tilde{\Pi}_{(L,N)}\Pi^{\rm c}_{L',\le Q}\right]\\ &\le 16\gamma^2\bar{J}^2{\rm tr}\left[\hat{n}^2_{L'[1]}\Pi_{L'[1],>Q}O_{X_0}(\tau_2)\rho_0(\tau_1)O_{X_0}(\tau_2)\right]\\ &\le 16\gamma^2\bar{J}^2{\rm tr}\left[\hat{n}^2_{i_1[\ell_1]}\Pi_{i_1[\ell_1],>Q}O_{X_0}(\tau_2)\rho_0(\tau_1)O_{X_0}(\tau_2)\right]\le 16\zeta_0^2\gamma^2\bar{J}^2Q^2e^{-[Q/(4b_0\ell_1^D)]^{1/\kappa}},\end{aligned} \tag{519}$$

where we use $L'[1]\subseteq i_1[\ell_1]$ from $L'\subseteq i_1[\ell_1-1]$ in the third inequality and apply the inequality (434) with $p=2$ in the last inequality. Note that we have assumed $d_{i_1[\ell_1],X_0}\ge\bar{\ell}_{t,R}$ (i.e., $i_1[\ell_1]\subset X_0[\bar{\ell}_{t,R}]^{\rm c}$).

[*5] In general, we cannot ensure $|A|^s\preceq|B|^s$ form $|A|\preceq|B|$ unless $[|A|,|B|]=0$. This point has made the proof of Subtheorem 1 rather challenging [see the discussion around Eq. (82)].

Supplementary Note 7. BOSONIC LIEB-ROBINSON BOUND: LOOSER RESULTS

Based on Proposition 20 (or Proposition 29), we can truncate the boson number on each of the sites up to $\mathcal{O}(\bar{\ell}_{t,R}^D) = \tilde{\mathcal{O}}(t^D)$ [see Eq. (433) for the definition of $\bar{\ell}_{t,R}$]. By such a truncation, we can upper-bound the local energy on each site by $\tilde{\mathcal{O}}(t^D)$. This energy upper bound allows us to derive the Lieb-Robinson bound with a velocity of $\tilde{\mathcal{O}}(t^D)$. However, as we will prove below, the optimal Lieb-Robinson velocity is given by $\tilde{\mathcal{O}}(t^{D-1})$ instead of $\tilde{\mathcal{O}}(t^D)$. Nevertheless, the following analyses are quite simple, and we can utilize them to develop a quantum algorithm to simulate the dynamics by quantum computer (see Result 3 in the main manuscript).

We here prove the following theorem:

Theorem 2. *Let us consider an initial state ρ_0 that satisfies Assumption 3 with $b_\rho = b_0/\gamma$ in (34). Also, we consider an arbitrary operator O_{X_0} ($X_0 \subseteq i_0[r_0]$[*6], $\|O_{X_0}\| = \zeta_0$) such that the condition (428) holds. Then, there exists a unitary operator $U_{X[R]}$ such that the following Lieb-Robinson bound holds:*

$$
\begin{aligned}
&\left\| \left[O_{X_0}(H,t) - U_{i_0[R]}^\dagger O_{X_0} U_{i_0[R]} \right] \rho_0 \right\|_1 \\
&\le 4\zeta_0 \gamma^2 R^D (1 + R/c_1) \exp\left[-\frac{1}{2} \left(\frac{R - r_0}{8 b_0 c_1 \bar{J} t \bar{\ell}_{t,R}^D} \right)^{1/\kappa} \right] + \zeta_0 R^{D+1} e^{-(R-r_0)/(4k)},
\end{aligned}
\tag{520}
$$

where $c_1 := 2^{6-D} e^2 k \gamma^3 (2k)^{2D}$ and we assume $R \ge \max\left[r_0 + 16k, r_0 + 2(\bar{\ell}_{t,R} + 1)\right]$.

Remark. By using the Θ notation in Eq. (9), we can express the inequality (520) as

$$
\begin{aligned}
\left\| \left[O_{X_0}(H,t) - U_{i_0[R]}^\dagger O_{X_0} U_{i_0[R]} \right] \rho_0 \right\|_1 &\le \zeta_0 \Theta(R^{D+1}) \left\{ e^{-[\Theta(R-r_0)/(t\bar{\ell}_{t,R}^D)]^{1/\kappa}} + e^{-(R-r_0)/(4k)} \right\} \\
&\le \zeta_0 \exp\left[-\Theta\left(\frac{R - r_0}{t \bar{\ell}_{t,R}^D} \right)^{1/\kappa} + \Theta(\log(R)) \right].
\end{aligned}
\tag{521}
$$

Because of $\bar{\ell}_{t,R} \propto t\log(Rq_0)$, for $r_0 = \mathcal{O}(1)$ and $q_0 = \mathrm{poly}(R)$, the approximation error becomes sufficiently small as long as

$$
R \propto t^{D+1} \log^{D+1}(t),
\tag{522}
$$

which gives the Lieb-Robinson velocity of $\tilde{\mathcal{O}}(t^D)$.

On the unitary operator $U_{i_0[R]}$, we show the explicit form in the inequality (533), which is constructed by using the effective Hamiltonian. The construction only depends on the interaction terms within the region of $i_0[R]$. Hence, when we consider the subset Hamiltonian $H_{i_0[R]}$, we obtain the same inequality as (520) for $\left\| \left[O_{X_0}(H_{i_0[R]},t) - U_{i_0[R]}^\dagger O_{X_0} U_{i_0[R]} \right] \rho_0 \right\|_1$ by using the same unitary operator $U_{i_0[R]}$, which yields

$$
\begin{aligned}
&\left\| \left[O_{X_0}(H,t) - O_{X_0}(H_{i_0[R]},t) \right] \rho_0 \right\|_1 \\
&\le \left\| \left[O_{X_0}(H,t) - U_{i_0[R]}^\dagger O_{X_0} U_{i_0[R]} \right] \rho_0 \right\|_1 + \left\| \left[O_{X_0}(H_{i_0[R]},t) - U_{i_0[R]}^\dagger O_{X_0} U_{i_0[R]} \right] \rho_0 \right\|_1 \\
&\le 8\zeta_0 \gamma^2 R^D (1 + R/c_1) \exp\left[-\frac{1}{2} \left(\frac{R - r_0}{8 b_0 c_1 \bar{J} t \bar{\ell}_{t,R}^D} \right)^{1/\kappa} \right] + 2\zeta_0 R^{D+1} e^{-(R-r_0)/(4k)}.
\end{aligned}
\tag{523}
$$

Therefore, we can obtain the approximation for $O_{X_0}(H,t)$ by simply considering the time evolution by the subset Hamiltonian $H_{i_0[R]}$ on the region $i_0[R]$.

A. Proof of Theorem 2: basic strategy

For the proof, we consider the projection operator $\bar{\Pi}_{\tilde{X}_0,\bar{q}}$ (see Fig. 14), where we use the notation in Eq. (452), i.e.,

$$
\bar{\Pi}_{\tilde{X}_0,\bar{q}} := \prod_{i \in \tilde{X}_0} \Pi_{i,\le \bar{q}}, \quad \tilde{X}_0 := X_0[2\ell_0] \setminus X_0[\ell_0] \quad (\ell_0 \ge 8k),
\tag{524}
$$

[*6] Roughly speaking, r_0 is the spatial size of the operator O_{X_0}.

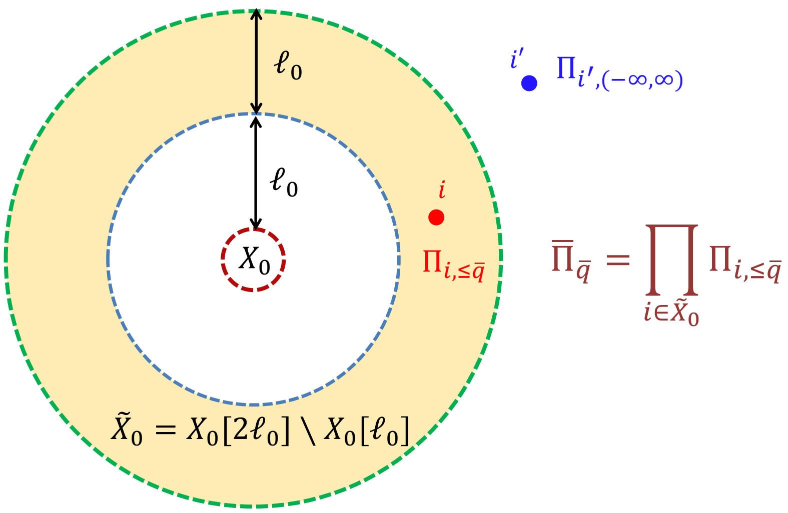

Supplementary Figure 14. Schematic picture of the region $\tilde{X}_0$. In this region, we truncate the boson number up to $\bar{q}$ and define the projection as $\bar{\Pi}_{\bar{q}}$. We first approximate the original dynamics e^{-iHt} by using the dynamics $e^{-i\bar{\Pi}_{\bar{q}}H\bar{\Pi}_{\bar{q}}t}$. Then, because the effective Hamiltonian $\bar{\Pi}_{\bar{q}}H\bar{\Pi}_{\bar{q}}$ has a finite local energy in the region $\tilde{X}_0$, we can derive the Lieb-Robinson bound and approximate $e^{-i\bar{\Pi}_{\bar{q}}H\bar{\Pi}_{\bar{q}}t}$ by $e^{-i\bar{\Pi}_{\bar{q}}H_{X_0[2\ell_0]}\bar{\Pi}_{\bar{q}}t}$.

where $X_0 \subseteq i_0[r_0]$ and

$$R = 2\ell_0 + r_0, \quad X_0[2\ell_0] \subseteq i_0[r_0 + 2\ell_0] = i_0[R]. \tag{525}$$

Note that the condition $R \ge r_0 + 16k$ implies $\ell_0 \ge 8k$. In the following, we simply denote it as

$$\bar{\Pi}_{\tilde{X}_0,\bar{q}} \to \bar{\Pi}_{\bar{q}} \tag{526}$$

by omitting the region dependence $\tilde{X}_0$. We choose $\bar{q}$ (≥ 2) such that

$$\bar{q} = \frac{\ell_0}{c_1 \bar{J} t}, \quad c_1 := 2^{6-D} e^2 k \gamma^3 (2k)^{2D}, \tag{527}$$

where the choice is utilized in ensuring the condition (554) below[*7].

The basic strategy for the proof is similar to the one in Ref. [1], and we summarize the tasks as follows:

1. We approximate the dynamics e^{-iHt} by using the effective Hamiltonian $\bar{\Pi}_{\bar{q}}H\bar{\Pi}_{\bar{q}}$; that is, our first task is to estimate the error of

$$\left\| \left[O_{X_0}(H,t) - O_{X_0}(\bar{\Pi}_{\bar{q}}H\bar{\Pi}_{\bar{q}}, t) \right] \rho_0 \right\|_1 . \tag{528}$$

2. For the time evolution of $O_{X_0}(\bar{\Pi}_{\bar{q}}H\bar{\Pi}_{\bar{q}}, t)$, we approximate the dynamics by using the Hamiltonian $\bar{\Pi}_{\bar{q}}H_{X_0[2\ell_0]}\bar{\Pi}_{\bar{q}}$ which is supported on $X_0[2\ell_0]$. Here, in the Hamiltonian $\bar{\Pi}_{\bar{q}}H\bar{\Pi}_{\bar{q}}$, the interaction terms are bounded in the region $\tilde{X}_0$ due to the truncation of the boson number. However, in the region $\tilde{X}_0^{\rm c}$, we still encounter the unboundedness of the norm of the interactions. In Ref. [1], the authors have developed analyses to derive the Lieb-Robinson bound for $e^{-i\bar{\Pi}_{\bar{q}}H\bar{\Pi}_{\bar{q}}t}$ only from the finite upper bound of the Hamiltonian norm in the region $\tilde{X}_0$.

We start from the estimation of the norm (528). The error is immediately obtained by using Proposition 29 with the choice of $L = \tilde{X}_0$ in (453):

$$\left\| \left[O_{X_0}(H,t) - O_{X_0}(\bar{\Pi}_{\bar{q}}H\bar{\Pi}_{\bar{q}}, t) \right] \rho_0 \right\|_1 \le 4\zeta_0 \gamma |\tilde{X}_0| \left(1 + \bar{J}t\bar{q}\right) e^{-\left[\bar{q}/(4b_0 \bar{\ell}_{t,R}^D)\right]^{1/\kappa}/2}, \tag{529}$$

[*7] Precisely speaking, in Eqs. (525) and (527) for the definitions of ℓ_0 and $\bar{q}$, we should define $\ell_0 = \lfloor (R - r_0)/2 \rfloor$ and $\bar{q} = \lfloor \ell_0/(c_1 t) \rfloor$, but for simplicity we assume they become integers without the floor function.

where the following condition should be satisfied

$$\ell_0 = d_{X_0,\tilde{X}_0} \geq \bar{\ell}_{t,R} + 1. \tag{530}$$

By using $|\tilde{X}_0| \leq |X_0[2\ell_0]| \leq |i_0[R]| \leq \gamma R^D$, and $\bar{J}t\bar{q} = \ell_0/c_1$ $(\leq R/c_1)$, we reduce the inequality (529) to

$$\left\| \left[O_{X_0}(H,t) - O_{X_0}(\bar{\Pi}_{\bar{q}} H \bar{\Pi}_{\bar{q}}, t) \right] \rho_0 \right\|_1 \leq 4\zeta_0 \gamma^2 R^D \left(1 + R/c_1\right) \exp\left[-\frac{1}{2} \left(\frac{R - r_0}{8b_0 c_1 \bar{J} t \bar{\ell}_{t,R}^D} \right)^{1/\kappa} \right], \tag{531}$$

where we use $\ell_0 = (R - r_0)/2$. Remember that the length $\bar{\ell}_{t,R}$ has been defined in Eq. (433). Therefore, the remaining task is to derive the Lieb-Robinson bound for the effective Hamiltonian $\bar{\Pi}_{\bar{q}} H \bar{\Pi}_{\bar{q}}$. In detail, we aim to estimate the approximation error of

$$\left\| \left[O_{X_0}(\bar{\Pi}_{\bar{q}} H \bar{\Pi}_{\bar{q}}, t) - O_{X_0}(\bar{\Pi}_{\bar{q}} H_{X_0[\ell]} \bar{\Pi}_{\bar{q}}, t) \right] \rho_0 \right\|_1 \leq \left\| O_{X_0}(\bar{\Pi}_{\bar{q}} H \bar{\Pi}_{\bar{q}}, t) - O_{X_0}(\bar{\Pi}_{\bar{q}} H_{X_0[\ell]} \bar{\Pi}_{\bar{q}}, t) \right\| \tag{532}$$

for a particular choice of ℓ, where we use $\|O_1 O_2\|_1 \leq \|O_1\| \cdot \|O_2\|_1$ in the inequality. For this purpose, we prove the following lemma:

Lemma 33. *Under the choice of $\bar{q}$ as in Eq. (527), we prove that*

$$\left\| O_{X_0}(\bar{\Pi}_{\bar{q}} H \bar{\Pi}_{\bar{q}}, t) - O_{X_0}(\bar{\Pi}_{\bar{q}} H_{X_0[2\ell_0 - 2k]} \bar{\Pi}_{\bar{q}}, t) \right\| \leq \zeta_0 R^{D+1} e^{-(R-r_0)/(4k)} \tag{533}$$

for $\ell_0 \geq 8k$. We show the proof in Sec. Supplementary Note 7B.

Finally, by combining the two inequalities (531) and (533), we obtain the main inequality (520). The two conditions $\ell_0 \geq \bar{\ell}_{t,R} + 1$ in Eq. (530) and $\ell_0 \geq 8k$ in Lemma 33 is integrated by the condition $R \geq \max\left[r_0 + 16k, r_0 + 2(\bar{\ell}_{t,R} + 1)\right]$ in the main statement, where $\ell_0 = (R - r_0)/2$. This completes the proof of Theorem 2. □

B. Proof of Lemma 33: derivation of the Lieb-Robinson bound

We here calculate the norm of

$$\left\| O_{X_0}(\bar{\Pi}_{\bar{q}} H \bar{\Pi}_{\bar{q}}, t) - O_{X_0}(\bar{\Pi}_{\bar{q}} H_{X_0[2\ell_0]} \bar{\Pi}_{\bar{q}}, t) \right\|. \tag{534}$$

Now, the effective Hamiltonian $\bar{\Pi}_{\bar{q}} H_{X_0[2\ell_0]} \bar{\Pi}_{\bar{q}}$ is supported on $X_0[2\ell_0]$ as $\bar{\Pi}_{\bar{q}}$ is supported on $\tilde{X}_0 \subset X_0[2\ell_0]$. For the estimation of the norm (534), we can utilize the technique in Ref. [1, Supplementary Section S.V.C]. By using the interaction picture, we start from the decomposition of

$$e^{-i\bar{\Pi}_{\bar{q}} H \bar{\Pi}_{\bar{q}} t} = e^{-i\bar{\Pi}_{\bar{q}} V \bar{\Pi}_{\bar{q}} t} \mathcal{T} \exp\left(-i \int_0^t e^{i\bar{\Pi}_{\bar{q}} V \bar{\Pi}_{\bar{q}} \tau} \bar{\Pi}_{\bar{q}} H_0 \bar{\Pi}_{\bar{q}} e^{-i\bar{\Pi}_{\bar{q}} V \bar{\Pi}_{\bar{q}} \tau} d\tau \right). \tag{535}$$

The primary motivation to use the interaction picture lies in that we impose no assumptions on the norm of V [see Def. 1 and Table 2].

For simplicity, we adopt the notations of

$$\begin{aligned} &\bar{H} := \bar{\Pi}_{\bar{q}} H \bar{\Pi}_{\bar{q}}, \quad \bar{H}_0 := \bar{\Pi}_{\bar{q}} H_0 \bar{\Pi}_{\bar{q}}, \quad \bar{V} := \bar{\Pi}_{\bar{q}} V \bar{\Pi}_{\bar{q}}, \\ &\bar{H}_\tau := e^{i\bar{V}\tau} \bar{H}_0 e^{-i\bar{V}\tau}, \end{aligned} \tag{536}$$

which simplify Eq. (535) as

$$e^{-i\bar{H}t} = e^{-i\bar{V}t} \mathcal{T} e^{-i\int_0^t \bar{H}_\tau d\tau}. \tag{537}$$

We expand $\bar{H}_\tau$ as

$$\begin{aligned} &\bar{H}_\tau = \sum_{\langle i,j \rangle} J_{i,j} e^{i\bar{V}\tau} \bar{\Pi}_{\bar{q}} (b_i^\dagger b_j + \text{h.c.}) \bar{\Pi}_{\bar{q}} e^{-i\bar{V}\tau} = \sum_{\langle i,j \rangle} \bar{h}_{\mathcal{B}_{i,j},\tau}, \\ &\bar{h}_{\mathcal{B}_{i,j},\tau} := \bar{\Pi}_{\bar{q}} e^{i\bar{V}\tau} (b_i^\dagger b_j + \text{h.c.}) e^{-i\bar{V}\tau} \bar{\Pi}_{\bar{q}}, \quad \mathcal{B}_{i,j} = i[k-1] \cup j[k-1], \quad \text{diam}(\mathcal{B}_{i,j}) \leq 2k, \end{aligned} \tag{538}$$

where use the fact that the operator $e^{i\bar{V}\tau} b_i e^{-i\bar{V}\tau}$ is supported on the subset $i[k-1]$ because $\bar{V}$ has interaction length of k (see Def. 1). We here obtain

$$\left\| \bar{h}_{\mathcal{B}_{i,j},\tau} \right\| \leq |J_{i,j}| \cdot \left\| \bar{\Pi}_{\bar{q}} (b_i^\dagger b_j + \text{h.c.}) \bar{\Pi}_{\bar{q}} \right\| \leq 4\bar{J}\bar{q} \quad (i, j \in \tilde{X}_0), \tag{539}$$

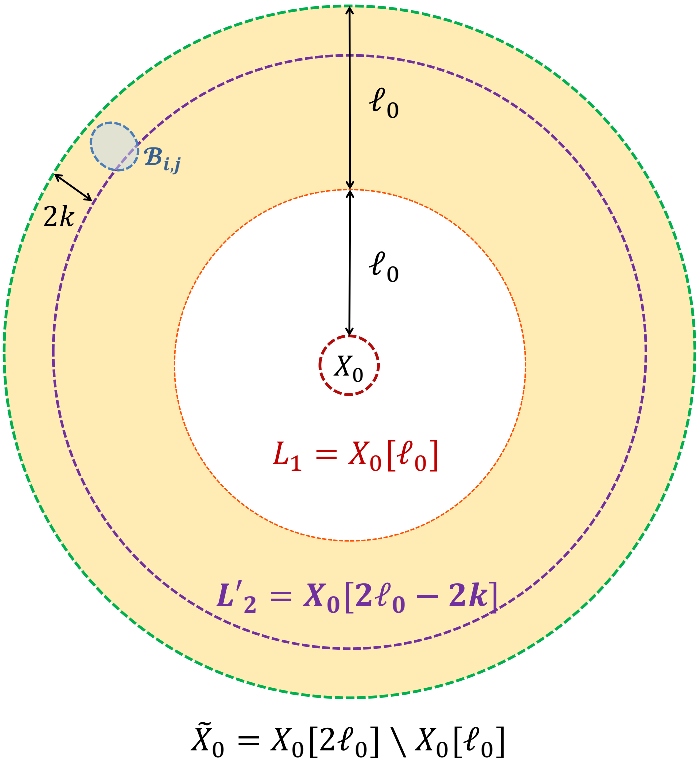

Supplementary Figure 15. Schematic picture for the positions of the subsets X_0, $\tilde{X}_0$, L_1 and L'_2. We here consider the time evolution of O_{X_0} for the decomposed unitary operator (537). Then, as in Eq. (549), the contribution from the interaction terms in V is included in the subset $X_0[k] \subseteq X_0[\ell_0] = L_1$. To treat the contribution from the interactions $H_\tau = \sum_{\langle i,j\rangle} \bar{h}_{\mathcal{B}_{i,j},\tau}$ in Eq. (538), we utilize Lemma 34. From the lemma, the time evolution $\bar{O}_{L_1}(\bar{H}_\tau, 0 \to t)$ is approximated by $\bar{O}_{L_1}\left(\bar{H}_{L'_2,\tau}, 0 \to t\right)$ with $L'_2 = X_0[2\ell_0 - 2k]$, where we should consider the time evolution of the boundary interaction terms $\bar{h}_{\mathcal{B}_{i,j},\tau}$ ($\mathcal{B}_{i,j} \in \mathcal{S}_{L'_2}$) as shown in the inequality (551). From the choice of L'_2, we can always ensure that any interaction term $\bar{h}_{\mathcal{B}_{i,j},\tau}$ in $\mathcal{S}_{L'_2}$ is supported on the region $\tilde{X}_0$.

where we use the inequality (459) to derive

$$\left\|\bar{\Pi}_{\bar{q}} b_i^\dagger b_j \bar{\Pi}_{\bar{q}}\right\| \le \left\|\bar{\Pi}_{\bar{q}} \left(\hat{n}_i + \hat{n}_j\right) \bar{\Pi}_{\bar{q}}\right\| \le 2\bar{q}. \tag{540}$$

Then, by using the inequality (539) and the upper bound of

$$\max_{i_0 \in \Lambda} \# \{\langle i,j\rangle | \mathcal{B}_{i,j} \ni i_0\} \le \gamma \max_{i_0 \in \Lambda} (|i_0[k]|) \le \gamma^2 k^D, \tag{541}$$

we obtain

$$\max_{i_0 \in \Lambda} \sum_{\substack{\langle i,j\rangle \\ \{i,j\} \subset \tilde{X}_0,\ \mathcal{B}_{i,j} \ni i_0}} \left\|\bar{h}_{\mathcal{B}_{i,j},\tau}\right\| \le 4\bar{J}\bar{q} \cdot \gamma^2 k^D = c_3 \ell_0, \tag{542}$$

where c_3 is defined as

$$c_3 := \frac{4\bar{J}\gamma^2 k^D \bar{q}}{\ell_0}. \tag{543}$$

In the following, we utilize the following established lemma:

Lemma 34 (Supplementary Lemma 14 in Ref. [1])**.** *Let H_τ be an arbitrary time-dependent Hamiltonian in the form of*

$$H_\tau = \sum_{Z \subset \Lambda} h_{Z,\tau}. \tag{544}$$

We also write a subset Hamiltonian on L as follows:

$$H_{L,\tau} = \sum_{Z: Z \subseteq L} h_{Z,\tau}. \tag{545}$$

Then, for an arbitrary subset L such that $L \supseteq X$, we obtain

$$\|O_X(H_\tau, 0 \to t) - O_X(H_{L,\tau}, 0 \to t)\| \le \sum_{Z: Z \in \mathcal{S}_L} \int_0^t \|[h_{Z,x}(H_{L,\tau}, x \to 0), O_X]\| \, dx, \tag{546}$$

where $\mathcal{S}_L$ is defined as a set of subsets $\{Z\}_{Z\subseteq\Lambda}$, which overlap the surface region of L:

$$\mathcal{S}_L := \{Z \subseteq \Lambda | Z \cap L \neq \emptyset, Z \cap L^{\mathrm{c}} \neq \emptyset\}. \tag{547}$$

Note that the notation $h_{Z,x}(H_{L,\tau}, x \to 0)$ has been defined in Eq. (33).

By using the decomposition (537), we now obtain (see Fig. 15)

$$O_{X_0}(\bar{H}, t) = \bar{O}_{L_1}(\bar{H}_\tau, 0 \to t), \quad L_1 = X_0[\ell_0], \tag{548}$$

where we define

$$\bar{O}_{L_1} = e^{i\bar{\Pi}_{\bar{q}} V \bar{\Pi}_{\bar{q}} t} O_{X_0} e^{-i\bar{\Pi}_{\bar{q}} V \bar{\Pi}_{\bar{q}} t} = e^{i\bar{\Pi}_{\bar{q}} V_{X_0[k]} \bar{\Pi}_{\bar{q}} t} O_{X_0} e^{-i\bar{\Pi}_{\bar{q}} V_{X_0[k]} \bar{\Pi}_{\bar{q}} t}, \tag{549}$$

where $V_{X_0[k]}$ picks up all the interaction terms v_Z such that $Z \subset X_0[k]$. In deriving Eq. (549), we use $[\bar{\Pi}_{\bar{q}} v_Z \bar{\Pi}_{\bar{q}}, \bar{\Pi}_{\bar{q}} v_{Z'} \bar{\Pi}_{\bar{q}}] = 0$ for arbitrary Z, Z'. Also, because of $\mathrm{diam}(Z) \le k$, we have $[\bar{\Pi}_{\bar{q}} v_Z \bar{\Pi}_{\bar{q}}, O_{X_0}] \neq 0$ only if Z is included in $X_0[k]$ (i.e., $Z \subseteq X_0[k]$).

We, in the following, aim to approximate $\bar{O}_{L_1}(\bar{H}_\tau, 0 \to t)$ by $\bar{O}_{L_1}(\bar{H}_{L'_2,\tau}, 0 \to t)$ with

$$L'_2 = X_0[2\ell_0 - 2k]. \tag{550}$$

Lemma 34 immediately gives the following upper bound on the approximation error:

$$\left\|\bar{O}_{L_1}(\bar{H}_\tau, 0 \to t) - \bar{O}_{L_1}(\bar{H}_{L'_2,\tau}, 0 \to t)\right\| \le \sum_{i,j: \mathcal{B}_{i,j} \in \mathcal{S}_{L'_2}} \int_0^t \left\|[\bar{h}_{\mathcal{B}_{i,j},x}(\bar{H}_{L'_2,\tau}, x \to 0), \bar{O}_{L_1}]\right\| dx. \tag{551}$$

For an arbitrary operator O_Z, we have already derived the following lemma:

Lemma 35 (Supplementary Lemma 15 in Ref. [1])**.** *Let us choose the subset Z such that $Z \in \mathcal{S}_{L'_2}$ and* $\mathrm{diam}(Z) \le 2k$*. Then, for an arbitrary operator O_Z, we have the upper bound of*

$$\left\|[O_Z(\bar{H}_{L'_2,\tau}, x \to 0), \bar{O}_{L_1}]\right\| \le 2e^3 \left\|\bar{O}_{L_1}\right\| \cdot \|O_Z\| \, e^{-\ell_0/(2k)} \tag{552}$$

under the conditions of

$$\ell_0 \ge 8k, \quad x \le \frac{1}{ec'_3}, \quad c'_3 := 16ekc_3\gamma(2k)^D = 2^{6-D} ek\bar{J}\gamma^3(2k)^{2D} \frac{\bar{q}}{\ell_0}, \tag{553}$$

where c_3 has been defined in (543)[*8]*.*

Under the choice of (527) for $\bar{q}$, we have

$$\frac{1}{ec'_3} = \frac{\ell_0}{\bar{q}} \cdot \frac{1}{2^{6-D} e^2 k\bar{J}\gamma^3(2k)^{2D}} = t, \tag{554}$$

which allows us to utilize the inequality (552) for $\forall x \le t$. Hence, we apply Lemma 35 to the inequality (551) as

$$\begin{aligned} \left\|\bar{O}_{L_1}(\bar{H}_\tau, 0 \to t) - \bar{O}_{L_1}(\bar{H}_{L'_2,\tau}, 0 \to t)\right\| &\le 2e^3\zeta_0 \int_0^t \sum_{i,j: \mathcal{B}_{i,j} \in \mathcal{S}_{L'_2}} \left\|\bar{h}_{\mathcal{B}_{i,j},x}\right\| e^{-(R-r_0)/(4k)} dx \\ &\le 8te^3\zeta_0 \bar{J}\bar{q}\gamma R^D e^{-(R-r_0)/(4k)}, \end{aligned} \tag{555}$$

where we use $\ell_0 = (R - r_0)/2$, $\left\|\bar{O}_{L_1}\right\| = \|O_{X_0}\| = \zeta_0$ [see Eq. (549)], and the norm inequality (539) for $\left\|\bar{h}_{\mathcal{B}_{i,j},x}\right\|$ to derive

$$\sum_{i,j: \mathcal{B}_{i,j} \in \mathcal{S}_{L'_2}} \left\|\bar{h}_{\mathcal{B}_{i,j},x}\right\| \le 4\bar{J}\bar{q} \sum_{i,j: \mathcal{B}_{i,j} \in \mathcal{S}_{L'_2}} 1 \le 4\bar{J}\bar{q} \sum_{i \in X_0[2\ell_0]} \sum_{j: d_{i,j}=1} 1 \le 4\bar{J}\bar{q}\gamma^2 R^D, \tag{556}$$

[*8] The condition of $\ell_0 \ge 8k$ is not explicitly assumed in the original statement of Ref. [1, Supplementary Lemma 15], but assumed implicitly (see [1, Supplementary (S.84)]).

where we use $|X_0[2\ell_0]| \le |i_0[r_0+2\ell_0]| = |i_0[R]| \le \gamma R^D$ from $X_0 \in i_0[r_0]$.

Finally, from the definition (527) for $\bar{q}$, we have

$$\bar{J}\bar{q}t \le \frac{\ell_0}{2^{6-D}e^2k\gamma^3(2k)^{2D}} = \frac{\ell_0}{8e^3\gamma^2} \cdot \frac{e}{2^{D+3}k^{2D+1}\gamma} \le \frac{R}{8e^3\gamma^2}, \tag{557}$$

which reduces the inequality (555) to

$$\left\| \bar{O}_{L_1}(\bar{H}_\tau, 0 \to t) - \bar{O}_{L_1}(\bar{H}_{L_2',\tau}, 0 \to t) \right\| \le \zeta_0 R^{D+1} e^{-(R-r_0)/(4k)}. \tag{558}$$

From the decomposition (537) and definition (549), we can write

$$\bar{O}_{L_1}(\bar{H}_{L_2',\tau}, 0 \to t) = \bar{O}_{L_1}(\bar{H}_{L_2'}, t) = O_{X_0}(\bar{\Pi}_{\bar{q}} H_{L_2'} \bar{\Pi}_{\bar{q}}, t), \tag{559}$$

which yields

$$\left\| [O_{X_0}(\bar{\Pi}_{\bar{q}} H \bar{\Pi}_{\bar{q}}, t) - O_{X_0}(\bar{\Pi}_{\bar{q}} H_{X_0[2\ell_0-2k]} \bar{\Pi}_{\bar{q}}, t) \right\| \le \zeta_0 R^{D+1} e^{-(R-r_0)/(4k)}, \tag{560}$$

where we use the explicit expression of $L_2' = X_0[2\ell_0 - 2k]$. This completes the proof of Lemma 33. □

Supplementary Note 8. MAIN SUBTHEOREM ON SMALL TIME EVOLUTION

As mentioned, the obtained bound in Theorem 2 is not optimal. In the following sections, we aim to refine the upper bound to the optimal forms as in Theorems 3 and 4. For this purpose, we first derive an essential analytical ingredient for the refinements.

Here, we consider the following setup (see Fig. 16). Let O_{Z_0} be an arbitrary operator supported on a subset $Z_0 \subseteq i_0[r_1]$. We then consider an approximation of the time-evolved operator $O_{Z_0}(\tau)$ in the region $L_0 = Z_0[\ell_0]$:

$$\left\| \left(O_{Z_0}(\tau) - \tilde{O}_{L_0}\right) |\psi\rangle \right\|, \quad L_0 := Z_0[\ell_0], \quad L_0^* := L_0 \setminus Z_0, \tag{561}$$

where $\tilde{O}_{L_0}$ is an appropriate approximation of the operator $O_{Z_0}(\tau)$ [see the inequality (564) for an example of the choice]. As a spectral constraint, we assume the equation of

$$\Pi_{\mathcal{S}(L_0,1/2,N_0)} \Pi_{L_0^*,\le Q} |\psi\rangle = |\psi\rangle, \tag{562}$$

where $\mathcal{S}(L_0, 1/2, N_0)$ has been defined by Eq. (464). The following subtheorem plays a central role in deriving the optimal Lieb-Robinson bounds:

Subtheorem 2. *Let the boson number Q be larger than $|L_0^*|$, i.e., $Q \ge |L_0^*|$. For an arbitrary operator O_{Z_0} ($Z_0 \subseteq i_0[r_1]$, $\|O_{Z_0}\| = \zeta_{Z_0}$) with the condition (49) with $q = q_{Z_0}$, there exists an appropriate operator $\tilde{O}_{L_0}$ such that*

$$\left\| \left(O_{Z_0}(\tau) - \tilde{O}_{L_0}\right) \Pi_{\mathcal{S}(L_0,1/2,N_0)} \Pi_{L_0^*,\le Q} \right\| \le \zeta_{Z_0} \Theta(Q^2) \left\{ e^{-\Theta(\ell_0)} + e^{\Theta(\ell_0)} \left[\Theta(\tau) + \Theta\left(\frac{\tau Q \log(Q)}{\ell_0^2} \right) \right]^{\Theta(\ell_0)} \right\}, \tag{563}$$

where $\tau \le 1/(4\gamma\bar{J})$ and $\ell_0 \ge \log[\Theta[\log(q_{Z_0}N_0)]]$ [see Eq. (584)]. Note that the Θ notation has been defined in Eq. (9).

Remark. In the inequality (563), the approximate operator $\tilde{O}_{L_0}$ is constructed as in Eq. (580) and Eq. (707). In the construction, we do not utilize information of the Hamiltonian outside the region L_0, and hence for the operator $O_{Z_0}(H_{L_0}, \tau)$, we obtain the same inequality as (563). Therefore, by using a similar analysis to (523), we can obtain

$$\begin{aligned} &\left\| [O_{Z_0}(H,\tau) - O_{Z_0}(H_{L_0},\tau)] \Pi_{\mathcal{S}(L_0,1/2,N_0)} \Pi_{L_0^*,\le Q} \right\| \\ &\le \zeta_{Z_0} \Theta(Q^2) \left\{ e^{-\Theta(\ell_0)} + e^{\Theta(\ell_0)} \left[\Theta(\tau) + \Theta\left(\frac{\tau Q \log(Q)}{\ell_0^2} \right) \right]^{\Theta(\ell_0)} \right\} \end{aligned} \tag{564}$$

by using the Hamiltonian H_{L_0} supported on the subset L_0.

A. Proof outline

For the proof, we cannot use a simple approach that has been used for Theorem 2. That is, if we simply truncate the boson number up to Q at each site, we should construct the effective Hamiltonian as in Eq. (528) with $\bar{q} = Q$.

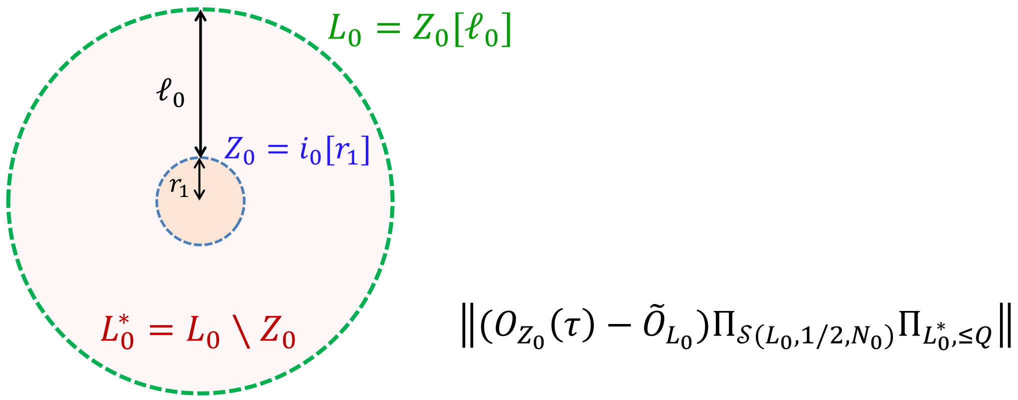

Supplementary Figure 16. Schematic picture of the setup of Subtheorem 2. As the initial condition, we consider the projections $\Pi_{\mathcal{S}(L_0,1/2,N_0)}$ and $\Pi_{L_0^*,\le Q}$, where $L_0 = Z_0[\ell_0]$ and $L_0^* = L_0 \setminus Z_0$. The projection $\Pi_{\mathcal{S}(L_0,1/2,N_0)}$ implies that there are not too many bosons around the region L_0 [see Eq. (464)]. On the projection $\Pi_{L_0^*,\le Q}$, the total number of bosons in the region L_0^* is smaller than Q. Under these two projections, we approximate the small time evolution $O_{Z_0}(H,\tau)$ in the better way as (563). This bound is the crucial ingredient for the optimal Lieb-Robinson bounds in Theorems 3 and 4.

By using similar analyses to Sec. Supplementary Note 7 B, we can obtain the simple upper bound to (533) in the form of

$$\left\|\left(O_{Z_0}(\tau) - U_{L_0}^\dagger O_{Z_0} U_{L_0}\right)\Pi_{\mathcal{S}(L_0,1/2,N_0)}\Pi_{L_0^*,\le Q}\right\| \lesssim \zeta_{Z_0} e^{-\Theta(\ell_0)}, \tag{565}$$

where U_{L_0} is an appropriate unitary operator and the boson number Q satisfies $Q \lesssim \ell_0/(\bar{J}\tau)$ from Eq. (527). In the above bound, the norm exponentially decays with ℓ_0 as long as $\tau \le \Theta(\ell_0/Q)$, which is qualitatively worse than the bound (563) ensuring the decay up to $\tau \le \Theta\left[\ell_0^2/(Q\log(Q))\right]$. To refine the upper bound (565), we need to utilize the fact that all the sites cannot have Q bosons simultaneously. For example, in the 1D case, in the subset from the site i_0 to $L_0^{\rm c}$, the number of bosons on one site is at most $\mathcal{O}(Q/\ell_0)$ *in average*. We need to take this point into account for the proof of Subtheorem 2.

For this purpose, we first define a threshold boson number $\bar{q}$ as follows:

$$\bar{q} = \frac{\mathfrak{q}}{\ell_0} Q, \tag{566}$$

where we choose $\bar{q}$ (or $\mathfrak{q}$) appropriately afterward [see Eq. (589) below]. We then construct a set $\tilde{\mathcal{S}}(L_0^*, \mathbf{N}_X, \bar{q})$ ($X \subseteq L_0^*$) as follows (see Fig. 17):

$$\tilde{\mathcal{S}}(L_0^*, \mathbf{N}_X, \bar{q}) = \{\mathbf{N} | N_i > \bar{q} \quad \text{for} \quad i \in X, \quad N_i \le \bar{q} \quad \text{for} \quad i \in L_0^* \setminus X\}. \tag{567}$$

In this set, the boson numbers are fixed in X and constrained only in $L_0^* \setminus X$. Here, N_i is larger than $\bar{q}$ in the region X but is less than or equal to $\bar{q}$ in the region $L_0^* \setminus X$. Then, we define the projection $\Pi_{\tilde{\mathcal{S}}(L_0^*, \mathbf{N}_X, \bar{q})}$ as

$$\Pi_{\tilde{\mathcal{S}}(L_0^*, \mathbf{N}_X, \bar{q})} := \sum_{\mathbf{N} \in \tilde{\mathcal{S}}(L_0^*, \mathbf{N}_X, \bar{q})} |\mathbf{N}\rangle\langle\mathbf{N}| = \prod_{i \in X} \Pi_{i,N_i} \prod_{i \in L_0^* \setminus X} \Pi_{i,\le \bar{q}}, \tag{568}$$

where $\Pi_{\tilde{\mathcal{S}}(L_0^*, \mathbf{N}_X, \bar{q})}$ is supported on the subset L_0^* and no restrictions are imposed for the boson numbers in $L_0^{*{\rm c}}$. We then obtain

$$\begin{aligned}\sum_{X: X \subseteq L_0^*} \sum_{\mathbf{N}_X : N_i > \bar{q}\ (i \in X)} \Pi_{\tilde{\mathcal{S}}(L_0^*, \mathbf{N}_X, \bar{q})} &= \sum_{X: X \subseteq L_0^*} \prod_{i \in X} \Pi_{i,>\bar{q}} \prod_{i \in L_0^* \setminus X} \Pi_{i,\le \bar{q}} \\ &= \prod_{i \in L_0^*} (\Pi_{i,>\bar{q}} + \Pi_{i,\le \bar{q}}) = \hat{1},\end{aligned} \tag{569}$$

where the summation $\sum_{\mathbf{N}_X : N_i > \bar{q}\ (i \in X)}$ takes all the patterns of $\{N_i\}_{i \in X}$ such that $N_i > \bar{q}$ $(i \in X)$. If the total number of bosons in the region L_0^* is smaller than or equal to Q, we have

$$\bar{q}|X| \le Q \longrightarrow |X| \le \frac{\ell_0}{\mathfrak{q}}. \tag{570}$$

Therefore, we can expand the projection $\Pi_{\mathcal{S}(L_0,1/2,N_0)}\Pi_{L_0^*,\le Q}$ as

$$\Pi_{\mathcal{S}(L_0,1/2,N_0)}\Pi_{L_0^*,\le Q} = \sum_{\substack{X: X \subset L_0^* \\ |X| \le \ell_0/\mathfrak{q}}} \sum_{\substack{\mathbf{N}_X : N_i > \bar{q}\ (i \in X) \\ N_X \le Q}} \Pi_{\tilde{\mathcal{S}}(L_0^*, \mathbf{N}_X, \bar{q})} \Pi_{\mathcal{S}(L_0,1/2,N_0)}\Pi_{L_0^*,\le Q}, \tag{571}$$

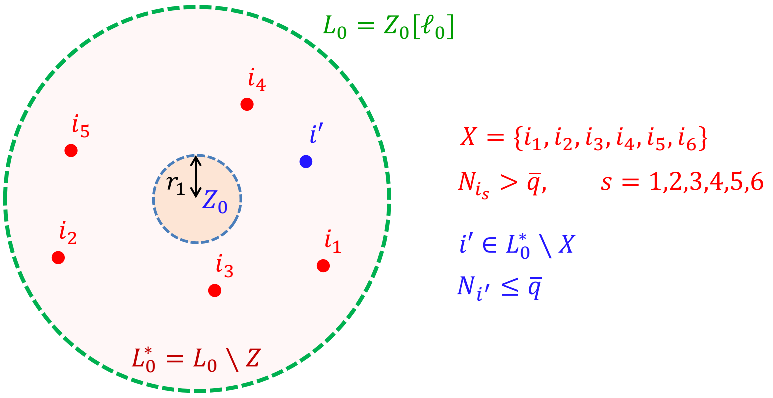

Supplementary Figure 17. Schematic picture of the subset $\tilde{\mathcal{S}}(L_0^*, \mathbf{N}_X, \bar{q})$. In the picture, we consider the case $X = \{i_1, i_2, i_3, i_4, i_5, i_6\}$. Then, if $\mathbf{N} \in \tilde{\mathcal{S}}(L_0^*, \mathbf{N}_X, \bar{q})$, we have $N_{i_s} > \bar{q}$ for $s \in [6]$ and $N_{i'} \le \bar{q}$ for $i' \in L_0^* \setminus X$. Here, the threshold $\bar{q}$ in Eq. (566) is appropriately chosen according to our purpose. By combining $\Pi_{\tilde{\mathcal{S}}(L_0^*, \mathbf{N}_X, \bar{q})}$ with the projection $\Pi_{L_0^*, \le Q}$, we can ensure that the size of X must be smaller than or equal to $Q/\bar{q}$, which leads to the expansion of Eq. (571).

which yields

$$O_{Z_0}(\tau)\Pi_{\mathcal{S}(L_0,1/2,N_0)}\Pi_{L_0^*,\le Q} = \sum_{\substack{X: X\subset L_0^* \\ |X|\le \ell_0/\mathfrak{q}}} \sum_{\substack{\mathbf{N}_X: N_i > \bar{q}\ (i\in X) \\ N_X \le Q}} O_{Z_0}(\tau)\Pi_{\tilde{\mathcal{S}}(L_0^*,\mathbf{N}_X,\bar{q})}\Pi_{\mathcal{S}(L_0,1/2,N_0)}\Pi_{L_0^*,\le Q}. \tag{572}$$

We thus need to consider the following quantity:

$$O_{Z_0}(\tau)\Pi_{\tilde{\mathcal{S}}(L_0^*,\mathbf{N}_X,\bar{q})}\Pi_{\mathcal{S}(L_0,1/2,N_0)}\Pi_{L_0^*,\le Q} \tag{573}$$

for each of the choices of $X \subset L_0^*$ and $\mathbf{N}_X$. In the following, we aim to estimate the approximation error of $O_{Z_0}(\tau)$ under the above projections:

Proposition 36. *Let us take ℓ_0 and $\delta\ell$ such that*

$$\ell_0 \ge 4\delta\ell + 12k + 4, \quad \delta\ell \ge 8\log\left[\mu_0\gamma N_0\left(\lambda_{1/4} + 1 + 2^{4D+3}D!\right) + 4c_{\tau,1}(4q_{Z_0}^3 + 2N_0\lambda_{1/2})\right] - 8\log(\mu_1), \tag{574}$$

$$\mu_0 := 4\left(\lambda_{1/4}c_{\tau,1}^2 + 2c_{\tau,1}\right), \quad \mu_1 := 4\left[2c_{\tau,2} + c_{\tau,1}c_{\tau,2}(1+\lambda_1)\right], \tag{575}$$

where the two conditions for ℓ_0 and $\delta\ell$ are introduced in (660) and (617), respectively. Then, for an arbitrary choices of X and $\bar{q}$ in $\tilde{\mathcal{S}}(L_0^*, \mathbf{N}_X, \bar{q})$, we obtain the Lieb-Robinson bound for an appropriate choices of U_{L_0} [see Eq. (707)]:

$$\left\|\left(O_{Z_0}(\tau) - U_{L_0}^\dagger O_{Z_0} U_{L_0}\right)\Pi_{\tilde{\mathcal{S}}(L_0^*,\mathbf{N}_X,\bar{q})}\Pi_{\mathcal{S}(L_0,1/2,N_0)}\Pi_{L_0^*,\le Q}\right\| \le \delta_1 + \delta_2, \tag{576}$$

where δ_1 and δ_2 are defined as follows:

$$\delta_1 = 8\zeta_{Z_0}|L_0^*|e^{-\tilde{q}} + 56\tau\gamma\zeta_{Z_0}\bar{J}e^{-\tilde{q}}\bar{N}_{\tilde{L}}, \tag{577}$$

$$\delta_2 = \frac{16\gamma\bar{J}\tau\zeta_{Z_0}}{2^D}\bar{N}_{\tilde{L}}\left[\frac{\tilde{c}_1\tau}{\ell_0}\left(\frac{\tilde{c}_2 Q}{\ell_0} + \tilde{c}_{\tilde{q},\bar{q}}\right)\right]^{\ell_0/(4k)}, \tag{578}$$

with

$$\tilde{c}_1 := 2^{D+6}e^2\gamma^3 k^{2D+1}\bar{J}, \quad \tilde{c}_2 := 2k(4 + \mu_0\lambda_{3/4}), \quad \tilde{c}_{\tilde{q},\bar{q}} := 4\mu_1\tilde{q} + 4\bar{q} + \mu_0\lambda_{3/4}\bar{q}. \tag{579}$$

Here, $\tilde{q}$ is a free parameter which can be arbitrarily chosen [see Eq. (636) below] and $\bar{N}_{\tilde{L}}$ is upper-bounded by the inequality (652).

From Proposition 36, by defining

$$\tilde{O}_{L_0} := \sum_{\substack{X: X\subset L_0^* \\ |X|\le \ell_0/\mathfrak{q}}} \sum_{\substack{\mathbf{N}_X: N_i > \bar{q}\ (i\in X) \\ N_X \le Q}} U_{L_0}(L_0^*, \mathbf{N}_X, \bar{q})^\dagger O_{Z_0} U_{L_0}(L_0^*, \mathbf{N}_X, \bar{q})\Pi_{\tilde{\mathcal{S}}(L_0^*,\mathbf{N}_X,\bar{q})} \tag{580}$$

with $U_{L_0}(L_0^*, \mathbf{N}_X, \bar{q})$ an appropriate unitary operator, we obtain

$$\begin{aligned}\left\|\left(O_{Z_0}(\tau) - \tilde{O}_{L_0}\right)\Pi_{\mathcal{S}(L_0,1/2,N_0)}\Pi_{L_0^*,\le Q}\right\| &\le \sum_{\substack{X: X\subset L_0^* \\ |X|\le \ell_0/\mathfrak{q}}} \sum_{\substack{\mathbf{N}_X: N_i > \bar{q}\ (i\in X) \\ N_X \le Q}} (\delta_1 + \delta_2) \le \sum_{\substack{X: X\subset L_0^* \\ |X|\le \ell_0/\mathfrak{q}}} Q^{|X|}(\delta_1+\delta_2) \\ &\le 2(\delta_1+\delta_2)(Q|L_0^*|)^{\ell_0/\mathfrak{q}} \le 2(\delta_1+\delta_2)Q^{2\ell_0/\mathfrak{q}} = 2(\delta_1+\delta_2)\exp\left[\frac{2Q}{\bar{q}}\log(Q)\right],\end{aligned} \tag{581}$$

where in the third inequality, we use

$$\sum_{r=0}^{\ell_0/\mathfrak{q}} \sum_{\substack{X:X\subset L_0^* \\ |X|=r}} 1 = \sum_{r=0}^{\ell_0/\mathfrak{q}} \binom{|L_0^*|}{r} \le 2|L_0^*|^{\ell_0/\mathfrak{q}}, \tag{582}$$

in the fourth inequality, we use $Q \ge |L_0^*|$ from the assumption, and in the last equation, we use $Q = \bar{q}\ell_0/\mathfrak{q}$.

B. Completing the proof of Subtheorem 2

We have obtained all the ingredients to complete the proof. In the following discussions, we fully utilize the Θ notation in Eq. (9) to avoid the complication of the notations. We first choose $\delta\ell$ and ℓ_0 such that the conditions in (574) are satisfied:

$$\begin{aligned}&\delta\ell \ge 8\log\left[\mu_0\gamma N_0\left(\lambda_{1/4}+1+2^{4D+3}D!\right)+4c_{\tau,1}(4q_{Z_0}^3+2N_0\lambda_{1/2})\right]-8\log(\mu_1)\\ &\longrightarrow \delta\ell = \Theta\left[\log(q_{Z_0}N_0)\right],\end{aligned} \tag{583}$$

and

$$\ell_0 \ge 4\delta\ell + 12k + 4 = \Theta\left[\log(q_{Z_0}N_0)\right]. \tag{584}$$

We second choose the free parameter $\tilde{q}$ such that

$$\tilde{c}_{\tilde{q},\bar{q}} = 4\mu_1\tilde{q} + 4\bar{q} + \mu_0\lambda_{3/4}\bar{q} \le 2\mu_0\lambda_{3/4}\tilde{q} \longrightarrow \tilde{q} = \Theta(\bar{q}), \tag{585}$$

where we have defined $\tilde{c}_{\tilde{q},\bar{q}}$ in Eq. (579). Then, the quantity $\bar{N}_{\tilde{L}}$ is upper-bounded from (652) as follows:

$$\bar{N}_{\tilde{L}} \le \Theta(Q+\bar{q}|L_0^*|) \le \Theta(\bar{q}Q), \tag{586}$$

which yields

$$\delta_1 = 8\zeta_{Z_0}|L_0^*|e^{-\tilde{q}} + 56\tau\gamma\zeta_{Z_0}\bar{J}e^{-\tilde{q}}\bar{N}_{\tilde{L}} \le \zeta_{Z_0}\Theta(\bar{q}Q)e^{-\Theta(\bar{q})}, \tag{587}$$

where we use $|L_0^*| \le Q$. Moreover, we have

$$\delta_2 = \frac{16\gamma\bar{J}\tau\zeta_{Z_0}}{2^D}\bar{N}_{\tilde{L}}\left[\frac{\tilde{c}_1\tau}{\ell_0}\left(\frac{\tilde{c}_2 Q}{\ell_0}+\tilde{c}_{\tilde{q},\bar{q}}\right)\right]^{\ell_0/(4k)} = \zeta_{Z_0}\Theta(\bar{q}Q)\left[\Theta\left(\frac{\tau Q}{\ell_0^2}\right)+\Theta\left(\frac{\tau\bar{q}}{\ell_0}\right)\right]^{\Theta(\ell_0)}, \tag{588}$$

where we use the fact that $\tilde{c}_1$ and $\tilde{c}_2$ are $\mathcal{O}(1)$ constants [see Eq. (579)].

In the following, we choose $\bar{q}$ such that

$$\bar{q} = \Theta\left(\frac{Q\log(Q)}{\ell_0}\right) + \Theta(\ell_0), \quad \text{which implies} \quad \exp\left[\frac{2Q}{\bar{q}}\log(Q)\right] \le e^{\Theta(\ell_0)}. \tag{589}$$

By combining the above choice with Eqs (587) and (588), we can upper-bound $2(\delta_1+\delta_2)\exp\left[\frac{2Q}{\bar{q}}\log(Q)\right]$ in the inequality (581) in the form of

$$2(\delta_1+\delta_2)\exp\left[\frac{2Q}{\bar{q}}\log(Q)\right] \le \zeta_{Z_0}\Theta(Q^2)\left\{e^{-\Theta(\ell_0)} + e^{\Theta(\ell_0)}\left[\Theta(\tau)+\Theta\left(\frac{\tau Q\log(Q)}{\ell_0^2}\right)\right]^{\Theta(\ell_0)}\right\}, \tag{590}$$

where we use $\bar{q} \le \Theta(Q) + \Theta(\ell_0) \le \Theta(Q)$ because of $Q \ge |L_0^*| = |Z_0[\ell_0]| - |Z_0| \ge \Theta(\ell_0)$. By applying the above inequality to (581), we obtain the main inequality (563). This completes the proof of Subtheorem 2. □

C. Proof of Proposition 36: spectral constraint for construction of effective Hamiltonian

We here consider an arbitrary quantum state $|\psi_{\mathbf{N}}\rangle$ such that

$$\Pi_{\tilde{\mathcal{S}}(L_0^*,\mathbf{N}_X,\bar{q})}\Pi_{\mathcal{S}(L_0,1/2,N_0)}\Pi_{L_0^*,\le Q}|\psi_{\mathbf{N}}\rangle = |\psi_{\mathbf{N}}\rangle. \tag{591}$$

Here, the quantum state $|\psi_{\mathbf{N}}\rangle$ is characterized by $\mathbf{N}$ as follows:

$$\begin{aligned}&\Pi_{i,\le N_i}|\psi_{\mathbf{N}}\rangle = |\psi_{\mathbf{N}}\rangle, \quad N_i = N_0 e^{d_{i,L_0}/2} \quad \text{for} \quad i \in L_0^{*\mathrm{c}},\\ &\Pi_{i,\le N_i}|\psi_{\mathbf{N}}\rangle = |\psi_{\mathbf{N}}\rangle, \quad N_i = \bar{q} \quad \text{for} \quad i \in L_0^* \setminus X,\\ &\Pi_{i,N_i}|\psi_{\mathbf{N}}\rangle = |\psi_{\mathbf{N}}\rangle, \quad \text{i.e.} \quad \hat{n}_i|\psi_{\mathbf{N}}\rangle = N_i|\psi_{\mathbf{N}}\rangle, \quad N_i > \bar{q} \quad \text{for} \quad i \in X,\end{aligned} \tag{592}$$

with

$$\sum_{i\in X} N_i \le Q, \tag{593}$$

where the condition for $i \in L_0^{*c}$ originates from the projection $\Pi_{\mathcal{S}(L_0,1/2,N_0)}$, the conditions for $i \in L_0^* \setminus X$ and $i \in X$ originate from the projection $\Pi_{\tilde{\mathcal{S}}(L_0^*,\mathbf{N}_X,\bar{q})}$, and the condition $\sum_{i\in X} N_i \le Q$ originates from the projection $\Pi_{L_0^*,\le Q}$. Note that any quantum state in the Hilbert space spanned by $\Pi_{\tilde{\mathcal{S}}(L_0^*,\mathbf{N}_X,\bar{q})}\Pi_{\mathcal{S}(L_0,1/2,N_0)}\Pi_{L_0^*,\le Q}$ is described by the form of $|\psi_{\mathbf{N}}\rangle$. Therefore, for an arbitrary operator O, we obtain

$$\left\| O\Pi_{\tilde{\mathcal{S}}(L_0^*,\mathbf{N}_X,\bar{q})}\Pi_{\mathcal{S}(L_0,1/2,N_0)}\Pi_{L_0^*,\le Q} \right\| = \sup_{\psi} \left\| O\Pi_{\tilde{\mathcal{S}}(L_0^*,\mathbf{N}_X,\bar{q})}\Pi_{\mathcal{S}(L_0,1/2,N_0)}\Pi_{L_0^*,\le Q}|\psi\rangle \right\| \le \sup_{\psi_{\mathbf{N}}} \|O|\psi_{\mathbf{N}}\rangle\| . \tag{594}$$

To prove the main inequality (576), we start from the approximation by using the effective Hamiltonian as

$$\tilde{H}[\tilde{L}',\mathbf{q}] := \bar{\Pi}_{\tilde{L}',\mathbf{q}} H \bar{\Pi}_{\tilde{L}',\mathbf{q}}, \quad \bar{\Pi}_{\tilde{L}',\mathbf{q}} := \prod_{i\in\tilde{L}'} \Pi_{i,\le q_i} \tag{595}$$

with

$$\tilde{L}' := \tilde{L}[-1], \tag{596}$$

where the subset $\tilde{L}$ ($\subset L_0^*$) will be defined in Eq. (615) (see also Fig. 18), and $\mathbf{q} = \{q_i\}_{i\in\tilde{L}'}$ are appropriately chosen as in Eq. (636). In the present section and the subsequent section Supplementary Note 8 D, we aim to upper-bound

$$\left\| e^{iH\tau} O_{Z_0} e^{-iH\tau} |\psi_{\mathbf{N}}\rangle - e^{i\tilde{H}[\tilde{L}',\mathbf{q}]\tau} O_{Z_0} e^{-i\tilde{H}[\tilde{L}',\mathbf{q}]\tau} |\psi_{\mathbf{N}}\rangle \right\| , \tag{597}$$

where the explicit upper bound for the above quantity will be given in Lemma 40.

For the estimation of the norm (597), we utilize Lemma 25, where it is critical to upper-bound the norm of

$$\left\| \Pi_{i,>q_i} e^{iH\tau_2} O_{Z_0} e^{-iH\tau_1} |\psi_{\mathbf{N}}\rangle \right\| \quad \text{for} \quad \forall i \in \tilde{L}. \tag{598}$$

In the present section, the final goal is to prove the statement in Corollary 39. We first prove the following lemma:

Lemma 37. *For arbitrary τ_1 and τ_2 such that $\tau_1, \tau_2 \le \tau \le 1/(4\gamma\bar{J})$, we have*

$$\left\| \hat{n}_i^{s/2} e^{iH\tau_2} O_{Z_0} e^{-iH\tau_1} |\psi_{\mathbf{N}}\rangle \right\|^2 \le \zeta_{Z_0}^2 \left[4N_i + \mu_0 \sum_{j\in\Lambda} e^{-3d_{i,j}/4} N_j + \mu_1 s + 4c_{\tau,1} e^{-d_{i,Z_0}} (4q_{Z_0}^3 + N_{Z_0}^*) \right]^s \tag{599}$$

for $\forall i \in L_0^*$, where we define μ_0, μ_1 and $N_{Z_0}^*$ as

$$\mu_0 := 4\left(\lambda_{1/4} c_{\tau,1}^2 + 2c_{\tau,1}\right), \quad \mu_1 := 4\left[2c_{\tau,2} + c_{\tau,1}c_{\tau,2}(1+\lambda_1)\right], \quad N_{Z_0}^* := N_{Z_0} + c_{\tau,1} \sum_{j\in Z_0} \sum_{j'\in\Lambda} e^{-d_{j,j'}} N_{j'} . \tag{600}$$

1. *Proof of Lemma 37*

The proof is similar to the one for Lemma 28. For the proof, we denote

$$\begin{aligned} \rho_{\tau_1} &= e^{-iH\tau_1} |\psi_{\mathbf{N}}\rangle\langle\psi_{\mathbf{N}}| e^{iH\tau_1}, \\ \rho_{\tau_1,\tau_2} &= e^{iH\tau_2} O_{Z_0} e^{-iH\tau_1} |\psi_{\mathbf{N}}\rangle\langle\psi_{\mathbf{N}}| e^{iH\tau_1} O_{Z_0}^\dagger e^{-iH\tau_2}. \end{aligned} \tag{601}$$

Then, by using Subtheorem 1, we obtain

$$\left\| \hat{n}_i^{s/2} e^{iH\tau_2} O_{Z_0} e^{-iH\tau_1} |\psi_{\mathbf{N}}\rangle \right\|^2 = \mathrm{tr}\left[\hat{n}_i^s \rho_{\tau_1,\tau_2}\right] \le \mathrm{tr}\left[\left(\hat{n}_i + c_{\tau,1}\hat{\mathcal{D}}_i + c_{\tau,2} s \right)^s O_{Z_0} \rho_{\tau_1} O_{Z_0}^\dagger \right], \tag{602}$$

where $\hat{\mathcal{D}}_i$ is defined as $\hat{\mathcal{D}}_i = \sum_{j\in\Lambda} e^{-d_{i,j}} \hat{n}_j$ using Eq. (75). We next apply Lemma 3, where the condition (49) is now given by

$$\Pi_{Z_0,>m+q_{Z_0}} O_{Z_0} \Pi_{Z_0,m}, \quad \Pi_{Z_0,<m-q_{Z_0}} O_{Z_0} \Pi_{Z_0,m} = 0, \tag{603}$$

which is assumed in the main statement of Subtheorem 2. We then apply the inequality (50) to (602) with $q \to q_{Z_0}$, $O_L \to O_{Z_0}$ and $\bar{\nu} \to c_{\tau,1} e^{-d_{i,Z_0}}$:

$$O_{Z_0}^\dagger \left(\hat{n}_i + c_{\tau,1}\hat{\mathcal{D}}_i + c_{\tau,2} s \right)^s O_{Z_0} \preceq 4^s \zeta_{Z_0}^2 \left[\hat{n}_i + c_{\tau,1}\hat{\mathcal{D}}_i + c_{\tau,2} s + c_{\tau,1} e^{-d_{i,Z_0}} \left(4q_{Z_0}^3 + \hat{n}_{Z_0} \right) \right]^s . \tag{604}$$

Note that $i \notin Z_0$ from $i \in \tilde{L}$ and $\tilde{L} \cap Z_0 = 0$ (see Fig. 18). By applying the inequality (604) to (602), we obtain

$$\mathrm{tr}\left[\hat{n}_i^s \rho_{\tau_1,\tau_2}\right] \le \zeta_{Z_0}^2 \mathrm{tr}\left\{4^s \left[\hat{n}_i + c_{\tau,1}\hat{\mathcal{D}}_i + c_{\tau,2}s + c_{\tau,1}e^{-d_{i,Z_0}}\left(4q_{Z_0}^3 + \hat{n}_{Z_0}\right)\right]^s \rho_{\tau_1}\right\}. \tag{605}$$

Then, by using the Hölder inequality as in (327), we have

$$\mathrm{tr}\left[\hat{n}_{i_1}\hat{n}_{i_2}\cdots\hat{n}_{i_s}\rho_{\tau_1}\right] \le \prod_{m=1}^{s}\left\{\mathrm{tr}\left[\hat{n}_{i_m}^s \rho_{\tau_1}\right]\right\}^{1/s}. \tag{606}$$

For each of $\{\mathrm{tr}\left[\hat{n}_{i_m}^s \rho_{\tau_1}\right]\}_{m=1}^s$, by applying Subtheorem 1, we have

$$\begin{aligned}\left\{\mathrm{tr}\left[\hat{n}_{i_m}^s \rho_{\tau_1}\right]\right\}^{1/s} &\le \left\{\mathrm{tr}\left[(\hat{n}_{i_m} + c_{\tau,1}\mathcal{D}_{i_m} + c_{\tau,2}s)^s \left|\psi_{\mathbf{N}}\right\rangle\left\langle\psi_{\mathbf{N}}\right|\right]\right\}^{1/s} \\ &\le N_{i_m} + c_{\tau,1}\mathcal{D}_{i_m}(\mathbf{N}) + c_{\tau,2}s\end{aligned} \tag{607}$$

with $\mathcal{D}_{i_m}(\mathbf{N})$ defined as

$$\mathcal{D}_{i_m}(\mathbf{N}) := \sum_{j\in\Lambda} e^{-d_{i_m,j}} N_j, \tag{608}$$

where we use $\rho_{\tau_1=0} = \left|\psi_{\mathbf{N}}\right\rangle\left\langle\psi_{\mathbf{N}}\right|$ and Eq. (592). We thus reduce the inequality (605) to the form of

$$\mathrm{tr}\left[\hat{n}_i^s \rho_{\tau_1,\tau_2}\right] \le 4^s\zeta_{Z_0}^2\left[N_i + c_{\tau,1}\mathcal{D}_i(\mathbf{N}) + c_{\tau,1}\tilde{\tilde{\mathcal{D}}}_i(\mathbf{N}) + 2c_{\tau,2}s + c_{\tau,1}e^{-d_{i,Z_0}}\left(4q_{Z_0}^3 + N_{Z_0}^* + c_{\tau,2}s\right)\right]^s, \tag{609}$$

where we define $\tilde{\tilde{\mathcal{D}}}_i(\mathbf{N})$ and $N_{Z_0}^*$ as

$$\begin{aligned}\tilde{\tilde{\mathcal{D}}}_i(\mathbf{N}) &= \sum_{j\in\Lambda} e^{-d_{i,j}}\left(N_j + c_{\tau,1}\mathcal{D}_j(\mathbf{N}) + c_{\tau,2}s\right), \\ N_{Z_0}^* &:= N_{Z_0} + c_{\tau,1}\sum_{j\in Z_0}\mathcal{D}_j(\mathbf{N}) = N_{Z_0} + c_{\tau,1}\sum_{j\in Z_0}\sum_{j'\in\Lambda} e^{-d_{j,j'}}N_{j'}.\end{aligned} \tag{610}$$

To estimate the RHS of (609), we consider

$$\begin{aligned}c_{\tau,1}\mathcal{D}_i(\mathbf{N}) + c_{\tau,1}\tilde{\tilde{\mathcal{D}}}_i(\mathbf{N}) =& c_{\tau,1}\sum_{j\in\Lambda} e^{-d_{i,j}}N_j + c_{\tau,1}\sum_{j\in\Lambda} e^{-d_{i,j}}\left(N_j + c_{\tau,2}s + c_{\tau,1}\sum_{j'\in\Lambda} e^{-d_{j,j'}}N_{j'}\right) \\ =& 2c_{\tau,1}\sum_{j\in\Lambda} e^{-d_{i,j}}N_j + c_{\tau,1}c_{\tau,2}s\sum_{j\in\Lambda} e^{-d_{i,j}} + c_{\tau,1}^2\sum_{j\in\Lambda}\sum_{j'\in\Lambda} e^{-d_{i,j}-d_{j,j'}}N_{j'}.\end{aligned} \tag{611}$$

By using the inequality (12) in Lemma 1, we obtain

$$c_{\tau,1}c_{\tau,2}s\sum_{j\in\Lambda} e^{-d_{i,j}} \le c_{\tau,1}c_{\tau,2}\lambda_1 s, \tag{612}$$

and

$$\sum_{j'\in\Lambda} e^{-d_{i,j'}-d_{j',j}} \le \sum_{j'\in\Lambda} e^{-(d_{i,j'}+d_{j',j})/4-3d_{i,j}/4} \le e^{-3d_{i,j}/4}\sum_{j'\in\Lambda} e^{-d_{i,j'}/4} \le e^{-3d_{i,j}/4}\lambda_{1/4}, \tag{613}$$

where we use $d_{i,j'} + d_{j',j} \ge d_{i,j}$.

By combining the inequalities (612) and (613) with (611), we have

$$c_{\tau,1}\mathcal{D}_i(\mathbf{N}) + c_{\tau,1}\tilde{\tilde{\mathcal{D}}}_i(\mathbf{N}) \le \left(\lambda_{1/4}c_{\tau,1}^2 + 2c_{\tau,1}\right)\sum_{j\in\Lambda} e^{-3d_{i,j}/4}N_j + c_{\tau,1}c_{\tau,2}\lambda_1 s. \tag{614}$$

By applying the inequality (614) to (609), we prove the main inequality (619). This completes the proof. □

———————————————————————————[**End of Proof of Lemma 37**]

In Lemma 37, the upper bound (619) is still complicated since it includes the summation with respect to all the sites $j \in \Lambda$. We focus on the case where $i \in \tilde{L}$ and further simplify the form of (619), where we define the subset $\tilde{L}$ as follows (see Fig. 18):

$$\tilde{L} := Z_0[\ell_0 - \delta\ell] \setminus Z_0[\delta\ell]. \tag{615}$$

Note that the region $\tilde{L}$ is separated from L_0^{c} and Z_0 by a distance $\delta\ell$, i.e.,

$$d_{\tilde{L},Z_0} = d_{\tilde{L},L_0^{\mathrm{c}}} = \delta\ell. \tag{616}$$

In the following, we aim to prove the following lemma:

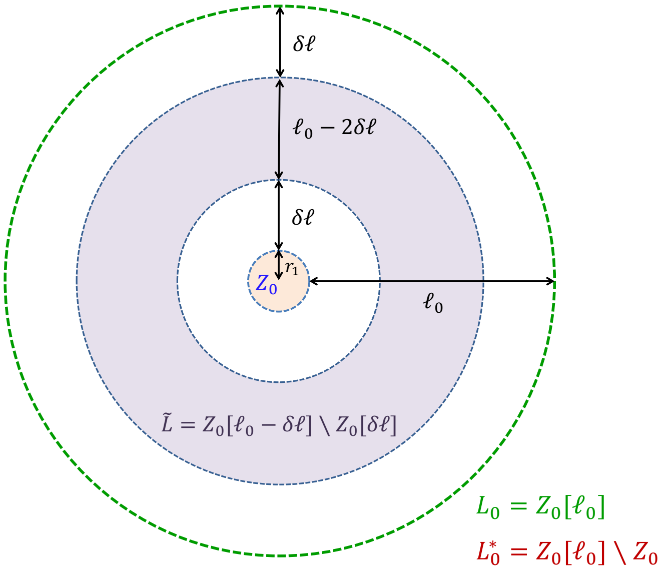

Supplementary Figure 18. Schematic picture of the region $\tilde{L}$. By defining the subset $\tilde{L}$ ($\subset L_0^*$) as in Eq. (615), we have $d_{\tilde{L},Z_0} = d_{\tilde{L},L_0^{\rm c}} = \delta\ell$. As shown in Eq. (595), we are going to perform the truncations of boson number in the region $\tilde{L}[-1]$.

Lemma 38. *If we choose $\delta\ell$ such that*

$$\delta\ell \geq 8\log\left[\mu_0\gamma N_0\left(\lambda_{1/4} + 1 + 2^{4D+3}D!\right) + 4c_{\tau,1}(4q_{Z_0}^3 + 2N_0\lambda_{1/2})\right] - 8\log(\mu_1), \tag{617}$$

we obtain

$$\left\|\hat{n}_i^{s/2} e^{iH\tau_2} O_{Z_0} e^{-iH\tau_1}|\psi_{\mathbf{N}}\rangle\right\|^2 \leq \zeta_{Z_0}^2\left(4N_i + \mu_0\sum_{j\in L_0^*} e^{-3d_{i,j}/4}N_j + 2\mu_1 s\right)^s \quad \textit{for} \quad \forall i \in \tilde{L}. \tag{618}$$

2. *Proof of Lemma 38*

Our purpose is to prove

$$4N_i + \mu_0\sum_{j\in\Lambda} e^{-3d_{i,j}/4}N_j + \mu_1 s + 4c_{\tau,1}e^{-d_{i,Z_0}}(4q_{Z_0}^3 + N_{Z_0}^*) \leq 4N_i + \mu_0\sum_{j\in L_0^*} e^{-3d_{i,j}/4}N_j + 2\mu_1 s \tag{619}$$

for $i \in \tilde{L}$, where the first term appears in (619) of Lemma 37. For the proof, we need to upper-bound the quantities of

$$\sum_{j\in Z_0} e^{-3d_{i,j}/4}N_j + \sum_{j\in L_0^{\rm c}} e^{-3d_{i,j}/4}N_j \quad \text{and} \quad N_{Z_0}^* = N_{Z_0} + \sum_{j\in Z_0}\sum_{j'\in\Lambda} e^{-d_{j,j'}}N_{j'}. \tag{620}$$

Note that $\Lambda \setminus L_0^* = Z_0 \sqcup L_0^{\rm c}$ ($L_0^* = L_0 \setminus Z_0$). We first consider $\sum_{j\in Z_0} e^{-3d_{i,j}/4}N_j + \sum_{j\in L_0^{\rm c}} e^{-3d_{i,j}/4}N_j$. From $\mathbf{N} \in \mathcal{S}(L_0, 1/2, N_0)$, we obtain $N_j \leq e^{d_{j,L_0}/2}N_0$ [see also Eq. (592)], and hence

$$\sum_{j\in Z_0} e^{-3d_{i,j}/4}N_j \leq \sum_{j\in Z_0} e^{-3d_{i,j}/4}e^{d_{j,L_0}/2}N_0 \leq e^{-d_{i,Z_0}/2}N_0\sum_{j\in\Lambda} e^{-d_{i,j}/4} \leq \lambda_{1/4}e^{-\delta\ell/2}N_0, \tag{621}$$

and

$$\sum_{j\in L_0^{\rm c}} e^{-3d_{i,j}/4}N_j \leq \sum_{j\in L_0^{\rm c}} e^{-3d_{i,j}/4}e^{d_{j,L_0}/2}N_0 \leq \sum_{r=\delta\ell}\sum_{j:d_{i,j}=r} e^{-d_{i,j}/4}N_0 \leq \sum_{r=\delta\ell}\gamma r^D e^{-r/4}N_0, \tag{622}$$

where we use $d_{i,Z_0} \geq \delta\ell$ and $d_{i,j} \geq \delta\ell$ for $\forall j \in L_0^{\rm c}$ as long as $i \in \tilde{L}$ (see Fig. 18). By using the inequality (11), we obtain

$$\sum_{r=\delta\ell} r^D e^{-r/4} \leq e^{-\delta\ell/8}\sum_{r=1} r^D e^{-r/8} \leq e^{-\delta\ell/8}e^{-1/8}\left(1 + 2^D D! 8^{D+1}\right) \leq e^{-\delta\ell/8}\left(\lambda_{1/4} + 1 + 2^{4D+3}D!\right). \tag{623}$$

By combining the inequalities (621), (622), and (623), we arrive at the inequality of

$$\sum_{j\in Z_0} e^{-3d_{i,j}/4}N_j + \sum_{j\in L_0^{\rm c}} e^{-3d_{i,j}/4}N_j \le \lambda_{1/4}e^{-\delta\ell/2}N_0 + \gamma N_0 e^{-\delta\ell/8}\left(\lambda_{1/4}+1+2^{4D+3}D!\right)$$
$$\le \gamma N_0 e^{-\delta\ell/8}\left(\lambda_{1/4}+1+2^{4D+3}D!\right) \quad (624)$$

We second consider $N^*_{Z_0}$ in Eq. (620), which is upper-bounded by using $d_{j',L_0} \le d_{j',Z_0} \le d_{j,j'}$ for $\forall j \in Z_0$ and $\forall j' \in L_0^{\rm c}$ as follows:

$$N^*_{Z_0} \le N_0 + N_0 \sum_{j\in Z_0}\left(\sum_{j'\in L_0^{\rm c}} e^{-d_{j,j'}}\left(e^{d_{j',L_0}/2}\right) + \sum_{j'\in L_0} e^{-d_{j,j'}}\right)$$
$$\le N_0 + N_0 \sum_{j\in Z_0}\left(\sum_{j'\in L_0^{\rm c}} e^{-d_{j,j'}/2} + \sum_{j'\in L_0} e^{-d_{j,j'}}\right)$$
$$\le N_0\left(1+\sum_{j\in Z_0}\sum_{j'\in\Lambda} e^{-d_{j,j'}/2}\right) \le 2N_0\lambda_{1/2}, \quad (625)$$

where in the fourth inequality we use the inequality (12) with $\lambda_{1/2} \ge 1$. By applying the inequalities (624) and (625) to the original bound (619), the LHS of (619) is bounded from above by

$$4N_i + \mu_0\sum_{j\in\Lambda} e^{-3d_{i,j}/4}N_j + \mu_1 s + 4c_{\tau,1}e^{-d_{i,Z_0}}\left(4q_{Z_0}^3 + N^*_{Z_0}\right)$$
$$\le 4N_i + \mu_0 \sum_{j\in L_0} e^{-3d_{i,j}/4}N_j + \mu_1 s + e^{-\delta\ell/8}\mu_0\gamma N_0\left(\lambda_{1/4}+1+2^{4D+3}D!\right) + 4c_{\tau,1}e^{-\delta\ell}\left(4q_{Z_0}^3 + 2N_0\lambda_{1/2}\right), \quad (626)$$

where we use $d_{i,Z_0} \ge \delta\ell$ from $i \in \tilde{L}$ [see Eq. (616)].

Finally, the condition (617) yields

$$e^{-\delta\ell/8}\mu_0\gamma N_0\left(\lambda_{1/4}+1+2^{4D+3}D!\right) + 4c_{\tau,1}e^{-\delta\ell}(4q_{Z_0}^3 + 2N_0\lambda_{1/2}) \le \mu_1 \le \mu_1 s. \quad (627)$$

By applying the above inequality to (626), we prove the main inequality (618). This completes the proof. □

———————————————————————————[**End of Proof of Lemma 38**]

By using Lemma 38, we immediately obtain the following corollary:

Corollary 39. *Let us define*

$$\tilde{N}_i := 4N_i + \mu_0 \sum_{j\in L_0^*} e^{-3d_{i,j}/4}N_j. \quad (628)$$

Then, for $Y \subset \tilde{L}$ and $q_i \ge e\tilde{N}_i + 2e\mu_1 p$, we obtain

$$\left\|\hat{n}_Y^{p/2}\Pi_{i,>q_i}e^{iH\tau_2}O_{Z_0}e^{-iH\tau_1}|\psi_{\mathbf{N}}\rangle\right\|^2 \le e\zeta_{Z_0}^2\left[e\tilde{N}_Y + |Y|(q_i - \tilde{N}_i)\right]^p \exp\left(\frac{-q_i + e\tilde{N}_i}{2e\mu_1}\right), \quad (629)$$

where $\tilde{N}_Y = \sum_{i\in Y}\tilde{N}_i$.

3. *Proof of Corollary 39*

From the definition (628) and the inequality (618), we have

$$\mathrm{tr}\left[\hat{n}_i^s\rho_{\tau_1,\tau_2}\right] \le \zeta_{Z_0}^2\left(\tilde{N}_i + 2\mu_1 s\right)^s \quad \text{for} \quad \forall i \in \tilde{L}, \quad (630)$$

where ρ_{τ_1,τ_2} is the density-matrix expression of $e^{iH\tau_2}O_{Z_0}e^{-iH\tau_1}|\psi_{\mathbf{N}}\rangle$ as in Eq. (601). In the following, we assume that s is larger than or equal to p, i.e., $s \ge p$. Then, by using a similar inequality to (606), we obtain

$$\mathrm{tr}\left[\hat{n}_Y^p\hat{n}_i^{s-p}\rho_{\tau_1,\tau_2}\right] \le \zeta_{Z_0}^2\left(\tilde{N}_Y + 2\mu_1|Y|s\right)^p\left(\tilde{N}_i + 2\mu_1 s\right)^{s-p} \quad \text{for} \quad \forall i \in \tilde{L}. \quad (631)$$

The proof of the inequality (629) is the same as that for Lemma 2. By using the Markov inequality, we obtain

$$\left\|\hat{n}_Y^{p/2}\Pi_{i,>q_i}e^{iH\tau_2}O_{Z_0}e^{-iH\tau_1}|\psi_{\mathbf{N}}\rangle\right\|^2 \le \frac{1}{q_i^{s-p}}\left\|\hat{n}_Y^{p/2}\hat{n}_i^{(s-p)/2}e^{iH\tau_2}O_{Z_0}e^{-iH\tau_1}|\psi_{\mathbf{N}}\rangle\right\|^2$$
$$\le \zeta_{Z_0}^2\left(\tilde{N}_Y+2\mu_1|Y|s\right)^p\left(\frac{\tilde{N}_i+2\mu_1 s}{q_i}\right)^{s-p}, \tag{632}$$

where the second inequality is derived from (631). Then, by choosing s as

$$s=\left\lfloor\frac{q_i-e\tilde{N}_i}{2e\mu_1}\right\rfloor, \tag{633}$$

we have $\tilde{N}_i+2\mu_1 s\le q_i/e$ and $\tilde{N}_Y+2\mu_1|Y|s\le \tilde{N}_Y+|Y|(q_i-\tilde{N}_i)/e$. Because of the initial condition for q_i, i.e., $q_i\ge e\tilde{N}_i+2e\mu_1 p$, we can ensure that the condition $s\ge p$ is satisfied for the above choice. Therefore, we prove the main inequality (629). □

[End of Proof of Corollary 39]

D. Proof of Proposition 36: Error estimation for the effective Hamiltonian

By using Corollary 39, we aim to upper-bound the approximation error (597). To estimate it, we utilize Lemma 25 with

$$|\psi\rangle\to|\psi_{\mathbf{N}}\rangle,\quad O\to O_{Z_0},\quad \text{and}\quad \bar{\Pi}\to\bar{\Pi}_{\tilde{L}',\mathbf{q}}=\prod_{i\in\tilde{L}'}\Pi_{i,\le q_i},$$

where we need to consider the quantities of

$$\left\|\bar{\Pi}^{\mathrm{c}}_{\tilde{L}',\mathbf{q}}e^{iH\tau_2}O_{Z_0}e^{-iH\tau_1}|\psi_{\mathbf{N}}\rangle\right\| \tag{634}$$

and

$$\left\|\bar{\Pi}_{\tilde{L}',\mathbf{q}}H_0\bar{\Pi}^{\mathrm{c}}_{\tilde{L}',\mathbf{q}}e^{iH\tau_2}O_{Z_0}e^{-iH\tau_1}|\psi_{\mathbf{N}}\rangle\right\| \tag{635}$$

for appropriate choices of τ_1, τ_2 and O_{Z_0}.

In the above setup, we need to estimate each term that appears in the inequality (408). We prove the following lemma:

Lemma 40. *Let $\tilde{q}$ be an arbitrary integer that will be appropriately chosen according to our purpose [see Eq. (585)]. We here choose $\{q_i\}_{i\in\tilde{L}}$ as*

$$q_i=e\tilde{N}_i+4e\mu_1\tilde{q}\quad \textit{for}\quad i\in\tilde{L}, \tag{636}$$

where $\tilde{N}_i$ has been defined in Eq. (628)[*9]*. Then, under the choices of $|\psi\rangle\to|\psi_{\mathbf{N}}\rangle$, $O\to O_{Z_0}$ and $\bar{\Pi}\to\bar{\Pi}_{\tilde{L}',\mathbf{q}}$, the error in the inequality (408) reduces to the following form:*

$$\left\|e^{iH\tau}O_{Z_0}e^{-iH\tau}|\psi_{\mathbf{N}}\rangle-e^{i\tilde{H}[\tilde{L}',\mathbf{q}]\tau}O_{Z_0}e^{-i\tilde{H}[\tilde{L}',\mathbf{q}]\tau}|\psi_{\mathbf{N}}\rangle\right\|\le 8\zeta_{Z_0}|L_0^*|e^{-\tilde{q}}+48\tau\gamma\zeta_{Z_0}\bar{J}e^{-\tilde{q}}\bar{N}_{\tilde{L}}=\delta_1, \tag{637}$$

where we use the definition (577) for δ_1, and we define

$$\bar{N}_{\tilde{L}}:=\sum_{i\in\tilde{L}}q_i. \tag{638}$$

Remark. In the definition (636), the region where $\{q_i\}_{i\in\tilde{L}}$ have been defined is $\tilde{L}$ instead of $\tilde{L}'$. The definition of q_i for $i\in\tilde{L}\setminus\tilde{L}'=\partial\tilde{L}$ is not necessary for the construction of the effective Hamiltonian, but only used for a technical reason in deriving the upper bound (646).

[*9] Strictly speaking, to ensure the condition in Corollary 39, i.e., $q_i\ge e\tilde{N}_i+2e\mu_1 p$, we have to add an additional condition $\tilde{q}\ge p/2$. Actually, we only utilize the cases of $p=0$ or $p=2$ in the proof, and hence $\tilde{q}\ge p/2=1$ is trivially satisfied.

1. Proof of Lemma 40

The proof is similar to the one for Proposition 29. First of all, from the definition $\bar{\Pi}_{\tilde{L}',\mathbf{q}} = \prod_{i\in\tilde{L}'}\Pi_{i,\le q_i}$, we can immediately derive

$$\left\|\bar{\Pi}^{\rm c}_{\tilde{L}',\mathbf{q}} e^{iH\tau_2} O_{Z_0} e^{-iH\tau_1}|\psi_{\mathbf{N}}\rangle\right\| \le \sum_{i\in\tilde{L}'}\left\|\Pi_{i,>q_i} e^{iH\tau_2} O_{Z_0} e^{-iH\tau_1}|\psi_{\mathbf{N}}\rangle\right\| \le \sqrt{e}\zeta_{Z_0}\sum_{i\in\tilde{L}'}\exp\left(\frac{-q_i+e\tilde{N}_i}{4e\mu_1}\right), \tag{639}$$

where we apply Corollary 39 with $p=0$. Here, we have chosen q_i as

$$q_i = e\tilde{N}_i + 4e\mu_1\tilde{q} \quad \text{for} \quad i\in\tilde{L}, \tag{640}$$

and hence we obtain

$$\left\|\bar{\Pi}^{\rm c}_{\tilde{L}',\mathbf{q}} e^{iH\tau_2} O_{Z_0} e^{-iH\tau_1}|\psi_{\mathbf{N}}\rangle\right\| \le 2\zeta_{Z_0}|\tilde{L}'|e^{-\tilde{q}}. \tag{641}$$

The second task is to upper-bound

$$\left\|\bar{\Pi}_{\tilde{L}',\mathbf{q}} H_0 \bar{\Pi}^{\rm c}_{\tilde{L}',\mathbf{q}} e^{iH\tau_2} O_{Z_0} e^{-iH\tau_1}|\psi_{\mathbf{N}}\rangle\right\|. \tag{642}$$

As in Eq. (458), we first obtain

$$\bar{\Pi}_{\tilde{L}',\mathbf{q}} H_0 \bar{\Pi}^{\rm c}_{\tilde{L}',\mathbf{q}} = \bar{\Pi}_{\tilde{L}',\mathbf{q}}\left(H_{0,\tilde{L}'} + \partial h_{\tilde{L}'}\right)\bar{\Pi}^{\rm c}_{\tilde{L}',\mathbf{q}}. \tag{643}$$

We then obtain the same inequality as (461):

$$\left\|\bar{\Pi}_{\tilde{L}',\mathbf{q}} H_0 \bar{\Pi}^{\rm c}_{\tilde{L}',\mathbf{q}} e^{iH\tau_2} O_{Z_0} e^{-iH\tau_1}|\psi_{\mathbf{N}}\rangle\right\| \le 2\bar{J}\sum_{i\in\tilde{L}'}\sum_{j:d_{i,j}=1}\left\|(\hat{n}_i+\hat{n}_j)\bar{\Pi}^{\rm c}_{i,j,q_i,q_j} e^{iH\tau_2} O_{Z_0} e^{-iH\tau_1}|\psi_{\mathbf{N}}\rangle\right\|, \tag{644}$$

where we define $\bar{\Pi}^{\rm c}_{i,j,q_i,q_j}$ in the similar way to Eq. (460), i.e.,

$$\bar{\Pi}_{i,j,q_i,q_j} = \begin{cases}\Pi_{i,\le q_i}\Pi_{j,\le q_j} & \text{for} \quad i,j\in\tilde{L}', \\ \Pi_{i,\le q_i} & \text{for} \quad i\in\tilde{L}', \quad j\in\tilde{L}'^{\rm c}.\end{cases} \tag{645}$$

Then, as in the inequality (462), we obtain

$$\begin{aligned}
&\left\|(\hat{n}_i+\hat{n}_j)\bar{\Pi}^{\rm c}_{i,j,q_i,q_j} e^{iH\tau_2} O_{Z_0} e^{-iH\tau_1}|\psi_{\mathbf{N}}\rangle\right\| \le \left\|\hat{n}_{\{i,j\}}\left(\Pi_{i,>q_i}+\Pi_{j,>q_j}\right) e^{iH\tau_2} O_{Z_0} e^{-iH\tau_1}|\psi_{\mathbf{N}}\rangle\right\| \\
&\le \left\|\hat{n}_{\{i,j\}}\Pi_{i,>q_i} e^{iH\tau_2} O_{Z_0} e^{-iH\tau_1}|\psi_{\mathbf{N}}\rangle\right\| + \left\|\hat{n}_{\{i,j\}}\Pi_{j,>q_j} e^{iH\tau_2} O_{Z_0} e^{-iH\tau_1}|\psi_{\mathbf{N}}\rangle\right\| \\
&\le \sqrt{e}\zeta_{Z_0}\left[e\tilde{N}_i+e\tilde{N}_j+2(q_i-\tilde{N}_i)\right]\exp\left(\frac{-q_i+e\tilde{N}_i}{4e\mu_1}\right) + \sqrt{e}\zeta_{Z_0}\left[e\tilde{N}_i+e\tilde{N}_j+2(q_j-\tilde{N}_j)\right]\exp\left(\frac{-q_j+e\tilde{N}_j}{4e\mu_1}\right) \\
&\le \sqrt{e}\zeta_{Z_0}\left[e\tilde{N}_i+e\tilde{N}_j+2(q_i-\tilde{N}_i)\right]e^{-\tilde{q}} + \sqrt{e}\zeta_{Z_0}\left[e\tilde{N}_i+e\tilde{N}_j+2(q_j-\tilde{N}_j)\right]e^{-\tilde{q}} \\
&\le 2\sqrt{e}\zeta_{Z_0}\left[q_i+q_j+(1-1/e)(e\tilde{N}_i+e\tilde{N}_j)\right]e^{-\tilde{q}} \le 2\sqrt{e}(2-1/e)\cdot\zeta_{Z_0}(q_i+q_j)e^{-\tilde{q}} \le 6\zeta_{Z_0}(q_i+q_j)e^{-\tilde{q}},
\end{aligned} \tag{646}$$

where we use Corollary 39 with $p=2$, $Y=\{i,j\}$ and the definition of $q_i = e\tilde{N}_i + 4e\mu_1\tilde{q}$ for $i\in\tilde{L}$. Note that for $i\in\tilde{L}'=\tilde{L}[-1]$ we have $j\in\tilde{L}$ as long as $d_{i,j}=1$.

By combining the inequalities (644) and (646), we obtain

$$\left\|\bar{\Pi}_{\tilde{L}',\mathbf{q}} H_0 \bar{\Pi}^{\rm c}_{\tilde{L}',\mathbf{q}} e^{iH\tau_2} O_{Z_0} e^{-iH\tau_1}|\psi_{\mathbf{N}}\rangle\right\| \le 12\zeta_{Z_0}\bar{J}e^{-\tilde{q}}\sum_{i\in\tilde{L}'}\sum_{j:d_{i,j}=1}(q_i+q_j) \le 24\gamma\zeta_{Z_0}\bar{J}e^{-\tilde{q}}\bar{N}_{\tilde{L}}, \tag{647}$$

where for the last inequality we use

$$\begin{aligned}
&\sum_{i\in\tilde{L}'}\sum_{j:d_{i,j}=1} q_i \le \sum_{i\in\tilde{L}'}\gamma q_i \le \gamma\sum_{i\in\tilde{L}} q_i = \gamma\bar{N}_{\tilde{L}}, \\
&\sum_{i\in\tilde{L}'}\sum_{j:d_{i,j}=1} q_j \le \sum_{j\in\tilde{L}'[1]}\sum_{i:d_{i,j}=1} q_j \le \gamma\sum_{j\in\tilde{L}} q_j = \gamma\bar{N}_{\tilde{L}}.
\end{aligned} \tag{648}$$

Note that $\tilde{L}'[1]=\tilde{L}$ from the definition of $\tilde{L}'=\tilde{L}[-1]$.

Thus, we have obtained the upper bounds for the norms of (634) and (635) in the forms of (641) and (647), respectively. By applying these bounds to the inequality (408), we have the error of

$$\begin{aligned}
&\left\| e^{iH\tau} O_{Z_0} e^{-iH\tau} |\psi_{\mathbf{N}}\rangle - e^{i\tilde{H}[\tilde{L}',\mathbf{q}]\tau} O_{Z_0} e^{-i\tilde{H}[\tilde{L}',\mathbf{q}]\tau} |\psi_{\mathbf{N}}\rangle \right\| \\
&\le \left\| \bar{\Pi}^{\rm c}_{\tilde{L}',\mathbf{q}} e^{iH\tau} O_{Z_0} e^{-iH\tau} |\psi_{\mathbf{N}}\rangle \right\| + \left\| \bar{\Pi}^{\rm c}_{\tilde{L}',\mathbf{q}} O_{Z_0} e^{-iH\tau} |\psi_{\mathbf{N}}\rangle \right\| + \zeta_{Z_0} \left\| \bar{\Pi}^{\rm c}_{\tilde{L}',\mathbf{q}} |\psi_{\mathbf{N}}\rangle \right\| + \zeta_{Z_0} \left\| \bar{\Pi}^{\rm c}_{\tilde{L}',\mathbf{q}} e^{-iH\tau} |\psi_{\mathbf{N}}\rangle \right\| \\
&\quad + \int_0^\tau \left(\left\| \bar{\Pi}_{\tilde{L}',\mathbf{q}} H_0 \bar{\Pi}^{\rm c}_{\tilde{L}',\mathbf{q}} e^{iH(\tau-\tau_1)} O_{Z_0} e^{-iH\tau} |\psi_{\mathbf{N}}\rangle \right\| + \zeta_{Z_0} \left\| \bar{\Pi}_{\tilde{L}',\mathbf{q}} H_0 \bar{\Pi}^{\rm c}_{\tilde{L}',\mathbf{q}} e^{-iH\tau_1} |\psi_{\mathbf{N}}\rangle \right\| \right) d\tau_1 \\
&\le 8\zeta_{Z_0} |\tilde{L}'| e^{-\tilde{q}} + 48\tau\gamma\zeta_{Z_0} \bar{J} e^{-\tilde{q}} \bar{N}_{\tilde{L}}.
\end{aligned} \tag{649}$$

We thus prove the main inequality (637) by using $|\tilde{L}'| \le |L_0^*|$. This completes the proof. □

———————————————————————————[**End of Proof of Lemma 40**]

We here consider the number of bosons in a region $Y \subseteq \tilde{L}$ under the projection $\bar{\Pi}_{\tilde{L}',\mathbf{q}}$. We are now interested in the upper bound of

$$\sum_{i\in Y} q_i \quad \text{for} \quad Y \subseteq \tilde{L}. \tag{650}$$

Note that by choosing $Y = \tilde{L}$ the above quantity reduces to $\bar{N}_{\tilde{L}}$ in Eq. (638). Originally, we imposed the condition (591) for the boson numbers in the region L_0, where the boson number is smaller than or equal to $\bar{q}$ in $\tilde{L} \setminus X^{\rm c}$, but larger than $\bar{q}$ in X. Here we can prove the following lemma:

Lemma 41. *Under the choice of (636), we obtain the following upper bound:*

$$\sum_{i\in Y} q_i \le e(4+\mu_0\lambda_{3/4})Q + e\left(4\mu_1\tilde{q} + 4\bar{q} + \mu_0\lambda_{3/4}\bar{q}\right)|Y|. \tag{651}$$

In particular, by choosing $Y = \tilde{L}$, we obtain

$$\bar{N}_{\tilde{L}} := \sum_{i\in\tilde{L}} q_i \le e(4+\mu_0\lambda_{3/4})Q + e\left(4\mu_1\tilde{q} + 4\bar{q} + \mu_0\lambda_{3/4}\bar{q}\right)|\tilde{L}|. \tag{652}$$

2. Proof of Lemma 41

By using the definitions of $q_i = e\tilde{N}_i + 4e\mu_1\tilde{q}$ and $\tilde{N}_i := 4N_i + \mu_0 \sum_{j\in L_0^*} e^{-3d_{i,j}/4} N_j$, we first consider

$$\begin{aligned}
\sum_{i\in Y} q_i &= \left(\sum_{i\in(Y\cap X)} + \sum_{i\in(Y\setminus X)} \right) \left(e\tilde{N}_i + 4e\mu_1\tilde{q} \right) \\
&= 4e\mu_1\tilde{q}|Y| + e\left(\sum_{i\in(Y\cap X)} + \sum_{i\in(Y\setminus X)} \right)\left(4N_i + \mu_0 \sum_{j\in L_0^*} e^{-3d_{i,j}/4} N_j \right).
\end{aligned} \tag{653}$$

First, we have

$$\sum_{i\in(Y\cap X)} N_i + \sum_{i\in(Y\setminus X)} N_i \le Q + \bar{q}|Y|, \tag{654}$$

where we use $\sum_{i\in X} N_i \le Q$ and $N_i \le \bar{q}$ for $i \in (Y\setminus X)$ from Eqs. (592) and (593), respectively. Note that $Y \subseteq \tilde{L} \subset L_0^*$. Second, we aim to estimate

$$\begin{aligned}
\left(\sum_{i\in(Y\cap X)} + \sum_{i\in(Y\setminus X)} \right) \sum_{j\in L_0^*} e^{-3d_{i,j}/4} N_j &= \sum_{i\in Y}\sum_{j\in L_0^*} e^{-3d_{i,j}/4} N_j \\
&\le \sum_{i\in Y}\sum_{j\in X} e^{-3d_{i,j}/4} N_j + \sum_{i\in Y}\sum_{j\in L_0^*\setminus X} e^{-3d_{i,j}/4}\bar{q}.
\end{aligned} \tag{655}$$

By using

$$\max_{i\in\Lambda}\left(\sum_{j\in\Lambda} e^{-3d_{i,j}/4} \right) \le \lambda_{3/4} \tag{656}$$

from the inequality (12), we reduce the above inequality to

$$\sum_{i\in Y}\sum_{j\in X} e^{-3d_{i,j}/4}N_j + \sum_{i\in Y}\sum_{j\in L_0^*\setminus X} e^{-3d_{i,j}/4}\bar{q} \le \lambda_{3/4}\sum_{j\in X} N_j + \lambda_{3/4}\sum_{i\in Y}\bar{q} \le \lambda_{3/4}(Q+\bar{q}|Y|). \tag{657}$$

Hence, we obtain

$$\left(\sum_{i\in(Y\cap X)} + \sum_{i\in(Y\setminus X)}\right)\sum_{j\in L_0^*} e^{-3d_{i,j}/4}N_j \le \lambda_{3/4}\left(Q+\bar{q}|Y|\right). \tag{658}$$

By combining the inequalities (654) and (658), we have

$$\begin{aligned}\sum_{i\in Y} q_i &= 4e\mu_1\tilde{q}|Y| + e\left(\sum_{i\in(Y\cap X)} + \sum_{i\in(Y\setminus X)}\right)\left(4N_i + \mu_0\sum_{j\in L_0^*} e^{-3d_{i,j}/4}N_j\right)\\ &\le 4e\mu_1\tilde{q}|Y| + e(4+\mu_0\lambda_{3/4})\left(Q+\bar{q}|Y|\right),\end{aligned} \tag{659}$$

which yields the desired inequality (651). This completes the proof. □

[End of Proof of Lemma 41]

E. Proof of Proposition 36: Lieb-Robinson bound for effective Hamiltonian

In this section, we adopt an additional condition for ℓ_0 as follows:

$$\ell_0 - (2\delta\ell + 2 + 6k) \ge \frac{\ell_0}{2} \longrightarrow \ell_0 \ge 4\delta\ell + 12k + 4, \tag{660}$$

where k has characterized the maximum interaction length in V [see Eq. (26)]. This condition will be used in (702) for the proof of Corollary 43.

In this section, we partially follow the discussion in Sec. Supplementary Note 7 B. We here calculate the norm of

$$\left\| O_{Z_0}\left(\tilde{H}[\tilde{L}',\mathbf{q}],\tau\right) - U_{L_0}^\dagger O_{Z_0} U_{L_0}\right\|, \quad \tilde{H}[\tilde{L}',\mathbf{q}] = \bar{\Pi}_{\tilde{L}',\mathbf{q}} H \bar{\Pi}_{\tilde{L}',\mathbf{q}}, \tag{661}$$

where U_{L_0} is an appropriate unitary operator [see Eq. (707)]. As in Eq. (535), we consider the decomposition of

$$e^{-i\tilde{H}[\tilde{L}',\mathbf{q}]\tau} = e^{-i\tilde{V}[\tilde{L}',\mathbf{q}]\tau}\,\mathcal{T}\exp\left(-i\int_0^\tau e^{i\tilde{V}[\tilde{L}',\mathbf{q}]x}\tilde{H}_0[\tilde{L}',\mathbf{q}]e^{-i\tilde{V}[\tilde{L}',\mathbf{q}]x}dx\right), \tag{662}$$

where $\tilde{V}[\tilde{L}',\mathbf{q}] = \bar{\Pi}_{\tilde{L}',\mathbf{q}} V \bar{\Pi}_{\tilde{L}',\mathbf{q}}$.

We here denote the time evolution of $e^{i\tilde{V}[\tilde{L}',\mathbf{q}]x}\tilde{H}_0[\tilde{L}',\mathbf{q}]e^{-i\tilde{V}[\tilde{L}',\mathbf{q}]x}$ by

$$\begin{aligned}\tilde{H}_x := e^{i\tilde{V}[\tilde{L}',\mathbf{q}]x}\tilde{H}_0[\tilde{L}',\mathbf{q}]e^{-i\tilde{V}[\tilde{L}',\mathbf{q}]x} &= \bar{\Pi}_{\tilde{L}',\mathbf{q}}\sum_{\langle i,j\rangle} J_{i,j}e^{i\tilde{V}[\tilde{L}',\mathbf{q}]x}(b_i^\dagger b_j + \text{h.c.})e^{-i\tilde{V}[\tilde{L}',\mathbf{q}]x}\bar{\Pi}_{\tilde{L}',\mathbf{q}}\\ &=: \sum_{\langle i,j\rangle}\tilde{h}_{\mathcal{B}_{i,j},x}, \quad \mathcal{B}_{i,j} = i[k-1]\cup j[k-1], \quad \operatorname{diam}(\mathcal{B}_{i,j}) \le 2k.\end{aligned} \tag{663}$$

By using the notation of $\tilde{H}_x$, we rewrite the decomposition (662) as

$$e^{-i\tilde{H}[\tilde{L}',\mathbf{q}]\tau} = e^{-i\tilde{V}[\tilde{L}',\mathbf{q}]\tau}\,\mathcal{T}e^{-\int_0^\tau \tilde{H}_x dx}. \tag{664}$$

We note that $\tilde{V}[\tilde{L}',\mathbf{q}]$ is a commuting Hamiltonian, i.e.,

$$\tilde{V}[\tilde{L}',\mathbf{q}] = \sum_{Z:\operatorname{diam}(Z)\le k}\tilde{v}_Z[\tilde{L}',\mathbf{q}], \quad \left[\tilde{v}_Z[\tilde{L}',\mathbf{q}],\tilde{v}_{Z'}[\tilde{L}',\mathbf{q}]\right] = 0. \tag{665}$$

Also, we have obtain a similar inequality to (539)

$$\left\|\tilde{h}_{\mathcal{B}_{i,j},x}\right\| \le |J_{i,j}|\cdot\left\|\bar{\Pi}_{\tilde{L}',\mathbf{q}}(b_i^\dagger b_j + \text{h.c.})\bar{\Pi}_{\tilde{L}',\mathbf{q}}\right\| \le 2\bar{J}(q_i+q_j) \quad (i,j\in\tilde{L}'). \tag{666}$$

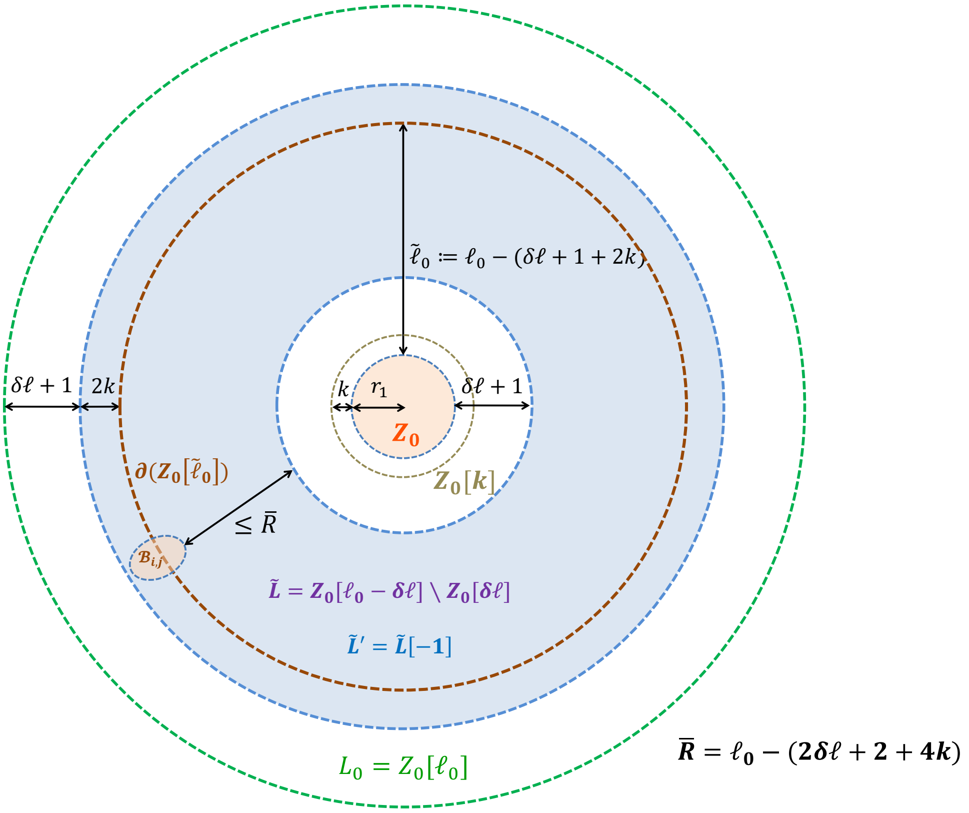

Supplementary Figure 19. Schematic pictures of the regions L_0, $\tilde{L}'$ and $Z_0[\tilde{\ell}_0]$. The time evolution $O_{Z_0}\left(\tilde{H}[\tilde{L}', \mathbf{q}], \tau\right)$ is decomposed as in Eq. (667), where the contribution from $\tilde{V}[\tilde{L}', \mathbf{q}]$ is included in $\tilde{O}_{Z_0[k]}$. To approximate the time evolution of $\tilde{O}_{Z_0[k]}(\tilde{H}_x, 0 \to \tau)$, we use Lemma 34 with the choice of the approximating region as $Z_0[\tilde{\ell}_0]$ (inside the region by brown dots). Then, from the inequality (671), we have to estimate the time evolution of the boundary-interacting terms $\tilde{h}_{\mathcal{B}_{i,j},x}(\tilde{H}_{Z_0[\tilde{\ell}_0],\tau_1}, x \to 0)$. Here, from $\mathrm{diam}(\mathcal{B}_{i,j}) \le 2k$, we can ensure $\mathcal{B}_{i,j} \subset \tilde{L}'$ for $\mathcal{B}_{i,j} \in \mathcal{S}_{Z_0[\tilde{\ell}_0]}$. The length $\bar{R}$ is utilized in Lemma 42, which is now given by the minimum distance between $\mathcal{B}_{i,j}$ and $Z_0[\delta\ell + 1]$; that is, it is bounded from above by $\tilde{\ell}_0 - 2k - (\delta\ell + 1) = \ell_0 - (2\delta\ell + 4k + 2)$ [see Eq. (699)].

We next consider the same equation as Eq. (548):

$$O_{Z_0}\left(\tilde{H}[\tilde{L}', \mathbf{q}], \tau\right) = \tilde{O}_{Z_0[k]}(\tilde{H}_x, 0 \to \tau), \tag{667}$$

where we use the notation (33) and $\tilde{O}_{Z_0[k]}$ ($\|\tilde{O}_{Z_0[k]}\| = \|O_{Z_0}\| = \zeta_{Z_0}$) is defined as

$$\tilde{O}_{Z_0[k]} \coloneqq O_{Z_0}\left(\tilde{V}[\tilde{L}', \mathbf{q}], \tau\right) = e^{i\tilde{V}_{Z_0[k]}[\tilde{L}', \mathbf{q}]\tau} O_{Z_0} e^{-i\tilde{V}_{Z_0[k]}[\tilde{L}', \mathbf{q}]\tau}. \tag{668}$$

Here, the operator $\tilde{V}_{Z_0[k]}[\tilde{L}', \mathbf{q}]$ picks up all the interaction terms $\tilde{v}_{\tilde{Z}}[\tilde{L}', \mathbf{q}]$ in Eq. (665) such that $\tilde{Z} \subset Z_0[k]$ [see also Eqs. (26) and (27)].

In the following, we approximate $\tilde{O}_{Z_0[k]}(\tilde{H}_x, 0 \to \tau)$ by using the subset Hamiltonian $\tilde{H}_{L,x}$:

$$\tilde{O}_{Z_0[k]}(\tilde{H}_x, 0 \to \tau) \approx \tilde{O}_{Z_0[k]}(\tilde{H}_{L_0,x}, 0 \to \tau), \quad \tilde{H}_{L,x} = \sum_{\langle i,j\rangle : \mathcal{B}_{i,j} \subset L} \tilde{h}_{\mathcal{B}_{i,j},x}, \tag{669}$$

where we choose the subset L appropriately afterward. To estimate the approximation error for (669), we utilize Lemma 34, in which we let $O_X \to \tilde{O}_{Z_0[k]}$ as in Eq. (668) and choose the subset L as $Z_0[\tilde{\ell}_0]$ (see also Fig. 19) with

$$\tilde{\ell}_0 = \ell_0 - (\delta\ell + 2k + 1). \tag{670}$$

From the inequality (546) in Lemma 34, we obtain the error for the approximation (669) in the following form:

$$\left\| \tilde{O}_{Z_0[k]}(\tilde{H}_x, 0 \to \tau) - \tilde{O}_{Z_0[k]}(\tilde{H}_{Z_0[\tilde{\ell}_0],x}, 0 \to \tau) \right\| \le \sum_{\langle i,j\rangle : \mathcal{B}_{i,j} \in \mathcal{S}_{Z_0[\tilde{\ell}_0]}} \int_0^\tau \left\| [\tilde{h}_{\mathcal{B}_{i,j},x}(\tilde{H}_{Z_0[\tilde{\ell}_0],\tau_1}, x \to 0), \tilde{O}_{Z_0[k]}] \right\| dx, \tag{671}$$

where $\mathcal{S}_{Z_0[\tilde{\ell}_0]}$ has been defined in Eq. (547). We note that as long as $\mathcal{B}_{i,j} \in \mathcal{S}_{Z_0[\tilde{\ell}_0]}$ we can ensure $\mathcal{B}_{i,j} \in \tilde{L}'$ because of $\mathrm{diam}(\mathcal{B}_{i,j}) \le 2k$. Then, we have to upper-bound the norm of the commutator like

$$\left\| [\tilde{h}_{\mathcal{B}_{i,j},x}(\tilde{H}_{Z_0[\tilde{\ell}_0],\tau_1}, x \to 0), \tilde{O}_{Z_0[k]}] \right\| \quad \left(\mathcal{B}_{i,j} \in \mathcal{S}_{Z_0[\tilde{\ell}_0]}\right). \tag{672}$$

The difficulty here is that the norms of the local interaction terms $\{\tilde{h}_{\mathcal{B}_{i,j},\tau}\}_{i,j}$ are not uniformly bounded; that is, the upper bound (666) implies that the norm is small on average but may be large for a particular choice of i,j.

Before showing the Lieb-Robinson bound for (672), we prove the following convenient lemma:

Lemma 42. *Let H be an arbitrary Hamiltonian such that*

$$H = \sum_{Z:\mathrm{diam}(Z)\le \bar{k}} h_Z, \quad \sum_{Z:Z\ni i} \mathrm{sign}(\|h_{Z,\tau}\|) \le \bar{\kappa}, \tag{673}$$

with $\mathrm{sign}(x)=1$ *($x>0$) and* $\mathrm{sign}(0)=0$. *Also, for a fixed region $X[\bar{R}]$, we assume*

$$\sum_{j=1}^{m} \left\|h_{Z_j}\right\| \le \bar{g}_0 + \bar{g}_1 m \quad \text{s.t.} \quad Z_j \cap Z_{j'} = \emptyset, \quad Z_j \subset X[\bar{R}] \quad \textit{for} \quad \forall j,j' \in [1,m], \tag{674}$$

where $\{Z_j\}_{j=1}^m$ are arbitrarily chosen. Then, the Hamiltonian H satisfies the Lieb-Robinson bound in the form of

$$\|[O_X(H,t),O_Y]\| \le \frac{2|\partial X|}{\gamma \bar{k}^D} \|O_X\|\cdot\|O_Y\| \left[\frac{4e\bar{\kappa}\gamma\bar{k}^{D+1}t}{R}\left(\frac{\bar{k}\bar{g}_0}{R}+\bar{g}_1\right)\right]^{R/\bar{k}}, \quad R=\min(d_{X,Y},\bar{R}-\bar{k}). \tag{675}$$

Remark. We here consider a time-independent Hamiltonian, but the same proof technique is applied to arbitrary time-dependent Hamiltonian as long as $H(\tau)$ $(0\le\tau\le t)$ satisfies the conditions (673) and (674)[*10].

The condition (674) roughly implies that the average energy of m interaction terms $\{h_{Z_j}\}_{j=1}^m$ is given by $\bar{g}_0/m+\bar{g}_1$. If we consider $\mathcal{O}(R)$ interaction terms (i.e., $m=\mathcal{O}(R)$), the average energy is $\mathcal{O}(R^{-1})\bar{g}_0+\bar{g}_1$. Thus, by denoting

$$\frac{\bar{g}_0}{(R/\bar{k})} + \bar{g}_1 = \epsilon_{\mathrm{ave}}, \tag{677}$$

we can rewrite the inequality (675) by

$$\|[O_X(H,t),O_Y]\| \le \frac{2|\partial X|}{\gamma \bar{k}^D} \|O_X\|\cdot\|O_Y\| \left(\frac{C\epsilon_{\mathrm{ave}}t}{R}\right)^{R/\bar{k}}, \tag{678}$$

where C is an $\mathcal{O}(1)$ constant. Therefore, this lemma refines the standard Lieb-Robinson bound so that the Lieb-Robinson velocity is proportional to the *average local energy* instead of the upper bound on the local energy[*11].

1. Proof of Lemma 42

For the proof, we refine the recursion method in the original proof [6, 7]. The key idea is to utilize the following slightly improved inequality:

$$\|[O_X(H,t),O_Y]\| \le 2\|O_X\| \sum_{Z:Z\in\mathcal{S}(X)} \int_0^t \|[h_Z(H_{X^{\mathrm{c}}},x),O_Y]\|\, dx \quad \text{for} \quad [O_X,O_Y]=0, \tag{679}$$

where $\mathcal{S}(X)$ characterizes the set of subsets that overlap with the surface of X as has been defined in Eq. (547). The derivation is given as follows. First of all, we adopt the decomposition of

$$e^{-iHt} = e^{-iH_X t} e^{-iH_{X^{\mathrm{c}}}t} \mathcal{T} e^{-i\int_0^t H_{\partial X}(H_X+H_{X^{\mathrm{c}}},x)dx}, \tag{680}$$

where we define $H_{\partial X} := H - H_{X^{\mathrm{c}}} - H_X = \sum_{Z:Z\in\mathcal{S}(X)} h_Z$. The above equation reduces the norm $\|[O_X(H,t),O_Y]\|$ to

$$\begin{aligned}\|[O_X(H,t),O_Y]\| &= \left\|\left(\mathcal{T}e^{-i\int_0^t H_{\partial X}(H_X+H_{X^{\mathrm{c}}},x)dx}\right)^\dagger e^{iH_{X^{\mathrm{c}}}t} O_X(H_X,t) e^{-iH_{X^{\mathrm{c}}}t}\left(\mathcal{T}e^{-i\int_0^t H_{\partial X}(H_X+H_{X^{\mathrm{c}}},x)dx}\right), O_Y]\right\| \\ &\le 2\|O_X\| \int_0^t \|[H_{\partial X}(H_X+H_{X^{\mathrm{c}}},x),O_Y]\|\,dx = 2\|O_X\|\int_0^t \|[H_{\partial X}(H_{X^{\mathrm{c}}},x),O_Y]\|\,dx,\end{aligned} \tag{681}$$

[*10] For time-dependent Hamiltonian, we need to assume

$$\sum_{j=1}^{m} \left\|h_{Z_j,\tau_j}\right\| \le \bar{g}_0 + \bar{g}_1 m \tag{676}$$

for arbitrary choices of $\{\tau_j\}_{j=1}^m$.

[*11] On the point that the Lieb-Robinson velocity is generally proportional to the local energy, please see Ref. [5, Inequality. (2.4)] for example.

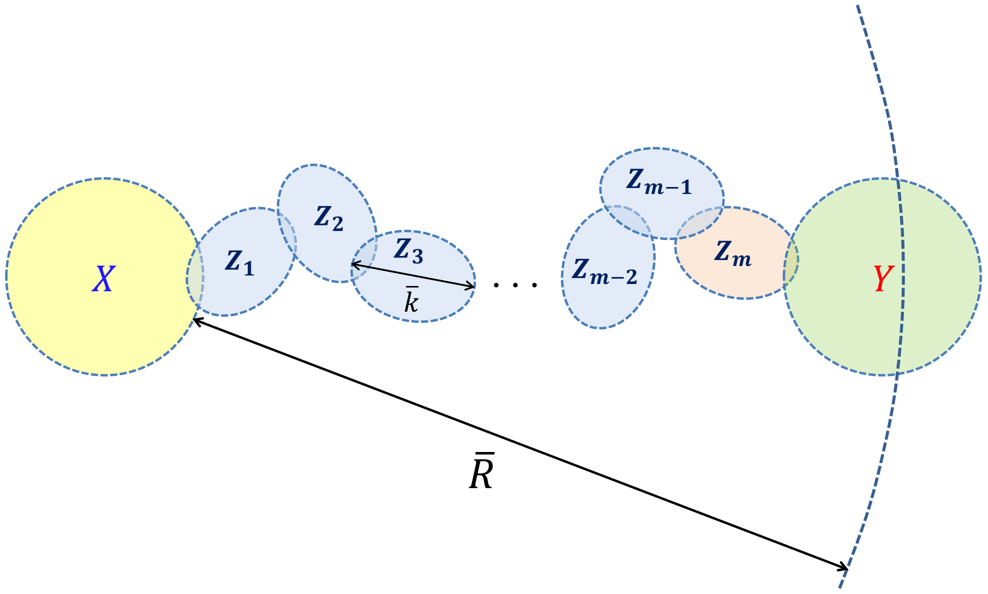

Supplementary Figure 20. Schematic picture of the subsets $\{Z_j\}_{j=1}^m$ that appear in the inequality (684). In the first step, from the inequality (679), the subset Z_1 should overlap with X. In the second step, from the inequality (682), the subset Z_2 has an overlap with Z_1 but no overlap with X. In the third step, from the inequality (683), the subset Z_3 has an overlap with Z_2, but no overlaps with X and Z_1. By repeating the step, the subset Z_{j_0} has overlaps with Z_{j_0-1} and Z_{j_0+1}, but no overlaps with the other subsets $\{Z_j\}_{j\neq j_0-1,j_0,j_0+1}$. This point is critical in efficiently upper-bounding the norm $\|h_{Z_1}\|\cdot\|h_{Z_2}\|\cdots\|h_{Z_m}\|$ by using the original condition (674).

which yields the inequality (679), where the first equation is derived from $[e^{iH_{X^c}t}O_X(H_X,t)e^{-iH_{X^c}t},O_Y] = [O_X(H_X,t),O_Y]=0$, and we use $\|[H_{\partial X}(H_X+H_{X^c},x),O_Y]\| = \|[H_{\partial X}(H_{X^c},x),O_Y(H_X,x)]\| = \|[H_{\partial X}(H_{X^c},x),O_Y]\|$ in the second equation.

By using the inequality (679), as long as $[h_{Z_1},O_Y]=0$, we also obtain

$$\|[h_{Z_1}(H_{X^c},x_1),O_Y]\| \le 2\|h_{Z_1}\| \sum_{\substack{Z_2:Z_2\cap Z_1\neq\emptyset\\ Z_2\cap X=\emptyset}} \int_0^{x_1} \left\|[h_{Z_2}(H_{(X\cup Z_1)^c},x_2),O_Y]\right\| dx_2, \tag{682}$$

where in the summation for Z_2 we pick up interactions in H_{X^c}, which yields the constraint of $Z_2\cap X=\emptyset$. Furthermore, as long as $[h_{Z_2},O_Y]=0$, we have

$$\left\|[h_{Z_2}(H_{(X\cup Z_1)^c},x_2),O_Y]\right\| \le 2\|h_{Z_2}\| \sum_{\substack{Z_3:Z_3\cap Z_2\neq\emptyset\\ Z_3\cap X=\emptyset, Z_3\cap Z_1=\emptyset}} \int_0^{x_2} \left\|[h_{Z_3}(H_{(X\cup Z_1\cup Z_2)^c},x_3),O_Y]\right\| dx_3. \tag{683}$$

By iteratively applying the inequality (679), we obtain (see Fig. 20)

$$\begin{aligned}
&\|[O_X(H,t),O_Y]\| \\
&\le 2^m\|O_X\| \sum_{Z_1:Z_1\in\mathcal{S}(X)} \|h_{Z_1}\| \sum_{\substack{Z_2:Z_2\cap Z_1\neq\emptyset\\ Z_2\cap X=\emptyset}} \|h_{Z_2}\| \sum_{\substack{Z_3:Z_3\cap Z_2\neq\emptyset\\ Z_3\cap X=\emptyset, Z_3\cap Z_1=\emptyset}} \|h_{Z_3}\|\cdots \sum_{\substack{Z_m:Z_m\cap Z_{m-1}\neq\emptyset\\ Z_m\cap X=\emptyset, Z_m\cap Z_1=\emptyset,\ldots,Z_m\cap Z_{m-2}=\emptyset}} \\
&\times \int_0^t\int_0^{x_1}\cdots\int_0^{x_m} \left\|[h_{Z_m}(H_{(X\cup Z_1\cup Z_2\cup\cdots\cup Z_{m-1})^c},x_m),O_Y]\right\| dx_1dx_2\cdots dx_m, \\
&\le \frac{2(2t)^m}{m!}\|O_X\|\cdot\|O_Y\| \sum_{Z_1:Z_1\in\mathcal{S}(X)} \|h_{Z_1}\| \sum_{\substack{Z_2:Z_2\cap Z_1\neq\emptyset\\ Z_2\cap X=\emptyset}} \|h_{Z_2}\|\cdots \sum_{\substack{Z_m:Z_m\cap Z_{m-1}\neq\emptyset\\ Z_m\cap X=\emptyset, Z_m\cap Z_1=\emptyset,\ldots,Z_m\cap Z_{m-2}=\emptyset}} \|h_{Z_m}\|,
\end{aligned} \tag{684}$$

where we assume $[h_{Z_{m-1}},O_Y]=0$ and $Z_m\in Z[\bar{R}]$. Because of the constraints $[h_{Z_{m-1}},O_Y]=0$ and $Z_m\in Z[\bar{R}]$, we choose the parameter m as

$$m=\min\left(\left\lfloor\frac{d_{X,Y}}{\bar{k}}\right\rfloor+1, \left\lfloor\frac{\bar{R}}{\bar{k}}\right\rfloor\right) \ge \frac{R}{\bar{k}} \tag{685}$$

with $R:=\min(d_{X,Y},\bar{R}-\bar{k})$ because of $\mathrm{diam}(Z_j)\le\bar{k}$ from the condition in Eq. (673).

We then aim to upper-bound the norm of

$$\|h_{Z_1}\| \cdot \|h_{Z_2}\| \cdots \|h_{Z_m}\| \le \left(\frac{\sum_{j=1}^m \|h_{Z_j}\|}{m}\right)^m \tag{686}$$

under the condition that

$$Z_j \cap Z_{j-1} \neq \emptyset, \quad Z_j \cap X = \emptyset, \quad Z_j \cap Z_1 = \emptyset, \quad \dots, \quad Z_j \cap Z_{j-2} = \emptyset \quad \text{for} \quad \forall j \in [m],$$

where we use the inequality of arithmetic and geometric means. Note that from the above condition we have $Z_j \cap Z_{j'} = \emptyset$ as long as $|j - j'| \neq 1$. Therefore, by using the condition (674), we have

$$\begin{aligned}\sum_{j=1}^m \|h_{Z_j}\| &= \sum_{j:\text{odd}} \|h_{Z_j}\| + \sum_{j:\text{even}} \|h_{Z_j}\| \\ &\le \bar{g}_0 + \sum_{j:\text{odd}} \bar{g}_1 + \bar{g}_0 + \sum_{j:\text{even}} \bar{g}_1 \\ &\le 2\bar{g}_0 + \bar{g}_1 m.\end{aligned} \tag{687}$$

By combining the inequalities (686) and (687), we have

$$\|h_{Z_1}\| \cdot \|h_{Z_2}\| \cdots \|h_{Z_m}\| \le \left(\frac{2\bar{g}_0}{m} + \bar{g}_1\right)^m. \tag{688}$$

Finally, for an arbitrary subset $Z \subset \Lambda$ with $\mathrm{diam}(Z) \le \bar{k}$, by using the condition (673), we have

$$\sum_{Z':Z'\cap Z\neq\emptyset} \mathrm{sign}\left(\|h_{Z'}\|\right) \le \sum_{i\in Z} \sum_{Z':Z'\ni i} \mathrm{sign}\left(\|h_{Z'}\|\right) \le \bar{\kappa}|Z| \le \bar{\kappa}\gamma\bar{k}^D, \tag{689}$$

where we use $|Z| \le \gamma\bar{k}^D$ from $\mathrm{diam}(Z) \le \bar{k}$. In the same way, we obtain

$$\sum_{Z:Z\in\mathcal{S}(X)} \mathrm{sign}\left(\|h_Z\|\right) \le \sum_{i\in\partial X} \sum_{Z:Z\ni i} \mathrm{sign}\left(\|h_Z\|\right) \le \bar{\kappa}|\partial X|. \tag{690}$$

Thus, by using the inequalities (687) and (690), we reduce the inequality (684) to

$$\begin{aligned}\|[O_X(H,t), O_Y]\| &\le \frac{2(2t)^m}{m!} \|O_X\| \cdot \|O_Y\| \cdot \bar{\kappa}|\partial X| \cdot \left(\bar{\kappa}\gamma\bar{k}^D\right)^{m-1} \left(\frac{2\bar{g}_0}{m} + \bar{g}_1\right)^m \\ &= \frac{2|\partial X|}{m! \cdot \gamma\bar{k}^D} \|O_X\| \cdot \|O_Y\| \left[2\bar{\kappa}\gamma\bar{k}^D t \left(\frac{2\bar{g}_0}{m} + \bar{g}_1\right)\right]^m \\ &\le \frac{2|\partial X|}{\gamma\bar{k}^D} \|O_X\| \cdot \|O_Y\| \left[\frac{4e\bar{\kappa}\gamma\bar{k}^D t}{m} \left(\frac{\bar{g}_0}{m} + \bar{g}_1\right)\right]^m,\end{aligned} \tag{691}$$

where we use $m! \le (m/e)^m$ in the last inequality. Under the choice of (685), we reduce the inequality (691) to (675). This completes the proof. □

[End of Proof of Lemma 42]

The final task is to obtain all the parameters in Lemma 42 to apply them to our purpose [i.e., upper-bounding the norm (672)]. First of all, from Eq. (663), the interaction length of the Hamiltonian $\tilde{H}_\tau$ at most $(2k)$, i.e.,

$$\bar{k} \to 2k. \tag{692}$$

Also, the constant $\bar{\kappa}$ characterizes how many interaction terms h_Z such that $Z \ni i$ exists in the Hamiltonian. For the case of $\tilde{H}_\tau$ in Eq. (669), the $\bar{\kappa}$ is given by

$$\bar{\kappa} = \max_{i_0\in\Lambda} \#\left\{\langle i,j\rangle | \mathcal{B}_{i,j} \ni i_0\right\} \le \max_{i_0\in\Lambda} \left(\gamma|i_0[k]|\right) \le \gamma^2 k^D, \tag{693}$$

because $\mathcal{B}_{i,j} = i[k-1] \cup j[k-1] \ni i_0$ $(d_{i,j} = 1)$ implies that the sites i and j should be included in $i_0[k]$. Therefore, we have

$$\bar{\kappa} \to \gamma^2 k^D. \tag{694}$$

The remaining parameters are $\bar{g}_0$ and $\bar{g}_1$ in the condition (674). For this purpose, we need to calculate the upper bound of

$$\sum_{s=1}^{m} \left\| h_{\mathcal{B}_{i_s,j_s}} \right\| \le 2\bar{J} \sum_{s=1}^{m} (q_{i_s} + q_{j_s}) \quad (\mathcal{B}_{i_s,j_s} \subseteq \tilde{L}',\ \mathcal{B}_{i_s,j_s} \cap \mathcal{B}_{i_{s'},j_{s'}} = \emptyset), \tag{695}$$

where the inequality is derived from (666). By using Lemma 41 with $Y = \{i_s, j_s\}_{s=1}^m$ ($|Y| = 2m$), we upper-bound

$$2\bar{J} \sum_{s=1}^{m} (q_{i_s} + q_{j_s}) \le 2e\bar{J}(4 + \mu_0 \lambda_{3/4}) Q + 4e\bar{J} \left(4\mu_1 \tilde{q} + 4\bar{q} + \mu_0 \lambda_{3/4} \bar{q}\right) m, \tag{696}$$

which gives

$$\bar{g}_0 \to 2e\bar{J}(4 + \mu_0 \lambda_{3/4}) Q, \quad \bar{g}_1 \to 4e\bar{J} \left(4\mu_1 \tilde{q} + 4\bar{q} + \mu_0 \lambda_{3/4} \bar{q}\right). \tag{697}$$

Finally, the length $\bar{R}$ is defined by the region such that the condition (674) is satisfied. Hence, in our setup, we have to choose the length $\bar{R}$ so that we can ensure the condition of

$$\mathcal{B}_{i,j}[\bar{R}] \subseteq \tilde{L}' \quad \text{for} \quad \forall \mathcal{B}_{i,j} \in \mathcal{S}_{Z_0[\tilde{\ell}_0]}. \tag{698}$$

Because of $\mathrm{diam}(\mathcal{B}_{i,j}) < 2k$ and $\tilde{\ell}_0 = \ell_0 - (\delta\ell + 2k + 1)$ as in Eq. (670), we have to set $\bar{R}$ as $\ell_0 - (2\delta\ell + 2 + 4k)$ (see Fig. 19), i.e.,

$$\bar{R} \to \ell_0 - (2\delta\ell + 2 + 4k). \tag{699}$$

Based on the above parameter correspondences, we can prove the following corollary:

Corollary 43. *For the operator of $\tilde{h}_{\mathcal{B}_{i,j},x}$ ($\mathcal{B}_{i,j} \in \mathcal{S}_{Z_0[\tilde{\ell}_0]}$), we obtain the Lieb-Robinson bound as follows:*

$$\left\| \left[\tilde{h}_{\mathcal{B}_{i,j},x}(\tilde{H}_{Z_0[\tilde{\ell}_0],x}, x \to 0), \tilde{O}_{Z_0[k]} \right] \right\| \le \frac{2\zeta_{Z_0} |\mathcal{B}_{i,j}|}{\gamma(2k)^D} \left\| \tilde{h}_{\mathcal{B}_{i,j},x} \right\| \left[\frac{\tilde{c}_1 x}{\ell_0} \left(\frac{\tilde{c}_2 Q}{\ell_0} + \tilde{c}_{\tilde{q},\bar{q}} \right) \right]^{\ell_0/(4k)}, \tag{700}$$

where $\tilde{c}_1$, $\tilde{c}_2$ and $\tilde{c}_3$ are defined as

$$\tilde{c}_1 := 2^{D+6} e^2 \gamma^3 k^{2D+1} \bar{J}, \quad \tilde{c}_2 := 2k(4 + \mu_0 \lambda_{3/4}), \quad \tilde{c}_{\tilde{q},\bar{q}} := 4\mu_1 \tilde{q} + 4\bar{q} + \mu_0 \lambda_{3/4} \bar{q}. \tag{701}$$

2. *Proof of Corollary 43*

We first notice that the parameter R in the inequality (675) satisfies $R = \min(d_{\mathcal{B}_{i,j},Z_0[k]}, \bar{R} - 2k) = \bar{R} - 2k$ because of $\mathcal{B}_{i,j} \in \mathcal{S}_{Z_0[\tilde{\ell}_0]}$, $\mathrm{diam}(\mathcal{B}_{i,j}) \le 2k$ and $\bar{R} = \ell_0 - (2\delta\ell + 2 + 4k)$ [see Eq. (699)]. We then obtain

$$\bar{R} - 2k = \ell_0 - (2\delta\ell + 2 + 6k) \ge \frac{\ell_0}{2}, \tag{702}$$

where the inequality results from the condition (660). Hence, under the choice of (699), we replace $R = \min(d_{X,Y}, \bar{R} - \bar{k})$ in (675) as

$$R \to \frac{\ell_0}{2}. \tag{703}$$

Then, by applying the replacements (692), (694), (699), and (697) to the inequality (675), we have the inequality (700) as follows:

$$\begin{aligned} &\left\| \left[\tilde{h}_{\mathcal{B}_{i,j},x}(\tilde{H}_{Z_0[\tilde{\ell}_0],x}, x \to 0), \tilde{O}_{Z_0[k]} \right] \right\| \\ &\le \frac{2|\mathcal{B}_{i,j}|}{\gamma(2k)^D} \left\| \tilde{h}_{\mathcal{B}_{i,j},x} \right\| \cdot \left\| \tilde{O}_{Z_0[k]} \right\| \left[\frac{4e\left(\gamma^2 k^D\right) \gamma(2k)^{D+1} x}{(\ell_0/2)} \left(\frac{(2k \cdot 2e\bar{J}(4 + \mu_0 \lambda_{3/4}) Q}{(\ell_0/2)} + 4e\bar{J} \left(4\mu_1 \tilde{q} + 4\bar{q} + \mu_0 \lambda_{3/4} \bar{q}\right) \right) \right]^{(\ell_0/2)/(2k)} \\ &= \frac{2\zeta_{Z_0} |\mathcal{B}_{i,j}|}{\gamma(2k)^D} \left\| \tilde{h}_{\mathcal{B}_{i,j},x} \right\| \left[\frac{2^{D+6} e^2 \gamma^3 k^{2D+1} \bar{J} x}{\ell_0} \left(\frac{2k(4 + \mu_0 \lambda_{3/4}) Q}{\ell_0} + 4\mu_1 \tilde{q} + 4\bar{q} + \mu_0 \lambda_{3/4} \bar{q} \right) \right]^{\ell_0/(4k)}, \end{aligned} \tag{704}$$

where we use $\left\| \tilde{O}_{Z_0[k]} \right\| = \zeta_{Z_0}$ from Eq. (668). Then, by using the definitions in (701), we reduce the above inequality to the main inequality (700). This completes the proof. □

———————————————————————————[**End of Proof of Corollary 43**]

By applying Corollary 43 to the inequality (671), we obtain

$$\begin{aligned}
&\left\|\tilde{O}_{Z_0[k]}(\tilde{H}_x, 0\to\tau) - \tilde{O}_{Z_0[k]}(\tilde{H}_{Z_0[\tilde{\ell}_0],x}, 0\to\tau)\right\| \\
&\le \sum_{\langle i,j\rangle:\mathcal{B}_{i,j}\in\mathcal{S}_{Z_0[\tilde{\ell}_0]}} \int_0^\tau \frac{2\zeta_{Z_0}|\mathcal{B}_{i,j}|}{\gamma(2k)^D} \left\|\tilde{h}_{\mathcal{B}_{i,j},x}\right\| \left[\frac{\tilde{c}_1 x}{\ell_0}\left(\frac{\tilde{c}_2 Q}{\ell_0} + \tilde{c}_{\bar{q},\bar{q}}\right)\right]^{\ell_0/(4k)} dx \\
&\le \sum_{\langle i,j\rangle:\mathcal{B}_{i,j}\in\mathcal{S}_{Z_0[\tilde{\ell}_0]}} \frac{2\tau\zeta_{Z_0}\cdot 2\gamma k^D}{\gamma(2k)^D}\cdot 2\bar{J}(q_i+q_j)\left[\frac{\tilde{c}_1 \tau}{\ell_0}\left(\frac{\tilde{c}_2 Q}{\ell_0} + \tilde{c}_{\bar{q},\bar{q}}\right)\right]^{\ell_0/(4k)} \\
&\le \frac{16\gamma\bar{J}\tau\zeta_{Z_0}}{2^D}\bar{N}_{\tilde{L}}\left[\frac{\tilde{c}_1 \tau}{\ell_0}\left(\frac{\tilde{c}_2 Q}{\ell_0} + \tilde{c}_{\bar{q},\bar{q}}\right)\right]^{\ell_0/(4k)},
\end{aligned} \tag{705}$$

where in the second inequality, we use $|\mathcal{B}_{i,j}| \le |i[k]| + |j[k]| \le 2\gamma k^D$ from $\mathcal{B}_{i,j} = i[k-1]\cup j[k-1]$ and $\left\|h_{\mathcal{B}_{i,j},\tau}\right\| \le 2\bar{J}(q_i+q_j)$ as in (666), and in the last inequality, we use

$$\sum_{\langle i,j\rangle:\mathcal{B}_{i,j}\in\mathcal{S}_{Z_0[\tilde{\ell}_0]}} (q_i+q_j) \le 2\sum_{i\in\tilde{L}}\gamma q_i \le 2\gamma\bar{N}_{\tilde{L}} \tag{706}$$

with $\bar{N}_{\tilde{L}}$ defined in Eq. (638).

Finally, we can estimate the norm (661). Because the operators $\tilde{V}_{Z_0[k]}[\tilde{L}',\mathbf{q}]$ and $\tilde{H}_{Z_0[\tilde{\ell}_0],x}$ are supported on $Z_0[\tilde{\ell}_0]\subseteq L_0 = Z_0[\ell_0]$, by choosing U_{L_0} as

$$U_{L_0} = e^{-i\tilde{V}_{Z_0[k]}[\tilde{L}',\mathbf{q}]\tau}\,\mathcal{T}\exp\left(-i\int_0^\tau \tilde{H}_{Z_0[\tilde{\ell}_0],x}d\tau\right), \tag{707}$$

we have

$$U_{L_0}^\dagger O_{Z_0} U_{L_0} = \tilde{O}_{Z_0[k]}(\tilde{H}_{Z_0[\tilde{\ell}_0],x}, 0\to\tau), \tag{708}$$

where $\tilde{H}_{Z_0[\tilde{\ell}_0],x}$ has been defined by Eq. (663) and Eq. (669). By using the above choice of U_{L_0}, we obtain

$$\left\|O_{Z_0}\left(\tilde{H}[\tilde{L}',\mathbf{q}],\tau\right) - U_{L_0}^\dagger O_{Z_0} U_{L_0}\right\| \le \frac{16\gamma\bar{J}\tau\zeta_{Z_0}}{2^D}\bar{N}_{\tilde{L}}\left[\frac{\tilde{c}_1 \tau}{\ell_0}\left(\frac{\tilde{c}_2 Q}{\ell_0} + \tilde{c}_{\bar{q},\bar{q}}\right)\right]^{\ell_0/(4k)} = \delta_2, \tag{709}$$

where use the definition (578).

By combining the inequalities (637) and (709), we prove the main inequality (576). This completes the proof of Proposition 36. □

F. Generalization: small time evolution by effective Hamiltonian

We here consider the same setup as Subtheorem 2 and generalize the statement to the effective Hamiltonian as

$$\bar{\Pi}H\bar{\Pi},\quad \bar{\Pi} = \Pi_{\bar{L}\setminus Z_0,I}\Pi_{\mathcal{S}(\bar{L},1/2,N_0)}\quad (I\subset\mathbb{N}), \tag{710}$$

where $\Pi_{\bar{L}\setminus Z_0,I}$ is a projection onto the eigenspace of $\hat{n}_{\bar{L}\setminus Z_0}$ with eigenvalues in the range of $I\subset\mathbb{N}$. Our purpose is to approximate the time evolution $O_{Z_0}(\bar{\Pi}H\bar{\Pi},\tau)$ onto the region $L_0 = Z_0[\ell_0] \subseteq \bar{L}$ (see Fig. 21). As has been discussed in Sec. Supplementary Note 6 D, the interactions in the Hamiltonian $\bar{\Pi}H\bar{\Pi}$ are still local in the region of $\bar{L}\setminus Z_0$ and $(\bar{L}\setminus Z_0)^{\rm c}$ (see Corollary 31), whereas boundary interactions around $\partial(\bar{L}\setminus Z_0)$ is non-local.

Then, we have to carefully treat the boundary interaction terms $\partial h_{\bar{L}}$ and ∂h_{Z_0} [see Eq. (28) for their notations]. As shown in Fig. 21, the hopping term

$$\bar{\Pi}b_i b_j^\dagger\bar{\Pi} = \Pi_{\mathcal{S}(\bar{L},1/2,N_0)}\Pi_{\bar{L}\setminus Z_0,I}b_i b_j^\dagger\Pi_{\bar{L}\setminus Z_0,I}\Pi_{\mathcal{S}(\bar{L},1/2,N_0)}\qquad \text{for}\quad i\in\bar{L}\setminus Z_0,\quad j\in(\bar{L}\setminus Z_0)^{\rm c} \tag{711}$$

is non-local in the region $\bar{L}\setminus Z_0$. That is, for the above pair of i,j, we have a commutation relation of

$$\left[\bar{\Pi}b_i b_j^\dagger\bar{\Pi}, \bar{\Pi}b_{i'} b_{j'}^\dagger\bar{\Pi}\right] \ne 0 \quad \text{for}\quad i',j'\in\bar{L}\setminus Z_0. \tag{712}$$

Note that we trivially obtain

$$\left[\bar{\Pi}b_i b_j^\dagger\bar{\Pi}, \bar{\Pi}b_{i''} b_{j''}^\dagger\bar{\Pi}\right] = 0 \quad \text{for}\quad i'',j''\in(\bar{L}\setminus Z_0)^{\rm c},\quad \{i'',j''\}\cap\{i,j\}=\emptyset. \tag{713}$$

In proving the optimal Lieb-Robinson bound in one-dimensional systems, we need to extend the statement of Subtheorem 2 to the effective Hamiltonian $\bar{\Pi}H\bar{\Pi}$ (see Sec. Supplementary Note 10 E 1). We here prove the following generalization:

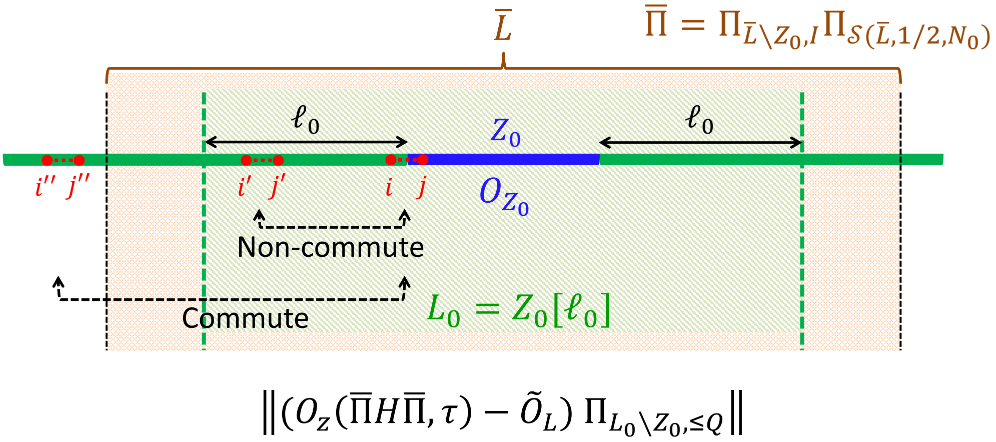

Supplementary Figure 21. Schematic picture of the setup of Proposition 44. Here, we consider the effective Hamiltonian (710) that is spanned by the projection $\bar{\Pi}$. The projection $\bar{\Pi}$ includes a non-local projection $\Pi_{\bar{L}\setminus Z_0,I}$ onto the region $\bar{L}\setminus Z_0$. Such a setup is indeed used in proving the optimal Lieb-Robinson bound in one-dimensional systems (see also Fig. 24). The difficulty here is that the locality of the interaction no longer holds at the boundary of $\bar{L}$ as in Eq. (712). Even in this situation, we can prove the same statement as Subtheorem 2.

Proposition 44. *Let us adopt the same setup as Subtheorem 2. Then, for $\tau \le 1/(4\gamma \bar{J})$, we have the following approximation error by using the subset Hamiltonian $\bar{\Pi}H_{L_0}\bar{\Pi}$ as*

$$\begin{aligned}&\left\| \left[O_{Z_0}(\bar{\Pi}H\bar{\Pi},\tau) - O_{Z_0}(\bar{\Pi}H_{L_0}\bar{\Pi},\tau)\right] \Pi_{\bar{L},\le Q} \right\| \\ &\le \zeta_{Z_0}\Theta(Q^4 N_0 \bar{L}) \left\{ e^{-\Theta(\ell_0)} + e^{\Theta(\ell_0)} \left[\Theta(\tau) + \Theta\left(\frac{\tau Q\log(Q)}{\ell_0^2}\right)\right]^{\Theta(\ell_0)} \right\},\end{aligned} \tag{714}$$

where $\ell_0 \ge \log[\Theta\left[\log(q_{Z_0}N_0)\right]]$.

G. Proof of Proposition 44

In the proof, we omit the projection $\bar{\Pi}$ and simply denote it as

$$\bar{\Pi}O\bar{\Pi} \to \grave{O} \tag{715}$$

for an arbitrary operator O. For the proof, we need to prove Proposition 36 for the effective Hamiltonian. First of all, we have the same statement as Subtheorem 1 for the effective Hamiltonian $\bar{\Pi}H\bar{\Pi}$ (see Corollary 4). Therefore, we can apply the same proof techniques in Secs. Supplementary Note 8 C and Supplementary Note 8 D to this case. In the following, we consider how the proof in Sec. Supplementary Note 8 E should be modified.

We here adopt the same notations as those in Sec. Supplementary Note 8 E. We separately treat the non-local interaction terms and start from the decomposition of

$$\begin{aligned}\tilde{\grave{H}}_0[\tilde{L}',\mathbf{q}] &= \bar{\Pi}_{\tilde{L}',\mathbf{q}}\grave{H}\bar{\Pi}_{\tilde{L}',\mathbf{q}} \\ &= \tilde{\grave{H}}_{0,Z_0}[\tilde{L}',\mathbf{q}] + \tilde{\grave{H}}_{0,\bar{L}\setminus Z_0}[\tilde{L}',\mathbf{q}] + \tilde{\grave{H}}_{0,\bar{L}^c}[\tilde{L}',\mathbf{q}] + \partial\tilde{\grave{h}}_{\bar{L}}[\tilde{L}',\mathbf{q}] + \partial\tilde{\grave{h}}_{Z_0}[\tilde{L}',\mathbf{q}],\end{aligned} \tag{716}$$

where we have defined $\bar{\Pi}_{\tilde{L}',\mathbf{q}} := \prod_{i\in\tilde{L}'}\Pi_{i,\le q_i}$ and $\tilde{L}' = \tilde{L}[-1]$. Note that the boundary interaction terms $\partial\tilde{\grave{h}}_{\bar{L}}[\tilde{L}',\mathbf{q}]$ and $\partial\tilde{\grave{h}}_{Z_0}[\tilde{L}',\mathbf{q}]$ are non-local in the region $\bar{L}\setminus Z_0$. As in Eq. (663), we define $\tilde{\grave{H}}_x$ as

$$\begin{aligned}\tilde{\grave{H}}_x := e^{i\tilde{\grave{V}}[\tilde{L}',\mathbf{q}]x}\tilde{\grave{H}}_0[\tilde{L}',\mathbf{q}]e^{-i\tilde{\grave{V}}[\tilde{L}',\mathbf{q}]x} &= \sum_{\langle i,j\rangle}\tilde{\grave{h}}_{\mathcal{B}_{i,j},x}, \quad \mathcal{B}_{i,j} = i[k-1]\cup j[k-1], \quad \mathrm{diam}(\mathcal{B}_{i,j}) \le 2k \\ &= \tilde{\grave{H}}'_x + \partial\tilde{\grave{h}}_{\bar{L},x} + \partial\tilde{\grave{h}}_{Z_0,x},\end{aligned} \tag{717}$$

where in the last decomposition, we use the notations of

$$\begin{aligned}\tilde{H}'_x &:= e^{i\tilde{V}[\tilde{L}',\mathbf{q}]x}\left(\tilde{H}_{0,Z_0}[\tilde{L}',\mathbf{q}] + \tilde{H}_{0,\bar{L}\setminus Z_0}[\tilde{L}',\mathbf{q}] + \tilde{H}_{0,\bar{L}^c}[\tilde{L}',\mathbf{q}]\right)e^{-i\tilde{V}[\tilde{L}',\mathbf{q}]x} \\ &= \sum_{\langle i,j\rangle:\{i,j\}\subset Z_0}\tilde{h}_{\mathcal{B}_{i,j},x} + \sum_{\langle i,j\rangle:\{i,j\}\subset \bar{L}\setminus Z_0}\tilde{h}_{\mathcal{B}_{i,j},x} + \sum_{\langle i,j\rangle:\{i,j\}\subset \bar{L}^c}\tilde{h}_{\mathcal{B}_{i,j},x},\end{aligned} \tag{718}$$

and

$$\partial\tilde{h}_{\bar{L},x} := e^{i\tilde{V}[\tilde{L}',\mathbf{q}]x}\partial\tilde{h}_{\bar{L}}[\tilde{L}',\mathbf{q}]e^{-i\tilde{V}[\tilde{L}',\mathbf{q}]x}, \quad \partial\tilde{h}_{Z_0,x} := e^{i\tilde{V}[\tilde{L}',\mathbf{q}]x}\partial\tilde{h}_{Z_0}[\tilde{L}',\mathbf{q}]e^{-i\tilde{V}[\tilde{L}',\mathbf{q}]x}. \tag{719}$$

Here, the Hamiltonian $\grave{\tilde{H}}'_x$ only includes local interactions in the sense that $\left[\grave{\tilde{h}}_{\mathcal{B}_{i,j},x}, \grave{\tilde{h}}_{\mathcal{B}_{i',j'},x}\right] = 0$ as long as $\{i,j\} \cap \{i',j'\} = \emptyset$. Also, we notice that $\tilde{H}'_x$ commute with $\bar{\Pi}$ in Eq. (710), i.e.,

$$[\tilde{H}'_x, \bar{\Pi}] = 0 \longrightarrow \grave{\tilde{H}}'_x = \tilde{H}'_x\bar{\Pi} = \bar{\Pi}\tilde{H}'_x \tag{720}$$

By using Eq. (717), we obtain a similar decomposition to Eq. (662) as

$$\begin{aligned} e^{-i\grave{\tilde{H}}[\tilde{L}',\mathbf{q}]\tau} &= e^{-i\grave{\tilde{V}}[\tilde{L}',\mathbf{q}]\tau}U_{\grave{\tilde{H}}_x,0\to\tau} = e^{-i\grave{\tilde{V}}[\tilde{L}',\mathbf{q}]\tau}\,\mathcal{T}\exp\left(-i\int_0^\tau \grave{\tilde{H}}_x dx\right) \\ &= e^{-i\grave{\tilde{V}}[\tilde{L}',\mathbf{q}]\tau}U_{\grave{\tilde{H}}'_x,0\to\tau}\mathcal{T}\exp\left[-i\int_0^\tau \partial\grave{\tilde{h}}_{\bar{L},\tau'}\left(\grave{\tilde{H}}'_x,\tau'\to\tau\right) + \partial\grave{\tilde{h}}_{Z_0,\tau'}\left(\grave{\tilde{H}}'_x,\tau'\to\tau\right)d\tau'\right], \end{aligned} \tag{721}$$

where we use the notations in Eq. (33), and the last equation is derived from

$$\mathcal{T}e^{-i\int_0^\tau (A_{\tau'}+B_{\tau'})d\tau'} = U_{A_x,0\to\tau}\mathcal{T}e^{-i\int_0^\tau B_{\tau'}(A_x,\tau'\to\tau)d\tau'} \tag{722}$$

for arbitrary time-dependent operators A_x and B_s.

By using the expression (721), we aim to analyze the following time evolution:

$$O_{Z_0}\left(\grave{\tilde{H}}[\tilde{L}',\mathbf{q}],\tau\right) = U_\partial^\dagger\tilde{O}_{Z_0[k]}\left(\grave{\tilde{H}}'_x, 0\to\tau\right)U_\partial, \tag{723}$$

where $\tilde{O}_{Z_0[k]}$ ($\|\tilde{O}_{Z_0[k]}\| = \|O_{Z_0}\| = \zeta_{Z_0}$) has been defined in Eq. (668), i.e.,

$$\tilde{O}_{Z_0[k]} := O_{Z_0}\left(\grave{\tilde{V}}[\tilde{L}',\mathbf{q}],\tau\right) = e^{i\grave{\tilde{V}}_{Z_0[k]}[\tilde{L}',\mathbf{q}]\tau}O_{Z_0}e^{-i\grave{\tilde{V}}_{Z_0[k]}[\tilde{L}',\mathbf{q}]\tau}, \quad [\tilde{O}_{Z_0[k]},\bar{\Pi}] = 0, \tag{724}$$

and U_∂ is defined as

$$U_\partial = \mathcal{T}\exp\left[-i\int_0^\tau \partial\grave{\tilde{h}}_{\bar{L},\tau'}\left(\grave{\tilde{H}}'_x,\tau'\to\tau\right) + \partial\grave{\tilde{h}}_{Z_0,\tau'}\left(\grave{\tilde{H}}'_x,\tau'\to\tau\right)d\tau'\right]. \tag{725}$$

In Eq. (723), we separately approximate the terms $U_\partial^\dagger$, $\tilde{O}_{Z_0[k]}\left(\grave{\tilde{H}}'_x, 0\to\tau\right)$, and U_∂.

In the following, we denote the approximation of U_∂ by $\tilde{U}_\partial$, which will be given in Eq. (731) explicitly. By using the notation of $\tilde{U}_\partial$, we obtain

$$\begin{aligned} &\left\|O_{Z_0}\left(\grave{\tilde{H}}[\tilde{L}',\mathbf{q}],\tau\right) - \tilde{U}_\partial^\dagger\tilde{O}_{Z_0[k]}\left(\grave{\tilde{H}}'_{Z_0[\ell_1],x},0\to\tau\right)\tilde{U}_\partial\right\| \\ &= \left\|U_\partial^\dagger\tilde{O}_{Z_0[k]}\left(\grave{\tilde{H}}'_x,0\to\tau\right)U_\partial - \tilde{U}_\partial^\dagger\tilde{O}_{Z_0[k]}\left(\grave{\tilde{H}}'_{Z_0[\ell_1],x},0\to\tau\right)\tilde{U}_\partial\right\| \\ &\le \left\|\tilde{O}_{Z_0[k]}\left(\grave{\tilde{H}}'_x,0\to\tau\right) - \tilde{O}_{Z_0[k]}\left(\grave{\tilde{H}}'_{Z_0[\ell_1],x},0\to\tau\right)\right\| \\ &\quad + \left\|U_\partial^\dagger\tilde{O}_{Z_0[k]}\left(\grave{\tilde{H}}'_{Z_0[\ell_1],x},0\to\tau\right)U_\partial - \tilde{U}_\partial^\dagger\tilde{O}_{Z_0[k]}\left(\grave{\tilde{H}}'_{Z_0[\ell_1],x},0\to\tau\right)\tilde{U}_\partial\right\| \\ &\le \left\|\tilde{O}_{Z_0[k]}\left(\grave{\tilde{H}}'_x,0\to\tau\right) - \tilde{O}_{Z_0[k]}\left(\grave{\tilde{H}}'_{Z_0[\ell_1],x},0\to\tau\right)\right\| + 2\zeta_{Z_0}\left\|U_\partial - \tilde{U}_\partial\right\|, \end{aligned} \tag{726}$$

where we use $\left\|\tilde{O}_{Z_0[k]}\left(\grave{\tilde{H}}'_{Z_0[\ell_1],x},0\to\tau\right)\right\| = \|O_{Z_0}\| = \zeta_{Z_0}$, and the length ℓ_1 is appropriately chosen afterward. Also, for the last inequality in (726), we used

$$\begin{aligned} \left\|U_1^\dagger O U_1 - U_2^\dagger O U_2\right\| = \left\|U_1^\dagger O U_1 - U_2^\dagger O U_1 + U_2^\dagger O U_1 - U_2^\dagger O U_2\right\| &\le \left\|(U_1-U_2)^\dagger O U_1\right\| + \left\|U_2^\dagger O(U_1-U_2)\right\| \\ &\le 2\|O\|\cdot\|U_1-U_2\|. \end{aligned} \tag{727}$$

Here, $\grave{\tilde{H}}'_{Z_0[\ell_1],x}$ is the subset Hamiltonian for $\grave{\tilde{H}}'_x$ in which we pick up the interaction terms in (718) such that $\mathcal{B}_{i,j} \subset Z_0[\ell_1]$.

To estimate the RHS of the inequality (726), we first aim to consider the approximation of $\tilde{O}_{Z_0[k]}\left(\grave{\tilde{H}}'_x, 0\to\tau\right)$ onto the region of $Z_0[\ell_1]$, where we assume that ℓ_1 is smaller than $\tilde{\ell}_0$ which has been defined in Eq. (670), i.e.,

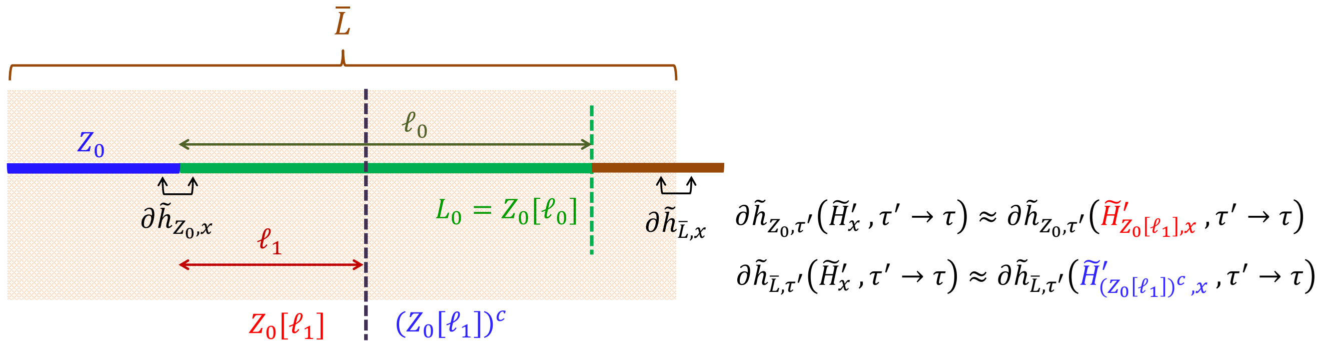

Supplementary Figure 22. Schematic picture of the approximations for $\partial\tilde{h}_{Z_0,\tau'}\left(\tilde{H}'_x, \tau' \to \tau\right)$ and $\partial\tilde{h}_{\bar{L},\tau'}\left(\tilde{H}'_x, \tau' \to \tau\right)$ in Eq. (730), where $\partial\tilde{h}_{\bar{L},x}$ and $\partial\tilde{h}_{Z_0,x}$ are defined in Eq. (717). The length ℓ_1 is a free parameter that is introduced in the inequality (726).

$\tilde{\ell}_0 = \ell_0 - (\delta\ell + 2k + 1)$. Because the Hamiltonian $\tilde{\tilde{H}}'_x$ does not include the non-local interactions, we can obtain the same approximation error as Eq. (705), which yields

$$\left\|\tilde{O}_{Z_0[k]}\left(\tilde{\tilde{H}}'_x, 0 \to \tau\right) - \tilde{O}_{Z_0[k]}\left(\tilde{\tilde{H}}'_{Z_0[\ell_1],x}, 0 \to \tau\right)\right\| \le \zeta_{Z_0}\Theta(\tau)\bar{N}_{\tilde{L}}\left\{\frac{\tau}{\ell_1}\left[\Theta(Q/\ell_1) + \Theta(\tilde{q}) + \Theta(\bar{q})\right]\right\}^{\Theta(\ell_1)}, \tag{728}$$

where $\ell_1 \ge \Theta(\delta\ell)$ that originates from a similar condition to (660).

We next estimate the error $\|U_\partial - \tilde{U}_\partial\|$. We first notice that because of Eq. (720)

$$\partial\tilde{\tilde{h}}_{Z_0,\tau'}\left(\tilde{\tilde{H}}'_x, \tau' \to \tau\right) = \bar{\Pi}\tilde{h}_{Z_0,\tau'}\left(\tilde{H}'_x, \tau' \to \tau\right)\bar{\Pi}. \tag{729}$$

The same equation holds for $\partial\tilde{\tilde{h}}_{\bar{L},\tau'}\left(\tilde{\tilde{H}}'_x, \tau' \to \tau\right)$. We then adopt the following approximations (see also Fig. 22):

$$\begin{aligned}
&\partial\tilde{h}_{Z_0,\tau'}\left(\tilde{H}'_x, \tau' \to \tau\right) \approx \partial\tilde{h}_{Z_0,\tau'}\left(\tilde{H}'_{Z_0[\ell_1],x}, \tau' \to \tau\right), \\
&\partial\tilde{h}_{\bar{L},\tau'}\left(\tilde{H}'_x, \tau' \to \tau\right) \approx \partial\tilde{h}_{\bar{L},\tau'}\left(\tilde{H}'_{Z_0[\ell_1]^{\rm c},x}, \tau' \to \tau\right).
\end{aligned} \tag{730}$$

By using the above approximated time evolution, we define

$$\begin{aligned}
\tilde{U}_\partial &:= \mathcal{T}\exp\left[-i\int_0^\tau \bar{\Pi}\partial\tilde{h}_{\bar{L},\tau'}\left(\tilde{H}'_{Z_0[\ell_1]^{\rm c},x}, \tau' \to \tau\right)\bar{\Pi} + \bar{\Pi}\partial\tilde{h}_{Z_0,\tau'}\left(\tilde{H}'_{Z_0[\ell_1],x}, \tau' \to \tau\right)\bar{\Pi}d\tau_1\right] \\
&= \mathcal{T}\exp\left[-i\int_0^\tau \bar{\Pi}\partial\tilde{h}_{\bar{L},\tau'}\left(\tilde{H}'_{Z_0[\ell_1]^{\rm c},x}, \tau' \to \tau\right)\bar{\Pi}d\tau_1\right] \otimes \mathcal{T}\exp\left[-i\int_0^\tau \bar{\Pi}\partial\tilde{h}_{Z_0,\tau'}\left(\tilde{H}'_{Z_0[\ell_1],x}, \tau' \to \tau\right)\bar{\Pi}d\tau_1\right].
\end{aligned} \tag{731}$$

Under the above choice of $\tilde{U}_\partial$, we obtain

$$\begin{aligned}
&\tilde{U}_\partial^\dagger\tilde{O}_{Z_0[k]}\left(\tilde{\tilde{H}}'_{Z_0[\ell_1],x}, 0 \to \tau\right)\tilde{U}_\partial = \tilde{U}^\dagger_{\partial,Z_0[\ell_1]}\tilde{O}_{Z_0[k]}\left(\tilde{\tilde{H}}'_{Z_0[\ell_1],x}, 0 \to \tau\right)\tilde{U}_{\partial,Z_0[\ell_1]}, \\
&\tilde{U}_{\partial,Z_0[\ell_1]} := \mathcal{T}\exp\left[-i\int_0^\tau \bar{\Pi}\partial\tilde{h}_{Z_0,\tau'}\left(\tilde{H}'_{Z_0[\ell_1],x}, \tau' \to \tau\right)\bar{\Pi}d\tau_1\right],
\end{aligned} \tag{732}$$

which only depends on the interaction terms of H inside $Z_0[\ell_1]$.

From now, we aim to upper-bound the norm difference between U_∂ and $\tilde{U}_\partial$. We start from the inequality of

$$\begin{aligned}
\|U_\partial - \tilde{U}_\partial\| \le &\int_0^\tau \left\|\partial\tilde{h}_{Z_0,\tau'}\left(\tilde{H}'_x, \tau' \to \tau\right) - \partial\tilde{h}_{Z_0,\tau'}\left(\tilde{H}'_{Z_0[\ell_1],x}, \tau' \to \tau\right)\right\| dx \\
&+ \int_0^\tau \left\|\partial\tilde{h}_{\bar{L},\tau'}\left(\tilde{H}'_x, \tau' \to \tau\right) - \partial\tilde{h}_{\bar{L},\tau'}\left(\tilde{H}'_{Z_0[\ell_1]^{\rm c},x}, \tau' \to \tau\right)\right\| dx.
\end{aligned} \tag{733}$$

Here, we use the inequality of

$$\left\|\mathcal{T}e^{-i\int_0^\tau A_x dx} - \mathcal{T}e^{-i\int_0^\tau B_x dx}\right\| \le \int_0^\tau \|A_x - B_x\| dx \tag{734}$$

for arbitrary time-dependent operators A_x and B_x, which is derived from the decomposition of

$$\mathcal{T}e^{-i\int_0^\tau A_x dx} - \mathcal{T}e^{-i\int_0^\tau B_x dx} = \sum_{m=0}^{M-1}\left(\mathcal{T}e^{-i\int_{\tau_m}^{\tau} A_x dx}\mathcal{T}e^{-i\int_0^{\tau_m} B_x dx} - \mathcal{T}e^{-i\int_{\tau_{m+1}}^{\tau} A_x dx}\mathcal{T}e^{-i\int_0^{\tau_{m+1}} B_x dx}\right)$$
$$= \sum_{m=0}^{M-1}\mathcal{T}e^{-i\int_{\tau_{m+1}}^{\tau} A_x dx}\left(-iA_{\tau_m}d\tau + iB_{\tau_{m+1}}d\tau\right)\mathcal{T}e^{-i\int_0^{\tau_m} B_x dx} + \mathcal{O}(d\tau^2), \tag{735}$$

where $\tau_m = md\tau$ and $\tau_M = \tau$ $(d\tau \to +0)$.

For the first approximation in (733), we can obtain the same approximation error as Eq. (728):

$$\left\|\partial\tilde{h}_{Z_0,\tau'}\left(\tilde{H}'_x, \tau' \to \tau\right) - \partial\tilde{h}_{Z_0,\tau'}\left(\tilde{H}'_{Z_0[\ell_1],x}, \tau' \to \tau\right)\right\|$$
$$\le \left\|\partial\tilde{h}_{Z_0,\tau'}\right\|\Theta(\tau)\bar{N}_{\tilde{L}}\left\{\frac{\tau}{\ell_1}\left[\Theta(Q/\ell_1) + \Theta(\tilde{q}) + \Theta(\bar{q})\right]\right\}^{\Theta(\ell_1)}, \tag{736}$$

Because of the projection $\Pi_{\mathcal{S}(\bar{L},1/2,N_0)}$, we have $\left\|\hat{n}_i\Pi_{\mathcal{S}(\bar{L},1/2,N_0)}\right\| \le N_0$ for $i \in \bar{L}$, and hence

$$\left\|\partial\tilde{h}_{Z_0,\tau'}\right\| \le \sum_{i\in\partial Z_0}\sum_{j\in Z_0^{\mathrm{c}}:d_{i,j}=1}\left\|\Pi_{\mathcal{S}(\bar{L},1/2,N_0)}J_{i,j}\left(b_i b_j^\dagger + \mathrm{h.c.}\right)\Pi_{\mathcal{S}(\bar{L},1/2,N_0)}\right\| \le 4\gamma\bar{J}|Z_0|N_0, \tag{737}$$

where we use the inequality of (666) and $|\partial Z_0| \le |Z_0|$. Therefore, by applying the inequalities (736) and (737) to the first term of the RHS in (733), we obtain

$$\int_0^\tau\left\|\partial\tilde{h}_{Z_0,\tau'}\left(\tilde{H}'_x, \tau' \to \tau\right) - \partial\tilde{h}_{Z_0,\tau'}\left(\tilde{H}'_{Z_0[\ell_1],x}, \tau' \to \tau\right)\right\|dx$$
$$\le 4\gamma\bar{J}|Z_0|N_0 \cdot \Theta(\tau^2)\bar{N}_{\tilde{L}}\left\{\frac{\tau}{\ell_1}\left[\Theta(Q/\ell_1) + \Theta(\tilde{q}) + \Theta(\bar{q})\right]\right\}^{\Theta(\ell_1)}. \tag{738}$$

For the second approximation in (733), we first utilize Lemma 34 by choosing

$$O_X \to \partial\tilde{h}_{\bar{L},\tau'}, \quad H_\tau \to \tilde{H}'_x, \quad L \to Z_0[\ell_1]^{\mathrm{c}} \tag{739}$$

in the inequality (546), which yields

$$\left\|\partial\tilde{h}_{\bar{L},\tau'}\left(\tilde{H}'_x, \tau' \to \tau\right) - \partial\tilde{h}_{\bar{L},\tau'}\left(\tilde{H}'_{Z_0[\ell_1]^{\mathrm{c}},x}, \tau' \to \tau\right)\right\|$$
$$\le \sum_{\mathcal{B}_{i,j}:\mathcal{B}_{i,j}\in\mathcal{S}_{Z_0[\ell_1]}}\int_{\tau'}^{\tau}\left\|\left[\tilde{h}_{\mathcal{B}_{i,j},\tau''}\left(\tilde{H}'_{Z_0[\ell_1]^{\mathrm{c}},x}, \tau'' \to \tau'\right), \partial\tilde{h}_{\bar{L},\tau'}\right]\right\|d\tau''. \tag{740}$$

For the time-evolved operator $\tilde{h}_{\mathcal{B}_{i,j},\tau''}\left(\tilde{H}'_{Z_0[\ell_1]^{\mathrm{c}},x}, \tau'' \to \tau'\right)$, we can apply the same inequality as (736), which gives

$$\left\|\tilde{h}_{\mathcal{B}_{i,j},\tau''}\left(\tilde{H}'_{Z_0[\ell_1]^{\mathrm{c}},x}, \tau'' \to \tau'\right) - \tilde{h}_{\mathcal{B}_{i,j},\tau''}\left(\tilde{H}'_{Z_0[\ell_1+\ell_2]\setminus Z_0[\ell_1],x}, \tau'' \to \tau'\right)\right\|$$
$$\le \left\|\tilde{h}_{\mathcal{B}_{i,j},\tau''}\right\|\Theta(\tau)\bar{N}_{\tilde{L}}\left\{\frac{\tau}{\ell_2}\left[\Theta(Q/\ell_2) + \Theta(\tilde{q}) + \Theta(\bar{q})\right]\right\}^{\Theta(\ell_2)} \quad \text{for} \quad \mathcal{B}_{i,j} \in \mathcal{S}_{Z_0[\ell_1]}, \tag{741}$$

where we choose ℓ_2 such that $\ell_1 + \ell_2 \le \tilde{\ell}_0$. By using the same inequality as (706) with (666), we obtain

$$\sum_{\mathcal{B}_{i,j}:\mathcal{B}_{i,j}\in\mathcal{S}_{Z_0[\ell_1]}}\left\|\tilde{h}_{\mathcal{B}_{i,j},\tau''}\right\| \le \sum_{\mathcal{B}_{i,j}:\mathcal{B}_{i,j}\in\mathcal{S}_{Z_0[\tilde{\ell}_0]}}\left\|\tilde{h}_{\mathcal{B}_{i,j},\tau''}\right\| \le 4\gamma\bar{J}\bar{N}_{\tilde{L}}. \tag{742}$$

From the inequalities (741) and (742), we reduce the inequality (740) to

$$\left\|\partial\tilde{h}_{\bar{L},\tau'}\left(\tilde{H}'_x, \tau' \to \tau\right) - \partial\tilde{h}_{\bar{L},\tau'}\left(\tilde{H}'_{Z_0[\ell_1]^{\mathrm{c}},x}, \tau' \to \tau\right)\right\|$$
$$\le \sum_{\mathcal{B}_{i,j}:\mathcal{B}_{i,j}\in\mathcal{S}_{Z_0[\ell_1]}}\int_{\tau'}^{\tau}\left\|\left[\tilde{h}_{\mathcal{B}_{i,j},\tau''}\left(\tilde{H}'_{Z_0[\ell_1+\ell_2]\setminus Z_0[\ell_1],x}, \tau'' \to \tau'\right), \partial\tilde{h}_{\bar{L},\tau'}\right]\right\|d\tau''$$
$$+ \sum_{\mathcal{B}_{i,j}:\mathcal{B}_{i,j}\in\mathcal{S}_{Z_0[\ell_1]}}\int_{\tau'}^{\tau}\left\|\left[\tilde{h}_{\mathcal{B}_{i,j},\tau''}\left(\tilde{H}'_{Z_0[\ell_1]^{\mathrm{c}},x}, \tau'' \to \tau'\right) - \tilde{h}_{\mathcal{B}_{i,j},\tau''}\left(\tilde{H}'_{Z_0[\ell_1+\ell_2]\setminus Z_0[\ell_1],x}, \tau'' \to \tau'\right), \partial\tilde{h}_{\bar{L},\tau'}\right]\right\|d\tau''$$
$$\le \sum_{\mathcal{B}_{i,j}:\mathcal{B}_{i,j}\in\mathcal{S}_{Z_0[\ell_1]}}\int_{\tau'}^{\tau}2\left\|\partial\tilde{h}_{\bar{L},\tau'}\right\|\cdot\left\|\tilde{h}_{\mathcal{B}_{i,j},\tau''}\right\|\Theta(\tau)\bar{N}_{\tilde{L}}\left\{\frac{\tau}{\ell_2}\left[\Theta(Q/\ell_2) + \Theta(\tilde{q}) + \Theta(\bar{q})\right]\right\}^{\Theta(\ell_2)}d\tau''$$
$$\le 2\tau\left\|\partial\tilde{h}_{\bar{L},\tau'}\right\|\cdot 4\gamma\bar{J}\bar{N}_{\tilde{L}}^2\Theta(\tau)\left\{\frac{\tau}{\ell_2}\left[\Theta(Q/\ell_2) + \Theta(\tilde{q}) + \Theta(\bar{q})\right]\right\}^{\Theta(\ell_2)}, \tag{743}$$

where in the second inequality we use

$$\left\| \left[\tilde{h}_{\mathcal{B}_{i,j},\tau''} \left(\tilde{H}'_{Z_0[\ell_1+\ell_2]\setminus Z_0[\ell_1],x}, \tau'' \to \tau' \right), \partial \tilde{h}_{\bar{L},\tau'} \right] \right\| = \left\| \left[\tilde{h}_{\mathcal{B}_{i,j},\tau''}, \partial \tilde{h}_{\bar{L},\tau'} \right] \right\| = 0, \tag{744}$$

because of $Z_0[\ell_1+\ell_2] \cap \partial \bar{L}[2k] = \emptyset$ for $\ell_1 + \ell_2 \le \tilde{\ell}_0$. Finally, we can derive the upper bound for $\left\| \partial \tilde{h}_{\bar{L},\tau'} \right\|$ as follows:

$$\left\| \partial \tilde{h}_{\bar{L},\tau'} \right\| \le \sum_{i \in \partial \bar{L}} \sum_{j \in \bar{L}^{\mathrm{c}}: d_{i,j}=1} \left\| \Pi_{\mathcal{S}(\bar{L},1/2,N_0)} J_{i,j} \left(b_i b_j^\dagger + \mathrm{h.c.} \right) \Pi_{\mathcal{S}(\bar{L},1/2,N_0)} \right\| \le 4e^{1/2} \gamma \bar{J} |\bar{L}| N_0, \tag{745}$$

where we use (459) and $\left\| \hat{n}_j \Pi_{\mathcal{S}(\bar{L},1/2,N_0)} \right\| \le e^{1/2} N_0$ for $j \in \bar{L}^{\mathrm{c}}$ with $d_{i,j} = 1$. We thus reduce the inequality (743) to

$$\left\| \partial \tilde{h}_{\bar{L},\tau'} \left(\tilde{H}'_x, \tau' \to \tau \right) - \partial \tilde{h}_{\bar{L},\tau'} \left(\tilde{H}'_{Z_0[\ell_1]^{\mathrm{c}},x}, \tau' \to \tau \right) \right\| \le \Theta\left(\tau^2 \bar{N}_{\bar{L}}^2 N_0 |\bar{L}| \right) \left\{ \frac{\tau}{\ell_2} \left[\Theta(Q/\ell_2) + \Theta(\tilde{q}) + \Theta(\bar{q}) \right] \right\}^{\Theta(\ell_2)}, \tag{746}$$

and hence we arrive at the inequality of

$$\begin{aligned} &\int_0^\tau \left\| \partial \tilde{h}_{\bar{L},\tau'} \left(\tilde{H}'_x, \tau' \to \tau \right) - \partial \tilde{h}_{\bar{L},\tau'} \left(\tilde{H}'_{Z_0[\ell_1]^{\mathrm{c}},x}, \tau' \to \tau \right) \right\| dx \\ &\le \Theta\left(\tau^3 \bar{N}_{\bar{L}}^2 N_0 |\bar{L}| \right) \left\{ \frac{\tau}{\ell_2} \left[\Theta(Q/\ell_2) + \Theta(\tilde{q}) + \Theta(\bar{q}) \right] \right\}^{\Theta(\ell_2)}. \end{aligned} \tag{747}$$

By combining the upper bounds of (738) and (747), we can obtain the approximation error $\left\| U_\partial - \tilde{U}_\partial \right\|$ from (733). Therefore, by choosing $\ell_1 = \ell_2 \propto \ell_0$ from $\ell_1 + \ell_2 \le \tilde{\ell}_0 = \ell_0 - (\delta\ell + 2k + 1)$, we obtain

$$\left\| U_\partial - \tilde{U}_\partial \right\| \le \Theta\left(\bar{N}_{\bar{L}}^2 N_0 |\bar{L}| \right) \left\{ \frac{\tau}{\ell_0} \left[\Theta(Q/\ell_0) + \Theta(\tilde{q}) + \Theta(\bar{q}) \right] \right\}^{\Theta(\ell_0)}, \tag{748}$$

where we use $\Theta\left(\tau^3 \bar{N}_{\bar{L}}^2 N_0 |\bar{L}| \right) \le \Theta\left(\bar{N}_{\bar{L}}^2 N_0 |\bar{L}| \right)$ because of $\tau \le 1/(4\gamma \bar{J}) = \mathcal{O}(1)$. By using the above inequality and (728), we finally reduce the inequality (726) to the form of

$$\left\| O_{Z_0}\left(\tilde{H}[\tilde{L}', \mathbf{q}], \tau \right) - \tilde{U}_\partial^\dagger \tilde{O}_{Z_0[k]} \left(\tilde{H}'_{Z_0[\ell_1],x}, 0 \to \tau \right) \tilde{U}_\partial \right\| \le \zeta_{Z_0} \Theta\left(\bar{q}^2 Q^2 N_0 |\bar{L}| \right) \left\{ \frac{\tau}{\ell_0} \left[\Theta(Q/\ell_0) + \Theta(\tilde{q}) + \Theta(\bar{q}) \right] \right\}^{\Theta(\ell_0)}, \tag{749}$$

where we use $\bar{N}_{\bar{L}} \le \bar{q} Q$ from the inequality (586). From the definition of Eq. (731), the approximate operator $\tilde{U}_\partial^\dagger \tilde{O}_{Z_0[k]} \left(\tilde{H}'_{Z_0[\ell_1],x}, 0 \to \tau \right) \tilde{U}_\partial$ is supported on $Z_0[\ell_1] \subseteq Z_0[\ell_0] = L_0$. We thus prove the same statement as Proposition 36 for the effective Hamiltonian.

Thus, by replacing the δ_2 in Proposition 36 with

$$\tilde{\delta}_2 = \zeta_{Z_0} \Theta\left(\bar{q}^2 Q^2 N_0 |\bar{L}| \right) \left\{ \frac{\tau}{\ell_0} \left[\Theta(Q/\ell_0) + \Theta(\tilde{q}) + \Theta(\bar{q}) \right] \right\}^{\Theta(\ell_0)}, \tag{750}$$

the same analyses as in Sec. Supplementary Note 8 B yields the main inequality (714). Note that we have followed the same discussion as in (564) in approximating $O_{Z_0}(\bar{\Pi} H_{L_0} \bar{\Pi}, \tau)$ by $O_{Z_0}(\bar{\Pi} H_{L_0} \bar{\Pi}, \tau)$ [see also Eq. (732)]. This completes the proof. □

Supplementary Note 9. OPTIMAL LIEB-ROBINSON BOUND: HIGH-DIMENSIONAL CASES

Based on Subtheorem 2, we prove the optimal Lieb-Robinson bound with the Lieb-Robinson velocity as $v_{\mathrm{LR}} \propto t^{D-1}$. Here, depending on the spatial dimension (i.e., $D \ge 2$ or $D = 1$), qualitatively different proofs are required. In the following sections Supplementary Note 9 and Supplementary Note 10, we derive the optimal Lieb-Robinson bounds for high dimensions and one dimension, respectively. For high-dimensional cases, we aim to prove the following theorem:

Theorem 3. *Let us consider the same setup as in Theorem 2. Then, under the condition*

$$R' := R - r_0 \ge \Theta\left[t^D \log^{D+2}(q_0 r_0 t) \right], \tag{751}$$

we obtain the Lieb-Robinson bound as

$$\left\| \left[O_{X_0}(t) - O_{X_0}\left(H_{i_0[R]}, t \right) \right] \rho_0 \right\|_1 \le \| O_{X_0} \| \exp\left[-\Theta\left(R'/t^D \right)^{1/\kappa D} + \Theta[\log(q_0 R)] \right]. \tag{752}$$

Remark. From the condition (751), we can conclude that the Lieb-Robinson velocity $v_{\rm LR}$ depends on the time as

$$v_{\rm LR} \approx \tilde{\mathcal{O}}\left(t^{D-1}\right) \quad \text{for} \quad D \geq 2. \tag{753}$$

For $D \geq 2$, this estimation is qualitatively optimal up to a logarithmic correction, while for $D = 1$, we cannot apply the same analyses to prove. In the 1D case, the choices in Eq. (769) give $\tau = \Theta\left(\ell_0/[\mathfrak{q}\log(\ell_0)]\right)$, but we have to make the parameter $\mathfrak{q}$ such that $\mathfrak{q} \geq \ell_0/\log(\ell_0)$ because of $\tau \leq 1/(4\gamma \bar{J})$. Therefore, in 1D, the condition $\tau \leq 1/(4\gamma\bar{J})$ implies $\Delta r \geq \Theta(R'/t)$ ($R' = R - r_0$) from Eq. (771), and hence the condition (777) is satisfied only for $R \geq \Theta(t^2)$, which is no longer better than Theorem 2. This point is contrast to the high dimensional cases ($D \geq 2$), where the choices in Eq. (769) does not influence the condition $\tau \leq 1/(4\gamma\bar{J})$ as long as $\mathfrak{q} \geq \Theta(1)$; that is, unlike the 1D case, we can freely control $\mathfrak{q}$.

In order to improve the Lieb-Robinson velocity to $\tilde{\mathcal{O}}\left(t^0\right)$ in 1D, we need more refined analyses which are summarized in Sec. Supplementary Note 10.

A. Proof of Theorem 3: preparation

Throughout the proof, we consider the Hilbert space which is spanned by the projection $\Pi_{\mathcal{S}(i_0[R],1/2,N_0)}$ with $N_0 = \text{poly}(R)$, which was defined in Eq. (38). We note that i_0 is chosen such that $X_0 \subseteq i_0[r_0]$. From Proposition 32 with $L \to i_0[R]$ and $N \to N_0$, we can achieve the approximation error of

$$\begin{aligned} &\left\| O_{X_0}(H,t)\rho_0 - O_{X_0}(\tilde{H}_{(i_0[R],N_0)},t)\tilde{\Pi}_{(i_0[R],N_0)}\rho_0\tilde{\Pi}_{(i_0[R],N_0)} \right\|_1 \\ &\leq \zeta_0 \Theta\left(t\xi_{t,R}^2 |i_0[R]|\right) e^{-[N_0/(2\xi_{t,R})]^{1/\kappa}/(8\kappa)} \\ &\leq \zeta_0 e^{-R}, \quad \xi_{t,R} := 16 b_0 (2\bar{\ell}_{t,R})^D |X_0|, \end{aligned} \tag{754}$$

where we use (470) in the first inequality, and for the second inequality, we choose N_0 appropriately as

$$N_0 = 64e^2 q_0^3 + \xi_{t,R} R^\kappa \text{polylog}(R) \leq \Theta\left[q_0^3, R^{2D+\kappa}\text{polylog}(R)\right] \leq \Theta\left(q_0^3, R^{2D+1+\kappa}\right). \tag{755}$$

Note that $64e^2q_0^3$ in the above choice comes from the condition (468), and we use $t \leq R$ and $|X_0| \leq |i_0[R]|$ to derive $\xi_{t,R} \leq \Theta[R^{2D}\log^D(q_0 R)]$. Under the above choice of N_0, in the following, we simply denote as

$$\tilde{H}_{i_0[R],N_0} \to H, \quad \tilde{\Pi}_{(i_0[R],N_0)}\rho_0\tilde{\Pi}_{(i_0[R],N_0)} \to \rho_0 \tag{756}$$

without expressing the truncation of the boson numbers.

B. Proof of Theorem 3

In the proof, we consider the length R such that $R \leq e^{\Theta(t)}$. In the asymptotic case as $R > e^{\Theta(t)}$, the obtained upper bound (520) is much stronger than the inequality (752). Throughout the proof, we denote

$$\|O_{X_0}\| = \zeta_0.$$

For the proof, as in Ref. [1], we utilize the connection of short-time unitary evolutions, which has also played critical roles in Refs. [8, 9]. In Ref. [1], the time-independence of the initial state (i.e., $\rho_0(t) = \rho$) has been assumed, but in the present case, we generalize the method to the time-dependent initial state. First, we introduce the time width $\tau \leq 1/(4\gamma\bar{J})$ and decompose the total time t to $\bar{m} := t/\tau$ [$= \mathcal{O}(t)$] pieces:

$$t := \bar{m}\tau. \tag{757}$$

For a fixed R, we define the subset X_m as follows:

$$X_m := i_0[r_0 + m\Delta r], \quad \Delta r = \left\lfloor \frac{R - r_0}{\bar{m}} \right\rfloor = \left\lfloor \frac{R'}{\bar{m}} \right\rfloor \quad (R' := R - r_0), \tag{758}$$

where $X_{\bar{m}} \subseteq i_0[R]$.

We start from the approximation of

$$O_{X_0}(\tau) \xrightarrow{\text{approximate}} O_{X_1}^{(1)} = O_{X_0}(H_{X_1},\tau), \tag{759}$$

whose approximation error is obtained from

$$\left\| \left[O_{X_0}(\tau) - O_{X_1}^{(1)}\right]\rho_0 \right\|_1. \tag{760}$$

In the next step, we connect the two approximations of

$$O_{X_0}(\tau) \xrightarrow{\text{approximate}} O^{(1)}_{X_1}, \quad O^{(1)}_{X_1}(\tau) \xrightarrow{\text{approximate}} O^{(2)}_{X_2} = O^{(1)}_{X_1}(H_{X_2}, \tau). \tag{761}$$

By using the decomposition of

$$\begin{aligned} O_{X_0}(2\tau)\rho_0 &= e^{iH\tau}\left[O_{X_0}(\tau) - O^{(1)}_{X_1}\right] e^{-iH\tau}\rho_0 + e^{iH\tau}O^{(1)}_{X_1}e^{-iH\tau}\rho_0 \\ &= e^{iH\tau}\left[O_{X_0}(\tau) - O^{(1)}_{X_1}\right]\rho_0(\tau)e^{-iH\tau} + \left[O^{(1)}_{X_1}(\tau) - O^{(2)}_{X_2}\right]\rho_0 + O^{(2)}_{X_2}\rho_0, \end{aligned} \tag{762}$$

we obtain the approximation error of

$$\left\|\left[O_{X_0}(2\tau) - O^{(2)}_{X_2}\right]\rho_0\right\|_1 \le \left\|\left[O_{X_0}(\tau) - O^{(1)}_{X_1}\right]\rho_0(\tau)\right\|_1 + \left\|\left[O^{(1)}_{X_1}(\tau) - O^{(2)}_{X_2}\right]\rho_0\right\|_1, \tag{763}$$

where we use the unitary invariance of the trace norm $\|\cdots\|_1$. By repeating the same process, we can obtain

$$\begin{aligned} &\left\|\left[O_{X_0}(\bar{m}\tau) - O^{(\bar{m})}_{X_{\bar{m}}}\right]\rho_0\right\|_1 \le \sum_{j=1}^{\bar{m}} \left\|\left[O^{(j-1)}_{X_{j-1}}(\tau) - O^{(j)}_{X_j}\right]\rho_0(\bar{m}\tau - j\tau)\right\|_1 \\ &\longrightarrow \left\|\left[O_{X_0}(t) - O^{(\bar{m})}_{i_0[R]}\right]\rho_0\right\|_1 \le \sum_{j=1}^{\bar{m}} \left\|\left[O^{(j-1)}_{X_{j-1}}(\tau) - O^{(j)}_{X_j}\right]\rho_0(\bar{m}\tau - j\tau)\right\|_1 \end{aligned} \tag{764}$$

by setting $O^{(\bar{m})}_{i_0[R]} = O^{(\bar{m})}_{X_{\bar{m}}}$ from $X_{\bar{m}} \subseteq i_0[R]$, where each of $\left\{O^{(j)}_{X_j}\right\}_{j=1}^{m}$ is chosen as

$$O^{(j)}_{X_j} = O^{(j-1)}_{X_{j-1}}(H_{X_j}, \tau) \quad \text{for} \quad j \in [\bar{m}]. \tag{765}$$

We notice that each of the operators $\{O^{(j)}_{X_j}\}_{j=1}^m$ satisfies the same inequality as (428), which originally holds for O_{X_0}[*12].

To estimate the approximation errors which appear in (764), we need to combine the following two points:

1. For the time-evolved density matrix $\rho_0(t - j\tau)$, the spectral constraint by Lemma 28 is imposed. Here, the initial low-boson-density condition (3) is loosened in the sense that only the average number of bosons in a ball region with radius $\bar{\ell}_{t,R}$ is small; that is, the boson number on one site can be as large as $\mathcal{O}(\bar{\ell}^D_{t,R}) = \tilde{\mathcal{O}}(t^D)$.
2. For short-time evolution, we have obtained a qualitatively optimal Lieb-Robinson bound as in Subtheorem 2 when the average boson number in a region is constrained as in Eq. (562).

By taking the above two points into account, we can prove the following proposition for short-time evolution:

Proposition 45. *Let us set*

$$\ell_0 \ge \log[\Theta(N_0)] \ge \bar{\ell}_{t,R}, \quad \|O_X\| = \zeta, \tag{767}$$

where N_0 has been chosen as in Eq. (755). Also, we assume that boson creation by the operator O_X ($X \subseteq i_0[R]$) is at most q_0; that is, the inequality (428) holds for O_X. Then, for a short-time evolution of $e^{iH\tau}$, we obtain

$$\begin{aligned} &\left\|\left[O_X(\tau) - O_X(H_{X[\ell]}, \tau)\right]\rho_0(t_1)\right\|_1 \\ &\le \zeta\Theta(N_0 R^{2D} Q^2)\left\{e^{-\Theta\left(Q/\ell_0^D\right)^{1/\kappa}} + e^{-\Theta(\ell_0)} + e^{\Theta(\ell_0)}\left[\Theta(\tau) + \Theta\left(\frac{\tau Q \log(Q)}{\ell_0^2}\right)\right]^{\Theta(\ell_0)}\right\}, \end{aligned} \tag{768}$$

for $\forall t_1 \le t - \tau$ and $X \subset i_0[R - \ell]$, where $\ell = 3\ell_0 + k$ [i.e., $\ell_0 = (\ell - k)/3$].

In the above proposition, we choose

$$Q = \Theta\left(\mathfrak{q}\ell_0^D\right) \quad [\mathfrak{q} \le \Theta(\ell_0)], \quad \tau = \Theta\left(\frac{\ell_0^{-D+2}}{\mathfrak{q}\log(\ell_0)}\right), \quad \Delta r = \ell = 3\ell_0 + k. \tag{769}$$

[*12] For an arbitrary operator O_{X_0} such that the inequality (428) holds, the time evolved operator $O_{X_0}(H_{X_0[r]}, t)$ also satisfies the same inequality by replacing $X_0 \to X_0[r]$ in (428) because of

$$\Pi_{X_0[r], > m+q_0} O_{X_0}(H_{X_0[r]}, t)\Pi_{X_0[r], m} = e^{iH_{X_0[r]}t}\Pi_{X_0[r], > m+q_0} O_{X_0}\Pi_{X_0[r], m}e^{-iH_{X_0[r]}t}, \tag{766}$$

where we use $[H_{X_0[r]}, \hat{n}_{X_0[r]}] = 0$.

We set the parameter $\mathfrak{q}$ appropriately afterward [see (773) below]. From the above choices, we can make the RHS of the inequality (768)

$$\Theta(N_0R^{2D}Q^2)\left\{e^{-\Theta\left(Q/\ell_0^D\right)^{1/\kappa}}+e^{-\Theta(\ell_0)}+e^{\Theta(\ell_0)}\left[\Theta(\tau)+\Theta\left(\frac{\tau Q\log(Q)}{\ell_0^2}\right)\right]^{\Theta(\ell_0)}\right\}$$
$$=\Theta\left(N_0R^{2D}Q^2\right)\left(e^{-\Theta(\mathfrak{q})^{1/\kappa}}+e^{-\Theta(\ell_0)}\right)\le\Theta\left(N_0R^{4D+2}\right)e^{-\Theta(\mathfrak{q})^{1/\kappa}}, \tag{770}$$

where in the last inequality, we use $Q\le\Theta\left(\ell_0^{D+1}\right)\le\Theta\left(R^{D+1}\right)$ and $e^{-\Theta(\mathfrak{q})^{1/\kappa}}\ge e^{-\Theta(\ell_0)}$ $(\kappa\ge1)$ from $\mathfrak{q}\le\Theta(\ell_0)$.

We then consider how to choose the parameter $\mathfrak{q}$. Here, from Eq. (758), we have $\Delta r=\left\lfloor\frac{R'}{\bar{m}}\right\rfloor=\left\lfloor\frac{R'}{(t/\tau)}\right\rfloor$, and hence the above choice (769) also yields

$$\Delta r=\left\lfloor\frac{R'}{(t/\tau)}\right\rfloor=\frac{R'}{t}\Theta\left(\frac{\ell_0^{-D+2}}{\mathfrak{q}\log(\ell_0)}\right)=\Theta\left(\frac{R'}{t}\cdot\frac{(\Delta r)^{-D+2}}{\mathfrak{q}\log(\Delta r)}\right) \tag{771}$$
$$\longrightarrow(\Delta r)^{D-1}\log(\Delta r)=\Theta\left(\frac{R'}{\mathfrak{q}t}\right)$$
$$\longrightarrow\Delta r=\Theta\left[\frac{R'}{\mathfrak{q}t\log(R'/t)}\right]^{1/(D-1)}=\frac{t}{\log^{1/(D-1)}(R'/t)}\Theta\left(\frac{R'}{\mathfrak{q}t^D}\right)^{1/(D-1)}$$
$$\longrightarrow\ell_0=\frac{t}{\log^{1/(D-1)}(R'/t)}\Theta\left(\frac{R'}{\mathfrak{q}t^D}\right)^{1/(D-1)}, \tag{772}$$

where we use $\ell_0=(\Delta r-k)/3=\Theta(\Delta r)$ in the last equation. Because of $\mathfrak{q}\le\Theta(\ell_0)$, by choosing $\mathfrak{q}$ as

$$\mathfrak{q}=\Theta\left(R'/t^D\right)^{1/D}, \tag{773}$$

we reduces Eq. (772) to

$$\ell_0=\frac{t}{\log^{1/(D-1)}(R'/t)}\Theta\left(R'/t^D\right)^{1/D}\propto\frac{t}{\log^{1/(D-1)}(R'/t)}\mathfrak{q}. \tag{774}$$

Now, the condition $\mathfrak{q}\le\Theta(\ell_0)$ is ensured from the assumption of $R'\le R\le e^{\Theta(t)}$. We thus adopt the choice of Eq. (773) in the following.

Under the choices of Eqs. (769) and (773), by applying the inequalities (768) and (770) to (764), we obtain

$$\left\|\left[O_{X_0}(t)-O_{X_{\bar{m}}}^{(\bar{m})}\right]\rho_0\right\|_1\le\bar{m}\zeta_0\Theta\left(N_0R^{4D+2}\right)\exp\left[-\Theta\left(R'/t^D\right)^{1/\kappa D}\right]$$
$$\le\zeta_0\Theta\left(N_0R^{4D+3}\right)\exp\left[-\Theta\left(R'/t^D\right)^{1/\kappa D}\right], \tag{775}$$

where in the second inequality we use $\bar{m}=\lfloor R/\Delta r\rfloor\le R$ from Eq. (758). From the choice of Eq. (755), we reduce $\Theta\left(N_0R^{4D+3}\right)$ to

$$\Theta\left(N_0R^{4D+3}\right)\le\Theta\left(q_0^3R^{4D+3+2D+1+\kappa}\right)=\Theta\left(q_0^3R^{6D+4+\kappa}\right). \tag{776}$$

By applying the above bound to the inequality (775), we obtain the desired upper bound for the approximation of $O_{X_0}(t)$. We prove the same upper bound for $\left\|\left[O_{X_0}(H_{i_0[R]},t)-O_{X_{\bar{m}}}^{(\bar{m})}\right]\rho_0\right\|_1$ under the choices as in Eq. (765), where $H_{i_0[R]}$ is the subset Hamiltonian on $i_0[R]$. We thus prove the main inequality (752) by letting $R'=R-r_0$.

Finally, we consider the condition $\ell_0\ge\bar{\ell}_{t,R}=\Theta[t\log(q_0R)]$ in Eq. (767) is now given by

$$\frac{t}{\log^{1/(D-1)}(R'/t)}\Theta\left(R'/t^D\right)^{1/D}\ge\Theta(\bar{\ell}_{t,R})\longrightarrow\left(R'/t^D\right)^{1/D}\ge\Theta(\log^{1+1/(D-1)}(q_0R))$$
$$\longrightarrow R-r_0\ge\Theta\left[t^D\log^{D^2/(D-1)}(q_0r_0t)\right]\longrightarrow R-r_0\ge\Theta\left[t^D\log^{D+2}(q_0r_0t)\right], \tag{777}$$

where we use $D^2/(D-1)\le D+2$ for $D\ge2$. This completes the proof. □

C. Proof of Proposition 45

For the proof, we utilize Subtheorem 2 appropriately to estimate the approximation error of

$$\left\|\left[O_X(\tau)-O_X(H_{X[\ell]},\tau)\right]\rho_0(t_1)\right\|_1 \tag{778}$$

for $t_1 \le t-\tau$. However, we cannot directly apply the subtheorem because we encounter the following difficulty. In high dimensional cases, when the radius ℓ becomes large, the subset size $|L_0^*|$ in Eq. (561) is roughly given by $|L_0^*| = |X[\ell] \setminus X| = \Theta\left[(\mathrm{diam}(X)+\ell)^D\right]$, and hence the number of bosons in the region L_0^*, denoted by Q, is proportional to $|L_0^*|$, i.e., $Q \propto \Theta\left[(\mathrm{diam}(X)+\ell)^D\right]$. Therefore, in applying the inequality (563) to an operator O_X supported on a large subset X such that $\mathrm{diam}(X) \gtrsim \ell$, we have to consider a significantly short time such that

$$\frac{\tau Q \log(Q)}{\ell^2} \propto \tau \left(\frac{\mathrm{diam}(X)}{\ell}\right)^2 \mathrm{diam}(X)^{D-2} \log[\mathrm{diam}(X)] \lesssim \frac{1}{e}, \tag{779}$$

which is necessary to make the inequality (563) meaningful. Unfortunately, under the above condition, we can no longer derive the optimal Lieb-Robinson bound. To avoid the constraint (779), before applying the subtheorem to the derivation of the Lieb-Robinson bound, we first reduce the problem to the time evolution of local operators (see Fig. 23 below).

For this purpose, we first define the projection $\bar{\Pi}_{Y,r,\le Q}$ as follows:

$$\bar{\Pi}_{Y,r,\le Q} := \prod_{i\in Y} \Pi_{i[r],\le Q}. \tag{780}$$

From the projection, for any site $i \in Y$, the number of bosons in the ball region $i[r]$ is smaller than or equal to Q. We also define the following subsets (see Fig. 23):

$$X_1 := X[2\ell_0], \quad X_2 := X[2\ell_0+k], \quad X_3 := X[3\ell_0+k], \quad \tilde{X} := X_2 \setminus X_1 = X[2\ell_0+k] \setminus X[2\ell_0], \tag{781}$$

where ℓ_0 has been defined in the main statement, i.e., $\ell_0 = (\ell-k)/3$. We then adopt the projection of

$$\bar{\Pi}^* := \bar{\Pi}_{\tilde{X},\ell_0,\le Q} = \prod_{i\in \tilde{X}} \Pi_{i[\ell_0],\le Q}. \tag{782}$$

We next consider the decomposition of

$$H = H_1 + H_2 + \partial H_0 \tag{783}$$

with

$$\begin{aligned} &H_1 = H_{0,X_1} + V_{X_2}, \quad H_2 = H_{0,X_2^{\mathrm{c}}} + V_{X_2^{\mathrm{c}}} + \sum_{Z\in \mathcal{S}_{X_2}} v_Z, \\ &\partial H_0 = H - H_1 - H_2 = H_{0,X_2\setminus X_1} + \partial h_{X_1} + \partial h_{X_2} = \sum_{\substack{\langle i,j\rangle \\ \{i,j\}\cap \tilde{X} \neq \emptyset}} J_{i,j}(b_i b_j^\dagger + \mathrm{h.c.}), \end{aligned} \tag{784}$$

where we use the notations of Eq. (28), and $\mathcal{S}_{X_2}$ has been defined in Eq. (547), i.e., $\mathcal{S}_{X_2} := \{Z \subseteq \Lambda | Z\cap X_2 \neq \emptyset, Z \cap X_2^{\mathrm{c}} \neq \emptyset\}$. Because the width of the region $X_2 \setminus X_1$ is equal to k (see Fig. 23), we ensure that the support of v_Z $(Z \in \mathcal{S}_{X_2})$ has no overlap with X_1, and hence

$$[H_1, H_2] = \sum_{Z\in \mathcal{S}_{X_2}} [H_{0,X_1}, v_Z] = 0. \tag{785}$$

By using the above notations, we consider

$$e^{-iH\tau} = e^{-i(H_1+H_2)\tau} \mathcal{T} e^{-i\int_0^\tau \partial H_{0,x} dx}, \tag{786}$$

with

$$\partial H_{0,x} := \partial H_0\left(H_1+H_2, x\right) = e^{i(H_1+H_2)x} \partial H_0 e^{-i(H_1+H_2)x}. \tag{787}$$

Under the above setup, we will take the following strategy to prove Proposition 45. From Eq. (786), we reduce the target time evolution $O_X(\tau)$ to

$$\begin{aligned} O_X(\tau) &= \left(\mathcal{T} e^{-i\int_0^\tau \partial H_{0,x} dx}\right)^\dagger O_X(H_1+H_2,\tau) \mathcal{T} e^{-i\int_0^\tau \partial H_{0,x} dx} \\ &=: O_{X_2,\tau}(\partial H_{0,x}, 0 \to \tau), \end{aligned} \tag{788}$$

where we denote $O_X(H_1+H_2,\tau) = O_X(H_1,\tau) =: O_{X_2,\tau}$. Note that from Eq. (785) and $[O_X, H_2] = 0$, we have

$$e^{i(H_1+H_2)\tau} O_X e^{-i(H_1+H_2)\tau} = e^{iH_1\tau} e^{iH_2\tau} O_X e^{-iH_2\tau} e^{-iH_1\tau} = e^{iH_1\tau} O_X e^{-iH_1\tau}.$$

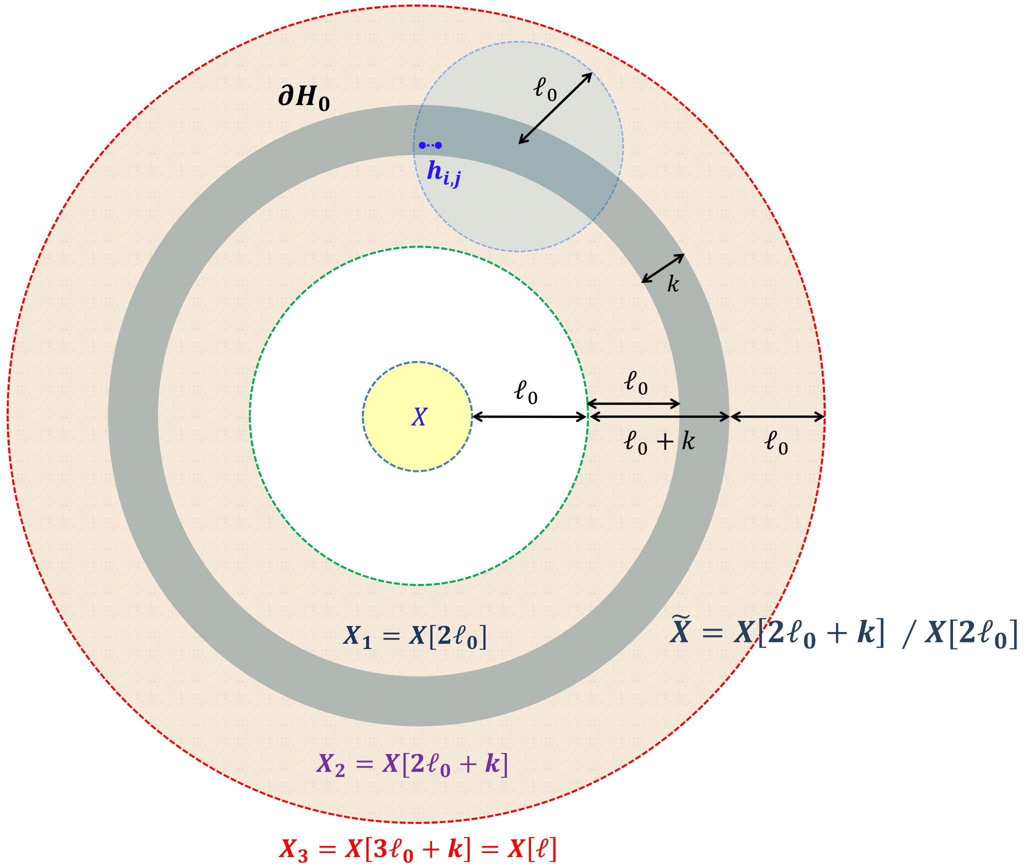

Supplementary Figure 23. Schematic picture of the setup for the proof. We first define the regions X_1, X_2 and X_3 as in Eq. (781). The shaded region $\tilde{X}$ is defined as $X_2 \setminus X_1$. The Hamiltonian is decomposed to $H_1 + H_2 + \partial H_0$ [see Eq. (784)]. Roughly speaking, the Hamiltonians H_1 and H_2 acts on the region X_1 and X_2^c, respectively, and the Hamiltonian ∂H_0 describes the interactions in the region $\tilde{X}$. By these choices, the Hamiltonians H_1 and H_2 are decoupled in the sense that $[H_1 + H_2] = 0$ as in Eq. (785). Then, the time evolution e^{-iHt} is decomposed to $e^{-i(H_1+H_2)\tau}$ and $\mathcal{T}e^{-i\int_0^\tau \partial H_{0,x} dx}$ as in Eq. (786), where $\partial H_{0,x} = \partial H_0 \left(H_1 + H_2, x\right)$. The time evolution $e^{-i(H_1+H_2)\tau} = e^{-iH_1\tau}e^{-iH_2\tau}$ keeps the operator O_X in the region X_2, whereas the time evolution $\mathcal{T}e^{-i\int_0^\tau \partial H_{0,x} dx}$ may induce the operator spreading outside the region X_3. In order to ensure the quasi-locality of the operator $\partial H_{0,x} = \partial H_0(H_1 + H_2, x)$, we first approximate $\partial H_{0,x}$ by the effective Hamiltonian $\bar{\Pi}^* \partial H_{0,x} \bar{\Pi}^*$ (Lemma 46), where $\bar{\Pi}^*$ is defined in Eq. (782). Afterward, by using Subtheorem 2, we approximate the effective Hamiltonian $\bar{\Pi}^* \partial H_{0,x} \bar{\Pi}^*$ onto the region X_3 [Corollary 47 and the inequality (813)].

We then aim to approximate the time evolution (788) by using the effective Hamiltonian as

$$\partial \tilde{H}_{0,x}^* := \bar{\Pi}^* \partial H_{0,x} \bar{\Pi}^* = \bar{\Pi}^* e^{i(H_1+H_2)x} \partial H_0 e^{-i(H_1+H_2)x} \bar{\Pi}^*, \tag{789}$$

where the second equation results from Eq. (787). As a first task, we need to estimate the approximation error of

$$O_{X_2,\tau}(\partial H_{0,x}, 0 \to \tau) \approx O_{X_2,\tau}(\bar{\Pi}^* \partial H_{0,x} \bar{\Pi}^*, 0 \to \tau), \tag{790}$$

and then our second task is to approximate $\bar{\Pi}^* \partial H_{0,x} \bar{\Pi}^*$ onto X_3 $(= X[\ell])$. Because $\partial H_{0,x}$ is constructed from $b_i b_j^\dagger$ with $\{i,j\} \cap \tilde{X} \neq \emptyset$, we need to obtain the approximation of

$$\bar{\Pi}^* e^{i(H_1+H_2)x} (b_i b_j^\dagger) e^{-i(H_1+H_2)x} \bar{\Pi}^* \approx \bar{\Pi}^* e^{i\left(H_{1,i[\ell_0]} + H_{2,i[\ell_0]}\right)x} (b_i b_j^\dagger) e^{-i\left(H_{1,i[\ell_0]} + H_{2,i[\ell_0]}\right)x} \bar{\Pi}^*. \tag{791}$$

Because of $\{i,j\} \cap \tilde{X} \neq \emptyset$, the subset Hamiltonian $H_{1,i[\ell_0]} + H_{2,i[\ell_0]}$ is still supported on X_3. To the above approximation, we can utilize Subtheorem 2. Combining the approximations (790) and (791) leads to the main inequality (768).

First of all, for the approximation (790), we prove the following lemma:

Lemma 46. *For the time-evolved quantum state $\rho_0(t_1)$ with an arbitrary t_1 $(\leq t - \tau)$, we upper-bound the norm of*

$$\begin{aligned} &\left\| \left[O_X(\tau) - O_{X_2,\tau}(\partial \tilde{H}_{0,x}^*, 0 \to \tau) \right] \rho_0(t_1) \right\|_1 \\ &= \left\| \left[O_{X_2,\tau}(\partial H_{0,x}, 0 \to \tau) - O_{X_2,\tau}(\partial \tilde{H}_{0,x}^*, 0 \to \tau) \right] \rho_0(t_1) \right\|_1 \leq \delta_{\ell_0,Q}^{(1)}, \end{aligned} \tag{792}$$

where $\delta^{(1)}_{\ell_0,Q}$ *is defined as follows:*

$$\delta^{(1)}_{\ell_0,Q} := 4\gamma R^D(1+2\tau\gamma^2\bar{J}N_0R^D)\zeta e^{-\left[Q/(4b_0\ell_0^D)\right]^{1/\kappa}/2} = \Theta(N_0R^{2D})e^{-\Theta\left(Q/\ell_0^D\right)^{1/\kappa}}. \tag{793}$$

1. Proof of Lemma 46

For the proof, we utilize Corollary 27. We here denote

$$U_{\tau'} = U_{\partial H_{0,x},0\to\tau'}. \tag{794}$$

From the inequality (414), in estimating the norm error in (792), we have to upper-bound

$$\left\| \bar{\Pi}^{*\mathrm{c}} U_{\tau_1} U_\tau^\dagger O_{X_2,\tau} U_\tau \sqrt{\rho_0(t_1)} \right\|_F, \tag{795}$$

and

$$\left\| \bar{\Pi}^{*} \partial H_{0,\tau-\tau_1} \bar{\Pi}^{*\mathrm{c}} U_{\tau_1} U_\tau^\dagger O_{X_2,\tau} U_\tau \sqrt{\rho_0(t_1)} \right\|_F. \tag{796}$$

Note that all the terms in LHS of (414) reduce to either of the above forms by letting $\tau_1 \to (0 \text{ or } \tau)$ and $O_{X_2} \to (\hat{1} \text{ or } O_{X_2})$

First, from the definition (787), we aim to upper-bound

$$\left\| \bar{\Pi}^{*} \partial H_{0,\tau-\tau_1} \bar{\Pi}^{*\mathrm{c}} \right\| \le \sum_{\substack{\langle i,j\rangle \\ \{i,j\}\cap\tilde{X}\neq\emptyset}} \bar{J} \left\| \bar{\Pi}^{*}\left(b_i b_j^\dagger + \text{h.c.}\right)\right\| \le \sum_{\substack{\langle i,j\rangle \\ \{i,j\}\cap\tilde{X}\neq\emptyset}} 2\bar{J}\left\|\bar{\Pi}^{*}\left(\hat{n}_i+\hat{n}_j\right)\right\|, \tag{797}$$

where we use (459) in the last inequality. Because the Hilbert space is restricted by $\tilde{\Pi}_{(i_0[R],N_0)}$ as in (756), we can upper-bound as $\|\hat{n}_i\| \le N_0$ for $i \in i_0[R]$, which yields

$$\left\| \bar{\Pi}^{*} \partial H_{0,\tau-\tau_1} \bar{\Pi}^{*\mathrm{c}} \right\| \le \sum_{\substack{\langle i,j\rangle \\ \{i,j\}\cap\tilde{X}\neq\emptyset}} 4\bar{J}N_0 \le 4\gamma\bar{J}N_0|X_2\setminus X_1| \le 4\gamma\bar{J}N_0|X_2|, \tag{798}$$

where we use $\tilde{X} := X_2 \setminus X_1$ as in Eq. (781). We hence upper-bound the norm (796) as

$$\left\| \bar{\Pi}^{*} \partial H_{0,\tau-\tau_1} \bar{\Pi}^{*\mathrm{c}} U_{\tau_1} U_\tau^\dagger O_{X_2,\tau} U_\tau \sqrt{\rho_0(t_1)} \right\|_F \le 4\gamma\bar{J}N_0|X_2| \left\| \bar{\Pi}^{*\mathrm{c}} U_{\tau_1} U_\tau^\dagger O_{X_2,\tau} U_\tau \sqrt{\rho_0(t_1)} \right\|_F. \tag{799}$$

Thus, we only have to estimate the norm (795).

We first reduce the norm (795) to

$$\begin{aligned}\left\| \bar{\Pi}^{*\mathrm{c}} U_{\tau_1} U_\tau^\dagger O_{X_2,\tau} U_\tau \sqrt{\rho_0(t_1)} \right\|_F &= \left\| \bar{\Pi}^{*\mathrm{c}} e^{i(H_1+H_2)\tau_1} e^{-iH\tau_1} e^{iH\tau} e^{-i(H_1+H_2)\tau} O_{X_2,\tau} e^{i(H_1+H_2)\tau} e^{-iH\tau} \sqrt{\rho_0(t_1)} \right\|_F \\ &= \left\| \bar{\Pi}^{*\mathrm{c}} e^{i(H_1+H_2)\tau_1} e^{iH(\tau-\tau_1)} O_X e^{-iH\tau} \sqrt{\rho_0(t_1)} \right\|_F,\end{aligned} \tag{800}$$

where in the first equation, we use from Eqs. (786) and (794)

$$U_{\tau'} = \mathcal{T} e^{-i\int_0^{\tau'} \partial H_0(H_1+H_2,x)dx} = e^{i(H_1+H_2)\tau'} e^{-iH\tau'}, \quad H = H_1+H_2+\partial H_0, \tag{801}$$

and the second equation is obtained from the definition of $O_X(H_1+H_2,\tau) = O_X(H_1,\tau) =: O_{X_2,\tau}$. By applying the definition (782) to Eq. (800), we have

$$\begin{aligned}&\left\| \bar{\Pi}^{*\mathrm{c}} e^{i(H_1+H_2)\tau_1} e^{iH(\tau-\tau_1)} O_X e^{-iH\tau} \sqrt{\rho_0(t_1)} \right\|_F \\ &\le \sum_{i\in\tilde{X}} \left\| \Pi_{i[\ell_0],>Q} e^{i(H_1+H_2)\tau_1} e^{iH(\tau-\tau_1)} O_X e^{-iH\tau} \sqrt{\rho_0(t_1)} \right\|_F.\end{aligned} \tag{802}$$

For the estimation of the norm of

$$\left\| \Pi_{i[\ell_0],>Q} e^{i(H_1+H_2)\tau_1} e^{iH(\tau-\tau_1)} O_X e^{-iH\tau} \sqrt{\rho_0(t_1)} \right\|_F = \left\| \Pi_{i[\ell_0],>Q} e^{i(H_1+H_2)\tau_1} e^{iH(\tau-\tau_1)} O_X e^{-iH\tau} e^{-iHt_1} \sqrt{\rho_0} \right\|_F,$$

we can apply the inequality (434) in Lemma 28 from $t_1+\tau \le t$. In the lemma, the boson creation by the operator O_{X_0} is assumed to be smaller than or equal to q_0 (see (49) for the formulation), which also holds for O_X from the

assumption of the main statement in Proposition 45. Moreover, from the assumption of $\ell_0 \geq \bar{\ell}_{t,R}$ and $d_{i,X} \geq 2\ell_0$ for $i \in \tilde{X}$ $(= X[2\ell_0 + k] \setminus X[2\ell_0])$, we have $i[\ell_0] \subset X[\ell_0]^{\rm c} \subseteq X[\bar{\ell}_{t,R}]^{\rm c}$. Therefore, by using the inequality (434) with $p = 0$ obtain

$$\left\| \Pi_{i[\ell_0],>Q} e^{i(H_1+H_2)\tau_1} e^{iH(\tau-\tau_1)} O_X e^{-iH\tau} \sqrt{\rho_0(t_1)} \right\|_F^2 \leq \zeta^2 e^{-[Q/(4b_0\ell_0^D)]^{1/\kappa}}. \tag{803}$$

By applying the inequality (803) to (800) and (802), we obtain

$$\left\| \bar{\Pi}^{*{\rm c}} U_{\tau_1} U_\tau^\dagger O_{X_2,\tau} U_\tau \sqrt{\rho_0(t_1)} \right\|_F = \left\| \bar{\Pi}^{*{\rm c}} e^{i(H_1+H_2)\tau_1} e^{iH(\tau-\tau_1)} O_X e^{-iH\tau} \sqrt{\rho_0(t_1)} \right\|_F \leq |\tilde{X}| \zeta e^{-[Q/(4b_0\ell_0^D)]^{1/\kappa}/2}, \tag{804}$$

which also yields from (799)

$$\left\| \bar{\Pi}^* \partial H_{0,\tau_1} \bar{\Pi}^{*{\rm c}} U_{\tau-\tau_1}^\dagger O_{X_2,\tau} U_\tau \sqrt{\rho_0(t_1)} \right\|_F \leq 4\gamma \bar{J} N_0 |X_2| \cdot |\tilde{X}| \zeta e^{-[Q/(4b_0\ell_0^D)]^{1/\kappa}/2}. \tag{805}$$

By combining the above two inequalities with Corollary 27, we obtain

$$\begin{aligned} &\left\| \left[O_{X_2,\tau}(\partial H_{0,x}, 0 \to \tau) - O_{X_2,\tau}(\partial \tilde{H}^*_{0,x}, 0 \to \tau) \right] \rho_0(t_1) \right\|_1 \\ \leq& 4|\tilde{X}|\zeta e^{-[Q/(4b_0\ell_0^D)]^{1/\kappa}/2} + 8\gamma \bar{J} N_0 |X_2| \tau \cdot |\tilde{X}| \zeta e^{-[Q/(4b_0\ell_0^D)]^{1/\kappa}/2} \\ \leq& 4\gamma R^D (1 + 2\tau\gamma^2 \bar{J} N_0 R^D) \zeta e^{-[Q/(4b_0\ell_0^D)]^{1/\kappa}/2}, \end{aligned} \tag{806}$$

where we use $\tilde{X}, X_2 \subset i_0[R]$, which yields $|\tilde{X}|, |X_2| \leq |i_0[R]| \leq \gamma R^D$. We thus prove the main inequality (792). This completes the proof. □

———————————————————————————[**End of Proof of Lemma 46**]

In the following, we aim to estimate the error to approximate $\partial \tilde{H}^*_{0,x}$ onto the subset X_3 $(= X[3\ell_0 + k])$. We here denote

$$h_{i,j} = J_{i,j}(b_i b_j^\dagger + {\rm h.c.}), \quad \tilde{h}^*_{i,j}(H_1 + H_2, x) = \bar{\Pi}^* h_{i,j}(H_1 + H_2, x) \bar{\Pi}^*. \tag{807}$$

We then consider each of the interaction terms $\tilde{h}^*_{i,j}(H_1 + H_2, x)$ that is included in $\partial \tilde{H}^*_{0,x}$, i.e.,

$$\partial \tilde{H}^*_{0,x} = \bar{\Pi}^* e^{i(H_1+H_2)x} \partial H_0 e^{-i(H_1+H_2)x} \bar{\Pi}^* = \sum_{\langle i,j \rangle : \{i,j\} \cap \tilde{X} \neq \emptyset} \tilde{h}^*_{i,j}(H_1 + H_2, x), \tag{808}$$

where we use Eqs. (789) and (784). For each of $\tilde{h}^*_{i,j}(H_1 + H_2, x)$, we aim to estimate the approximation error of

$$\bar{\Pi}^* h_{i,j}(H_1 + H_2, x) \bar{\Pi}^* \approx \bar{\Pi}^* h_{i,j \to X_3, x} \bar{\Pi}^* = \tilde{h}^*_{i,j \to X_3, x}, \tag{809}$$

where the operator $\tilde{h}_{i,j \to X_3, x}$ is an appropriate operator supported on X_3. Here, from the definitions in Eq. (781), we can ensure (see also Fig. 23)

$$\{i,j\}[\ell_0 - 1] \subset X_3 \quad \text{for} \quad \{i,j\} \cap \tilde{X} \neq \emptyset \quad (d_{i,j} = 1).$$

By applying Subtheorem 2, we immediately obtain the following corollary:

Corollary 47. *Under the constraints on the Hilbert space as in (756) and the projection (782), there exists an appropriate operator $\tilde{h}_{i,j \to X_3, x}$ such that*

$$\left\| \left[h_{i,j}(H_1 + H_2, x) - \tilde{h}^*_{i,j \to X_3, x} \right] \bar{\Pi}^* \Pi_{\mathcal{S}(i_0[R], 1/2, N_0)} \right\| \leq \|h_{i,j}\| \delta^{(2)}_{\ell_0,Q} \quad \textit{for} \quad x \leq \tau, \tag{810}$$

where $\ell_0 \geq \log[\Theta(N_0)]$ and $\delta_{\ell_0,Q}$ is defined as

$$\delta^{(2)}_{\ell_0,Q} := \Theta(Q^2) \left\{ e^{-\Theta(\ell_0)} + e^{\Theta(\ell_0)} \left[\Theta(\tau) + \Theta\left(\frac{\tau Q \log(Q)}{\ell_0^2} \right) \right]^{\Theta(\ell_0)} \right\}. \tag{811}$$

The statement is proved by letting $O_{Z_0} \to h_{i,j}$ $(r_1 = 1,\ q_{Z_0} = 1)$ in Subtheorem 2.

Therefore, by using the notation of

$$\partial \tilde{H}^*_{0,X_3,x} = \sum_{\substack{\langle i,j \rangle \\ \{i,j\} \cap \tilde{X} \neq \emptyset}} \tilde{h}^*_{i,j \to X_3, x}, \tag{812}$$

we obtain

$$
\begin{aligned}
&\left\|\mathcal{T}e^{-i\int_0^\tau \partial\tilde{H}^*_{0,x}dx} - \mathcal{T}e^{-i\int_0^\tau \partial\tilde{H}^*_{0,X_3,x}dx}\right\| \le \int_0^\tau \left\|\partial\tilde{H}^*_{0,x} - \partial\tilde{H}^*_{0,X_3,x}\right\| dx \\
&\le \sum_{\substack{\langle i,j\rangle \\ \{i,j\}\cap\tilde{X}\neq\emptyset}} \int_0^\tau \left\|\tilde{h}^*_{i,j}\left(H_1+H_2,x\right) - \tilde{h}^*_{i,j\to X_3,x}\right\| dx \\
&= \sum_{\substack{\langle i,j\rangle \\ \{i,j\}\cap\tilde{X}\neq\emptyset}} \int_0^\tau \left\|\bar{\Pi}^*\left[h_{i,j}\left(H_1+H_2,x\right) - h_{i,j\to X_3,x}\right]\bar{\Pi}^*\Pi_{\mathcal{S}(i_0[R],1/2,N_0)}\right\| dx \\
&\le \sum_{\substack{\langle i,j\rangle \\ \{i,j\}\cap\tilde{X}\neq\emptyset}} \tau\left\|h_{i,j}\right\|\delta^{(2)}_{\ell_0,Q} \le 4\tau\gamma\bar{J}N_0|X_2|\delta^{(2)}_{\ell_0,Q}, \qquad (813)
\end{aligned}
$$

where the equation is a consequence from the constraints of the Hilbert space on $\Pi_{\mathcal{S}(i_0[R],1/2,N_0)}$ as in (756), and we use the same analysis as (798) in the last inequality.

By using the above inequality, we obtain the approximation of

$$
O_{X_2,\tau}(\partial\tilde{H}^*_{0,x}, 0\to\tau) \approx O_{X_2,\tau}(\partial\tilde{H}^*_{0,X_3,x}, 0\to\tau). \qquad (814)
$$

From the inequality of

$$
\left\|U^\dagger O U - \tilde{U}^\dagger O\tilde{U}\right\| = \left\|U^\dagger O U - U^\dagger O\tilde{U} + U^\dagger O\tilde{U} - \tilde{U}^\dagger O\tilde{U}\right\| \le 2\left\|O\right\|\cdot\left\|U-\tilde{U}\right\|, \qquad (815)
$$

we obtain the approximation error of (814) by using the inequality (813) as

$$
\begin{aligned}
\left\|O_{X_2,\tau}(\partial\tilde{H}^*_{0,x}, 0\to\tau) - O_{X_2,\tau}(\partial\tilde{H}^*_{0,X_3,x}, 0\to\tau)\right\| &\le 2\left\|O_X\right\|\cdot\left\|\mathcal{T}e^{-i\int_0^\tau \partial\tilde{H}^*_{0,x}dx} - \mathcal{T}e^{-i\int_0^\tau \partial\tilde{H}^*_{0,X_3,x}dx}\right\| \\
&\le 8\zeta\tau\gamma\bar{J}N_0|X_2|\delta^{(2)}_{\ell_0,Q} \le 8\zeta\tau\gamma^2\bar{J}N_0R^D\delta^{(2)}_{\ell_0,Q}, \qquad (816)
\end{aligned}
$$

where we use $|X_2| \le i_0[R] \le \gamma R^D$.

By combining the inequality (792) in Lemma 46 with (816), we finally arrive at the inequality of

$$
\begin{aligned}
&\left\|\left[O_X(\tau) - O_{X_2,\tau}(\partial\tilde{H}^*_{0,X_3,x}, 0\to\tau)\right]\rho_0(t_1)\right\|_1 \\
&\le \delta^{(1)}_{\ell_0,Q} + 8\zeta\tau\gamma^2\bar{J}N_0R^D\delta^{(2)}_{\ell_0,Q} \\
&= \Theta(N_0R^{2D})e^{-\Theta\left(Q/\ell_0^D\right)^{1/\kappa}} + \zeta\Theta(N_0R^D)\cdot\Theta(Q^2)\left\{e^{-\Theta(\ell_0)} + e^{\Theta(\ell_0)}\left[\Theta(\tau) + \Theta\left(\frac{\tau Q\log(Q)}{\ell_0^2}\right)\right]^{\Theta(\ell_0)}\right\} \\
&= \zeta\Theta(N_0R^{2D}Q^2)\left\{e^{-\Theta\left(Q/\ell_0^D\right)^{1/\kappa}} + e^{-\Theta(\ell_0)} + e^{\Theta(\ell_0)}\left[\Theta(\tau) + \Theta\left(\frac{\tau Q\log(Q)}{\ell_0^2}\right)\right]^{\Theta(\ell_0)}\right\}. \qquad (817)
\end{aligned}
$$

Therefore, we can choose the approximate operator for $O_X(\tau)$ as $O_{X_2,\tau}(\partial\tilde{H}^*_{0,X_3,x}, 0\to\tau)$. Here, the operator $O_{X_2,\tau}(\partial\tilde{H}^*_{0,X_3,x}, 0\to\tau)$ does not depend on the interaction terms outside X_3, and hence we can adopt the operator $O_{X_2,\tau}(\partial\tilde{H}^*_{0,X_3,x}, 0\to\tau)$ for the approximation of $O_X(H_{X_3},\tau)$. Thus, by using the same analysis as (523), we prove the main inequality (768). This completes the proof of Proposition 45. □

Supplementary Note 10. LIEB-ROBINSON BOUND IN ONE DIMENSIONAL SYSTEMS

In this section, we prove the optimal Lieb-Robinson bound in which the Lieb-Robinson light cone is almost linear as $\tilde{\mathcal{O}}(t)$. The main statement is given as follows:

Theorem 4. *We here adopt the same setups as in Theorems 2 and 3. Then, under the condition of*

$$
R'' := R - r_0 - \bar{\ell}_{t,R} - 1 \ge \Theta\left[t\log^2(q_0r_0t)\right], \qquad (818)
$$

we obtain the Lieb-Robinson bound as

$$
\left\|\left[O_{X_0}(t) - O_{X_0}\left(H_{i_0[R]},t\right)\right]\rho_0\right\|_1 \le \left\|O_{X_0}\right\|\exp\left\{-\Theta\left(\frac{R''}{t\log(R)}\right)^{1/\kappa} + \Theta[\log(R)]\right\}. \qquad (819)
$$

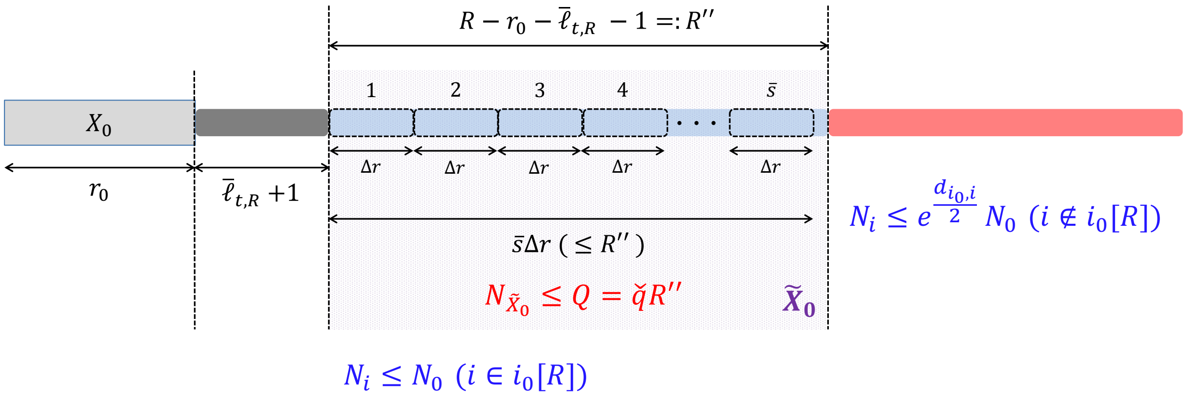

Supplementary Figure 24. Schematic picture of the setup for the proof of Theorem 4. We adopt the projections $\Pi_{\mathcal{S}(i_0[R],1/2,N_0)}$ and $\Pi_{\tilde{X}_0,\le Q}$, where $\tilde{X}_0$ is defined as $\tilde{X}_0 := i_0[R] \setminus X_0[\bar{\ell}_{t,R}+1]$. We restrict ourselves to the Hilbert space spanned by these projections, and the error is estimated in the inequality (825). In the main proof in Sec. Supplementary Note 10 C, we decompose $\tilde{X}_0$ into $\bar{s}$ subspaces and refine the connection of short-time unitary evolutions in (764).

Remark. In the case where $r_0 = \mathcal{O}(R)$ and $q_0 = \mathcal{O}(R)$, the RHS of (819) decays with R as long as

$$R \ge \Theta[t \log^{1+\kappa}(t)]. \tag{820}$$

Therefore, the light cone becomes almost linear as $\tilde{\mathcal{O}}(t)$ in one-dimensional systems.

As a related result, Yin and Lucas have proved [10] that in one-dimensional systems

$$\mathrm{tr}\left([O_X(t), O_Y]\, \rho_0\right) \le \left(\frac{\Theta(t)}{d_{X,Y}}\right)^{\Theta(d_{X,Y})} \tag{821}$$

under a similar assumption to Assumption 3 on the initial state ρ_0. The considered quantity is rather weaker than $\left\|[O_X(t), O_Y]\, \rho_0\right\|_1$ [*13], but the upper bound is stronger in the sense that the commutator decays for $R \ge \Theta(t)$, which means the strictly linear light cone. Moreover, the provided proof there is much simpler than ours. Thus, it leaves a hope to improve the present statement in Theorem 4 to the better form (821) with a more straightforward proof.

A. Preparation

As in Sec. Supplementary Note 9 A, we restrict the Hilbert space which is spanned by the Fock state on $\mathcal{S}(i_0[R], 1/2, N_0)$ ($X_0 = i_0[r_0]$, see also Fig. 24). In addition, we adopt the projection $\Pi_{\tilde{X}_0,\le Q}$ onto the region $\tilde{X}_0$, where $\tilde{X}_0$ is defined as

$$\tilde{X}_0 := i_0[R] \setminus X_0[\bar{\ell}_{t,R} + 1]. \tag{822}$$

In Proposition 32, we set

$$L \to i_0[R], \quad N \to N_0, \quad L' \to \tilde{X}_0, \quad Q \to \check{q}R'' \quad (R'' = R - r_0 - \bar{\ell}_{t,R} - 1). \tag{823}$$

Note that the definitition (822) implies

$$d_{X_0,L'} = \bar{\ell}_{t,R} + 1, \tag{824}$$

and hence the condition (493) is satisfied. Here, we have $|\tilde{X}_0| = R''$, and we can replace ℓ_1 in Eq. (493) by R'', i.e., $\ell_1 \to R''$. Then, from the inequality (500), we obtain

$$\begin{aligned}&\left\| O_{X_0}(H,t)\rho_0 - O_{X_0}\left(\tilde{H}_{(i_0[R],N_0,\tilde{X}_0,Q)}, t\right) \tilde{\Pi}_{(i_0[R],N_0,\tilde{X}_0,Q)}\rho_0\tilde{\Pi}_{(i_0[R],N_0,\tilde{X}_0,Q)} \right\|_1 \\ &\le \zeta_0 e^{-R} + \zeta_0 \Theta\left(tQ\right) e^{-\Theta\left(Q/R''\right)^{1/\kappa}} = \zeta_0 e^{-R} + \zeta_0 \Theta\left(tQ\right) e^{-\Theta(\check{q})^{1/\kappa}},\end{aligned} \tag{825}$$

where the effective Hamiltonian $\tilde{H}_{(i_0[R],N_0,\tilde{X}_0,Q)}$ has been defined in Eq. (494), and we choose N_0 as in Eq. (755), i.e.,

$$N_0 = \Theta\left(q_0^3, R^{2D+1+\kappa}\right) = \Theta\left(q_0^3, R^{\kappa+3}\right) \qquad \text{for} \quad D = 1. \tag{826}$$

[*13] For example, we cannot discuss the gate complexity of the digital quantum simulation only from the bound (821).

We recall that the projection $\tilde{\Pi}_{(i_0[R],N_0,\tilde{X}_0,Q)}$ has been defined in Eq. (502), i.e.,

$$\tilde{\Pi}_{(i_0[R],N_0,\tilde{X}_0,Q)} = \Pi_{\tilde{X}_0,\le Q}\tilde{\Pi}_{(i_0[R],N_0)} = \Pi_{\tilde{X}_0,\le Q}\Pi_{\mathcal{S}(i_0[R],1/2,N_0)}. \tag{827}$$

Also, as in Eq. (756), we adopt the simplified notation of

$$\tilde{H}_{(i_0[R],N_0,\tilde{X}_0,Q)} \to H, \quad \tilde{\Pi}_{(i_0[R],N_0,\tilde{X}_0,Q)}\rho_0\tilde{\Pi}_{(i_0[R],N_0,\tilde{X}_0,Q)} \to \rho_0. \tag{828}$$

B. Main proposition and the proof

The proof of Theorem 4 relies on the following proposition:

Proposition 48. *Let us choose $\check{q}$ as*

$$\check{q} = \Theta\left(\frac{R''}{t\log(R)}\right). \tag{829}$$

Then, under the notations in Eq. (828), we can obtain the following approximation error

$$\left\| \left[O_{X_0}(t) - \bar{O}_{i_0[R]}\right]\rho_0 \right\|_1 \le \zeta_0 e^{\Theta[t\log(N_0)]-\Theta(R'')} \quad \textit{for} \quad R'' \ge \Theta[t\log^{1+\kappa}(N_0)], \tag{830}$$

where we can choose $\bar{O}_{i_0[R]}$ as $O_{X_0}(H_{i_0[R]},t)$ by following the same discussion as in Subtheorem 2 [see also (564)].

By using Proposition 48 with (825), we obtain

$$\left\| \left[O_{X_0}(t) - O_{X_0}(H_{i_0[R]},t)\right]\rho_0 \right\|_1 \le \zeta_0 e^{-R} + \zeta_0\Theta(tQ)\exp\left[-\Theta\left(\frac{R''}{t\log(R)}\right)^{1/\kappa}\right] + \zeta_0 e^{\Theta[t\log(R)]-\Theta(R'')}. \tag{831}$$

Because the second term in the RHS is dominant, we prove the main inequality (819). Finally, because N_0 is given by Eq. (826), the condition $R'' \ge \Theta[t\log^{1+\kappa}(N_0)]$ reduces to (818). This completes the proof of Theorem 4. □

C. Proof of Proposition 48: refined decomposition of the time evolution

In the case of one-dimensional systems, if we use the same approach in Sec. Supplementary Note 9 B, we cannot obtain the optimal Lieb-Robinson light cone as $R = \tilde{\mathcal{O}}(t)$ (see also Remark in Theorem 3). To refine the bound, we take a similar but different approach. In this section, we adopt the notations τ, X_m and $\bar{m}$ similar to Eqs. (757) and (758) (see also Fig. 24):

$$t := \bar{m}\tau,$$
$$X_m := i_0[r_0 + \bar{\ell}_{t,R} + 1 + m\Delta r], \tag{832}$$
$$\Delta r = \left\lfloor \frac{R-(r_0+\bar{\ell}_{t,R}+1)}{\bar{s}} \right\rfloor = \left\lfloor \frac{R''}{\bar{s}} \right\rfloor \quad (\bar{s} \ge \bar{m}), \tag{833}$$
$$R'' := R - r_0 - \bar{\ell}_{t,R} - 1, \tag{834}$$

where $X_{\bar{s}} \subseteq i_0[R]$ and $\bar{s}$ is appropriately chosen [see (910) below].

Here, we choose τ as an $\mathcal{O}(1)$ constant [$\tau \le 1/(4\gamma \bar{J})$], which also implies

$$\bar{m} := t/\tau = \Theta(t). \tag{835}$$

On the other hand, we redefine the parameter Δr as follows:

$$\Delta r = \left\lfloor \frac{R''}{\bar{s}} \right\rfloor \quad (\bar{s} \ge \bar{m}), \tag{836}$$

where By using Δr and R, we choose $\check{q}$ such that

$$\check{q} = \Theta\left(\frac{\Delta r}{\log(R)}\right), \tag{837}$$

which reduces to the form of Eq. (829) from $\Delta r = \Theta(R''/\bar{s})$ and $\bar{s} = \Theta(t)$. From the inequality (825), $\check{q}$ should be at least larger than $\log^{\kappa}(R)$, or else the upper bound in (825) is worse than the trivial bound. Thus, from $\kappa \ge 1$, we add the following additional condition to Δr as

$$\frac{\Delta r}{\log(R)} \ge \Theta[\log^{\kappa}(R)], \tag{838}$$

which leads to the choice of Δr as

$$\Delta r = \Theta[\log^{1+\kappa}(N_0)] \ge \Theta[\log^{1+\kappa}(R)]. \tag{839}$$

The above condition yields the condition $R'' \ge \Theta[t \log^{1+\kappa}(N_0)]$ in the main inequality (830).

In the following, we refine the previous decomposition, which has been given in Eq. (764) (see Fig. 25 for the schematic picture of the present decomposition). Our purpose here is to derive the equation (850) and the inequality (852). We start from the decomposition of the time-evolved operator $O_{X_0}(\tau)$ as follows:

$$O_{X_0}(\tau) = \sum_{s_1=1}^{\bar{s}+1} \Delta O_{s_1}, \tag{840}$$

where we define $\Delta O_{s_1}^{(1)}$ as

$$\begin{aligned}
&\Delta O_1 := U_1^\dagger O_{X_0} U_1, \\
&\Delta O_{s_1} := U_{s_1}^\dagger O_{X_0} U_{s_1} - U_{s_1-1}^\dagger O_{X_0} U_{s_1-1} \quad (2 \le s_1 \le \bar{s}), \\
&\Delta O_{\bar{s}+1} := O_{X_0}(\tau) - U_{\bar{s}}^\dagger O_{X_0} U_{\bar{s}},
\end{aligned} \tag{841}$$

where, for an arbitrary s, we denote U_s as the time evolution in the region X_s, i.e.,

$$U_s := e^{-iH_{X_s}\tau}. \tag{842}$$

In the next step, we consider the decomposition of $\Delta O_{s_1}(\tau)$ as follows:

$$\Delta O_{s_1}(\tau) = \sum_{s_2=\min(\bar{s}+1, s_1+1)}^{\bar{s}+1} \Delta O_{s_1,s_2}, \tag{843}$$

where $\Delta O_{s_1,s_2}$ $(s_1 \le \bar{s})$ is defined as

$$\begin{aligned}
&\Delta O_{s_1,s_1+1} := U_{s_1+1}^\dagger \Delta O_{s_1} U_{s_1+1}, \\
&\Delta O_{s_1,s_2} := U_{s_2}^\dagger \Delta O_{s_1} U_{s_2} - U_{s_2-1}^\dagger \Delta O_{s_1} U_{s_2-1} \quad (s_1+2 \le s_2 \le \bar{s}), \\
&\Delta O_{s_1,\bar{s}+1} := \Delta O_{s_1}(\tau) - U_{\bar{s}}^\dagger \Delta O_{s_1} U_{\bar{s}}.
\end{aligned} \tag{844}$$

Note that we adopt the same definition for U_s as in Eq. (842). In the case of $s_1 = \bar{s}+1$, we define $\Delta O_{s_1,s_2}$ as

$$\Delta O_{\bar{s}+1,\bar{s}+1} := \Delta O_{\bar{s}+1}(\tau) \quad \text{for} \quad s_1 = \bar{s}+1. \tag{845}$$

By combining Eqs. (840) and (843), we obtain

$$O_{X_0}(2\tau) = \sum_{s_1=1}^{\bar{s}+1} \sum_{s_2=\min(\bar{s}+1, s_1+1)}^{\bar{s}+1} \Delta O_{s_1,s_2}. \tag{846}$$

By repeating this process, we recursively define $\Delta O_{\mathbf{s}_m}$ ($\mathbf{s}_m := \{s_1, \ldots, s_{m-1}, s_m\}$, $s_{m-1} \le \bar{s}$) as

$$\begin{aligned}
&\Delta O_{\mathbf{s}_m} := U_{s_{m-1}+1}^\dagger \Delta O_{\mathbf{s}_{m-1}} U_{s_{m-1}+1} \quad (s_m = s_{m-1}+1), \\
&\Delta O_{\mathbf{s}_m} := U_{s_m}^\dagger \Delta O_{\mathbf{s}_{m-1}} U_{s_m} - U_{s_m-1}^\dagger \Delta O_{\mathbf{s}_{m-1}} U_{s_m-1} \quad (s_{m-1}+2 \le s_m \le \bar{s}), \\
&\Delta O_{\mathbf{s}_m} := \Delta O_{\mathbf{s}_{m-1}}(\tau) - U_{\bar{s}}^\dagger \Delta O_{\mathbf{s}_{m-1}} U_{\bar{s}} \quad (s_m = \bar{s}+1).
\end{aligned} \tag{847}$$

In the case of $s_{m-1} = \bar{s}+1$, we define $\Delta O_{\mathbf{s}_m}$ as

$$\Delta O_{\mathbf{s}_m} := \Delta O_{\mathbf{s}_{m-1}}(\tau) \quad \text{for} \quad s_{m-1} = s_m = \bar{s}+1. \tag{848}$$

By using the above notations, we obtain

$$\Delta O_{\mathbf{s}_{m-1}}(\tau) = \sum_{s_m=\min(\bar{s}+1, s_{m-1}+1)}^{\bar{s}+1} \Delta O_{\mathbf{s}_m}. \tag{849}$$

By applying Eq. (849) iteratively, we have the decomposition of $O_{X_0}(\bar{m}\tau) = O_{X_0}(t)$ as follows:

$$O_{X_0}(\bar{m}\tau) = O_{X_0}(t) = \sum_{s_1=1}^{\bar{s}+1} \sum_{s_2=\min(\bar{s}+1, s_1+1)}^{\bar{s}+1} \cdots \sum_{s_{\bar{m}}=\min(\bar{s}+1, s_{\bar{m}-1}+1)}^{\bar{s}+1} \Delta O_{\mathbf{s}_{\bar{m}}}. \tag{850}$$

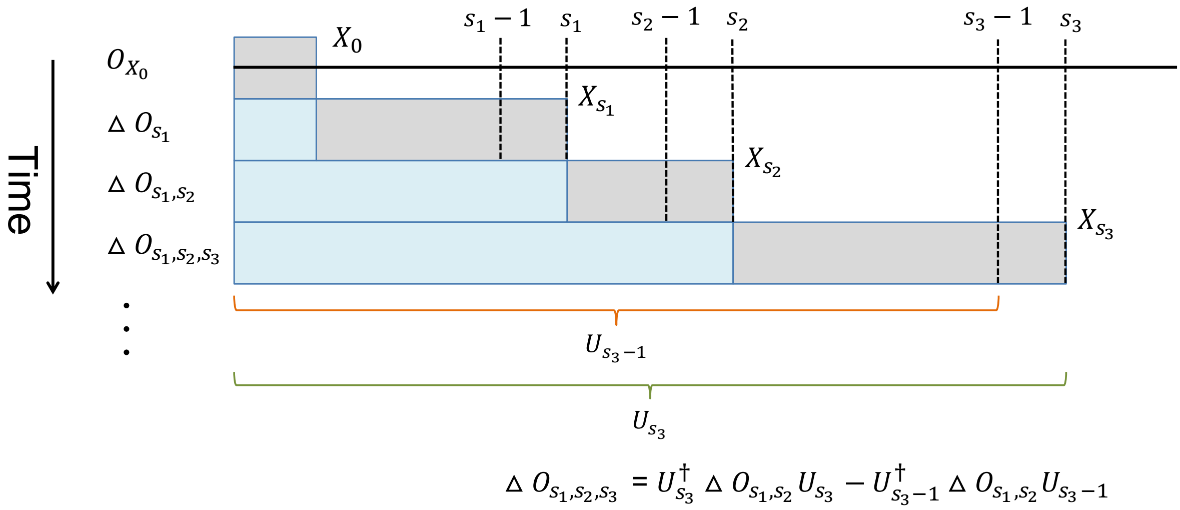

Supplementary Figure 25. Schematic picture of the new decomposition (850). We start from the time evolution of $O_{X_0}(\tau)$, which is decomposed to the summation of $\{\Delta O_{s_1}\}_{s_1=1}^{\bar{s}+1}$ as in Eq. (840). Here, ΔO_{s_1} is defined by using the unitary operator U_{s_1} [see Eq. (842)] and is supported on the subset X_{s_1}. In the next step, the time evolution of the decomposed operator, i.e., $\Delta O_{s_1}(\tau)$, is also decomposed to the summation of $\{\Delta O_{s_1,s_2}\}_{s_2=s_1+1}^{\bar{s}+1}$ as in Eq. (843). In the same way, the time evolution of $\Delta O_{s_1,s_2}(\tau)$ is further decomposed to $\{\Delta O_{s_1,s_2,s_3}\}_{s_3=s_2+1}^{\bar{s}+1}$. By connecting the decompositions, we obtain the full decomposition as in Eq. (850). By truncating the summation for $s_1, s_2, \dots, s_{\bar{m}}$ up to $\bar{s}$, all the decomposed operators $\Delta O_{\mathbf{s}_{\bar{m}}}$ are supported on the subset $X_{\bar{s}} \subseteq i_0[R]$, and hence we can obtain the refined approximation for the time evolution $O_{X_0}(t)$ [see Eq. (851)].

Then, by truncating the summation for each of $\{s_m\}_{m=1}^{\bar{m}}$ up to $\bar{s}$, we define $\bar{O}_{X_{\bar{s}}}$ as

$$\bar{O}_{X_{\bar{s}}} := \sum_{s_1=1}^{\bar{s}} \sum_{s_2=s_1+1}^{\bar{s}} \cdots \sum_{s_m=s_{m-1}+1}^{\bar{s}} \Delta O_{\mathbf{s}_m}. \tag{851}$$

Here, the operator $\bar{O}_{X_{\bar{s}}}$ is supported on the subset $X_{\bar{s}} \subseteq i_0[R]$ [see Eq. (832)]. Therefore, we need to upper-bound the norm of

$$\left\| \left[O_{X_0}(t) - \bar{O}_{X_{\bar{s}}}\right] \rho_0 \right\|_1 \le \sum_{\mathbf{s}_{\bar{m}} \in V_{\bar{s}+1}} \|\Delta O_{\mathbf{s}_{\bar{m}}} \rho_0\|_1, \tag{852}$$

where $V_{\bar{s}+1}$ is a set of $\mathbf{s}_{\bar{m}}$ such that at least one $s_p \in \mathbf{s}_{\bar{m}}$ is equal to $\bar{s}+1$.

In general, for the estimation of $\|\Delta O_{\mathbf{s}_m} \rho_0\|_1$ $(1 \le m \le \bar{m})$, we need to estimate the norms like

$$\left\| \left(U_{s_m}^{\dagger} \Delta O_{\mathbf{s}_{m-1}} U_{s_m} - U_{s_m-1}^{\dagger} \Delta O_{\mathbf{s}_{m-1}} U_{s_m-1} \right) \rho_0 \right\|_1. \tag{853}$$

However, we here encounter the following obstacle. By using the short-time Lieb-Robinson bound in Proposition 45, we can only obtain an upper bound in the form of

$$\left\| \left(U_{s_m}^{\dagger} \Delta O_{\mathbf{s}_{m-1}} U_{s_m} - U_{s_m-1}^{\dagger} \Delta O_{\mathbf{s}_{m-1}} U_{s_m-1} \right) \rho_0 \right\|_1 \le \left\| \Delta O_{\mathbf{s}_{m-1}} \right\| \varepsilon_{s_{m-1},s_m} \tag{854}$$

instead of

$$\left\| \left(U_{s_m}^{\dagger} \Delta O_{\mathbf{s}_{m-1}} U_{s_m} - U_{s_m-1}^{\dagger} \Delta O_{\mathbf{s}_{m-1}} U_{s_m-1} \right) \rho_0 \right\|_1 \le \left\| \Delta O_{\mathbf{s}_{m-1}} \rho_0 \right\|_1 \varepsilon_{s_{m-1},s_m}, \tag{855}$$

where $\varepsilon_{s_{m-1},s_m}$ is a small constant which depends on s_{m-1} and s_m. If we could obtain the bound as (855), we can iteratively upper-bound $\|\Delta O_{\mathbf{s}_m} \rho_0\|_1$ for $m \ge 1$, which may yield the desired inequality (830). However, the inequality (855) does not seem to hold in general.

In the following sections, we aim to refine the decomposition (850) furthermore.

D. Proof of Proposition 48: inserting projections

To overcome the difficulty, we introduce a projection operator $P_{s,s'}^{(\sigma)}$ $(0 \le s \le s'$, $\sigma = 0, 1)$ such that $P_{s,s'}^{(0)} + P_{s,s'}^{(1)} = 1$ which is supported on $X_{s'} \setminus X_s$ (see also Fig. 26). By using the projection, we first decompose ΔO_{s_1} as

$$\Delta O_{s_1} = \Delta O_{s_1} P_{0,s_1}^{(0)} + \Delta O_{s_1} P_{0,s_1}^{(1)} =: \Delta \tilde{O}_{s_1,0} + \Delta \tilde{O}_{s_1,1}. \tag{856}$$

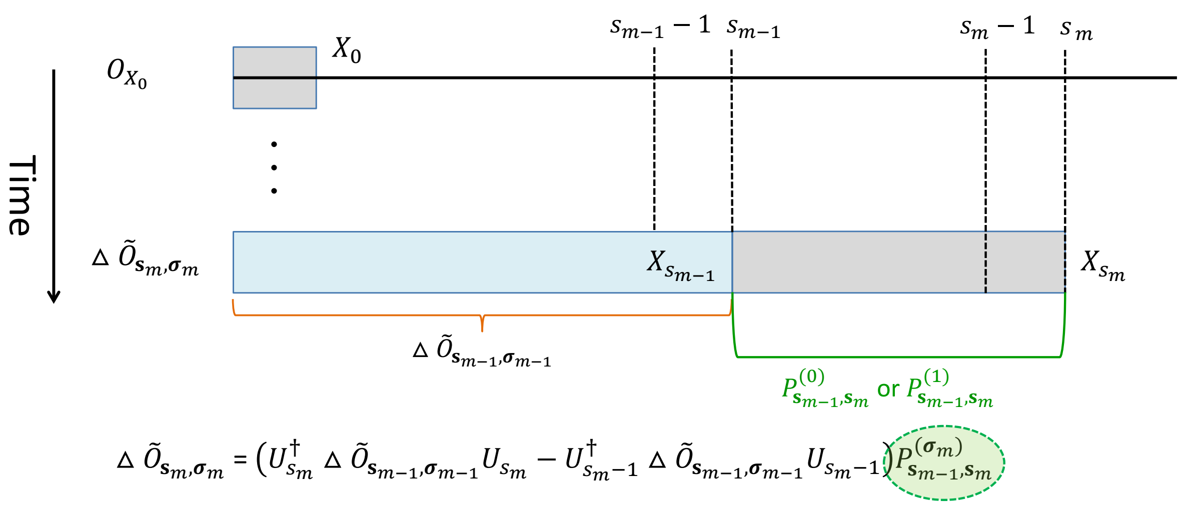

Supplementary Figure 26. New decomposed operator $\Delta\tilde{O}_{\mathbf{s}_m,\boldsymbol{\sigma}_m}$ in Eq. (860). We here adopt the projection operators $P^{(\sigma)}_{s,s'}$ with $0\le s\le s'\le \bar{s}+1$ and $\sigma=0,1$, which is supported on $X_{s'}\setminus X_s$. The explicit definition of the projection will be given in Eq. (863). In every step from the time $(m-1)\tau$ to $m\tau$, we insert the projection $P^{(0)}_{s_{m-1},s_m}$ or $P^{(1)}_{s_{m-1},s_m}$. Regarding the projection indices $\boldsymbol{\sigma}_m=\{\sigma_j\}_{j=1}^m$, we redefine the new decomposed operators $\Delta\tilde{O}_{\mathbf{s}_m,\boldsymbol{\sigma}_m}$ as in Eq. (859).

In the same way, we decompose $\Delta O_{s_1,s_2}$ in Eq. (844) as

$$
\begin{aligned}
\Delta O_{s_1,s_2} &= \sum_{\sigma_1=0,1}\left(U^\dagger_{s_2}\Delta\tilde{O}_{s_1,\sigma_1}U_{s_2} - U^\dagger_{s_2-1}\Delta\tilde{O}_{s_1,\sigma_1}U_{s_2-1}\right)\left(P^{(0)}_{s_1,s_2}+P^{(1)}_{s_1,s_2}\right)\\
&=: \sum_{\sigma_1=0,1}\sum_{\sigma_2=0,1}\Delta\tilde{O}_{s_1,s_2,\sigma_1,\sigma_2},
\end{aligned}
\tag{857}
$$

where we consider the case of $s_1+2\le s_2\le\bar{s}$ in Eq. (844) for simplicity. The above decomposition reduces Eq. (846) to

$$
O_{X_0}(2\tau) = \sum_{s_1=1}^{\bar{s}+1}\sum_{s_2=\min(\bar{s}+1,s_1+1)}^{\bar{s}+1}\sum_{\sigma_1=0,1}\sum_{\sigma_2=0,1}\Delta\tilde{O}_{s_1,s_2,\sigma_1,\sigma_2}.
\tag{858}
$$

By repeating the same procedure, we decompose $\Delta\tilde{O}_{\mathbf{s}_m}$ as

$$
\begin{aligned}
\Delta\tilde{O}_{\mathbf{s}_m} &= \sum_{\boldsymbol{\sigma}_m\in\{0,1\}^{\otimes m}}\Delta\tilde{O}_{\mathbf{s}_m,\boldsymbol{\sigma}_m},\\
\Delta\tilde{O}_{\mathbf{s}_m,\boldsymbol{\sigma}_m} &:= \left(U^\dagger_{s_m}\Delta\tilde{O}_{\mathbf{s}_{m-1},\boldsymbol{\sigma}_{m-1}}U_{s_m} - U^\dagger_{s_m-1}\Delta\tilde{O}_{\mathbf{s}_{m-1},\boldsymbol{\sigma}_{m-1}}U_{s_m-1}\right)P^{(\sigma_m)}_{s_{m-1},s_m}.
\end{aligned}
\tag{859}
$$

By using the notation of $\Delta\tilde{O}_{\mathbf{s}_m,\boldsymbol{\sigma}_m}$, we reduce Eqs. (850) and (851) to

$$
\begin{aligned}
O_{X_0}(t) &= \sum_{\boldsymbol{\sigma}_{\bar{m}}\in\{0,1\}^{\otimes\bar{m}}}\sum_{s_1=1}^{\bar{s}+1}\sum_{s_2=\min(\bar{s}+1,s_1+1)}^{\bar{s}+1}\cdots\sum_{s_{\bar{m}}=\min(\bar{s}+1,s_{\bar{m}-1}+1)}^{\bar{s}+1}\Delta\tilde{O}_{\mathbf{s}_{\bar{m}},\boldsymbol{\sigma}_{\bar{m}}},\\
\bar{O}_{X_{\bar{s}}} &= \sum_{\boldsymbol{\sigma}_{\bar{m}}\in\{0,1\}^{\otimes\bar{m}}}\sum_{s_1=1}^{\bar{s}}\sum_{s_2=s_1+1}^{\bar{s}}\cdots\sum_{s_{\bar{m}}=s_{\bar{m}-1}+1}^{\bar{s}}\Delta\tilde{O}_{\mathbf{s}_{\bar{m}},\boldsymbol{\sigma}_{\bar{m}}}.
\end{aligned}
\tag{860}
$$

By using the above equations, we have the approximation of

$$
\left\|\left[O_{X_0}(t)-\bar{O}_{X_{\bar{s}}}\right]\rho_0\right\|_1 \le \sum_{\boldsymbol{\sigma}_{\bar{m}}\in\{0,1\}^{\otimes\bar{m}}}\sum_{\mathbf{s}_{\bar{m}}\in V_{\bar{s}+1}}\left\|\Delta\tilde{O}_{\mathbf{s}_{\bar{m}},\boldsymbol{\sigma}_{\bar{m}}}\rho_0\right\|_1.
\tag{861}
$$

Remember that $V_{\bar{s}+1}$ has been defined as the set of $\mathbf{s}_{\bar{m}}$ such that at least one $s_p\in\mathbf{s}_{\bar{m}}$ is equal to $\bar{s}+1$. We aim to upper-bound the norm $\left\|\left[O_{X_0}(t)-\bar{O}_{X_{\bar{s}}}\right]\rho_0\right\|_1$ based on this decomposition by appropriately defining the projection $P^{(\sigma)}_{s,s'}$.

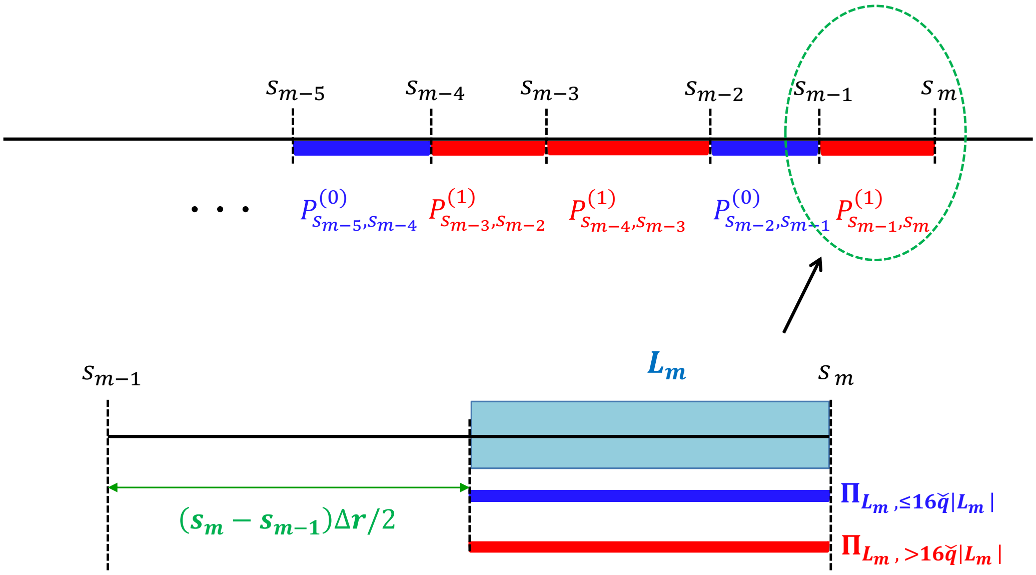

Supplementary Figure 27. Schematic picture of the definition (863) for the projections $P^{(0)}_{s_{m-1},s_m}$ and $P^{(1)}_{s_{m-1},s_m}$. Each of the projections characterizes the number of bosons in the region L_m, which is the half region between X_{s_m} and $X_{s_{m-1}}$, i.e., $L_m = X_{s_m} \setminus X_{s_{m-1}}[(s_m - s_{m-1})\Delta r/2]$. Here, the projection $P^{(0)}_{s_{m-1},s_m}$ implies a small boson number in the region L_m, while the projection $P^{(1)}_{s_{m-1},s_m}$ implies a large boson number.

E. Proof of Proposition 48: estimation of the decomposed operators $\Delta\tilde{O}_{\mathbf{s}_m,\boldsymbol{\sigma}_m}$

In the following, we aim to upper-bound the norm of

$$\left\|\Delta\tilde{O}_{\mathbf{s}_m,\boldsymbol{\sigma}_m}\right\| \tag{862}$$

for arbitrary fixed choices of $\mathbf{s}_m$ ($\in V_{\bar{s}+1}$) and $\boldsymbol{\sigma}_m$. We here choose the projection operator $P^{(\sigma_m)}_{s_{m-1},s_m}$ as follows (see Fig. 27):

$$\begin{aligned}
&P^{(0)}_{s_{m-1},s_m} = \Pi_{L_m,\le 16\check{q}|L_m|},\\
&P^{(1)}_{s_{m-1},s_m} = \Pi_{L_m,>16\check{q}|L_m|},\\
&L_m := X_{s_m} \setminus X_{s_{m-1}}[(s_m - s_{m-1})\Delta r/2],
\end{aligned} \tag{863}$$

where the constant $\check{q}$ is an appropriately defined parameter as in Eq. (829). We recall that $\Pi_{L_m,\le 16\check{q}|L_m|}$ has been defined as a projector onto the eigenspace of $\hat{n}_{L_m}$ with the eigenvalues smaller than $16\check{q}|L_m|$. Here, L_m is a half region from the point s_{m-1} to s_m (see Fig. 27). Roughly speaking, the projection $P^{(0)}_{s_{m-1},s_m}$ restricts the boson number in the region L_m, while the projection $P^{(1)}_{s_{m-1},s_m}$ permits unlimited number of bosons in the region L_m.

Based on the above definition, we take the following strategy (see also Fig. 28):

1. The operator $\left\|\Delta\tilde{O}_{\mathbf{s}_m,\boldsymbol{\sigma}_m}\right\|$ includes m projections $\{P^{(\sigma_j)}_{s_{j-1},s_j}\}_{j=1}^m$. If the projection is given by $P^{(0)}_{s_{j-1},s_j}$, we can apply the Lieb-Robinson bound for the short-time evolution (i.e., Proposition 44) from time $(j-1)\tau$ to $j\tau$ in the region $X_{s_m} \setminus X_{s_{m-1}}$. On the other hand, if the projection is given by $P^{(1)}_{s_{j-1},s_j}$, we cannot obtain a meaningful Lieb-Robinson bound.

2. If many of the region have the indices $\sigma_j = 0$ [Fig. 28 (a)], we can ensure that the norm $\left\|\Delta\tilde{O}_{\mathbf{s}_m,\boldsymbol{\sigma}_m}\right\|$ is sufficiently small by connecting the short-time Lieb-Robinson bound [see Lemma 49 and Corollary 50].

3. Conversely, if many of the region have the indices $\sigma_j = 1$ [Fig. 28 (b)], we have to take another approach to upper-bound the norm $\left\|\Delta\tilde{O}_{\mathbf{s}_m,\boldsymbol{\sigma}_m}\right\|$. We have already upper-bounded the total number of bosons in the region $\tilde{X}_0$ by Q as in Eq. (828). Hence, such a constraint allows us to upper-bound $\left\|\Delta\tilde{O}_{\mathbf{s}_m,\boldsymbol{\sigma}_m}\right\|$ when many of the region have large boson numbers [see Lemma 51].

We first consider the case with many low-boson-density projections [Fig. 28 (a)]. By using Proposition 44, we prove the following lemma:

Lemma 49. *Let us choose $\check{q}$ as in (837). Then, we can upper-bound the norm $\left\|\Delta\tilde{O}_{\mathbf{s}_m,\boldsymbol{\sigma}_m}\right\|$ by using $\left\|\Delta\tilde{O}_{\mathbf{s}_{m-1},\boldsymbol{\sigma}_{m-1}}\right\|$ as follows:*

$$\begin{aligned}
&\left\|\Delta\tilde{O}_{\mathbf{s}_m,\boldsymbol{\sigma}_m}\right\| \le 2\left\|\Delta\tilde{O}_{\mathbf{s}_{m-1},\boldsymbol{\sigma}_{m-1}}\right\| \quad \textit{for} \quad \sigma_m = 1,\\
&\left\|\Delta\tilde{O}_{\mathbf{s}_m,\boldsymbol{\sigma}_m}\right\| \le \lambda_Q \delta_{\Delta r}^{s_m - s_{m-1} - 2}\left\|\Delta\tilde{O}_{\mathbf{s}_{m-1},\boldsymbol{\sigma}_{m-1}}\right\| \quad \textit{for} \quad \sigma_m = 0,
\end{aligned} \tag{864}$$

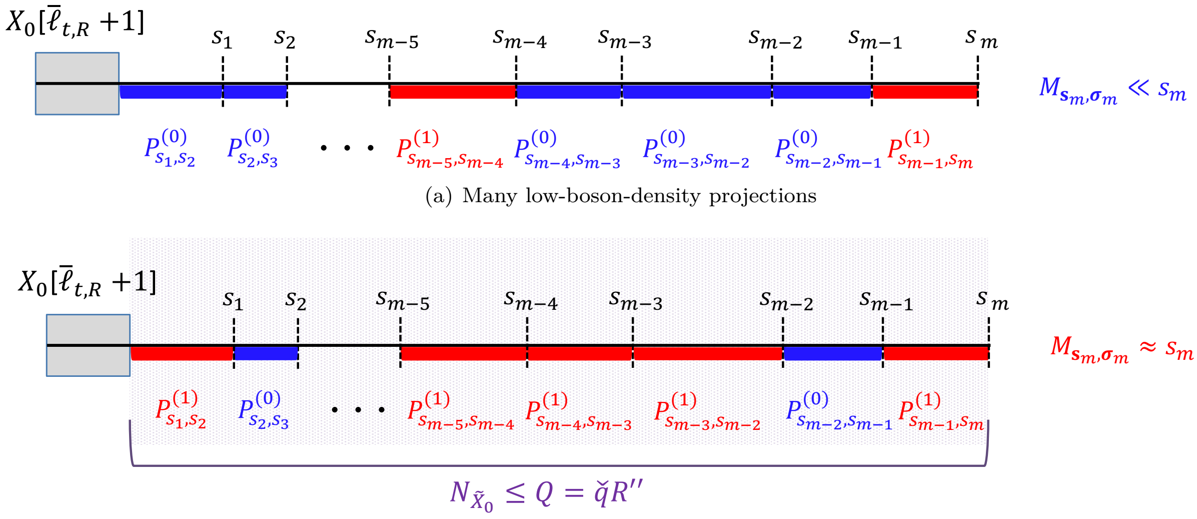

(a) Many low-boson-density projections

(b) Many high-boson-density projections

Supplementary Figure 28. The two cases of the projection patterns $\{P^{(\sigma_j)}_{s_{j-1},s_j}\}_{j=1}^m$. Now, the parameter $M_{\mathbf{s}_m,\boldsymbol{\sigma}_m} = \sum_{j=1}^m \sigma_j(s_j - s_{j-1})$ in Eq. (875) is used to characterize the ratio of the region with low (or high)-boson density. Note that $0 \le M_{\mathbf{s}_m,\boldsymbol{\sigma}_m} \le s_m$. In the first case (a), the majority of the projections have the index $\sigma_j = 0$; that is, we have $M_{\mathbf{s}_m,\boldsymbol{\sigma}_m} \ll s_m$. We then apply the short-time Lieb-Robinson bound in Proposition 44 to the time evolution with the projections $\{P^{(\sigma_j)}_{s_{j-1},s_j}\}_{j=1}^m$ such that $\sigma_j = 0$, which yields Lemma 49 and Corollary 50. On the other hand, in the second case (b), the majority of the projections have the index $\sigma_j = 1$, i.e., $M_{\mathbf{s}_m,\boldsymbol{\sigma}_m} \approx s_m$. In this case, it means that so many bosons exist in the region $X_{s_m} \setminus X_0[\bar{\ell}_{t,R}+1]$. However, we have truncated the number of bosons in the region $\tilde{X}_0 := i_0[R] \setminus X_0[\bar{\ell}_{t,R}+1]$ by Q as in Eq. (828). This allows us to efficiently upper-bound the norm $\left\|\Delta\tilde{O}_{\mathbf{s}_m,\boldsymbol{\sigma}_m}\right\|$ as in Lemma 51.

where $\delta_{\Delta r}$ and λ_Q are constants such that

$$\delta_{\Delta r} = e^{-\Theta(\Delta r)}, \quad \lambda_Q = \Theta(Q^4 R^D N_0). \tag{865}$$

1. Proof of Lemma 49

From the definition (859), the first inequality in (864) is trivial, and hence we need to prove the second inequality. By using the expression (859) and the triangle inequality, we first obtain

$$\begin{aligned}
\left\|\Delta\tilde{O}_{\mathbf{s}_m,\boldsymbol{\sigma}_m}\right\| \le& \left\|\left[\Delta\tilde{O}_{\mathbf{s}_{m-1},\boldsymbol{\sigma}_{m-1}}(H,\tau) - U^\dagger_{s_m}\Delta\tilde{O}_{\mathbf{s}_{m-1},\boldsymbol{\sigma}_{m-1}}U_{s_m}\right] P^{(\sigma_m)}_{s_{m-1},s_m}\right\| \\
&+ \left\|\left[\Delta\tilde{O}_{\mathbf{s}_{m-1},\boldsymbol{\sigma}_{m-1}}(H,\tau) - U^\dagger_{s_m-1}\Delta\tilde{O}_{\mathbf{s}_{m-1},\boldsymbol{\sigma}_{m-1}}U_{s_m-1}\right] P^{(\sigma_m)}_{s_{m-1},s_m}\right\| \\
=& \left\|\left[\Delta\tilde{O}_{\mathbf{s}_{m-1},\boldsymbol{\sigma}_{m-1}}(H,\tau) - \Delta\tilde{O}_{\mathbf{s}_{m-1},\boldsymbol{\sigma}_{m-1}}(H_{X_{s_m}},\tau)\right] P^{(\sigma_m)}_{s_{m-1},s_m}\right\| \\
&+ \left\|\left[\Delta\tilde{O}_{\mathbf{s}_{m-1},\boldsymbol{\sigma}_{m-1}}(H,\tau) - \Delta\tilde{O}_{\mathbf{s}_{m-1},\boldsymbol{\sigma}_{m-1}}(H_{X_{s_m-1}},\tau)\right] P^{(\sigma_m)}_{s_{m-1},s_m}\right\|,
\end{aligned} \tag{866}$$

where we use the definition (842) of U_s. From the abbreviation of the notation as in (828), H, $H_{X_{s_m}}$ and $H_{X_{s_m-1}}$ are given by the effective Hamiltonian under the projection $\tilde{\Pi}_{(i_0[R],N_0,\tilde{X}_0,Q)}$. For $\sigma_m = 0$, we need to estimate

$$\begin{aligned}
&\left\|\left[\Delta\tilde{O}_{\mathbf{s}_{m-1},\boldsymbol{\sigma}_{m-1}}(H,\tau) - \Delta\tilde{O}_{\mathbf{s}_{m-1},\boldsymbol{\sigma}_{m-1}}(H_{X_{s_m}},\tau)\right] P^{(0)}_{s_{m-1},s_m}\right\| \\
&= \left\|\left[\Delta\tilde{O}_{\mathbf{s}_{m-1},\boldsymbol{\sigma}_{m-1}}(H,\tau) - \Delta\tilde{O}_{\mathbf{s}_{m-1},\boldsymbol{\sigma}_{m-1}}(H_{X_{s_m}},\tau)\right] \Pi_{L_m,\le 16\check{q}|L_m|}\right\|,
\end{aligned} \tag{867}$$

where we use the definition (863) for $P^{(0)}_{s_{m-1},s_m}$.

Because of the abbreviated notation (828), we here consider the effective Hamiltonian which is constructed by the projections $\Pi_{\tilde{X}_0,\le Q}$ and $\Pi_{\mathcal{S}(i_0[R],1/2,N_0)}$. We hence apply Proposition 44 to $\Delta\tilde{O}_{\mathbf{s}_{m-1},\boldsymbol{\sigma}_{m-1}}(\tau)$ instead of Subtheorem 2. The operator $\Delta\tilde{O}_{\mathbf{s}_{m-1},\boldsymbol{\sigma}_{m-1}}$ is supported on $X_{s_{m-1}} \subseteq i_0[R]$, and hence the boson creation/annihilation by the operator is upper-bounded by

$$N_0|X_{s_{m-1}}| \le \gamma R^D N_0, \quad \text{i.e.,} \quad q_{Z_0} \to \gamma R^D N_0, \tag{868}$$

because of the constraint $\mathcal{S}(i_0[R],1/2,N_0)$. In the inequality (714) of Proposition 44, we set

$$O_{Z_0} \to \Delta\tilde{O}_{\mathbf{s}_{m-1},\boldsymbol{\sigma}_{m-1}}, \quad \zeta_{Z_0} \to \left\|\Delta\tilde{O}_{\mathbf{s}_{m-1},\boldsymbol{\sigma}_{m-1}}\right\|, \quad \ell_0 \to |L_m|, \quad Q \to 16\check{q}|L_m|, \quad \tilde{L} \to L_m, \quad \bar{L} = i_0[R]. \tag{869}$$

Here, the condition $\ell_0 \geq \Theta[\log(q_{Z_0} N_0)]$ in Proposition 44 reduces to $|L_m| \geq \Theta[\log(N_0)]$ because of $q_{Z_0} \leq \text{poly}(N_0)$ from Eq. (826). We thus obtain

$$\begin{aligned}&\left\| \left[\Delta\tilde{O}_{\mathbf{s}_{m-1},\boldsymbol{\sigma}_{m-1}}(H,\tau) - \Delta\tilde{O}_{\mathbf{s}_{m-1},\boldsymbol{\sigma}_{m-1}}(H_{X_{s_m}},\tau)\right] P^{(0)}_{s_{m-1},s_m} \right\| \\ &\leq \left\|\Delta\tilde{O}_{\mathbf{s}_{m-1},\boldsymbol{\sigma}_{m-1}}\right\| \Theta(Q^4 R^D N_0) \left\{ e^{-\Theta(|L_m|)} + e^{\Theta(|L_m|)} \left[\Theta(\tau) + \Theta\left(\frac{\check{q}\tau \log(\check{q}|L_m|)}{|L_m|}\right)\right]^{\Theta(|L_m|)} \right\}. \end{aligned} \tag{870}$$

By using the condition (837), i.e., $\check{q} \leq \Theta[\Delta r/\log(R)]$, and $|L_m| \geq \Theta(\Delta r)$, we have

$$\Theta(\tau) + \Theta\left(\frac{\check{q}\tau \log(\check{q}|L_m|)}{|L_m|}\right) = \Theta(\tau), \tag{871}$$

which reduces the inequality (870) to

$$\begin{aligned}&\left\| \left[\Delta\tilde{O}_{\mathbf{s}_{m-1},\boldsymbol{\sigma}_{m-1}}(H,\tau) - \Delta\tilde{O}_{\mathbf{s}_{m-1},\boldsymbol{\sigma}_{m-1}}(H_{X_{s_m}},\tau)\right] P^{(0)}_{s_{m-1},s_m} \right\| \\ &\leq \left\|\Delta\tilde{O}_{\mathbf{s}_{m-1},\boldsymbol{\sigma}_{m-1}}\right\| \Theta(Q^4 R^D N_0) e^{-\Theta(|L_m|)}\end{aligned} \tag{872}$$

under an appropriate choice of τ. In the same way, we obtain

$$\begin{aligned}&\left\| \left[\Delta\tilde{O}_{\mathbf{s}_{m-1},\boldsymbol{\sigma}_{m-1}}(H,\tau) - \Delta\tilde{O}_{\mathbf{s}_{m-1},\boldsymbol{\sigma}_{m-1}}(H_{X_{s_{m-1}}},\tau)\right] P^{(0)}_{s_{m-1},s_m} \right\| \\ &\leq \left\|\Delta\tilde{O}_{\mathbf{s}_{m-1},\boldsymbol{\sigma}_{m-1}}\right\| \Theta(Q^4 R^D N_0) e^{-\Theta(|L_m|-\Delta r)},\end{aligned} \tag{873}$$

where we use $X_{s_m} = X_{s_m - 1}[\Delta r]$. By applying the inequalities (872) and (873) to (866), we finally obtain

$$\begin{aligned}\left\|\Delta\tilde{O}_{\mathbf{s}_m,\boldsymbol{\sigma}_m}\right\| &\leq \left\|\Delta\tilde{O}_{\mathbf{s}_{m-1},\boldsymbol{\sigma}_{m-1}}\right\| \Theta(Q^4 R^D N_0) e^{-\Theta(|L_m|-\Delta r)} \\ &= \Theta(Q^4 R^D N_0) e^{-\Theta[(s_m - s_{m-1} - 2)\Delta r/2]} \left\|\Delta\tilde{O}_{\mathbf{s}_{m-1},\boldsymbol{\sigma}_{m-1}}\right\|,\end{aligned} \tag{874}$$

where we use the definition (863) of L_m, which implies $|L_m| = (s_m - s_{m-1})\Delta r/2$. We thus prove the inequality (864). □

[**End of Proof of Lemma 49**]

By iteratively applying Lemma 49 to $\left\|\Delta\tilde{O}_{\mathbf{s}_m,\boldsymbol{\sigma}_m}\right\|$, we can immediately prove the following corollary:

Corollary 50. *Let us define* $M_{\mathbf{s}_m,\boldsymbol{\sigma}_m}$ *as*

$$M_{\mathbf{s}_m,\boldsymbol{\sigma}_m} := \sum_{j=1}^{m} \sigma_j (s_j - s_{j-1}), \tag{875}$$

where we set $s_0 = 0$*. We then obtain the upper bound of*

$$\left\|\Delta\tilde{O}_{\mathbf{s}_m,\boldsymbol{\sigma}_m}\right\| \leq \zeta_0 \left(2\lambda_Q \delta_{\Delta r}^{-2}\right)^m \delta_{\Delta r}^{s_m - M_{\mathbf{s}_m,\boldsymbol{\sigma}_m}} \|O_{X_0}\|, \tag{876}$$

where ζ_0 *has been defined as* $\zeta_0 = \|O_{X_0}\|$*.*

Proof of Corollary 50. We here define m_0 and m_1 as the numbers of $\{\sigma_j\}_{j=1}^m$ such that $\sigma_j = 0$ and $\sigma_j = 1$, respectively. Note that $m_0 + m_1 = m$. Then, by recursively applying the inequality (864) to $\left\|\Delta\tilde{O}_{\mathbf{s}_m,\boldsymbol{\sigma}_m}\right\|$, we obtain

$$\left\|\Delta\tilde{O}_{\mathbf{s}_m,\boldsymbol{\sigma}_m}\right\| \leq 2^{m_1} \cdot \left(\lambda_Q \delta_{\Delta r}^{-2}\right)^{m_0} \delta_{\Delta r}^{M^{\mathrm{c}}_{\mathbf{s}_m,\boldsymbol{\sigma}_m}} \leq \left(2\lambda_Q \delta_{\Delta r}^{-2}\right)^m \delta_{\Delta r}^{M^{\mathrm{c}}_{\mathbf{s}_m,\boldsymbol{\sigma}_m}} \|O_{X_0}\|, \tag{877}$$

where we define $M^{\mathrm{c}}_{\mathbf{s}_m,\boldsymbol{\sigma}_m}$ as

$$M^{\mathrm{c}}_{\mathbf{s}_m,\boldsymbol{\sigma}_m} := \sum_{j=1}^{m} (1-\sigma_j)(s_j - s_{j-1}). \tag{878}$$

By using $M_{\mathbf{s}_m,\boldsymbol{\sigma}_m} + M^{\mathrm{c}}_{\mathbf{s}_m,\boldsymbol{\sigma}_m} = \sum_{j=1}^m (s_j - s_{j-1}) = s_m - s_0 = s_m$ from $s_0 = 0$, we prove the inequality (876). This completes the proof. □

[**End of Proof of Corollary 50**]

From the above corollary, we can ensure that the norm $\left\|\Delta\tilde{O}_{\mathbf{s}_m,\boldsymbol{\sigma}_m}\right\|$ decays exponentially as $M_{\mathbf{s}_m}$ decreases (i.e., $s_m - M_{\mathbf{s}_m}$ increases). Then, if $M_{\mathbf{s}_{\bar{m}},\boldsymbol{\sigma}_{\bar{m}}} \ll s_m$, the RHS of the inequality (876) is sufficiently small as long as

$$(s_m - M_{\mathbf{s}_{\bar{m}},\boldsymbol{\sigma}_{\bar{m}}})\,\Delta r \gtrsim m \log\left(\lambda_Q \delta_{\Delta r}^{-2}\right).$$

On the other hand, for $M_{\mathbf{s}_{\bar{m}},\boldsymbol{\sigma}_{\bar{m}}} \approx s_m$, the above condition cannot be satisfied unless we set Δr sufficiently large beyond the condition $\Delta r = \Theta[\log^2(N_0)]$ as in (839). To obtain meaningful bound without taking Δr so large, we need another analysis to upper-bound $\left\|\Delta\tilde{O}_{\mathbf{s}_m,\boldsymbol{\sigma}_m}\right\|$. To see the point, let us expand $\Delta\tilde{O}_{\mathbf{s}_m,\boldsymbol{\sigma}_m}$ in Eq. (859). Then, the expanded terms, for example, include

$$\left(U_{s_1}\cdots U_{s_{m-1}}U_{s_m}\right)^\dagger O_{X_0}\left(U_{s_1}P_{0,s_1}^{(\sigma_1)}\cdots U_{s_{m-1}}P_{s_{m-2},s_{m-1}}^{(\sigma_{m-1})}U_{s_m}P_{s_{m-1},s_m}^{(\sigma_m)}\right). \tag{879}$$

In more detail, all the expanded terms are given in the form of

$$\Delta\tilde{O}_{\mathbf{s}_m,\boldsymbol{\sigma}_m}\rho_0 = \sum_{\zeta_1,\zeta_2,\ldots,\zeta_m=0,1}\left(U_{s_1-\zeta_1}\cdots U_{s_{m-1}-\zeta_{m-1}}U_{s_m-\zeta_m}\right)^\dagger O_{X_0}\Phi(\mathbf{s}_m,\boldsymbol{\sigma}_m,\boldsymbol{\zeta}_m)\rho_0$$
$$\Phi(\mathbf{s}_m,\boldsymbol{\sigma}_m,\boldsymbol{\zeta}_m) = \prod_{j=1}^m(-1)^{\zeta_j}U_{s_j-\zeta_j}P_{s_{j-1},s_j}^{(\sigma_j)}, \quad \boldsymbol{\zeta}_m := \{\zeta_1,\zeta_2,\ldots,\zeta_m\} \tag{880}$$

where the term (879) corresponds to the case of $\zeta_j = 0$ for $j = 1,2,\ldots,m$. In the case where $M_{\mathbf{s}_{\bar{m}},\boldsymbol{\sigma}_{\bar{m}}} \ll m$, the operator $\Phi(\mathbf{s}_m,\boldsymbol{\sigma}_m,\boldsymbol{\zeta}_m)$ roughly plays a role of projection onto quantum states with large boson number in the region $(X_{s_m}\setminus X_0[\ell_{t,R}+1]) \subseteq \tilde{X}_0$ [see also Fig. 28 (b)]. Therefore, because the number of bosons is truncated up to Q in the region $\tilde{X}_0$, we expect that

$$\|\Phi(\mathbf{s}_m,\boldsymbol{\sigma}_m,\boldsymbol{\zeta}_m)\rho_0\| \ll 1 \quad \text{for} \quad M_{\mathbf{s}_{\bar{m}},\boldsymbol{\sigma}_{\bar{m}}} \approx s_m. \tag{881}$$

The difficulty stems from the unitary operator $U_{s_j-\zeta_j}$ that is inserted between $P_{s_{j-1},s_j}^{(\sigma_j)}$ and $P_{s_{j-2},s_{j-1}}^{(\sigma_j)}$ [see (884) below].

In the following, we aim to derive a complementary statement to Corollary 50, where the norm $\left\|\Delta\tilde{O}_{\mathbf{s}_m,\boldsymbol{\sigma}_m}\right\|$ decays exponentially as $M_{\mathbf{s}_m}$ increases. Indeed, we can prove the following lemma:

Lemma 51. *The norm of $\Delta O_{\mathbf{s}_m,\boldsymbol{\sigma}_m}\rho_0$ is bounded from above by*

$$\|\Delta O_{\mathbf{s}_m,\boldsymbol{\sigma}_m}\rho_0\|_1 \le 2^{2m}\zeta_0\exp\left[-\Theta(1)\left(\frac{M_{\mathbf{s}_m,\boldsymbol{\sigma}_m}\check{q}\Delta r}{2} - \frac{Q}{8}\right)\right], \tag{882}$$

where $M_{\mathbf{s}_m,\boldsymbol{\sigma}_m}$ has been defined in Eq. (875).

2. Proof of Lemma 51

For arbitrary choices of $\boldsymbol{\zeta}_m$, we aim to prove

$$\|\Phi(\mathbf{s}_m,\boldsymbol{\sigma}_m,\boldsymbol{\zeta}_m)\rho_0\|_1 \le 2^m\exp\left[-\Theta(1)\left(\frac{M_{\mathbf{s}_m,\boldsymbol{\sigma}_m}\check{q}\Delta r}{2} - \frac{Q}{8}\right)\right], \tag{883}$$

which immediately yields the desired inequality (882) by counting all the patterns of $\{\boldsymbol{\zeta}_m\}$ in Eq. (880).

We here consider the case of $\zeta_j = 0$ for $j = 1,2,\ldots,m$ for simplicity, but the same calculations are applied to the other cases. We then need to consider the norm of

$$\left\|U_{s_1}P_{0,s_1}^{(\sigma_1)}\cdots U_{s_{m-1}}P_{s_{m-2},s_{m-1}}^{(\sigma_{m-1})}U_{s_m}P_{s_{m-1},s_m}^{(\sigma_m)}\rho_0\right\|_1. \tag{884}$$

If we simply consider the product of the projections $\{P_{s_{j-1},s_j}^{(\sigma_j)}\}_{j=1}^m$, it becomes a projection onto a region $X_m\setminus X_0[\bar{\ell}_{t,R}+1]$ with a large boson number. However, due to the unitary operators $\{U_{s_j}\}_{j=1}^m$, such a simple discussion cannot be applied. For example, let us consider the case of $\sigma_m = 1$ (see also Fig. 29). Then, by the projection $P_{s_{m-1},s_m}^{(1)}$, a quantum state $P_{s_{m-1},s_m}^{(1)}|\psi\rangle$ has a large boson number in the region $X_{s_m}\setminus X_{s_{m-1}}$. However, because of the unitary operator U_{s_m}, the bosons in $X_{s_m}\setminus X_{s_{m-1}}$ may outflow from the region, and hence the quantum state $U_{s_m}P_{s_{m-1},s_m}^{(1)}|\psi\rangle$ may no longer have many bosons in $X_{s_m}\setminus X_{s_{m-1}}$.

To avoid the above difficulty, we decompose $U_{s_j}P_{s_{j-1},s_j}^{(1)}$ as follows:

$$U_{s_j}P_{s_{j-1},s_j}^{(1)} = \left(\tilde{P}_{s_{j-1},s_j}^{(1,0)} + \tilde{P}_{s_{j-1},s_j}^{(1,1)}\right)U_{s_j}P_{s_{j-1},s_j}^{(1)}, \tag{885}$$

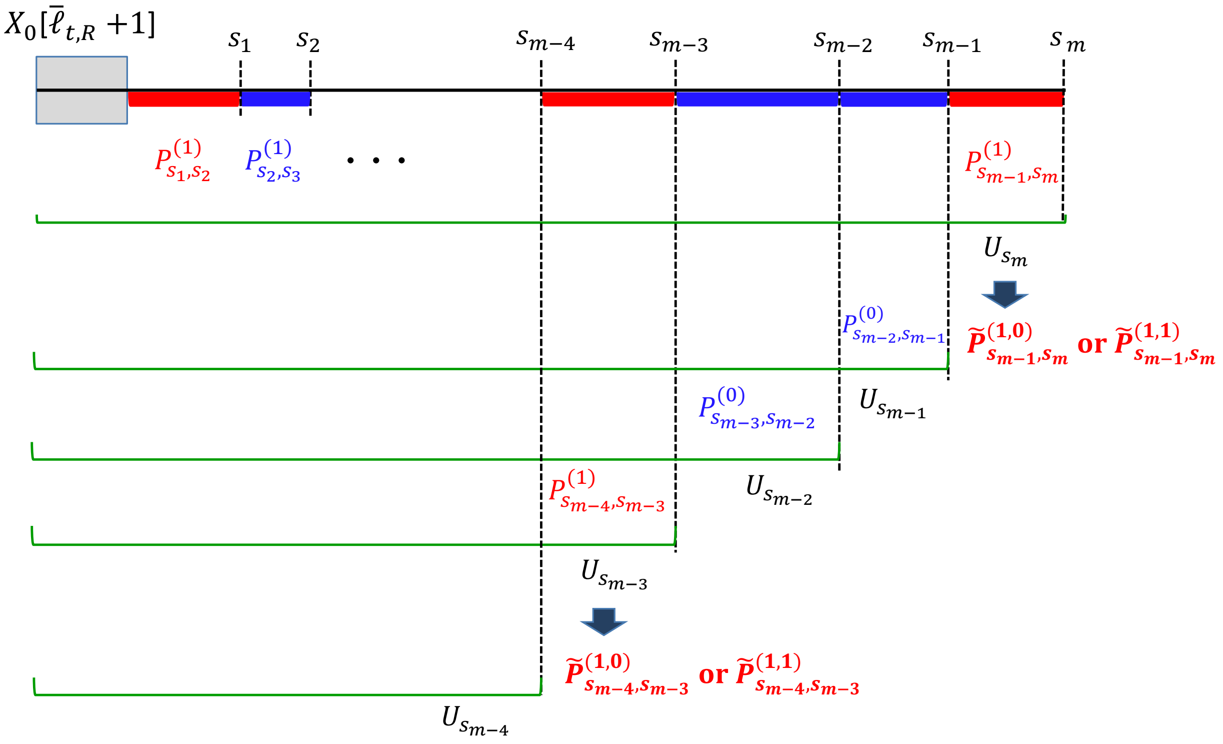

Supplementary Figure 29. Schematic picture of the decomposition of $U_{s_j}P^{(1)}_{s_{j-1},s_j}$. We have mentioned that the operator $\Phi(\mathbf{s}_m,\boldsymbol{\sigma}_m,\boldsymbol{\zeta}_m)$ in Eq. (880) roughly plays a role in projection onto states with a large boson number. However, we cannot apply this intuitive discussion due to the outflow of bosons by the unitary operators $\{U_{s_j}\}_{j=1}^m$. To identify the influence of the unitary operators quantitatively, we introduce new projections $\tilde{P}^{(1,0)}_{s_{j-1},s_j}$ and $\tilde{P}^{(1,1)}_{s_{j-1},s_j}$ after $U_{s_j}P^{(1)}_{s_{j-1},s_j}$. The projection $\tilde{P}^{(1,0)}_{s_{j-1},s_j}$ characterizes the case that the over half of the bosons outflow from the region $X_{s_j}\setminus X_{s_{j-1}}$, while the projection $\tilde{P}^{(1,1)}_{s_{j-1},s_j}$ characterizes the case that many of the bosons still remains in the region $X_{s_j}\setminus X_{s_{j-1}}$.

where $\tilde{P}^{(1,0)}_{s_{j-1},s_j}$ and $\tilde{P}^{(1,1)}_{s_{j-1},s_j}$ are defined as follows (see Fig. 29):

$$\begin{aligned}\tilde{P}^{(1,0)}_{s_{j-1},s_j} &= \Pi_{X_{s_j}\setminus X_{s_{j-1}},\le 8\check{q}|L_j|},\\ \tilde{P}^{(1,1)}_{s_{j-1},s_j} &= \Pi_{X_{s_j}\setminus X_{s_{j-1}},> 8\check{q}|L_j|}, \quad 8\check{q}|L_j| = 4\check{q}\Delta r(s_j-s_{j-1}),\end{aligned} \tag{886}$$

where L_j has been defined in Eq. (863), i.e., $L_j := X_{s_j}\setminus X_{s_{j-1}}[(s_j-s_{j-1})\Delta r/2]$. Note that original projection $P^{(1)}_{s_{j-1},s_j}$ ensures at least $16\check{q}|L_j|$ bosons in the region L_j as in Eq. (863). Hence, the projection $\tilde{P}^{(1,0)}_{s_{j-1},s_j}$ implies that over half of the bosons outflows from the region $X_{s_j}\setminus X_{s_{j-1}}$, while the projection $\tilde{P}^{(1,1)}_{s_{j-1},s_j}$ means that many of the bosons still remains in the region $X_{s_j}\setminus X_{s_{j-1}}$.

Because the projection $\tilde{P}^{(1,0)}_{s_{j-1},s_j}$ is supported on $X_{s_j}\setminus X_{s_{j-1}}$, it commutes with $U_{s_{j'}}P^{(\sigma_{j'})}_{s_{j'-1},s_{j'}}$ for $\forall j'\le j-1$ (see also Fig. 29):

$$\left[\tilde{P}^{(1,\theta_j)}_{s_{j-1},s_j}, U_{s_{j'}}P^{(\sigma_{j'})}_{s_{j'-1},s_{j'}}\right]=0 \quad \text{for} \quad \forall j'\le j-1, \quad \theta_j=0,1. \tag{887}$$

For the fixed $\{\sigma_j\}_{j=1}^m$, we define the indices $\{j_1,j_2,\ldots,j_{m_1}\}$ such that

$$\sigma_{j_1}=\sigma_{j_2}=\cdots=\sigma_{j_{m_1}}=1. \tag{888}$$

To see how to obtain an upper bound of $\left\|U_{s_1}P^{(\sigma_1)}_{0,s_1}\cdots U_{s_{m-1}}P^{(\sigma_{m-1})}_{s_{m-2},s_{m-1}}U_{s_m}P^{(\sigma_m)}_{s_{m-1},s_m}\rho_0\right\|_1$ using Eq. (887), let us consider a simple case of $m=4$ and the norm $\left\|U_{s_1}P^{(0)}_{0,s_1}U_{s_2}P^{(1)}_{s_1,s_2}U_{s_3}P^{(0)}_{s_2,s_3}U_{s_4}P^{(1)}_{s_3,s_4}\rho_0\right\|_1$ ($\sigma_1=\sigma_3=0$ and $\sigma_2=\sigma_4=1$). We first obtain

$$\left\|U_{s_1}P^{(0)}_{0,s_1}U_{s_2}P^{(1)}_{s_1,s_2}U_{s_3}P^{(0)}_{s_2,s_3}U_{s_4}P^{(1)}_{s_3,s_4}\rho_0\right\|_1=\left\|\sum_{\theta_2,\theta_4}U_{s_1}P^{(0)}_{0,s_1}\tilde{P}^{(1,\theta_2)}_{s_1,s_2}U_{s_2}P^{(1)}_{s_1,s_2}U_{s_3}P^{(0)}_{s_2,s_3}\tilde{P}^{(1,\theta_4)}_{s_3,s_4}U_{s_4}P^{(1)}_{s_3,s_4}\rho_0\right\|_1. \tag{889}$$

Then, because the Hilbert space has been restricted by the projections $\Pi_{\mathcal{S}(i_0[R],1/2,N_0)}$ and $\Pi_{\tilde{X}_0,\le Q}$ [see Eq. (828)],

we have the following two inequalities:

$$\begin{aligned}
&\left\| U_{s_1} P^{(0)}_{0,s_1} \tilde{P}^{(1,\theta_2)}_{s_1,s_2} U_{s_2} P^{(1)}_{s_1,s_2} U_{s_3} P^{(0)}_{s_2,s_3} \tilde{P}^{(1,\theta_4)}_{s_3,s_4} U_{s_4} P^{(1)}_{s_3,s_4} \rho_0 \right\|_1 \\
&= \left\| \Pi_{\tilde{X}_0,\le Q} \tilde{P}^{(1,\theta_2)}_{s_1,s_2} \tilde{P}^{(1,\theta_4)}_{s_3,s_4} U_{s_1} P^{(0)}_{0,s_1} P^{(1)}_{s_1,s_2} U_{s_2} U_{s_3} P^{(0)}_{s_2,s_3} U_{s_4} P^{(1)}_{s_3,s_4} \rho_0 \right\|_1 \\
&\le \left\| \Pi_{\tilde{X}_0,\le Q} \tilde{P}^{(1,\theta_2)}_{s_1,s_2} \tilde{P}^{(1,\theta_4)}_{s_3,s_4} \right\| \cdot \left\| U_{s_1} P^{(0)}_{0,s_1} P^{(1)}_{s_1,s_2} U_{s_2} U_{s_3} P^{(0)}_{s_2,s_3} U_{s_4} P^{(1)}_{s_3,s_4} \rho_0 \right\|_1 \le \left\| \Pi_{\tilde{X}_0,\le Q} \tilde{P}^{(1,\theta_2)}_{s_1,s_2} \tilde{P}^{(1,\theta_4)}_{s_3,s_4} \right\|
\end{aligned} \tag{890}$$

and

$$\begin{aligned}
&\left\| U_{s_1} P^{(0)}_{0,s_1} \tilde{P}^{(1,\theta_2)}_{s_1,s_2} U_{s_2} P^{(1)}_{s_1,s_2} U_{s_3} P^{(0)}_{s_2,s_3} \tilde{P}^{(1,\theta_4)}_{s_3,s_4} U_{s_4} P^{(1)}_{s_3,s_4} \rho_0 \right\|_1 \\
&\le \left\| U_{s_1} P^{(0)}_{0,s_1} \right\| \cdot \left\| \tilde{P}^{(1,\theta_2)}_{s_1,s_2} U_{s_2} P^{(1)}_{s_1,s_2} \Pi_{\mathcal{S}(i_0[R],1/2,N_0)} \right\| \cdot \left\| U_{s_3} P^{(0)}_{s_2,s_3} \right\| \cdot \left\| \tilde{P}^{(1,\theta_4)}_{s_3,s_4} U_{s_4} P^{(1)}_{s_3,s_4} \Pi_{\mathcal{S}(i_0[R],1/2,N_0)} \right\| \\
&\le \left\| \tilde{P}^{(1,\theta_2)}_{s_1,s_2} U_{s_2} P^{(1)}_{s_1,s_2} \Pi_{\mathcal{S}(i_0[R],1/2,N_0)} \right\| \cdot \left\| \tilde{P}^{(1,\theta_4)}_{s_3,s_4} U_{s_4} P^{(1)}_{s_3,s_4} \Pi_{\mathcal{S}(i_0[R],1/2,N_0)} \right\| ,
\end{aligned} \tag{891}$$

where we use the commutation relation (887) in the first inequality. We then obtain

$$\begin{aligned}
&\left\| U_{s_1} P^{(0)}_{0,s_1} U_{s_2} P^{(1)}_{s_1,s_2} U_{s_3} P^{(0)}_{s_2,s_3} U_{s_4} P^{(1)}_{s_3,s_4} \rho_0 \right\|_1 \\
&\le \sum_{\theta_2,\theta_4} \min \left(\left\| \Pi_{\tilde{X}_0,\le Q} \tilde{P}^{(1,\theta_2)}_{s_1,s_2} \tilde{P}^{(1,\theta_4)}_{s_3,s_4} \right\| , \left\| \tilde{P}^{(1,\theta_2)}_{s_1,s_2} U_{s_2} P^{(1)}_{s_1,s_2} \Pi_{\mathcal{S}(i_0[R],1/2,N_0)} \right\| \cdot \left\| \tilde{P}^{(1,\theta_4)}_{s_3,s_4} U_{s_4} P^{(1)}_{s_3,s_4} \Pi_{\mathcal{S}(i_0[R],1/2,N_0)} \right\| \right) .
\end{aligned}$$

By generalizing the above inequality, we can derive

$$\begin{aligned}
&\left\| U_{s_1} P^{(\sigma_1)}_{0,s_1} \cdots U_{s_{m-1}} P^{(\sigma_{m-1})}_{s_{m-2},s_{m-1}} U_{s_m} P^{(\sigma_m)}_{s_{m-1},s_m} \rho_0 \right\|_1 \\
&\le \sum_{\theta_{j_1},\theta_{j_2}\cdots\theta_{j_{m_1}}=0,1} \min \left(\left\| \prod_{p=1}^{m_1} \tilde{P}^{(1,\theta_{j_p})}_{s_{j_p-1},s_{j_p}} \Pi_{\tilde{X}_0,\le Q} \right\| , \prod_{p=1}^{m_1} \left\| \tilde{P}^{(1,\theta_{j_p})}_{s_{j_p-1},s_{j_p}} U_{s_{j_p}} P^{(1)}_{s_{j_p-1},s_{j_p}} \Pi_{\mathcal{S}(i_0[R],1/2,N_0)} \right\| \right) .
\end{aligned} \tag{892}$$

In the following, we consider the terms

$$\left\| \prod_{p=1}^{m_1} \tilde{P}^{(1,\theta_{j_p})}_{s_{j_p-1},s_{j_p}} \Pi_{\tilde{X}_0,\le Q} \right\| \quad \text{and} \quad \prod_{p=1}^{m_1} \left\| \tilde{P}^{(1,\theta_{j_p})}_{s_{j_p-1},s_{j_p}} U_{s_{j_p}} P^{(1)}_{s_{j_p-1},s_{j_p}} \Pi_{\mathcal{S}(i_0[R],1/2,N_0)} \right\| , \tag{893}$$

separately.

We first consider the first term in (893). We here define $Q(\theta_1,\dots,\theta_{m_1})$ as

$$Q(\theta_{j_1},\dots,\theta_{j_{m_1}}) = \sum_{p=1}^{m_1} 8\theta_{j_p} \check{q} |L_{j_p}|. \tag{894}$$

Then, from the definition (886), the produce of the projections $\prod_{p=1}^{m_1} \tilde{P}^{(1,\theta_{j_p})}_{s_{j_p-1},s_{j_p}}$ ensures at least more than $Q(\theta_1,\dots,\theta_{m_1})$ bosons in the region $X_{s_m} \setminus X_0[\bar{\ell}_{t,R}+1] \subseteq \tilde{X}_0$. Therefore, we have

$$\prod_{p=1}^{m_1} \tilde{P}^{(1,\theta_{j_p})}_{s_{j_p-1},s_{j_p}} \Pi_{\tilde{X}_0,\le Q(\theta_1,\dots,\theta_{m_1})} = 0, \tag{895}$$

which yields the equation of

$$\left\| \prod_{p=1}^{m_1} \tilde{P}^{(1,\theta_{j_p})}_{s_{j_p-1},s_{j_p}} \Pi_{\tilde{X}_0,\le Q} \right\| = 0 \quad \text{for} \quad Q(\theta_1,\dots,\theta_{m_1}) \ge Q. \tag{896}$$

We next consider the second term in (893). In the estimation, we can apply Proposition 22 to the norm of (see Fig. 30)

$$\left\| \tilde{P}^{(1,0)}_{s_{j-1},s_j} U_{s_j} P^{(1)}_{s_{j-1},s_j} \Pi_{\mathcal{S}(i_0[R],1/2,N_0)} \right\| . \tag{897}$$

For an arbitrary quantum state $|\psi\rangle$, the quantum state $|\tilde{\psi}\rangle = P^{(1)}_{s_{j-1},s_j} \Pi_{\mathcal{S}(i_0[R],1/2,N_0)} |\psi\rangle$ satisfies

$$\Pi_{\mathcal{S}(i_0[R],1/2,N_0)} |\tilde{\psi}\rangle = |\tilde{\psi}\rangle, \quad \Pi_{L_j,>16\check{q}|L_j|} |\tilde{\psi}\rangle = |\tilde{\psi}\rangle. \tag{898}$$

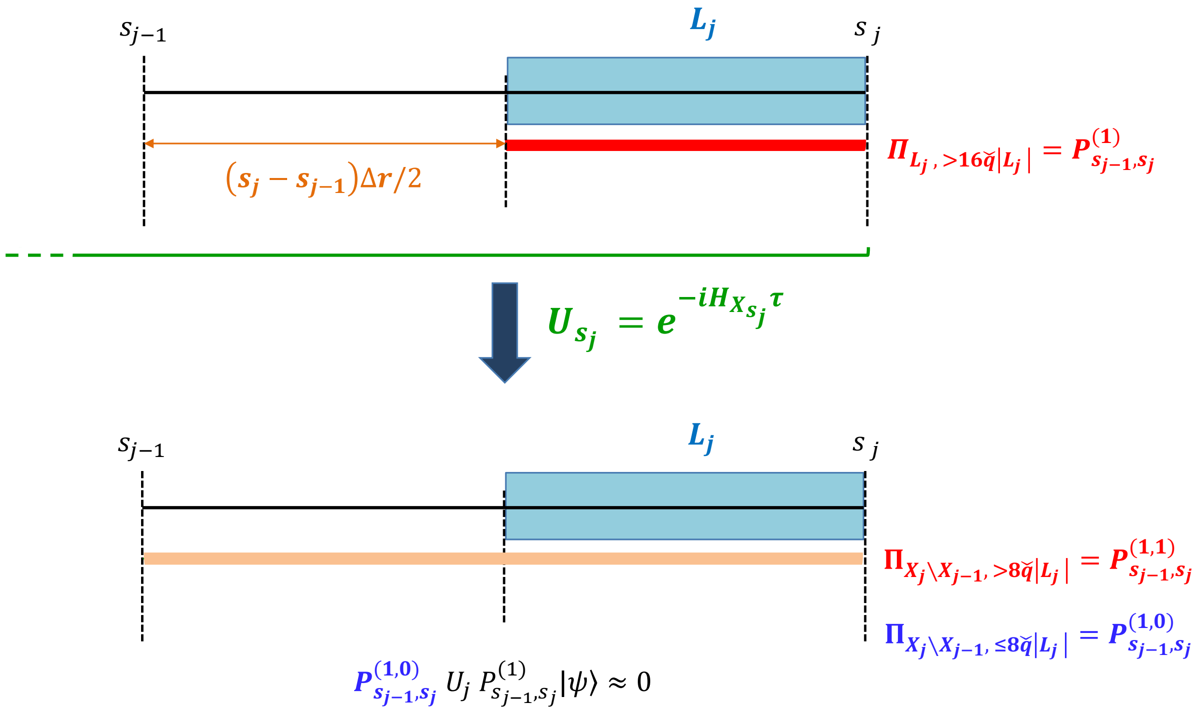

Supplementary Figure 30. Estimation of the norm (897), i.e., $||\tilde{P}^{(1,0)}_{s_{j-1},s_j} U_{s_j} P^{(1)}_{s_{j-1},s_j} \Pi_{\mathcal{S}(i_0[R],1/2,N_0)}||$. The setup is similar to the one in Proposition 22. We consider the setup that a quantum state initially has more than $16\check{q}|L_j|$ bosons in the region L_j. Then, the unitary operator U_{s_j} induces the outflow of the bosons from the region L_j. Proposition 22 provides the probability of the number of bosons that still remain in the region $X_{s_j} \setminus X_{s_{j-1}}$. From the statement, we can ensure that the probability to observe less than $8\check{q}|L_j|$ bosons is smaller than $e^{-\Theta(\check{q}|L_j|)}$ as in the inequality (899).

By using the inequality (359) with

$$N \to 16\check{q}|L_j|, \quad q_* \to N_0, \quad \delta N \to 8\check{q}|L_j|, \quad X \to L_j, \quad \ell \to \frac{(s_j - s_{j-1})\Delta r}{2},$$

we obtain for the time evolution $U_{s_j} = e^{-iH_{X_{s_j}}\tau}$

$$\left\|\Pi_{X_j\setminus X_{j-1},\le 8\check{q}|L_j|} U_{s_j}|\tilde{\psi}\rangle\right\|^2 \le \frac{2}{1-e^{-1/(8ec_{\tau,2})}} \exp\left[\frac{-8\check{q}|L_j|}{8ec_{\tau,2}} + 3c'_\ell\left(1+c'_\ell D\right)\log^2\left(N_0\gamma|L_j|\right)\right] \tag{899}$$

as long as the following condition from Eq. (360) is satisfied:

$$\frac{(s_j - s_{j-1})\Delta r}{2} \ge \left\lceil \frac{2}{\tilde{f}_\tau}\log\left(\frac{5c_{\tau,1}\lambda_{1/2}e^{2\tilde{f}_\tau}\gamma(D/\tilde{f}_\tau)^D N_0|L_j|}{2c_{\tau,2}}\right)\right\rceil = c'_\ell \log\left(N_0\gamma|L_j|\right),$$
$$\tilde{f}_\tau := \frac{1}{2}\log\left(1+\frac{1}{10ec_{\tau,1}\lambda_{1/2}+2}\right). \tag{900}$$

Because of the condition (839), i.e., $\Delta r = \Theta[\log^{1+\kappa}(N_0)]$, the condition (900) is satisfied.

By applying the inequality (899) to the norm (897), we have

$$\left\|\tilde{P}^{(1,0)}_{s_{j-1},s_j} U_{s_j} P^{(1)}_{s_{j-1},s_j} \Pi_{\mathcal{S}(i_0[R],1/2,N_0)}\right\| = \sup_{\psi} \left\|\Pi_{X_j\setminus X_{j-1},\le 8\check{q}|L_j|} U_{s_j}|\tilde{\psi}\rangle\right\| \le e^{-\Theta(1)\check{q}|L_j|}. \tag{901}$$

On the other hand, for $\theta_j = 1$, we only obtain the trivial inequality of

$$\left\|\tilde{P}^{(1,\theta_j)}_{s_{j-1},s_j} U_{s_j} P^{(1)}_{s_{j-1},s_j} \Pi_{\mathcal{S}(i_0[R],1/2,N_0)}\right\| \le 1 \quad \text{for} \quad \theta_j = 1. \tag{902}$$

By applying the inequalities (901) and (902) to the second term in (893), we obtain the following upper bound:

$$\prod_{p=1}^{m_1} \left\|\tilde{P}^{(1,\theta_{j_p})}_{s_{j_p-1},s_{j_p}} U_{s_{j_p}} P^{(1)}_{s_{j_p-1},s_{j_p}} \Pi_{\mathcal{S}(i_0[R],1/2,N_0)}\right\| \le \exp\left[-\Theta(1)\sum_{p=1}^{m_1}\left(1-\theta_{j_p}\right)\check{q}|L_{j_p}|\right]$$
$$\le \exp\left[-\Theta(1)\left(\frac{M_{\mathbf{s}_m,\boldsymbol{\sigma}_m}\check{q}\Delta r}{2} - \frac{Q(\theta_1,\ldots,\theta_{m_1})}{8}\right)\right], \tag{903}$$

where we use the definition (894) and the inequality of

$$\sum_{p=1}^{m_1} |L_{j_p}| = \frac{\Delta r}{2} \sum_{p=1}^{m_1} (s_{j_p} - s_{j_p-1}) = \frac{\Delta r}{2} \sum_{j=1}^{m} \sigma_j (s_j - s_{j-1}) = \frac{M_{\mathbf{s}_m,\boldsymbol{\sigma}_m} \Delta r}{2}. \tag{904}$$

Note that we have defined as in Eqs. (888) and (875).

By combining the inequalities (896) and (903), we reduce the inequality (892) to

$$\begin{aligned} &\left\| U_{s_1} P_{0,s_1}^{(\sigma_1)} \cdots U_{s_{m-1}} P_{s_{m-2},s_{m-1}}^{(\sigma_{m-1})} U_{s_m} P_{s_{m-1},s_m}^{(\sigma_m)} \rho_0 \right\|_1 \\ &\le \sum_{\theta_{j_1},\theta_{j_2}\cdots\theta_{j_{m_1}}=0,1} \exp\left[-\Theta(1)\left(\frac{M_{\mathbf{s}_m,\boldsymbol{\sigma}_m} \check{q} \Delta r}{2} - \frac{Q}{8}\right)\right] \le 2^m \exp\left[-\Theta(1)\left(\frac{M_{\mathbf{s}_m,\boldsymbol{\sigma}_m} \check{q} \Delta r}{2} - \frac{Q}{8}\right)\right], \end{aligned} \tag{905}$$

where we use $\sum_{\theta_{j_1},\theta_{j_2}\cdots\theta_{j_{m_1}}=0,1} 1 \le 2^{m_1} \le 2^m$. We thus prove the inequality (883). This completes the proof. □

——————————————————————————[**End of Proof of Lemma 51**]

F. Proof of Proposition 48: completing the proof

We have finally obtained all the ingredients to prove Proposition 48. By combining Corollary 50 and Lemma 51, we obtain

$$\begin{aligned} \|\Delta O_{\mathbf{s}_m,\boldsymbol{\sigma}_m} \rho_0\|_1 &\le \zeta_0 \min\left(2^{2m} \exp\left[-\Theta(1)\left(\frac{M_{\mathbf{s}_m,\boldsymbol{\sigma}_m} \check{q} \Delta r}{2} - \frac{Q}{8}\right)\right], \left(2\lambda_Q \delta_{\Delta r}^{-2}\right)^m \delta_{\Delta r}^{s_m - M_{\mathbf{s}_m,\boldsymbol{\sigma}_m}}\right) \\ &\le \zeta_0 \max\left(2^{2m} \exp\left[-\Theta(1)\left(\frac{\check{q} s_m \Delta r}{4} - \frac{\check{q} R''}{8}\right)\right], \left(2\lambda_Q \delta_{\Delta r}^{-2}\right)^m \delta_{\Delta r}^{s_m/2}\right), \end{aligned} \tag{906}$$

where we use $Q = \check{q} R''$ as in Eq. (823). Note that the condition $0 \le M_{\mathbf{s}_m,\boldsymbol{\sigma}_m} \le s_m$ from the definition (875) implies that either $M_{\mathbf{s}_m,\boldsymbol{\sigma}_m}$ or $(s_m - M_{\mathbf{s}_m,\boldsymbol{\sigma}_m})$ is greater than $s_m/2$.

We now consider the case of $m = \bar{m}$ and $\mathbf{s}_{\bar{m}} \in V_{\bar{s}+1}$ [see (861)]. Then, we can replace s_m with $\bar{s}$ in the inequality (906). Also, because of the definition of Δr in Eq. (833), we have

$$\Delta r \ge \frac{R''}{\bar{s}} - 1 \longrightarrow \bar{s} \Delta r \ge R'' - \bar{s}. \tag{907}$$

Then, the above inequality reduces the inequality (906) to

$$\begin{aligned} \|\Delta O_{\mathbf{s}_{\bar{m}},\boldsymbol{\sigma}_{\bar{m}}} \rho_0\|_1 &\le \zeta_0 \max\left(2^{2\bar{m}} \exp\left[\Theta(1)\left(\frac{-\check{q} R'' + 2\check{q}\bar{s}}{8}\right)\right], \left(2\lambda_Q \delta_{\Delta r}^{-2}\right)^{\bar{m}} \delta_{\Delta r}^{\bar{s}/2}\right) \\ &= \zeta_0 \left(2\lambda_Q \delta_{\Delta r}^{-2}\right)^{\bar{m}} \delta_{\Delta r}^{\bar{s}/2} \end{aligned} \tag{908}$$

where $\left(2\lambda_Q \delta_{\Delta r}^{-2}\right)^{\bar{m}} \delta_{\Delta r}^{\bar{s}/2}$ is larger than $e^{-\Theta(\check{q} R'')}$ because of $\delta_{\Delta r}^{\bar{s}/2} = e^{-\Theta(R'')}$ from the definition (865), i.e., $\delta_{\Delta r} = e^{-\Theta(\Delta r)}$. By applying the above inequality to (861), we obtain

$$\begin{aligned} \left\| \left[O_{X_0}(t) - \bar{O}_{X_{\bar{s}}}\right] \rho_0 \right\|_1 &\le \sum_{\boldsymbol{\sigma}_{\bar{m}} \in \{0,1\}^{\otimes \bar{m}}} \sum_{\mathbf{s}_{\bar{m}} \in V_{\bar{s}+1}} \zeta_0 \left(2\lambda_Q \delta_{\Delta r}^{-2}\right)^{\bar{m}} \delta_{\Delta r}^{\bar{s}/2} \\ &\le \zeta_0 \left[4(\bar{s}+1)\lambda_Q \delta_{\Delta r}^{-2}\right]^{\bar{m}} \delta_{\Delta r}^{\bar{s}/2}. \end{aligned} \tag{909}$$

Finally, by choosing $\bar{s} = 8\bar{m}$ in the inequality (909), we arrive at the desired inequality of

$$\left\| \left[O_{X_0}(t) - \bar{O}_{X_{\bar{s}}}\right] \rho_0 \right\|_1 \le \zeta_0 \exp\left\{\bar{m} \log\left[4(\bar{s}+1)\lambda_Q\right] - 2\bar{m} \log(\delta_{\Delta r})\right\} = \zeta_0 e^{\Theta[t \log(N_0)] - \Theta(R'')}, \tag{910}$$

where we use $\log(\lambda_Q) = \Theta[\log(N_0)]$ from Eq. (865) and $\bar{m} \log(\delta_{\Delta r}) = \bar{m}\Theta(\Delta r) = \Theta(R'')$. This completes the proof of Proposition 48. □

Supplementary Note 11. POSSIBLE SCENARIO FOR ACCELERATION BY TIME-INDEPENDENT HAMILTONIAN

Finally, we discuss the possibility of realizing the acceleration of information propagation through more natural Hamiltonian dynamics. In the protocol of the main manuscript, we employed a highly artificial Hamiltonian by

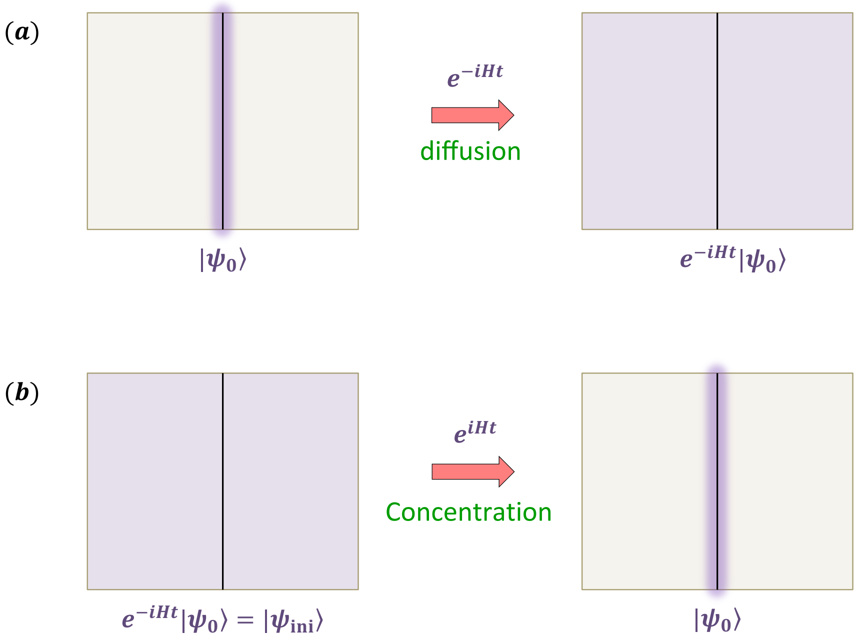

Supplementary Figure 31. In process (a), the bosons diffuse, and the state transitions to a steady state with low-boson density. By reversing the time evolution, we induce boson concentration (b). During this process, the acceleration of information propagation can occur.

fine-tuning local interactions. Therefore, considering the standard Bose-Hubbard Hamiltonian with a simple initial state (e.g., the Mott state), it is natural to expect that the linear light cone should be established, and acceleration no longer occurs. Additionally, even if a 1D information path with high boson densities is realized, it should not persist for a long time under Hamiltonian dynamics with repulsive interactions. However, these simple intuitions are extremely challenging to make mathematically rigorous[*14].

Here, we demonstrate that a special choice of the initial state can induce the acceleration of information propagation. To illustrate this, we first highlight the essence of acceleration, summarized by the following two points: i) concentrating bosons on a particular one-dimensional region, ii) the speed of information propagation is proportional to local boson numbers (see Ref. [11]). While the first step is unlikely in natural dynamics, using reverse time evolution allows us to consider a situation where bosons automatically concentrate on a one-dimensional region (Fig. 31):

1. We first consider a quantum state $|\psi_0\rangle$ that has an information path with high boson densities on a 1D region.

2. We then evolve the state by a translation-invariant Bose-Hubbard Hamiltonian, resulting in a steady-state $e^{-iHt}|\psi_0\rangle$ after a long time.

3. We choose the state $e^{-iHt}|\psi_0\rangle$ as our initial state of interest $|\psi_{\rm ini}\rangle$, which is expected to satisfy the low-boson density condition.

4. Considering the Hamiltonian $-H$, the time evolution $e^{iHt}|\psi_{\rm ini}\rangle$ yields boson concentration onto the one-dimensional path.

Importantly, the initial state $|\psi_{\rm ini}\rangle$ is quite rare in the whole phase space, but we always have to exclude the possibility that the initial state of interest is not included in this class of states. In the above situation, it might be possible to observe the acceleration of information propagation in the above setup.

At this stage, the computational cost for the numerical simulation of the above dynamics is quite substantial. However, it remains an intriguing open question to identify whether we can observe the acceleration of information propagation under the above dynamics or other natural situations.

[*14] Indeed, even for the homogeneous Bose-Hubbard model with the Mott state as the initial state, there have been no mathematical tools to treat information propagation in general.

SUPPLEMENTARY REFERENCES

[1] T. Kuwahara and K. Saito, *Lieb-Robinson Bound and Almost-Linear Light Cone in Interacting Boson Systems*, Phys. Rev. Lett. **127**, 070403 (2021).

[2] N. Schuch, S. K. Harrison, T. J. Osborne, and J. Eisert, *Information propagation for interacting-particle systems*, Phys. Rev. A **84**, 032309 (2011).

[3] H. Finner, *A Generalization of Holder's Inequality and Some Probability Inequalities*, The Annals of Probability **20**, 1893 (1992), full publication date: Oct., 1992.

[4] D. Sutter, *Approximate quantum Markov chains*, arXiv preprint arXiv:1802.05477 (2018), arXiv:1802.05477.

[5] T. Kuwahara, *Fundamental inequalities in quantum many-body systems*, PhD thesis, University of Tokyo (2015).

[6] M. B. Hastings and T. Koma, *Spectral Gap and Exponential Decay of Correlations*, Communications in Mathematical Physics **265**, 781 (2006).

[7] B. Nachtergaele, Y. Ogata, and R. Sims, *Propagation of Correlations in Quantum Lattice Systems*, Journal of Statistical Physics **124**, 1 (2006).

[8] T. Kuwahara, *Exponential bound on information spreading induced by quantum many-body dynamics with long-range interactions*, New Journal of Physics **18**, 053034 (2016).

[9] T. Kuwahara and K. Saito, *Absence of Fast Scrambling in Thermodynamically Stable Long-Range Interacting Systems*, Phys. Rev. Lett. **126**, 030604 (2021).

[10] C. Yin and A. Lucas, *Finite Speed of Quantum Information in Models of Interacting Bosons at Finite Density*, Phys. Rev. X **12**, 021039 (2022).

[11] P. Barmettler, D. Poletti, M. Cheneau, and C. Kollath, *Propagation front of correlations in an interacting Bose gas*, Phys. Rev. A **85**, 053625 (2012).
